# The genomes of two key bumblebee species with primitive eusocial organization

**DOI:** 10.1186/s13059-015-0623-3

**Published:** 2015-04-24

**Authors:** Ben M Sadd, Seth M Barribeau, Guy Bloch, Dirk C de Graaf, Peter Dearden, Christine G Elsik, Jürgen Gadau, Cornelis JP Grimmelikhuijzen, Martin Hasselmann, Jeffrey D Lozier, Hugh M Robertson, Guy Smagghe, Eckart Stolle, Matthias Van Vaerenbergh, Robert M Waterhouse, Erich Bornberg-Bauer, Steffen Klasberg, Anna K Bennett, Francisco Câmara, Roderic Guigó, Katharina Hoff, Marco Mariotti, Monica Munoz-Torres, Terence Murphy, Didac Santesmasses, Gro V Amdam, Matthew Beckers, Martin Beye, Matthias Biewer, Márcia MG Bitondi, Mark L Blaxter, Andrew FG Bourke, Mark JF Brown, Severine D Buechel, Rossanah Cameron, Kaat Cappelle, James C Carolan, Olivier Christiaens, Kate L Ciborowski, David F Clarke, Thomas J Colgan, David H Collins, Andrew G Cridge, Tamas Dalmay, Stephanie Dreier, Louis du Plessis, Elizabeth Duncan, Silvio Erler, Jay Evans, Tiago Falcon, Kevin Flores, Flávia CP Freitas, Taro Fuchikawa, Tanja Gempe, Klaus Hartfelder, Frank Hauser, Sophie Helbing, Fernanda C Humann, Frano Irvine, Lars S Jermiin, Claire E Johnson, Reed M Johnson, Andrew K Jones, Tatsuhiko Kadowaki, Jonathan H Kidner, Vasco Koch, Arian Köhler, F Bernhard Kraus, H Michael G Lattorff, Megan Leask, Gabrielle A Lockett, Eamonn B Mallon, David S Marco Antonio, Monika Marxer, Ivan Meeus, Robin FA Moritz, Ajay Nair, Kathrin Näpflin, Inga Nissen, Jinzhi Niu, Francis MF Nunes, John G Oakeshott, Amy Osborne, Marianne Otte, Daniel G Pinheiro, Nina Rossié, Olav Rueppell, Carolina G Santos, Regula Schmid-Hempel, Björn D Schmitt, Christina Schulte, Zilá LP Simões, Michelle PM Soares, Luc Swevers, Eva C Winnebeck, Florian Wolschin, Na Yu, Evgeny M Zdobnov, Peshtewani K Aqrawi, Kerstin P Blankenburg, Marcus Coyle, Liezl Francisco, Alvaro G Hernandez, Michael Holder, Matthew E Hudson, LaRonda Jackson, Joy Jayaseelan, Vandita Joshi, Christie Kovar, Sandra L Lee, Robert Mata, Tittu Mathew, Irene F Newsham, Robin Ngo, Geoffrey Okwuonu, Christopher Pham, Ling-Ling Pu, Nehad Saada, Jireh Santibanez, DeNard Simmons, Rebecca Thornton, Aarti Venkat, Kimberly KO Walden, Yuan-Qing Wu, Griet Debyser, Bart Devreese, Claire Asher, Julie Blommaert, Ariel D Chipman, Lars Chittka, Bertrand Fouks, Jisheng Liu, Meaghan P O’Neill, Seirian Sumner, Daniela Puiu, Jiaxin Qu, Steven L Salzberg, Steven E Scherer, Donna M Muzny, Stephen Richards, Gene E Robinson, Richard A Gibbs, Paul Schmid-Hempel, Kim C Worley

**Affiliations:** School of Biological Sciences, Illinois State University, Normal, IL 61790 USA; Experimental Ecology, Institute of Integrative Biology, Eidgenössiche Technische Hochschule (ETH) Zürich, CH-8092 Zürich, Switzerland; Department of Biology, East Carolina University, Greenville, NC 27858 USA; Department of Ecology, Evolution, and Behavior, The Alexander Silberman Institute of Life Sciences, The Hebrew University of Jerusalem, Jerusalem, Israel; Laboratory of Zoophysiology, Faculty of Sciences, Ghent University, Krijgslaan 281, S2, 9000 Ghent, Belgium; Laboratory for Evolution and Development, Genetics Otago and the National Research Centre for Growth and Development, Department of Biochemistry, University of Otago, P.O. Box 56, Dunedin, 9054 New Zealand; Division of Animal Sciences, Division of Plant Sciences, and MU Informatics Institute, University of Missouri, Columbia, MO 65211 USA; Department of Biology, Georgetown University, Washington, DC 20057 USA; School of Life Sciences, Arizona State University, Tempe, AZ 85287 USA; Center for Functional and Comparative Insect Genomics, Department of Biology, University of Copenhagen, Copenhagen, Denmark; University of Hohenheim, Institute of Animal Science, Garbenstrasse 17, 70599 Stuttgart, Germany; Department of Biological Sciences, University of Alabama, Tuscaloosa, AL 35487 USA; Department of Entomology, University of Illinois at Urbana-Champaign, Urbana, IL 61801 USA; Laboratory of Agrozoology, Department of Crop Protection, Faculty of Bioscience Engineering, Ghent University, Ghent, Belgium; Institute of Biology, Martin-Luther-University Halle-Wittenberg, Wittenberg, Germany; Department of Genetic Medicine and Development, University of Geneva Medical School, rue Michel-Servet 1, 1211 Geneva, Switzerland; Swiss Institute of Bioinformatics, rue Michel-Servet 1, 1211 Geneva, Switzerland; Computer Science and Artificial Intelligence Laboratory, Massachusetts Institute of Technology, 32 Vassar Street, Cambridge, MA 02139 USA; The Broad Institute of MIT and Harvard, 7 Cambridge Center, Cambridge, MA 02142 USA; Westfalian Wilhelms University, Institute of Evolution and Biodiversity, Huefferstrasse 1, 48149 Muenster, Germany; Centre for Genomic Regulation (CRG), Dr. Aiguader 88, 08003 Barcelona, Spain; Universitat Pompeu Fabra (UPF), Barcelona, Spain; Ernst Moritz Arndt University Greifswald, Institute for Mathematics and Computer Science, Walther-Rathenau-Str. 47, 17487 Greifswald, Germany; Genomics Division, Lawrence Berkeley National Laboratory, Berkeley, CA 94720 USA; National Center for Biotechnology Information, National Library of Medicine, Bethesda, USA; Department of Chemistry, Biotechnology and Food Science, Norwegian University of Food Science, N-1432 Aas, Norway; School of Computing Sciences, University of East Anglia, Norwich Research Park, Norwich, NR4 7TJ UK; Institute of Evolutionary Genetics, Heinrich Heine University Duesseldorf, Universitaetsstrasse 1, 40225 Duesseldorf, Germany; University of Cologne, Institute of Genetics, Cologne, Germany; Departamento de Biologia, Faculdade de Filosofia, Ciências e Letras de Ribeirão Preto, Universidade de São Paulo, 14040-901 Ribeirão Preto, Brazil; Institute of Evolutionary Biology and Edinburgh Genomics, The Ashworth Laboratories, The King’s Buildings, University of Edinburgh, Edinburgh, EH9 3FL UK; School of Biological Sciences, University of East Anglia, Norwich Research Park, Norwich, NR4 7TJ UK; School of Biological Sciences, Royal Holloway University of London, London, UK; Maynooth University Department of Biology, Maynooth University, Co, Kildare, Ireland; School of Biological Sciences, University of Bristol, 24 Tyndall Avenue, Bristol, BS8 1TQ UK; Land and Water Flagship CSIRO, Canberra, Australia; Department of Zoology, School of Natural Sciences, Trinity College Dublin, Dublin, Ireland; Institute of Zoology, Zoological Society of London, Regent’s Park, London, NW1 4RY UK; Theoretical Biology, Institute of Integrative Biology, Eidgenössiche Technische Hochschule (ETH) Zürich, CH-8092 Zürich, Switzerland; Swiss Institute of Bioinformatics, Lausanne, Switzerland; Computational Evolution, Department of Biosystems Science and Engineering, ETH Zürich, Basel, Switzerland; USDA-ARS Bee Research Laboratory, Maryland, USA; Departamento de Genética, Faculdade de Medicina de Ribeirão Preto, Universidade de São Paulo, 14040-900 Ribeirão Preto, Brazil; Center for Research in Scientific Computation, North Carolina State University Raleigh, Raleigh, NC USA; Laboratory of Insect Ecology, Graduate School of Agriculture, Kyoto University, Kyoto, Japan; Departamento de Biologia Celular e Molecular e Bioagentes Patogênicos, Faculdade de Medicina de Ribeirão Preto, Universidade de São Paulo, 14040-900 Ribeirão Preto, Brazil; Instituto Federal de Educação, Ciência e Tecnologia de São Paulo, 15991-502 Matão, Brazil; Department of Entomology, The Ohio State University, Wooster, OH 44791 USA; Department of Biological and Medical Sciences, Faculty of Health and Life Sciences, Oxford Brookes University, Oxford, OX3 0BP UK; Department of Biological Sciences, Xi’an Jiaotong-Liverpool University, Suzhou, China; Department of Laboratory Medicine, University Hospital Halle (Saale), Halle, Germany; German Centre for Integrative Biodiversity Research (iDiv) Halle-Jena-Leipzig, Leipzig, Germany; University of Southampton, Southampton, UK; Department of Biology, University of Leicester, Leicester, UK; Departamento de Genética e Evolução, Centro de Ciências Biológicas e da Saúde, Universidade Federal de São Carlos, 13565-905 São Carlos, Brazil; Departamento de Tecnologia, Faculdade de Ciências Agrárias e Veterinárias, Universidade Estadual Paulista, 14884-900 Jaboticabal, Brazil; Department of Biology, University of North Carolina at Greensboro, 321 McIver Street, Greensboro, NC 27403 USA; Institute of Biosciences & Applications, National Center for Scientific Research Demokritos, Athens, Greece; Ludwig Maximilian University, Munich, Germany; Human Genome Sequencing Center, Department of Molecular and Human Genetics, Baylor College of Medicine, MS BCM226, One Baylor Plaza, Houston, TX 77030 USA; Roy J. Carver Biotechnology Center, University of Illinois Urbana-Champaign, Urbana, IL USA; Department of Crop Sciences and Institute of Genomic Biology, University of Illinois at Urbana-Champaign, Urbana, IL 61801 USA; Molecular Genetic Technology Program, School of Health Professions, MD Anderson Cancer Center, 1515 Holcombe Blvd, Unit 2, Houston, TX 77025 USA; Department of Human Genetics, University of Chicago, Chicago, IL USA; Laboratory of Protein Biochemistry and Biomolecular Engineering, Department of Biochemistry and Microbiology, Ghent University, K.L. Ledeganckstraat 35, 9000 Ghent, Belgium; Department of Biological and Experimental Psychology, School of Biological and Chemical Sciences, Queen Mary University of London, Mile End Road, London, E1 4NS UK; School of Life Sciences, Guangzhou University, Guangzhou, China; Center for Computational Biology, McKusick-Nathans Institute of Genetic Medicine, Johns Hopkins University, Baltimore, MD 21205 USA; Carl R. Woese Institute for Genomic Biology, Department of Entomology, Neuroscience Program, University of Illinois at Urbana-Champaign, 1206 West Gregory Drive, Urbana, IL 61801 USA

## Abstract

**Background:**

The shift from solitary to social behavior is one of the major evolutionary transitions. Primitively eusocial bumblebees are uniquely placed to illuminate the evolution of highly eusocial insect societies. Bumblebees are also invaluable natural and agricultural pollinators, and there is widespread concern over recent population declines in some species. High-quality genomic data will inform key aspects of bumblebee biology, including susceptibility to implicated population viability threats.

**Results:**

We report the high quality draft genome sequences of *Bombus terrestris* and *Bombus impatiens*, two ecologically dominant bumblebees and widely utilized study species. Comparing these new genomes to those of the highly eusocial honeybee *Apis mellifera* and other Hymenoptera, we identify deeply conserved similarities, as well as novelties key to the biology of these organisms. Some honeybee genome features thought to underpin advanced eusociality are also present in bumblebees, indicating an earlier evolution in the bee lineage. Xenobiotic detoxification and immune genes are similarly depauperate in bumblebees and honeybees, and multiple categories of genes linked to social organization, including development and behavior, show high conservation. Key differences identified include a bias in bumblebee chemoreception towards gustation from olfaction, and striking differences in microRNAs, potentially responsible for gene regulation underlying social and other traits.

**Conclusions:**

These two bumblebee genomes provide a foundation for post-genomic research on these key pollinators and insect societies. Overall, gene repertoires suggest that the route to advanced eusociality in bees was mediated by many small changes in many genes and processes, and not by notable expansion or depauperation.

**Electronic supplementary material:**

The online version of this article (doi:10.1186/s13059-015-0623-3) contains supplementary material, which is available to authorized users.

## Background

Social living, and in particular eusociality (the social system in which many individuals forego reproduction), represents one of the major transitions in evolution [1], where a balance between cooperation and conflict must be met [2,3]. Eusociality has arisen multiple times [2,4]. Although the selective bases of the evolution of eusociality are relatively well understood [2,5,6], the evolutionary origins and dynamics of the molecular mechanisms underpinning eusociality remain obscure [7,8], making the understanding of the genomics of eusocial species a priority. Genome sequencing of social and eusocial species is expanding [9-17], but existing data do not span the spectrum of sociality or the phylogenetic diversity of social taxa.

In the Hymenoptera, the honeybees (tribe Apini), stingless bees (Meliponini), and certain ant species display advanced eusocial traits, including a permanent reproductive division of labor between queen and worker castes, worker females that show high degrees of task specialization, and, sometimes, caste polymorphism, and large perennial colonies with complex communication and organization [18,19]. In addition to these advanced eusocial species, the Hymenoptera include species with a spectrum of social traits [2]. The four tribes of corbiculate bees, Apini, Meliponini, Bombini, and Euglossini, are thought to have shared a primitively eusocial ancestor. Subsequently, the Meliponini and Apini evolved advanced eusociality independently, while the predominantly solitary behavior of the Euglossini was secondarily derived [18]. Although rare overall, advanced eusociality has arisen twice in this group, once following the split of honeybee and bumblebee lineages (approximately 77 to 95 million years ago (mya)), and once following the split of stingless bee and bumblebee lineages (approximately 66 to 82 mya) [18].

**Figure 1:**
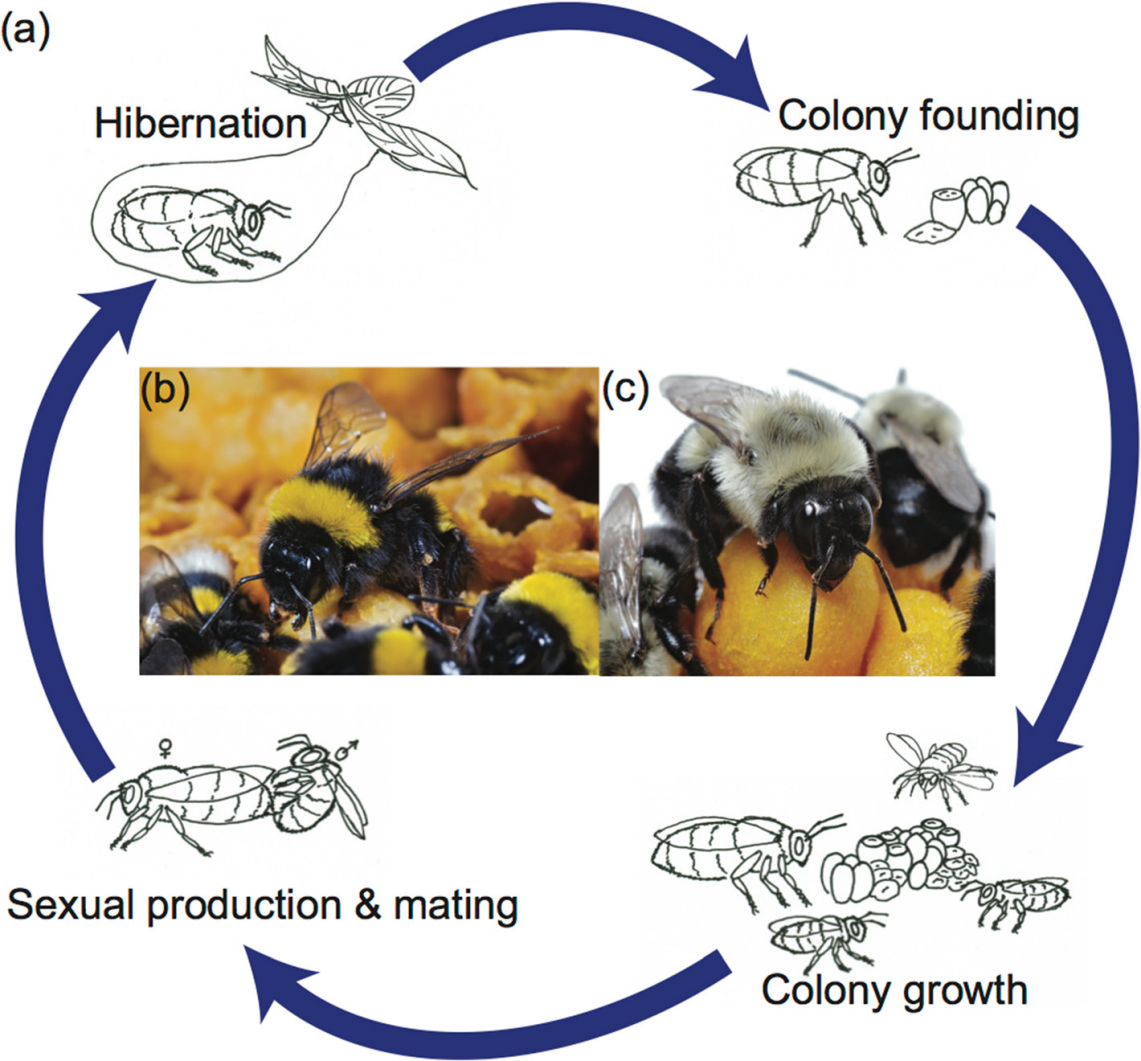
An illustrative colony cycle of bumblebee species living in temperate regions **(a)**. This is representative of the colony cycles of *Bombus terrestris* **(b)** and *B. impatiens* **(c)**. Queen bumblebees emerge from hibernation, establish a nest as a single foundress and provision it with pollen and nectar. Egg batches are laid that develop into female worker offspring. Once these offspring have developed and emerged as adults they take over foraging duties from the queen, and tend to developing brood. After sustained colony growth, males and new queens are produced. These sexuals leave the colony and mate. The new queens hibernate while males and the remainder of the colony perish.

The ‘primitively eusocial’ bumblebees (*Bombus spp.*, Bombini) share some traits with advanced eusocial bees, yet lack particular aspects that would qualify them as advanced eusocial organisms (Table 1). In comparison to honeybees, they have queen-worker caste differentiation based mainly on body size and physiology, annual colonies of hundreds rather than many thousands of individuals, and worker offspring that have lost the ability to mate, but can reproduce readily by laying haploid (male) eggs [20]. Bumblebees typically exhibit an annual colony cycle (Figure 1), although perennial colonies have been recorded in some bumblebee species such as the neotropical *B. atratus* [21], and social parasitic cuckoo bumblebees do not found their own colonies. There is a clear value to investigating bumblebees as they hold a key, intermediate position on the eusocial spectrum.

**Table 1:** **Key differences and similarities between honeybees,** ***Apis mellifera*** **, and the bumblebees** ***Bombus impatiens*** **and** ***B. terrestris***

	**Honeybee**	**Bumblebees**	
	***A. mellifera***	***B. impatiens***	***B. terrestris***
Native range	Africa/Asia/Europe	Temperate North America	Palaearctic region
Nesting	Cavity nesters		
Nest location	Trees	Ground	
Foraging	Generalist foragers of nectar and pollen		
Colony cycle	Perennial	Annual with queen diapause	
Colony founding	Colony fission	Solitary nest founding	
Sociality	Advanced eusocial	Primitively eusocial	
Colony size	Approximately 20,000-100,000 workers	<400 workers	
Queen mating system	Highly polyandrous	Limited polyandry	Monandrous
Worker division of labor	Age-based	Some size- and age-based	
Caste differentiation	Morphology/Size/Physiology	Size/Physiology	
Worker reproduction	Rare	Common	
Human links	Managed (hundreds-thousands of years)	Managed (decades)

Bumblebees are natively found around the globe, except for sub-Saharan Africa and Oceania, but reach their highest diversity in temperate, alpine, and arctic zones [20]. Two species of bumblebee, *B. terrestris* and *B. impatiens*, have in particular been the focus of research activity in a wide variety of fields. These include, among others, social evolution and organization [22-24], caste-structured development [25], learning [26], color vision [27], host-parasite interactions [28,29], plant-pollinator interactions [30], and community ecology [31,32]. The lineages leading to *B. terrestris* and *B. impatiens* separated approximately 18 mya [33,34]. *B. terrestris* is native to the Palaearctic and is common in many parts of Europe, North Africa, and parts of Western Asia, while the Nearctic *B. impatiens* is frequent in bumblebee communities of eastern North America. The species are placed in distinct sub-genera (*B. (Bombus) terrestris* and *B. (Pyrobombus) impatiens*) within the genus *Bombus* [33] and have some important biological differences (Table 1).

As a major component of the wild pollinator assemblage, bumblebees provide vital ecosystem services [35,36] and are also important for the productivity of agroecosystems [37]. Due to their effective pollination abilities, which are distinct from those of honeybees, bumblebees have also been employed in commercial pollination services [38]. *B. terrestris* and *B. impatiens* are both reared commercially and distributed internationally. The key role of bumblebees as pollinators is highlighted by losses in managed honeybee populations placing an increasing pollination burden on alternative pollinators [39]. However, many bumblebee species have also experienced marked population decreases recently [40,41]. Nonetheless, to date, *B. terrestris* and *B. impatiens* have proven to be relatively resilient to these declines. Relative abundances of *B. terrestris* in bumblebee communities in Europe have increased [42], while their absolute abundance has remained relatively stable [43]. *B. impatiens* has likewise increased in relative abundance in bumblebee communities in North America, since its populations have remained stable against a trend of declines in other bumblebee species [40,44]. In addition to remaining stable in its native range, *B. terrestris* has also proven to be an effective invader, further expanding its range as the result of human introductions [41,45,46]. Threats to both bumblebees and honeybees come from a variety of sources [47], and recently it has come to light that there is significant overlap of important pathogens between the two [48].

Genomic data form a rich platform on which comparative biology can be based. Comparative analyses of the genomes of honeybees and bumblebees will be crucial for understanding the relationships between these species, the dynamics of the evolution of eusociality, their resistances to pathogens, and their susceptibilities to other threats to pollinator health. Genomes of pollinators, such as bumblebees, will not only help understanding of the pollinator organisms themselves, but will also aid understanding of interactions between pollinators and plants [49]. For both *B. terrestris* and *B. impatiens* some genetic, genomic, and transcriptomic resources already exist [8,50-54].

Here we present high-quality draft genome sequences of two bumblebees, *B. terrestris* (Bter_1.0, accession AELG00000000.1) and *B. impatiens* (BIMP_2.0, accession AEQM00000000.2), and analyses that highlight both shared and divergent features compared to the honeybee (*A. mellifera*), other Hymenoptera, and further selected insects.

- We present high sequence coverage assembled genomes of *B. terrestris* (249 Mb) and *B. impatiens* (248 Mb).
- The two bumblebee genomes exhibit extensive synteny, with limited rearrangements over the estimated 18 My of divergence between the two lineages.
- We find relatively few repetitive elements and a low diversity of transposable elements, although there is some evidence of recent activity.
- Orthology and protein domain analysis uncovered bee- and bumblebee-specific genes and domains, with hints of evolutionary processes differentially acting upon aspects relating to chemosensation and muscle function in the bumblebee lineage.
- *B. terrestris* and *B. impatiens* are extremely similar in terms of gene content related to developmental pathways in molting, metamorphosis, and exoskeleton dynamics. This gene repertoire shows striking similarities among social and non-social Hymenoptera.
- A similar set of genes underlying haplo-diploid sex determination is present relative to honeybees, despite an alternative primary signal for sex determination being employed.
- Genes involved in behavior, neurophysiology, and endocrinology are broadly conserved between *A. mellifera* and bumblebees, yet limited differences do exist, and in particular among Juvenile Hormone Binding Proteins this may be connected to functional differences between these species.
- Xenobiotic detoxifying enzymes were found to be depauperate, as in *A. mellifera*, which has consequences for the ability of these species to deal with novel environmental xenobiotics, such as insecticides.
- Genes involved in chemoreception show expected complex patterns of gene birth and death. However, surprisingly, the gene repertoire of *B. terrestris* suggests that, relative to honeybees, bumblebees emphasize gustation over olfaction.
- Venom constituents, in general, are highly similar between honeybees and bumblebees.
- While components of all major immune pathways are present, as in *A. mellifera*, the complement of immune genes in the bumblebees is much reduced relative to Dipteran models, suggesting this is not a honeybee-specific characteristic, nor is it linked to advanced eusociality. Rather, it is likely that a reduced immune repertoire is basal to the bee lineage.
- RNAi core genes, RNA editing, and DNA methylation genes and genome wide patterns are highly conserved between *A. mellifera* and the two bumblebees.
- MicroRNAs (miRNAs) show a distinct pattern between the bumblebees and honeybees. Unique miRNAs were identified in both groups as well as potentially functionally relevant changes in conserved miRNAs. These are excellent candidates that may tune key biological differences between advanced eusocial honeybees and primitively eusocial bumblebees.

## Results and discussion

### Genome sequence and organization

We sequenced and *de novo* assembled the genomes of *B. terrestris* and *B. impatiens* from DNA derived from haploid males. *B. terrestris* sequence reads were assembled into a draft genome containing 236 Mb of sequence and spanning 249 Mb including estimated gaps (Table 2). Further genome information and statistics for Bter_1.0 can be found in Additional file 1. The *B. impatiens* genome was sequenced and *de novo* assembled to produce an assembly containing 243 Mb of sequence spanning 248 Mb including estimated gaps (Table 2). These genomes can also be accessed through BeeBase ([55], hymenopteragenome.org), which provides Genome Browser capabilities and BLAST searches against scaffolds and all gene predictions of both bumblebee genomes. Both genomes had high completeness as judged by presence of conserved gene sets (Additional file 1) and representation of independent transcriptome data.

**Table 2:** **Genome assembly statistics of** ***Bombus terrestris*** **(Bter_1.0) and** ***Bombus impatiens*** **(BIMP_2.0)**

**Genome assembly**	**Bter_1.0**	**BIMP_2.0**
Total sequence length	236 Mb	243 Mb
Total assembly length	249 Mb	247 Mb
Number of scaffolds	5,678	1,505
Scaffold N50	3.5 Mb	1.4 Mb
Number of contigs	10,672	12,033
Contig N50	76.0 Kb	57.1 Kb

Statistics are based on all scaffolds longer than 1,000 bp for BIMP_2.0.

### Synteny between the bumblebee genomes

Large-scale synteny is observed between the 18 chromosomes of *B. terrestris* and their corresponding *B. impatiens* scaffolds and contigs (details of coordinates in Additional file 2). Ninety-four *B. terrestris* scaffolds from the 18 chromosomes with the addition of 11 unplaced scaffolds (average length, 2.34 Mb; median, 1.34 Mb; range, 0.7 kb to 13.65 Mb; total length, 220.2 Mb) are covered by 101 synteny blocks formed from *B. impatiens* scaffolds and contigs (average synteny block length, 2.25 Mb; median, 1.16 Mb; range, 1.7 kb to 12.9 Mb) spanning 226.9 Mb. Thus, 89% of the investigated *B. terrestris* assembly is covered by synteny blocks representing 91% of the *B. impatiens* assembly. While synteny is high, it is likely an underestimate, being constrained by the fragmented genome assemblies of the two species. The existence of 14 large-sized synteny blocks (>5 Mb) corroborates this. Moreover, only eight and 10 cases were detected of intra- and inter- chromosomal rearrangements, respectively. The finding of a high degree of synteny between both bumblebee genomes is striking, as it is known that several social bees, including *B. terrestris*, have high genomic recombination rates [53,56]. This would lead to the expectation of higher frequencies of genomic rearrangements. However, these results concur with comparative linkage map based analyses suggesting a high conservation of genetic architecture within the Apidae [53].

### Repetitive elements in the bumblebee genomes

The *B. terrestris* and *B. impatiens* genomes were found to have 1,043 and 1,688 *de novo* predicted repetitive elements, respectively, of which 812 and 1,304 were validated by annotation of at least one complete copy. In total, 14.8% (36.2 Mb) of the *B. terrestris* assembly and 17.9% (44.6 Mb) of the *B. impatiens* assembly was found to be repetitive, with the diversity and abundance of transposable (interspersed) elements appearing similar across the two species (Additional file 1). Class I retroid elements and derivatives make up a large proportion of the genomes (8.5% in *B. terrestris*, 12.2% in *B. impatiens*). *Gypsy* is the most common long terminal repeat (LTR) retrotransposon covering 2.4 Mb in *B. terrestris* and 4.8 Mb in *B. impatiens*. Non-LTR retroid long interspersed elements (LINEs) have a similar cumulative length, with the majority being *Jockey*-like (2.6 Mb in both bumblebee species). Short interspersed elements (SINEs) are scarce. A major fraction of retroid elements (13.8 Mb in *B. terrestris* and 18.8 Mb in *B. impatiens*) were classified as large retrotransposon derivatives (LARD) or terminal repeat retrotransposons in miniature (TRIM). Class II DNA transposons were less frequent, with the majority being terminal inverted repeat (TIR) transposons, of which only *Mariner* and *PiggyBac* elements were common. Numerous repeat elements could not be assigned to a class (3.9 Mb in *B. terrestris* and 6.7 Mb in *B. impatiens*), and require further investigation.

The majority of the repeat elements appear shared between the two bumblebees. A large fraction of the *Gypsy* and *Mariner* elements were very similar to previously known transposable elements (two *Gypsy*, two *Mariner*) in *B. terrestris* (RepBase v17.01, [57]). The few *R2* clade elements show a more distant similarity to the single previously described *R2* element in *A. mellifera*. Other classified retroid elements show similarities to elements in other insect species, including the wasp *Nasonia vitripennis*, the ants, mosquitoes, and *Drosophila*. Interestingly, some of the bumblebee *PiggyBac* elements showed high similarities to other such elements from the beetle *Tribolium castaneum* or the moth *Bombyx mori*. Despite the lineage divergence time of 18 My, we found high degrees of sequence similarity between subsets of *Gypsy*, *Mariner*, and *PiggyBac* elements in *B. terrestris* and *B. impatiens*, suggesting a recent invasion by horizontal transfer into both species. A number of transposable elements are present in potentially active copies, with a high copy number indicating recent activity.

The two bumblebee genomes have an overall low number of transposable elements, together with a low diversity relative to other sequenced arthropods that typically have much higher percentages of repetitive DNA with higher diversity. For example, repetitive DNA in the genome comprises of 48.8% in the centipede *Strigama maritima* [58], 30% in the beetle *T. castaneum* [59], 38% in the aphid *Acyrthosiphon pisum* [60], over 30% in the wasp *N. vitripenis* [61], and typically greater than 20% in *Drosophila* [62] and ant species genomes [9,11,13,15,63]. The two bumblebee genomes contain only very small fractions or are almost devoid of *Copia*, *BelPao*, *Retrovirus*, and many of the LINE retroid elements, which are more common in other genomes [13,60,61]. Similarly, many superfamilies of DNA transposons are absent or only found in small quantities. For example, *hAT* and *P* elements, frequent in other insects [13,60,64], are scarce in these bumblebees. Some ant species have similar percentages of repetitive DNA in their genomes as the bumblebee genomes presented here [9,12], but only extremely specialized insects, the figwasp *Ceratosolen solmsi* (6.4%) [65] and an Antarctic midge *Belgica antarctica* (0.49%) [66], exhibit greatly reduced percentages in comparison. Within the Arthropoda, but outside of the Insecta, *Daphnia pulex* has a genomic repeat content of 9.4% [67]. A low percentage in *A. mellifera* (9.5%) also [16], however, suggests that the overall low number of transposable elements and low diversity in bumblebees is more deeply rooted in the Apinae.

### Predicted protein coding genes in the bumblebee genomes

Protein-coding genes were predicted from the Bter_1.0 and BIMP_2.0 assemblies using a diversity of *de novo* pipelines (NCBI RefSeq and Gnomon pipelines, AUGUSTUS, SGP2, GeneID, Fgenesh++ and N-SCAN; see Additional file 1). A merged gene set for each species was produced using GLEAN [68]. Targeted manual annotation was used to validate 657 gene models from *B. terrestris* and 346 gene models from *B. impatiens*. Approximately one-quarter of the automated models (24%) were edited, usually only to correct start and stop codon usage and intron-exon boundaries using transcriptome and comparative evidence (see Additional file 2 for details of these genes, along with gene information of species-specific gene names reported in some subsequent sections (for example, Bter_ or Bimp_)). Analyses focusing on specific gene families and pathways identified additional issues with some automated predictions (see below, for example, chemoreceptors).

### Analysis of orthology relationships of bumblebee genes

**Figure 2:**
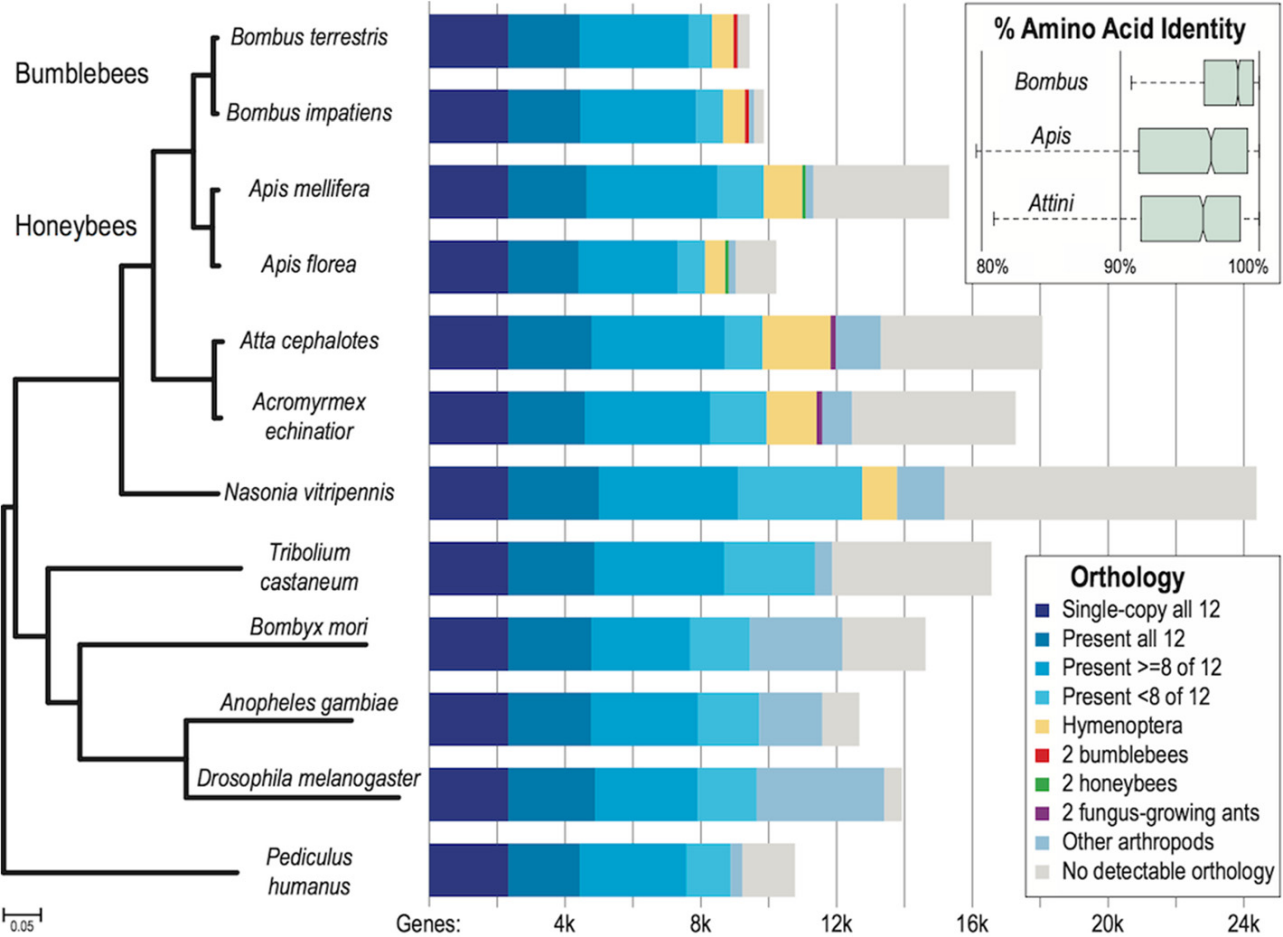
Bumblebee orthology with selected Hymenopterans and representative species from other insect orders. The maximum likelihood phylogenetic tree built from the concatenated alignments of 2,294 single-copy orthologs recovers the expected phylogeny rooted with the human body louse, *Pediculus humanus*. The tree highlights the pairs of closely-related bumblebees (*Bombus terrestris* and *Bombus impatiens*), honeybees (*Apis mellifera* and *Apis florea*), and fungus-growing ants (*Atta cephalotes* and *Acromyrmex echinatior*). It also shows slow average rates of molecular evolution in the Hymenopterans, similar to the flour beetle, *Tribolium castaneum*, but much slower than the silk moth, *Bombyx mori*, the malaria mosquito, *Anopheles gambiae*, and the fruit fly, *Drosophila melanogaster*. The bars represent the total gene counts in each species partitioned according to their homology to genes in the other species and other arthropods: from universally present single-copy orthologs (dark blue, left) to lineage-specific orthologs, and genes with no detectable orthology (gray, right). A small fraction made up of about 100 to 150 genes in each of the bee and ant species exhibit orthology only to genes from the most closely-related species (red, green, purple). The inset boxplots show the distributions of percent amino acid identities between pairs of *Bombus*, *Apis*, and *Attini* (ants) universal single-copy orthologs, where the identity is much higher between the bumblebee orthologs than between the honeybees or the ants.

Ortholog analysis was carried out with OrthoDB6 [69,70] based on the Refseq gene sets of *B. impatiens* and *B. terrestris*. As expected, the vast majority of genes have orthologous relationships across the Hymenoptera (Figure 2). Ortholog sets that are only found in particular lineages are likely to play important roles in lineage-specific biological traits, and thus deserve further attention. The addition of the bumblebee genomes allowed for the identification of 38 orthologs specific to bees (*B. terrestris*, *B. impatiens*, *A. florea*, *A. mellifera*) (Additional file 2). Of greater interest for bumblebee biology are the 118 orthologs that, at this time, are found solely in the bumblebee lineage (Additional file 2). These bumblebee-specific ortholog groups were in general poorly annotated with InterPro domains [71], but 13 of the 24 orthologs in which domains could be found contained an olfactory receptor domain (IPR004117). Further, one bumblebee lineage specific gene ortholog (OrthoDB group: EOG6VDNJ0) has likely duplicated in *B. terrestris*. Genes containing this domain are seven-transmembrane proteins and are candidate odorant receptors in other species. These preliminary insights suggest alterations in chemosensation in the bumblebees, further explored below. However, considerable work will be required to discover how these uncovered bumblebee-specific genes relate to bumblebee biology.

### Patterns of protein domain evolution in *B. terrestris*

The evolutionary dynamics of protein domains are often distinct from dynamics on the gene level, and sometimes signals of adaptation only become apparent at the protein domain level. Protein domains of *B. terrestris* were compared to several reference species (*A. mellifera*, *Atta cephalotes*, *Culex cinquefasciatus*, *Drosophila melanogaster*, *Daphnia pulex*, *Harpegnathos saltator*, *Pediculus humanus*, *T. castaneum*) (Additional file 1). Domains found uniquely in one species compared to closely related species can indicate horizontal gene transfer. One *B. terrestris* domain not found in the other arthropods was an RNA-dependent RNA-polymerase (Pfam domain PF00978)*.* This domain is found on predicted gene au6.g7950 between 97 and 133 kb of scaffold CM001185.1. The predicted gene has 17 exons, three of which are annotated with Pfam domains, with PF00978 located in the eighth exon. This domain is normally part of RNA viruses, and its presence in the *B. terrestris* genome may be a nuclear insertion of genetic material from a viral infection, but no longer encode a functional RNA-dependent RNA-polymerase.

Expansions and contractions of domains relate to lineage-specific changes in domain copy numbers. These can be linked to gene duplication or loss, but can also be independent of this process. When comparing *B. terrestris* domains to all reference species, only two domains were significantly different in their occurrences (*P* <0.05, Fisher’s exact test). Both of these domains (PF07530 (*P* <0.001) and PF07727 (*P* = 0.001)) appear related to transposable elements [72,73]. In comparison to *A. mellifera* alone, 16 expansions or contractions were found in *B. terrestris*, with most being related to transposable elements (Additional file 2) or the zinc finger receptor family. However, three of these domain changes are of further interest. A major royal jelly protein (PF03022 (*P* = 0.007)), involved in honeybee larval nutrition and caste differentiation [74] is contracted in *B. terrestris*, with six copies in comparison to the 22 in *A. mellifera*. PF07993 (*P* = 0.01), involved in age-related decreases of transcript levels in *D. melanogaster* [75], is expanded to six copies in *B. terrestris*, but is not present in *A. mellifera*. Three seven-transmembrane receptors involved in chemoreception (PF02949 (*P* <0.001), PF00001 (*P* = 0.016), PF08395 (*P* = 0.016)) are contracted in *B. terrestris* based on analyses of these domains, and are found at 12%, 53%, and 0%, respectively, of their abundances in *A. mellifera*. A comparison of the bees (*B. terrestris* and *A. mellifera*) to the ants (*A. cephalotes* and *H. saltator*) revealed seven significant domain expansions or contractions, with four being related to transposable elements. The others were, in bees relative to ants, a contracted trypsin domain (PF00089), an expanded trypsin inhibitor domain (PF01826), and an expanded zinc finger domain (PF13912).

Domain repeat numbers within a protein often change rapidly, with multiplication of a domain within a protein potentially indicating a strong signal of selection. Repeat numbers of domains within proteins were compared between *B. terrestris* and *A. mellifera*. Nine domains were found to differ significantly in their repeat numbers. Several of these domains have functions related to muscle, and hint at potential selection pressure on the muscle apparatus of bumblebees. These adaptations could be related to distinct muscle features of bumblebees, for example, in warming up the flight muscles [20].

### Development related genes in the bumblebee genomes

Examination of both bumblebee genomes for developmental genes indicates that the developmental gene complement of *A. mellifera*, with its loss of specific developmental genes, is shared with bumblebees, strengthening the finding that these are indeed missing from the *A. mellifera* genome and indicating that early development may have evolved differently in the corbiculate bees as a whole.

The complement of genes involved in segmentation and dorsoventral patterning are identical to those of *A. mellifera*. Both bumblebees have all components found in the *A. mellifera* for *Notch*, *Wingless*, *hedgehog*, and *TGF-beta* signaling. They also have similar *runt* [76], *E(spl)* [77], and *Hox* complexes [78] to *A. mellifera*. An interesting finding from the *A. mellifera* genome sequence was that a set of genes that act in early patterning of *Drosophila* embryos were absent [78]. This set of genes (*torso*, *trunk*, *gurken*, *bicoid*, *swallow*, and *oskar*) is also completely lacking in the bumblebee genomes. While some of these genes arose in the dipteran lineage (for example, *bicoid* and *swallow*) [78-80], others are found in the genomes of hemimetabolous insects such as the pea aphid (*torso*) [81], or in other Hymenoptera (for example, *torso*, *oskar*) [82]. This indicates within-Hymenoptera lineage-specific loss of at least the *torso* and *oskar* genes.

Hemocyanin derived genes include hexamerins (*hex*), involved in metamorphic molting [83], and prophenoloxidases (*PPO*), associated with melanisation and exoskeleton pigmentation [84]. As in *A. mellifera*, four *hex* genes, with the genomic clustering of three of these genes also being conserved, and a single copy of *PPO*, with transcriptomic evidence for alternative splicing, were found. Other genes with a single copy and likely one-to-one orthologous relationships include many involved in post-embryonic development, including *ftz-f1*, Broad-complex, calponin (*Chd64*), eclosion homone (*EH*), ecdysis triggering hormone (*ETH*), bursicon α and β, cuticular peroxidase, dopa-decarboxylase (*DDC*), chitinase, and tyrosine hydroxylase (*TH*). Predicted gene models and/or transcriptional evidence indicate alternative isoforms for most of these genes in bumblebees. *Tweedle*, *apidermins*, and other cuticular proteins (such as *CPLCP* and *CPF*) have the same numbers as *A. mellifera* [85]. Multicopper oxidases (*MCOs*), including laccases, have roles in development and a wide variety of other biological processes [86]. Seven *MCO* genes are found in both bumblebee genomes, while there are only five in *A. mellifera* and 11 in *Nasonia spp*. These genes show clear orthology, but also species-specific expansion (for example, *Nasonia spp.*) and loss (for example, *A. mellifera*). CPR cuticular proteins are encoded by 37 to 58 genes across sequenced Hymenopterans, which is considerably fewer than are found in other insects (86 to 241). Other genes involved in development, including members of the basic Helix-Loop-Helix/Per-Arnt-Sim (*bHLH-PAS*) transcriptional factors, show deep conservation within the insects.

Genes encoding proteins that are important for development and differentiation of the central nervous system and the brain, cell polarity, axon guidance, Malphigian tubule morphogenesis, eye development, and pathways like the Notch signaling pathway are conserved in the bumblebees. The genes *achaete*, *scute*, *lethal of scute*, and *asense* are part of the *achaete-scute* complex of *D. melanogaster* [87] and encode transcriptional activators of the bHLH class. They are important proneural genes that instruct clusters of cells to become competent to form neuroblasts [88] and play a critical role in the formation of the central nervous system of the embryo and the peripheral nervous system (sensory bristles) of adults [87]. Of the four genes of the *achaete-scute* complex, only one copy is present in *B. terrestris* while two copies are found in *A. mellifera* [89]. This finding suggests that a single gene of the *achaete-scute* gene family is sufficient in *B. terrestris* to form the neuroblasts and the CNS. Another gene involved in the development of the CNS, *escargot* [90] is not present in *B. terrestris.* In *D. melanogaster, escargot* acts with redundant function with other members of the snail protein family (*snail* and *worniu*) to control embryonic central nervous system development [90]. The homeobox containing genes *ladybird late* and *ladybird early* encode transcription regulators, which play an important role in neurogenesis, myogenesis, and cardiogenesis [91], and are also missing in *B. terrestris*.

### Haplo-diploid sex determination in the bumblebee genomes

Hymenoptera species do not possess sex-specific chromosomes, but instead employ a haplodiploid mode of sex determination, which typically leads to males arising from unfertilized eggs (haploid) and females from fertilized eggs (diploid). Single-locus sex determination has been extensively studied in *A. mellifera*, where the initial signal has been identified to be the multiallelic gene *complementary sex determiner* (*csd*) [92]. Bumblebees share orthologs for numerous genes known to be involved in *Drosophila* and *Apis* sex determination such as *doublesex* (*dsx*), *transformer 2* (*tra 2*), *fruitless* (*fru*), and *transformer* (*tra*)/*feminizer* (*fem*). The single sex determination locus described for *A. mellifera* harbors the tandemly arrayed paralogous genes *csd* and *fem* [93], whereas in *B. impatiens* and *B. terrestris* the paralog of *fem*, *feminizer 1 (fem1)*, is located on different chromosomes. In contrast to the allelic variability of *Apis csd*, *fem1* lacks allelic variability based on sequences from natural *B. terrestris* populations, suggesting that *fem1* is unlikely to be an allelic factor acting as a primary signal in the sex determination pathway (Hasselmann *et al.*, unpublished). In other Hymenopteran genomes than *Apis* and *Bombus*, paralogous copies of *fem* (*tra*) have been found in ants and the Halictid bee *Lasioglossum albipes*, suggesting this gene duplication as a potentially ancestral event in the early evolutionary history of Hymenopteran species [15,94,95]. However, analysis provides evidence that *csd* and *fem1* are not orthologs, and originated independently by gene duplication from the *fem* gene in *A. mellifera*, the bumblebees, and ants [96,97]. Bumblebee *dsx* and *fem* are sex-specifically spliced, consistent with the evolutionary conservation of the pathway at this level. Alternative splice variants are also found for *fem1*, which is not the case for *csd* in *A. mellifera* (Hasselmann *et al.*, unpublished)*.* For three genes (*sisterless A*, *outstreched*, *suppressor of variegation 3-7*) no potential orthologs were identified in the bumblebee or any other Hymenopteran genome. All genes involved in dosage compensation in *D. melanogaster* have orthologs in the bumblebee genomes, despite the lack of sex chromosomes. These genes might have additional, for example chromatin-related, functions in the bumblebee. It is also tempting to speculate about an association to haplodiploidy given the complexity by which those genes orchestrate with the transcription regulating machinery [98]. Consequently, for example, these orthologs could fine-tune the transcription of maternally and paternally provided genetic material in fertilized eggs, compensating allele-specific differences.

### Behavior, neurophysiology, and endocrinology related genes in the bumblebee genomes

Advanced eusociality requires extensive behavioral coordination, and castes typically differ in their behavioral phenotypes, spatially or temporally. In the primitively eusocial bumblebees, while behavioral differentiation is present, it is not as distinct as in the advanced eusocial honeybees, making the landscape of genes involved in behavior and the neuronal and physiological processes underlying behavioral phenotypes an interesting avenue of investigation.

Innate circadian clocks govern the daily timing of many organismal processes, from gene expression to behavior. The set of clock genes in bumblebees is highly similar to those of *A. mellifera* [99]. Only a timeout (*Tim2*), but no timeless (*Tim1*), and a mammalian-like cryptochrome (*Cry-m*), but not a *Drosophila*-like cryptochrome (*Cry-d*) were found in the bumblebee genomes. Thus, the core circadian feedback loop is mammalian-like rather than *Drosophila-*like, a finding also recently described in ants [100].

The Takeout/juvenile hormone binding proteins (*To/JHBP*), present only in insects, share a defining domain thought to bind small lipophilic molecules such as juvenile hormone (JH) [101], yet the exact ligands are mostly unknown. In *Drosophila*, *To* is linked to circadian rhythms, with *To* mutants showing abnormal locomotor activity rhythms and rapid death on starvation [102,103]. In *A. mellifera*, eight genes of the *To/JHBP* family have been identified [104]. The genomes of the two bumblebees each contain 11 putative *To/JHBP* family genes (Additional file 1). Interestingly, two of these genes have no true orthologs in *A. mellifera* (GB13060_1, and GB17010), but orthologs are present in *Nasonia*, suggesting the loss of these two genes since the separation of the honeybee and bumblebee lineages (Additional file 1). It would be interesting to test if these differences in *To/JHBP* complements are related to different physiologies of honeybees and bumblebees, such as the apparent differences in JH signaling. In bumblebees JH regulates fertility and female reproductive physiology [105], whereas in adult honeybees JH influences worker division of labor but not fertility and reproduction [106]. All protein models of the identified genes contain significant JH-binding domains, and signal peptides were identified by at least one of the two methods used in all except Bter_GB17010 and Bimp_GB17010 (Additional file 1). All putative *To/JHBPs* are co-localized on *B. terrestris* LG B09, apart from Bter_GB19811, which is located on LG B08. The location of the *JHBPs* in the genome of *B. terrestris* is comparable to that in *A. mellifera*, suggesting high synteny for this group of genes, which is consistent with the premise that the *To/JHBP* family was created by ancient duplication events.

The genes of the cys-loop ligand-gated ion channel (cysLGIC) superfamily mediate synaptic transmission in insects. The genomes of *B. terrestris* and *B. impatiens* both contain the same complement of 21 cysLGIC genes, with 11 of these genes encoding putative nicotinic acetylcholine receptor subunits (nAChRs), while the remainder of the bumblebee *cysLGIC* superfamily include genes for ion channels gated by gamma-aminobutyric acid (GABA receptors), glutamate (GluCls), and histamine. This complement of 21 cysLGIC genes is the same as found in *A. mellifera* [107]. Studies in honeybees have shown that nAChRs, GABA receptors, and GluCls play key roles in behavior, such as olfactory learning and memory [108,109]. Insect cysLGICs are also of importance as they are targets of widely used insecticides, examples of which are fipronil (which acts on GABA receptors and GluCls) and neonicotinoids (which act on nAChRs) [110]. The bumblebee nAChRs may mediate sublethal effects of neonicotinoid pesticides on foraging behavior and colony traits [111-113]. CysLGIC sequence information from diverse species, including key pollinating insects, is a valuable starting point for understanding the interaction of insecticides with their targets, and may prove instructive in the future design and development of improved insecticides with enhanced specificity and reduced effects on non-target beneficial species.

Biogenic amines, neuropeptides, protein hormones, and their G-protein-coupled receptors (GPCRs) play a central role in the physiology of insects and control many important processes, including behavior, development, feeding, and reproduction [114]. Insects have 16 to 22 biogenic amine GPCRs for identified insect biogenic amines (acetylcholine, adenosine, dopamine, octopamine, tyramine, and serotonin) [115,116]. Both bumblebees have a similar set of 20 biogenic amine GPCRs (Additional file 1). Compared to other sequenced insects, one octopamine receptor was found to be duplicated in the two bumblebees, as was also found in *A. mellifera*. The two bumblebees have a similar set of 34 neuropeptide preprohormone genes coding for approximately 65 different neuropeptides (Additional file 1). However, functionality of *corazonin*, thought to be involved in copulation behavior [117], carbohydrate and lipid mobilization [118], and stress [119], is likely different in *B. impatiens*, as the preprohormone cannot be cleaved to a functional *corazonin* (Additional file 1). Twelve neuropeptide genes found in other arthropods are absent in both bumblebee species, with their GPCRs also absent where they are known. Interestingly, the two bumblebee species have a similar, but still unique, neuropeptide suite compared to *A. mellifera*, with *sulfakinin* found only in *A. mellifera* and *trissin* found only in bumblebees (Additional file 1). This unique suite of neuropeptides is likely to underlie bumblebee*-*specific physiology and behavior.

TRP (Transient Receptor Potential) channels are activated by diverse stimuli and function as the primary integrators of sensory information such as vision, thermosensation, olfaction, hearing, and mechanosensation. The TRP superfamily is divided into seven subfamilies (TRPA, TRPC, TRPM, TRPML, TRPN, TRPP, and TRPV) [120]. The bumblebee genomes contain the same set of TRP channel genes (5 TRPA, 3 TRPC, 1 TRPM, 1 TRPML, 1 TRPN, and 2 TRPV subfamily members) as *A. mellifera* and *N. vitripennis*. Both bumblebee species lack *TRPA1*, but maintain *TRPA5* (a TRPA subfamily member lost in Diptera) and *HsTRPA*. Three other conserved TRPA channels, *Painless, Pyrexia*, and *Wtrw*, are present. Thus, the pattern of TRPA subfamily members is conserved between these two bumblebees, *A. mellifera*, and *N. vitripennis*.

### Xenobiotic detoxifying enzymes and related genes in the bumblebee genomes

Overall bumblebees, similar to honeybees, have a reduced set of detoxification enzymes. Being mutualistic pollinators, bumblebees are not faced with a plethora of toxic plant secondary metabolites that require detoxification, as herbivores are. This, and the potential of low incidence of xenobiotics due to their social lifestyle, could account for why these xenobiotic detoxifying enzymes are so impoverished in these species. However, xenobiotics are now encountered in the form of systemic insecticides, and investigations of how a general lack of detoxification related genes, along with species-specific alterations, influences susceptibility is vital to understand one of the major purported threats to pollinator health.

Glutathione-S-transferases (GSTs), carboxyl/cholinesterases (CCEs), and cytochrome P450 monooxygenases (p450s) are involved in the detoxification of xenobiotics, along with playing roles in key physiological pathways. These gene families were found to be extremely depauperate in the *A. mellifera* genome [121].

GST and CCE numbers are comparable in the two bumblebees to those numbers found in *A. mellifera* (Table 3). Thus, the reduction in numbers in *A. mellifera* [121] is not unique, but rather taxonomically more widespread. Despite a similarity in overall numbers, there are key changes in CCEs between the bumblebees and honeybees on the level of clades and their classes. In bumblebees, relative to *A. mellifera*, dietary/detoxification associated CCE genes are reduced in number, while hormone and semiochemical processing associated CCE genes are increased.

**Table 3:** **Detoxification enzymes and related genes**

**Gene family**	**Clade/Class/Clan**	**B. imp.**	**B. ter.**	**A. mel.**	**A. flo.**	**P. bar.**	**N. vit.**	**T. cas.**	**D. mel.**
GSTs	Delta	5	5	4	4	-	4	-	-
	Epsilon	0	0	0	0	-	0	-	-
	Omega	2	2	2	2	-	2	-	-
	Sigma	4	4	4	4	-	8	-	-
	Theta	1	1	1	1	-	3	-	-
	Zeta	1	1	1	1	-	1	-	-
	Total GSTs	13	13	12	12	-	18	-	-
CCEs	A (DD)	3	3	5	5	-	10	-	-
	B (DD)	2	2	3	3	-	6	-	-
	C (DD)	0	0	0	0	-	0	-	-
	D (HSP)	3	3	1	2	-	5	-	-
	E (HSP)	3	3	2	2	-	12	-	-
	F (HSP)	2	2	2	2	-	2	-	-
	G (HSP)	0	0	0	0	-	0	-	-
	H (NDCA)	1	1	1	1	-	1	-	-
	I (NDCA)	1	1	1	1	-	1	-	-
	J (NDCA)	2	2	2	2	-	2	-	-
	K (NDCA)	1	1	1	1	-	1	-	-
	L (NDCA)	5	5	5	5	-	5	-	-
	M (NDCA)	1	1	1	1	-	1	-	-
	Total CCEs	24	24	24	25	-	46	-	-
P450s	CYP3	27	27	28	-	40	49	65	36
	CYP4	4	4	4	-	18	29	41	32
	CYP2	7	7	8	-	7	7	8	6
	Mitochondrial	6	6	6	-	7	7	9	11
	Total P450s	44	44	46	-	72	92	123	85

Gene counts of glutathione-S-transferases (GSTs), esterases (CCEs), and P450s among selected holometabolous insect genomes.

A. flo = *Apis florea*, A. mel = *Apis mellifera*, B. imp = *Bombus impatiens*, B. ter = *Bombus terrestris*, D. mel = *Drosophila melanogaster*, DD = Dietary and detoxification, HSP = hormone and semiocheical processing, N. vit = *Nasonia vitripennis*, NDCA = Neuro-developmental and cell adhesion, P. bar = *Pogonomyrmex barbatus*, T. cas = *Tribolium castaneum*.

- = species not included in particular analysis.

The bumblebee genomes contain 44 putatively functional cytochrome P450 monooxygenase genes (P450s) and seven pseudogenes, very similar to the complement of 46 P450s encoded in the genome of *A. mellifera*, but considerably smaller than the number of P450s in the genomes of most other holometabolous insects (Table 3) [12,59,121,122]. The bumblebee genomes include all other expected orthologous P450s in the CYP2 and mitochondrial clans, which are involved in ecdysteroid hormone synthesis and breakdown [123]. The insect steroid hormone, 20-hydroxyecdysone (20E), controls and coordinates insect development through the ecdysteroid-signaling cascade. Enzymes responsible for 20E synthesis are a group of cytochrome P450s (Additional file 1). To date, four P450 enzymes, namely CYP306A1 (*Phantom, Phm*), CYP302A1 (*Disembodied, Dib*), CYP315A1 (*Shadow, Sad*) and CYP314A1 (*Shade, Shd*), involved in ecdysteroid biosynthesis have been identified and characterized. Additionally, a group of paralogous CYPs (CYP307A1 (*Spook, Spo*), CYP307A2 (*Spookier, Spok*), the paralog gene of *Spo*, and CYP307B1 (*Spookiest, Spot*)) are identified. They are all involved in the initial conversion process from 7-dehydrochoresterol into ketodiol, but their biochemical functions are not well understood [124]. They are called the Halloween genes. All Halloween genes present in *A. mellifera* are found in *B. terrestris* and *B. impatiens* (Additional file 1). Similar to *A. mellifera*, no ortholog for *Spo*, which is present in multiple other hemimetabolous and holometabolous insects, was found. Phylogenetic analysis demonstrated the identity of the *Spot* paralog and also confirmed the identity of the other Halloween genes (Additional file 1).

Honeybees and bumblebees are uniquely depauperate in the CYP4 P450s, as each bee genome encodes just four well-conserved orthologs in this clan, while other insect genomes contain a great diversity of genes in this group. Lack of CYP4 P450 diversity in the bees is somewhat surprising because this group has been associated with pheromone synthesis and breakdown [125]. Two of the four CYP4 P450s shared by bees, the pair of CYP4G orthologs, are known to be involved in the synthesis of cuticular hydrocarbons in other insects [126] and may be involved in the production of secreted wax in bumblebees and honeybees.

The CYP3 group members are the only P450s in the sequenced bees that do not display clear 1:1 orthology with other insects. This clan shows evidence of recent gene duplication and divergence in species specific ‘blooms’ [123]. Members of the CYP3 clan detoxify pesticides and natural xenobiotics in honeybees and other insects [127,128]. While the *A. mellifera* and two bumblebee genomes appear to encode similar numbers of CYP3 P450s, this gene count masks gene birth and death events occurring in each genome. *Apis mellifera* has three CYP9Q P450s, which metabolize synthetic insecticides [127]. While *B. impatiens* also has three CYP9Qs, *B. terrestris* has a single putatively functional CYP9Q P450.

### Chemoreceptors in the bumblebee genome

Chemosensation plays a major role in social interactions in insect societies, and is critical to the ecological interactions of bees. The odorant receptor (OR) family of seven-transmembrane proteins in insects mediates most of insect olfaction [129], with additional contributions from a subset of the distantly related gustatory receptor (GR) family, for example, the carbon dioxide receptors in flies [130], and a subset of the unrelated ionotropic receptors (IRs) [131].

#### Odorant receptors (ORs)

The *A. mellifera* genome revealed an expansion of the OR family relative to previously sequenced fly genomes [132], with a total of 177 genes (updated in [11,12]). This expansion has been even greater in other sequenced Hymenoptera, including *Nasonia* wasps with around 300 OR genes [133] and several ant species with around 400 OR genes [11,12,15,134]. Analysis of *Bombus* ORs indicates that they have a slightly less diverse OR family than *A. mellifera*, with 164 genes (Additional file 1). There are just five pseudogenes (3%), which is even lower than the 5% in *A. mellifera*. The result is 159 apparently intact OR proteins, although there is a small subfamily (BtOr128-139, related to AmOr97-105) with an additional short coding exon for the start codon, which usually could not be confidently identified, so their functionality remains uncertain. As expected, there is a single conserved ortholog of the *DmOr83b* protein, now called *Orco* [135], sharing 92% amino acid identity with *AmOrco* (*Or2*) and 63% with *DmOrco* (*Or83b*). There are no other orthologous relationships of bee ORs to the *Drosophila* ORs [132]. Comparing *B. terrestris* and *A. mellifera* ORs there is a combination of single orthologs for many genes, duplications of genes in one or both species, several large species-specific gene lineage expansions, and at least 22 gene losses, reflecting the birth-and-death gene family evolution typical of these receptors (Additional file 1). The largest known tandem duplication of insect chemoreceptors is AmOr1-61 with equivalents in *Bombus* of BmOr1-46, and this large subfamily contains the only Hymenopteran OR for which a ligand is known, AmOr11, which perceives the major bee queen pheromone component 9-ODA [136].

Another large subfamily is the 9-exon gene subfamily, totaling 49 genes (BtOr116-164 and AmOr97-113, 122–139, 140, 159, 172–177), which all share the same gene structure, except that AmOr97-113 and BtOr128-139 have an additional very short 5’ exon containing the start codon. The major expansion of this subfamily in ants was suggested to indicate that it comprises the cuticular hydrocarbon receptors involved in nestmate and kin recognition [11,12,134]. This subfamily has the largest species-specific expansions in both *B. terrestris* and *A. mellifera* (Additional file 1). Based on branch lengths, these also appear to be among the most rapidly evolving ORs. Meanwhile, the oldest lineages in this subfamily, AmOr 159, 176, and 177 and BtOr156, 157, and 159, each appear to have been lost from the other species (indeed a non-functional fragment of an AmOr177 ortholog remains in the bumblebee genome), suggesting that their functions are being outlived. This 9-exon subfamily also contains the first clear case of trans-splicing observed in the insect chemoreceptor family, something that is becoming better known in other insect genes [137]. The lineage of BtOr161-163 and AmOr140 has the first coding exon in the appropriate location upstream of the remaining exons, but in reverse orientation. While this unusual arrangement was recognized for AmOr140, it was discounted and that gene was previously treated as having an unrecognized N-terminus (AmOr140NTE) [132]. Discovery of the same arrangement for this exon in the related bumblebee genes makes it clear that these are trans-spliced genes.

#### Gustatory receptors (GRs)

The GR family of seven-transmembrane proteins in insects mediates most of insect gustation [129], as well as some aspects of olfaction. In contrast to the OR family, the GR repertoire was considerably reduced in *A. mellifera* compared with flies, at just 12 genes [11,12,132], which is far fewer than the number found in the other available Hymenoptera [11,12,133,134]. Compared to *A. mellifera*, somewhat surprisingly, *B. terrestris* has 25 GR genes (Additional file 1). Of these, 23 are apparently intact proteins. The phylogenetic tree (Additional file 1) reveals the relationships of *B. terrestris* and *A. mellifera* GRs in relation to those of *D. melanogaster*. As is the case for *A. mellifera* [132] and other Hymenoptera examined to date [138], there are no *B. terrestris* orthologs for the carbon dioxide receptors (DmGr21a and 63a). *B. terrestris* has conserved orthologs for the two candidate sugar receptors in *A. mellifera*, BtGr1/2 [139]. The BtGr3/AmGr3 lineage is the ortholog of the DmGr43a receptor that has recently been shown to be a fructose receptor that also functions as a brain nutrient receptor [140]. The AmGr4/5 lineage appears to be an *Apis*-specific duplication, because there is only one gene in *B. terrestris*, and it is now a pseudogene (BtGr4PSE). Remaining *B. terrestris* and *A. mellifera* GRs have no convincing relationships with *D. melanogaster* GRs to allow for functional inference, but show expected patterns of birth-and-death typical of the chemoreceptor and other environmentally-relevant gene families. While Gr6 and Gr7 are simple orthologs, the others provide interesting comparisons. BtGr5 is an intact relative of the large set of highly degraded pseudogenes in the *A. mellifera* genome, represented here by the AmGrX-Z constructs. This lineage apparently both expanded and completely pseudogenized within the *Apis* and related bee lineages. Gr10 and Gr11 are pseudogenes in *B. terrestris* and *Apis*, respectively, so their respective functions have been lost. The AmGr8/9 pair of duplicated genes have experienced a repeated set of duplications as a pair in *B. terrestris*, yielding five genes each (BtGr8/9, and 14–21), although BtGr17 is a pseudogene. The newly recognized AmGr12 is also repeatedly duplicated in *B. terrestris*, again yielding five genes, all still intact (BtGr12 and 22-25). Finally, a truncated version of a highly divergent GR was recognized in each genome, called Gr13. Gr13 is missing the usually conserved C-terminus, but is otherwise a seemingly intact gene.

**Figure 3:**
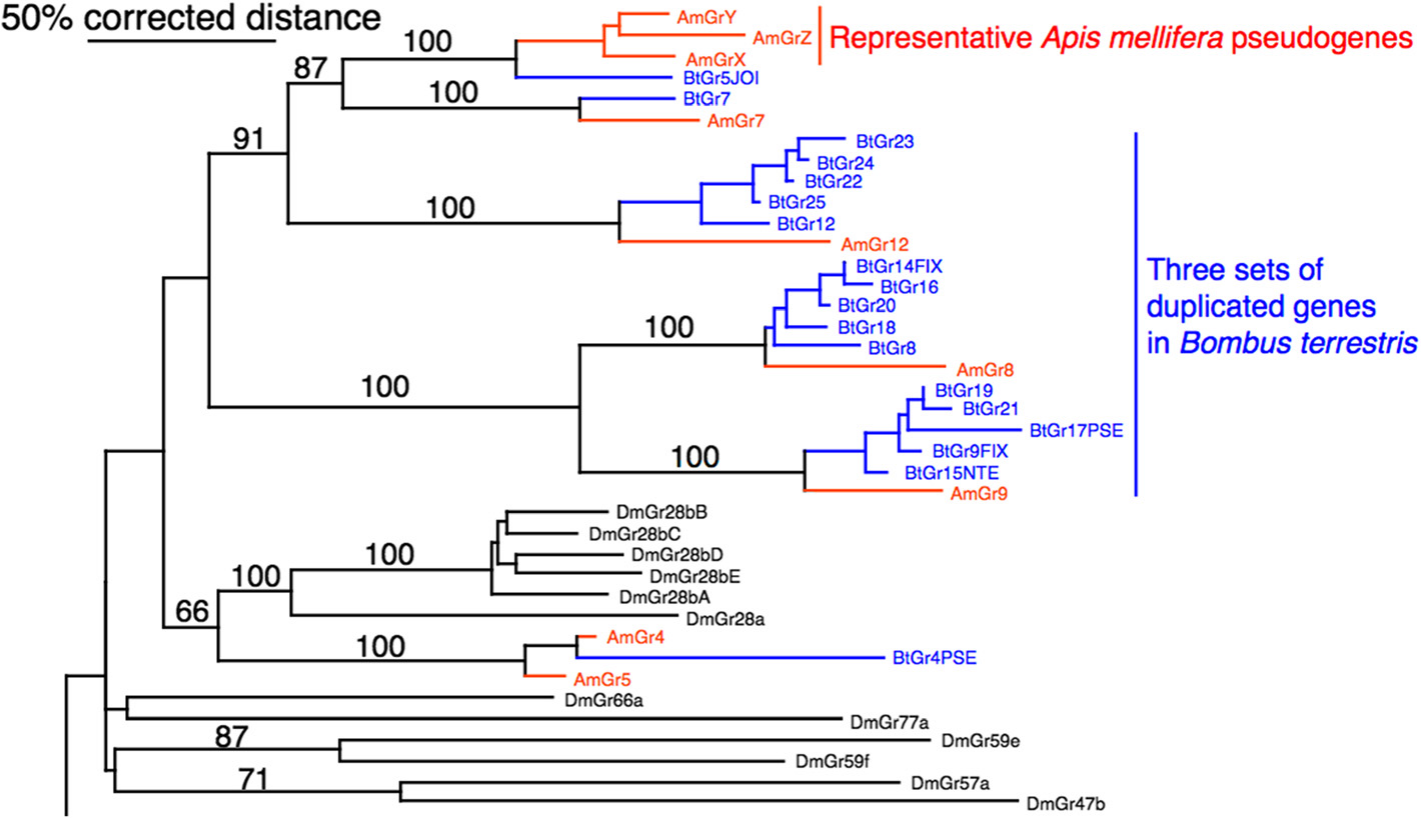
Section of the phylogenetic tree of the *Bombus terrestris*, *Apis mellifera*, and *Drosophila melanogaster* gustatory receptors (GRs) showing the impressive *B. terrestris-*specific expansion. This is a corrected distance tree. *B. terrestris* and *A. mellifera* proteins are highlighted in orange and blue, respectively, as are the branches leading to them to emphasize gene lineages. Bootstrap support level in percentage of 10,000 replications of uncorrected distance analysis is shown above major branches. The full phylogenetic tree of GRs can be found in Additional file 1.

The most impressive feature of the GR gene family in *B. terrestris* is the expansion of three *A. mellifera* GRs into 15 genes in *B. terrestris* (Figure 3). These expansions are all very recent, being unique to *Bombus*, and have short branches to each new gene. Thus, while the total GR family size in *B. terrestris* is considerably larger than that of *A. mellifera*, the difference is not an ancient one involving the loss of *Apis* genes, but rather a lineage-specific and recent expansion in bumblebees. Unfortunately, there is little information on what ligands these novel *Bombus-*specific GRs might detect, but it is likely that they are bitter taste receptors [141], perhaps related to the more diverse nest-building habits of bumblebees.

#### Ionotropic receptors (IRs)

The IR family also contributes to insect olfaction and gustation. *Apis mellifera*, *Nasonia*, and various ants do not differ much in their repertoire sizes [11,12,131], and the IR family in *B. terrestris* is comparable, with 22 genes (Additional file 1). The IR family contains several conserved orthologous genes shared across insects. The co-receptor IR8a and 25a genes are unusually highly conserved and cluster confidently with the ionotropic glutamate receptors from which they clearly evolved [131]. They were therefore used as an outgroup to root a phylogenetic tree of IRs (Additional file 1). Somewhat surprisingly, IR25a has a duplicate gene copy in *B. terrestris* (BtIR25a.2) that is rapidly evolving, and encodes only the second half of the protein. While this could be a pseudogenic copy, it has the hallmarks of a functional gene, and is the only known instance of a duplication of IR25a to date. The other orthologous lineages are rather more rapidly evolving, including IR93a, 76b, and 68a. *A. mellifera* and *B. terrestris* have several highly divergent IRs, only one of which (IR218) was noted in Croset *et al.* [131]. The new genes are IR328-339, which mostly have simple orthologs in both species, except that AmIR338 is a pseudogene in *B. terrestris*, while *B. terrestris* has a paralog of IR332, numbered IR333, which has been lost from *A. mellifera.*

#### Odorant binding proteins (OBPs)

Finally, OBPs are involved in the initial transport of odorants from the air to the sensory neuron dendrites in olfactory sensilla. The classic OBPs were the only sub-family identified in *A. mellifera*, and this is also the case for *B. terrestris*. While *A. mellifera* have 21 OBPs [142], there are 16 members in *B. terrestris* (Additional file 1), with eight of the 16 being simple orthologs with eight of *A. mellifera* OBPs. There is some species-specific gene duplication and loss (Additional file 1). Most impressive, however is the evolution of AmOBP13 and its relatives AmOBP14-21. As noted in [142], the latter are a large tandemly duplicated set derived from AmOBP13. In *B. terrestris* there are just three genes duplicated from an ortholog to AmOBP13. However, the functions of these proteins in honeybee and bumblebee biology are largely unknown.

Thus, the chemosensory repertoire of bumblebees appears to emphasize gustation over olfaction relative to honeybees. In addition, within each chemoreceptor family there has been an expected pattern of gene birth and death, even when gene numbers are comparable between *A. mellifera* and *B. terrestris*. However, the ligand specificity of just one *A. mellifera* OR is known [136], so extensive work will be required to identify precisely how their chemosensory abilities have changed to suit their different social and ecological situations.

### Defense and venom constituents in the bumblebees

Defense and venom constituents in the bumblebees were characterized by incorporating information from a venom proteome of *B. terrestris* workers and the bumblebee genomes. The proteomic analysis revealed 519 unique peptides and provided evidence for 57 venom proteins in *B. terrestris*. Of these, 52 were previously not described for *B. terrestris* venom. Manual annotation of genes supported by the venom peptides (Additional file 2) showed that most venom genes are fully (72%) or partially (23%) covered by transcriptomic evidence. Venom proteome evidence was not found for several well-characterized honeybee venom compounds, although based on searches for syntenic regions and homology, five of these missing components were recovered from the *B. terrestris* genome (Additional file 2). However, *apamin* and *tertiapin*, two neurotoxic honeybee venom compounds, were not found in the bumblebee genomes, including when searching initial sequence reads. Highly similar protein sequences to those identified in *B. terrestris* were identified in *B. impatiens* (Additional file 2). Additional details can be found in [143].

### Immune components and responses in the bumblebees

Given the perceived high risk of disease in densely packed social groups, it was surprising when *A. mellifera* was found to have only one-third as many immune-related genes as solitary Dipteran model insects [144]. Dietetic differences, artifacts of honeybee breeding, and advanced eusociality allowing for complex group-based defenses or hygienic behavior, were all possible reasons advanced for the presence of this depauperate immune complement. Comparatively examining immune genes in bumblebees addresses some of these hypotheses. Furthermore, given the potential role of parasites in concerning declines of some bumblebee species [40,48], understanding the architecture of the bumblebee immune system has a clear importance.

**Figure 4:**
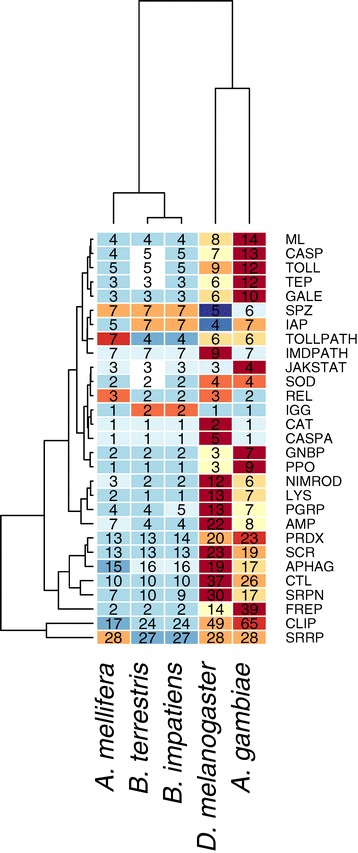
Immune gene counts in bumblebees relative to selected insects. Number of genes belonging to 29 categories of immune genes are presented in the cells. Heat colours in a cell reflect the number of genes in that category relative to those other species (light blue: fewer - dark red: more). The tree represents a clustering analysis using Euclidean distances based on the number of genes within these groups. AMP = Antimicrobial peptide, APHAG = Autophagy, CASP = Caspase, CASPA = Caspase A, CAT = Catalase, CLIP = CLIP serine protease, CTL = C-type lectin, FREP = Fibrinogen-like, GALE = Galectin, GNBP = Gram-negative binding protein/Beta-glucan recognition protein, IAP = IAP repeat, IGG = Immunoglobulin, IMDPATH = Imd pathway, JAKSTAT = JAK/STAT pathway, LYS = Lysozyme, ML = MD-2-related lipid recognition, NIMROD = nimrod, PGRP = Peptidoglycan recognition protein, PPO = Prophenoloxidase, PRDX = Peroxidase, REL = Relish, SCR = Scavenger receptor, SOD = Superoxide dismutase, SPZ = Spatzle, SRPN = Serine protease inhibitor, SRRP = Small RNA regulatory pathway, TEP = Thioester-containing protein, TOLL = Toll genes, TOLLPATH = Toll pathway.

Both bumblebee genomes contain components of all major immune pathways described in insects and exhibit a similar immune repertoire to *A. mellifera* (Additional file 2). The total number of immune genes in bumblebees is similar to *A. mellifera*, and therefore also considerably lower than in Dipteran model species (Figure 4). While numeric representation of immune components is similar, the bumblebee immune repertoire is not however completely undifferentiated from that of the honeybee. Both *Bombus* species have only a single copy of the antimicrobial peptide (AMP) *defensin*, which is present in two copies in *A. mellifera*, and have an expanded set of serine protease inhibitors. In *B. terrestris*, there are five, highly similar (average 75% sequence similarity), putative serpin 3/4-like genes, while only a single ortholog is identified in *A. mellifera*. A homolog of the apoptosis-involved caspase *decay*, which has not been described in either *A. mellifera* or the parasitoid wasp *N. vitripennis*, and a Hymenoptera-specific clade of caspases that are most similar to *Ice* in *Drosophila* are also present. A recently duplicated species-specific peptidoglycan receptor protein (PGRP) is present in *B. impatiens.* Further in-depth analyses are reported in a companion paper on immune genes [145]. Quantitative expression analyses in *B. terrestris* confirm expression changes of many immune-related genes following immune-stimulation. Interacting with parasites, including those that are co-evolving, make immune genes an interesting focus of molecular evolution studies. In the bumblebees, patterns of evolutionary selection differ across immune system components, with certain genes showing lineage-specific patterns of selection. Broadly however, the comparative analysis of immune genes present in the two bumblebee genomes show a reduced immune complement is not the result of honeybee-specific traits or those relating to complex social defenses in advanced eusocial organisms, such as hygienic behavior, but is instead basal in the bees and independent of the level of sociality [145].

#### Bumblebee queen hemolymph proteome

To further improve our understanding of the immune potential of *B. terrestris* queens and to cross-check the draft genome with further proteomic data, a proteomic analysis of the hemolymph, an important transporter of metabolic and immune components, and site of immune activity, was conducted. In total 821 peptides were identified (Additional file 2) representing 98 proteins, 46 of which had associated immune function. Gene ontology mapping also indicated that the hemolymph proteome comprised proteins associated with primary and secondary metabolism, protein transport, olfaction, chemosensory processes, and venom. The presence of venom and chemosensory proteins in the hemolymph may represent novel functions and processes for these proteins.

A relatively large proportion of the hemolymph proteome (17 of 98 proteins) comprises proteins of unknown function. These genes were provisionally annotated as hemolymph associated proteins (HAP 1-14) with three proteins grouped into a novel protein family (HAP family A1-3). Homology searches indicated that many of the HAPs show restricted taxonomic distribution including genes specific to *Bombus* (n = 2), Apidae (n = 2), Apoidea (n = 1), Hymenoptera (n = 7), and Hexapoda (n = 5) (Additional file 1). A comparison of proteomic data for *A. mellifera* queen hemolymph [146] identified ubiquitous proteins involved in defense-related processes, such as immunity and antioxidant activity. However, a significant proportion (over 45%) of the proteomes vary in composition, with the majority of this variation being attributed to the *B. terrestris* HAPs indicating a potential role in the behavioral, physiological, and social differences observed between these two species. The mass spectrometry data generated here was utilized in a proteogenomic capacity to identify missed protein coding genes (12 genes in total) and correct inaccurate gene models.

### Regulation of gene expression in the bumblebee genomes

Gene regulation is likely to be especially important in eusocial organisms, where it will shape behavioral and developmental differences between castes.

#### RNAi

RNAi leads to sequence-specific gene silencing, which plays a role in immunity against viruses and mobile genetic elements, gene regulation, and cellular development [147]. The functionality of the RNAi machinery has been demonstrated in bumblebees [148], but the genes involved had, as yet, not been identified. In both bumblebee species homologs for genes encoding the core RNAi machinery proteins were found (that is, *dicer*, *drosha*, *argonaute*, *aubergine*, *pasha*, *R2D2*, *loquacious*) (Additional file 1). In addition, genes involved in the uptake and spread of the silencing signal were found to be consistent with the findings from *A. mellifera*, with *Snipper* and *sid-1* homologs being present, but a *sid-2* homolog being absent. Phylogenetic analysis of the bumblebee SID homologs shows they cluster with SID proteins from other Hymenoptera (Additional file 1).

#### MicroRNAs

MicroRNAs (miRNAs) are a class of small non-coding RNAs that regulate gene expression at the post-transcriptional level, and several studies have shown that miRNAs are implicated in the regulation of social behavior in social insects in general [9,14]. In honeybees, miRNAs have been associated with development [149], queen-worker caste differentiation [150], and task specialization and polyethism in worker bees [151,152]. Most recently, miRNAs have been shown to play a role in response to some of the physiological changes associated with vitellogenin in worker bees [153].

**Figure 5:**
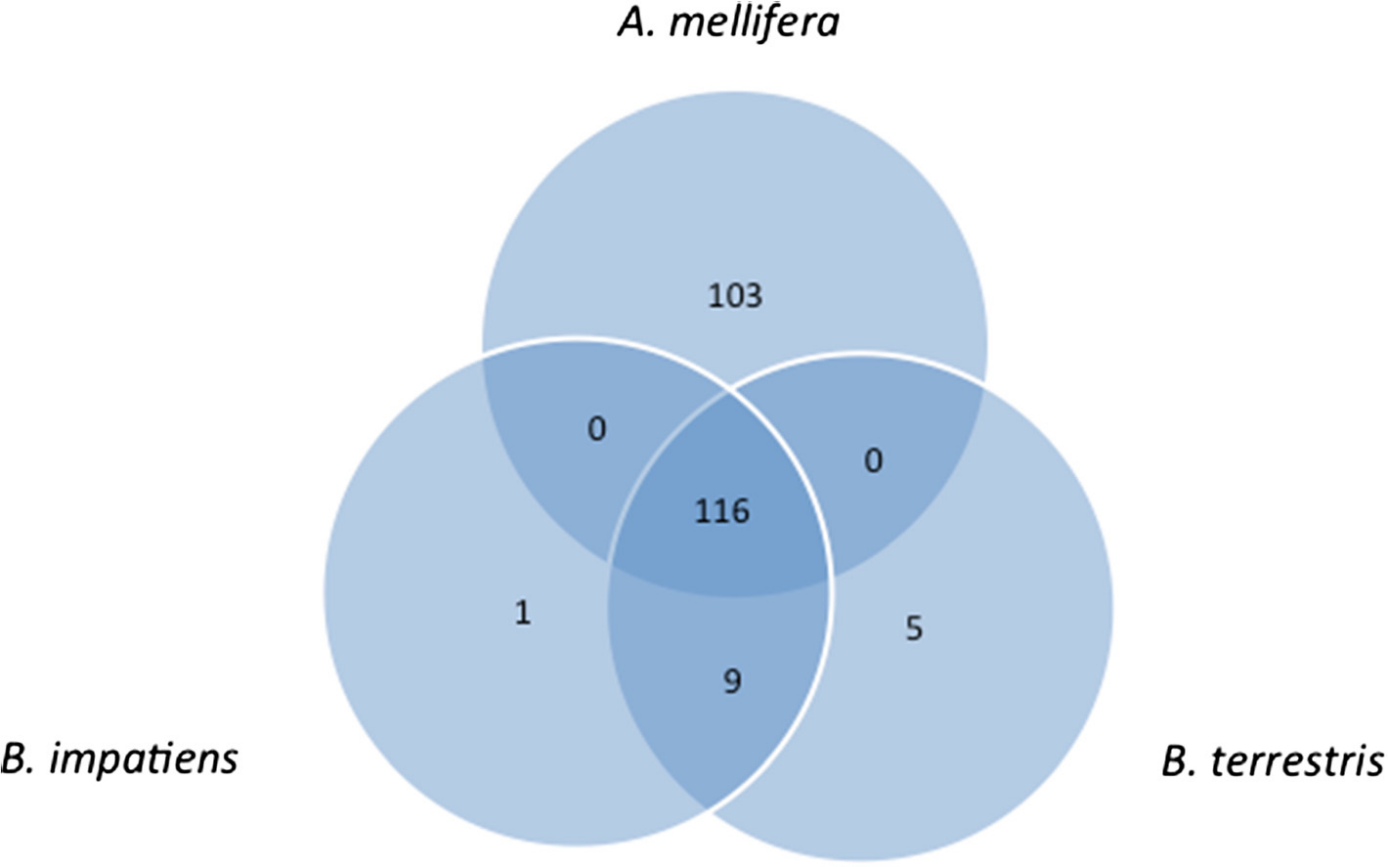
Venn diagram of the distribution of unique and shared miRNAs across the two bumblebee species investigated and *Apis mellifera*. A total of 116 miRNAs were found in the genomes of all three species. Strikingly, 103 miRNAs of the 219 in *A. mellifera* were not found in the genome of either bumblebee species.

Here, a diverse complement of methods was used to identify miRNAs in the bumblebee genomes, including sequencing of miRNAs in *B. terrestris*, a homology search of *A. mellifera* miRNAs from miRBase [154], and miRNA prediction using miRCat [155] and miR-abela [156]. These methods identified 130 and 115 miRNAs in *B. terrestris* and *B. impatiens*, respectively (Additional file 2), including a number of previously uncharacterized miRNAs in *B. terrestris*. Of these bumblebee miRNAs, 17 miRNAs had not previously been identified in *A. mellifera*. Comparing new miRNAs back to the *A. mellifera* genome revealed that two miRNAs are conserved across the two bumblebee species and *A. mellifera*, but 14 miRNAs were unique to one of the two bumblebee species, with five being unique to *B. terrestris* and one potentially representing a unique duplicated miRNA in *B. impatiens* (Figure 5).

Those miRNAs with homologs in *A. mellifera* and the two bumblebees are highly conserved, being identical or near identical. This fits with expectations from models of miRNA evolution [157]. However, mir-263a and mir-3736 showed substitutions in bases 2 to 8 of the mature miRNA. This is the ‘seed region’, that is, where miRNAs bind to the target transcript, so substitutions here are most likely to change the function of the mature miRNA. Historically, the most expressed sequence has been classified as the mature miRNA and this is the sequence that is assumed to have a regulator function, while the other sequence (historically termed the *sequence) is lost or degraded. It is becoming increasingly clear that sometimes these sequence arms switch between species, so the *sequence in one species might not be the same in another [158]. In addition, it is clear that, for some miRNAs, both arms of the duplex have biological activity, and the most highly expressed will vary in different tissues. Ten miRNAs were found to have switched arms, with the most abundantly expressed arm in *B. terrestris* being the opposite of that in *A. mellifera*. This implies that while the sequences of the miRNAs themselves were conserved, functional changes could have happened due to such switches.

In spite of the conservation of several miRNAs between *A. mellifera* and the bumblebees, there is a large disparity with 103 of the 217 *A. mellifera* miRNAs apparently being exclusively found in the *A. mellifera* genome (Figure 5). *A. mellifera* has 217 miRNAs identified in miRBase, but for at least 97 of these there is very little or no evidence that they are present in the genomes of the two bumblebee species, and neither is there evidence (according to miRBase) that they are present in other species with sequenced miRNAs. Furthermore, six *A. mellifera* miRNAs are duplicated in the *A. mellifera* genome, but have only one copy in the bumblebee genomes, meaning there are currently 103 miRNA genes that appear exclusively in the *A. mellifera* genome. These differences in miRNA numbers between honeybees and bumblebees are surprising given that new RNAs are thought to evolve and be maintained at a low rate [157]. For example, one study of miRNA evolution in *Drosophila* found that species diverged by up to 60 My were far more similar in their miRNA expression profiles [159]. However, insects appear to have a particularly high rate of miRNA generation and disappearance [159] and an especially high rate in *A. mellifera* (but not *Bombus*) could explain the disparity in numbers with bumblebees. An alternative is that there are a high number of false positives in the *Apis* miRNA set, an issue that was guarded against in the identification of unique bumblebee miRNAs by taking into account several parameters in the prediction process [155]. However, this would not account for the miRNA duplications in *A. mellifera*.

The results of the miRNA analysis show that despite conservation of a core set of miRNAs between bumblebees and honeybees, there are important differences that likely affect miRNA functionality. Given the role that miRNAs have been shown to have in traits relating to social behavior, these differences are striking and may underlie key biological differences between advanced eusocial honeybees and primitively eusocial bumblebees.

#### DNA methylation

DNA methylation plays an important role across taxa in epigenetic modification that alters expression patterns, and in this way it can impact on development, behavior, learning, memory formation, and phenotypic plasticity [160]. In honeybees, DNA methylation has an important role underlying eusocial characteristics, influencing developmental divergence of queens and workers, and changes in worker behavior [161-163]. Thus, DNA methylation is an important process to investigate and understand in bumblebees. While not as distinct as in honeybees, caste differences in bumblebees are also marked, and in *B. terrestris* methylation is associated with plastic reproductive division of labor [164]. DNA methylation in the two bumblebee genomes has many defining features that are similar to those of *A. mellifera* (Additional file 1). This indicates that DNA methylation may play an equally important role in directing caste differentiation and behavioral divergence in bumblebees as it does in their advanced eusocial relatives. Both bumblebee genomes have a complement of DNA methyltransferases (DNMTs) similar to *A. mellifera*, including two copies of the maintenance methyltransferase (*DNMT1*) and one *de novo* DNA methyltransferase (*DNMT3*) (Additional file 1). Enzymes functioning in DNA methylation targeting (*PIWI*) and removal (*TET*) are also present. A computational prediction of the methylation landscape of the bumblebee genomes based on CpG depletion demonstrated that it is very similar to that of *A. mellifera*, and half of all genes can be methylated, with DNA methylation primarily targeted to exons (Additional file 1). Based on gene ontology terms, genes with metabolism and ubiquitous housekeeping functions were significantly enriched for genes with predicted methylation (Additional file 1). The similarity in methylation between *A. mellifera* and the bumblebees was further confirmed by DNA methylation quantification, with 0.5 to 0.6% of all CpGs being methylated (Additional file 1).

### Selenoproteins and selenoprotein-related genes in the bumblebee genomes

Selenoproteins are a class of proteins that include selenocysteine (Sec), an unusual amino acid inserted through the recoding of a UGA codon (normally a translation stop). The number of selenoproteins encoded in genomes varies among eukaryotic lineages [165]. While other animals show extensive conservation of selenoproteins, some insect lineages have been reported to be devoid [166]. Interestingly, this is attributed to multiple independent events of loss in the different lineages. The bumblebee genomes, like all other Hymenoptera investigated thus far, lack selenoproteins. They have also lost part of the machinery necessary to build and insert selenocysteine. The bumblebee genomes possess no *tRNAsec*, *eEFsec*, and *pstk*, while they retain *SecS*, *secp43*, and *SBP2*. These losses fit with the mosaic pattern of selenoprotein loss through multiple independent events in insects due to relaxed selective constraints, with Lepidopteran, Hymeopteran, Dipteran, and Coleopteran lineages retaining varying complements of selenoprotein machinery [166]. Given their conservation across multiple Hymenopteran species, the retained proteins have probably acquired a function unrelated to selenocysteine. There is conservation in the number of Sec machinery genes found, and in their exonic structure, between the bumblebees and *A. mellifera*. The extinction of selenoproteins in the Hymenoptera opens an interesting field of research for the study of evolution of selenoprotein genes and selenium metabolism in insects.

### Using the bumblebee genomes: an example of genome-wide patterns of diversity based on SNPs in *B. impatiens*

High quality genomes provide important resources for post-genomic research, including population-level assays of single nucleotide polymorphism (SNP) variation that can be challenging when no reference is available. The newly sequenced *Bombus* genomes will provide a major resource for studies of genetic diversity, demographic history, natural selection, and genome-wide associations with disease, for example, that may promote understanding of factors involved in pollinator declines. To illustrate the value of the *Bombus* genome for next-generation SNP analysis, RAD-tag sequencing [167] was carried out on 22 *B. impatiens* worker samples collected throughout the geographic range of this species (Additional file 1), and reads were mapped to the *B. impatiens* genome. After stringent filtering, 9,607 SNPs were identified across the 22 diploid individuals over 1.113 Mb of sequence. Genome-wide diversity was estimated at *θ*_*pi*_ = 0.0014 per site, consistent with previous *de novo* analyses of RAD-tags [168]. The SNP positioning information from the *B. impatiens* genome enables population genetic assessment of linkage disequilibrium (LD) that was not possible for *de novo* RADseq analyses. Overall LD was low and declined rapidly with distance between SNPs: average *r*^2^ within 10 kb was 0.15 and decayed to 0.05 over larger distances. Such weak LD is consistent with *A. mellifera* subspecies that experience recombination rates of 19 cM/Mb [169] and a similarly high rate is also present in bumblebees [56]. Weak LD is expected for the large population sizes typical for *B. impatiens* [40]. The two bumble bee genomes will thus provide an excellent reference that enables resequencing studies in *B. impatiens* and *B. terrestris*, and furthermore, because of the substantial synteny revealed here, will also become powerful resources for positioning and annotating data in other closely related *Bombus* species.

## Conclusions

In addition to providing an excellent basis for future post-genomic studies, the two bumblebee genomes presented here illuminate key aspects of the biology of these important pollinator insects, and, based on comparisons with honeybees, offer an insight into potential foundations of advanced eusociality. Given the plethora of differences between the two bumblebees investigated here and *A. mellifera* (Table 1), it is surprising that in general the gene repertoires of the species are highly similar. Some of these similarities are informative in themselves, and demonstrate that certain genome characteristics found in *A. mellifera* are not unique. For example, depauperate complements of xenobiotic detoxification and immune genes in comparison with many other insects are not honeybee-specific. With regard to protein-coding genes, striking differences relate to chemosensation, with bumblebees emphasizing gustation relative to olfaction, which is likely tied to specific ecology of these species.

While mechanisms of gene regulation are known to have an important role in insect societies, the surprising divergence in miRNA complement and potential functionality represents a major difference between the primitively eusocial bumblebees and advanced eusocial honeybees. It may well be that these elements are what tune complex behavior and organization in the advanced eusocial bees. However, selection on gene regulation, potentially driven or limited by environmental constraints (for example, temperate and tropical, annual and perennial) could underlie other key biological differences aside from the level of eusociality.

In general, the gene repertoires reported here for two key bumblebee species suggest that the evolution of advanced eusociality in bees did not occur through large leaps involving notable gene expansions and/or depauperation between lineages. Rather, the route to advanced social living was mediated by many small changes in many genes and processes.

## Methods

### Genome sequencing and assembly

#### Bombus terrestris

DNA from a single haploid drone was used for XLR fragment data. Pools of haploid drones were used for the 8 kb and 20 kb libraries. Drones were provided by the ETH group of Paul Schmid-Hempel, Switzerland. These drones were the offspring of field-caught queens collected in the spring of 2008 in Northern Switzerland. DNA extraction was carried out using Genomic tips and the blood and cell culture DNA kit (Qiagen). A whole genome shotgun sequence was generated using the Roche 454 platform, with fragment (NCBI SRA: SRX016989) and mate-pair (NCBI SRA: SRX016990, SRX016992, SRX016991) DNA libraries. Library preparation and sequencing protocols were carried out as previous described [14]. The *B. terrestris* assembly was produced by assembling the approximately 14.3 million Roche 454 reads representing approximately 21× coverage of the genome. Sequences were combined with the Newbler-assembler (2.3-PreRelease-10/19/2009) and then reads from each Newbler generated scaffold were grouped, along with any missing mate-pairs, and reassembled using Phrap in an attempt to close the gaps within each Newbler scaffold.

#### B. impatiens

DNA extracted using a standard chloroform/phenol procedure was used from a single drone taken from a colony obtained from Koppert USA. Sequence was generated on an Illumina GAIIx sequencer at the University of Illinois. Libraries of 500 bp, 3 kb, and 8 kb were prepared using standard Illumina protocols and sequenced, producing a total of 497 million reads and representing approximately 108× raw coverage of the genome (NCBI SRA: SRX679085, SRX679084, SRX679082). Read lengths were in the range of 100 to 124 bp. Duplicates were removed and all reads were error-corrected with the Quake program [170]. After error correction and duplicate removal, 276 million reads remained which were assembled using both CABOG and SOAPdenovo. The final assembly used SOAPdenovo release 1.9 with a k-mer size of 47. The final assembly contained 5,559 scaffolds ≧200 bp, of which 1,505 were >1,000 bp. The 5,559 scaffolds contained 16,215 contigs, while the 1,505 scaffolds contained 12,033 contigs.

### Synteny

Both the Bter_1.0 and the BIMP_2.0 assemblies were scanned for microsatellite loci and compared based on sequence similarity to identify loci shared (that is, ‘homologous’) between both species. The procedure is described in detail in [171]. The relative positions and order of homologous loci were compared. Using the Bter_1.0 assembly as a reference, homologous scaffolds were identified, ordered, and oriented. If two scaffolds had consecutive homologous loci at their ends, they were considered linked. Single loci, missing in the consecutive order or those homologous to a distinct scaffold than the surrounding loci in the scaffold were ignored, whether at the ends or within scaffolds. As an exception to this, loci homologous to unplaced scaffolds were included, but only where several consecutive loci justified the position of the unplaced scaffold within gaps or at scaffold ends. This way, based on marker order and distances, previously unmapped small scaffolds and contigs could be putatively placed and were considered in the analysis of the synteny lengths if they contributed to an extension or linkage between scaffolds.

### Repetitive elements

Repetitive elements were detected and annotated with the REPET software package ([172], version 2.0). First, repeated sequences were detected by similarity (all-by-all blast using BLASTER) and LTR retrotransposons were detected by structural search (LTRharvest). The similarity matches were clustered with GROUPER, RECON, and PILER, the structural matches with single-linkage NCBI BLASTCLUST. From each cluster a consensus sequence is generated by multiple alignment with Map. The consensus sequences were analyzed for terminal repeats (TRsearch), tandem repeats (TRF), open reading frames (dbORF.py, REPET), and poly-A tails (polyAtail, REPET). Furthermore, the consensuses were screened for matches to nucleotide and amino acid sequences from known transposable elements (RepBase 17.01, [57]) using BLASTER (TBLASTX, BLASTX) as well as searched for HMM profiles (Pfam database 26.0, [173]) using hmmer3. Based on the detected structural features and homologies, the consensuses are classified by PASTEC according to [174]. Redundancies are removed (BLASTER, MATCHER) as well as elements classified as SSRs (>0.75 SSR coverage) or unclassified elements built from less than 10 fragments. This set of *de novo* detected repetitive elements was used to mine the genome in the second pipeline with BLASTER (NCBI BLAST, sensitivity 4, followed by MATCHER), RepeatMasker (NCBI BLAST/CrossMatch, sensitivity q, cutoff at 200) and CENSOR (NCBI BLAST). False positive matches were removed by an empirical statistical filter. Satellites were detected with TRF, MREPS, and RepeatMasker and were then merged. Furthermore the genomic sequences were screened for matching nucleotide and amino acid sequences from known transposable elements (RepBase 17.01) via BLASTER (TBLASTX, BLASTX) followed by MATCHER. Finally a removal of TE doubletons, removal of SSR annotations included into TE annotations and ‘long join procedure’ to connect distant fragments was performed. Sequences from the *de novo* repetitive element library found to have at least one perfect match in the genome were then used to rerun the whole analysis. To ensure compatibility and to avoid bias, a manual curation or clustering of the *de novo* detected elements was not performed before mining the genome. However, *post hoc* all elements were manually analyzed that were previously classified into class I retrotransposon or class II DNA transposon elements or unclassified elements with detected coding element features (similarity to known transposable elements) due to potential chimeric insertion. At this stage derivative elements (LARD, TRIM, MITE) were excluded from detailed further inspection unless carrying such a feature. Elements classified as ‘potential Hostgene’ or unclassified elements (noCat) were also excluded. Manual inspection was carried out with ORF Finder (NCBI), CDD search (NCBI, [175]), with a search in the most up to date online RepBase database (accessed December 2012 to February 2013) via CENSOR [176] and phylogenetic analysis for LINE RT domains with RTclass1 [177] in order to achieve a detailed classification for each element, determine its potential relation to a family of known elements, to evaluate the completeness, and to detect potential active elements. Elements were defined as complete if they possessed the relevant coding parts with the element-typical domains and the structural features (LTR, TIR). The potential activity was defined according to the region an intact ORF, if present, covered. If an intact ORF seemed to cover a complete region including the typical domains (for example, GAG, POL, Tase) then the element is considered to potentially active. If a Tase domain is covered by a truncated ORF or the Tase itself appears to be truncated but is covered by an intact ORF, or if the RT domain is covered by an active ORF but not the remaining element-typical domains, then the element is considered to be potentially active. During the manual classification to at least superfamily level, novel transposable element types not covered by the system of [174] were also considered: Kolobok, Sola, Chapaev, Ginger, Academ, Novosib, and ISL2EU class II DNA transposons [178,179]. Simple sequence repeats and other low complexity regions were extracted from the REPET pipeline database and processed to calculate the total coverage of these types of repetitive DNA, while omitting those overlapping with transposable element annotation.

### Gene predictions

#### NCBI Refseq and Gnomon

Bter_1.0 and BIMP_2.0 assemblies were annotated with NCBI’s eukaryotic genome annotation pipeline (v.3, see [180]). Evidence used for *B. terrestris* included Refseq protein annotation of *A. mellifera*, 214 k TSA assemblies of Illumina RNAseq reads from *B. terrestris* queen heads (NCBI SRA: SRX090531), queen ovaries (NCBI SRA: SRX090532) and male heads (NCBI SRA: SRX090533), Roche 454 *B. terrestris* RNAseq reads (NCBI SRA: SRX040734 and ERP000936), and additional cross-species protein alignments. Evidence used for *B. impatiens* included the above, plus 406 k of Roche 454 RNAseq reads (NCBI SRA: SRX040732).

#### AUGUSTUS

AUGUSTUS can be used as an *ab initio* gene prediction tool, but can also integrate extrinsic evidence from various sources [181]. Training gene structures for *B. terrestris* were generated using transcriptome data and an AUGUSTUS parameter set for *A. mellifera* [16]. RNAseq data mentioned above were mapped to the genome using BLAT [182] and alignments were integrated into gene predictions using AUGUSTUS. RNAseq data were mapped to predicted genes and fully covered transcripts selected as training genes to optimize a species-specific parameter set, with the flanking region being set to 10,000 nucleotides and UTR parameters adopted from *A. mellifera*. Final gene predictions were made using the *B. terrestris* parameter set, the above-mentioned RNAseq evidence, available peptides [143] and repeat information [183]. Greater weight was given to informing sequences from the target species. Genes in *B. impatiens* were predicted using the *B. terrestris* parameter set. Extrinsic evidence was generated as described for *B. terrestris*, without the peptide data, and with *B. impatiens* repeat information [184].

#### Fgenesh++

Predictions were made using FGENESH 3.1.1 [185]. RNAseq data for *B. impatiens* and *B. terrestris* described above were incorporated, along with the GenBank NR database to predict genes similar to known proteins.

#### GeneID

GeneID [186] is an *ab initio* gene prediction program used to find potential protein-coding genes in anonymous genomic sequences. An initial training set, as used in AUGUSTUS, was used to develop a *B. terrestris* specific parameter file based on a method employed to obtain a *D. melanogaster* parameter file [187].

#### SGP2

SGP2 [188] combines *ab initio* gene prediction (GeneID) combined with TBLASTX searches between genomes. Genomes of *Nasonia giraulti*, *N. longicornis*, and *N. vitripennis* were used as reference to develop the *B. terrestris* parameter file. The *B. terrestris*-specific parameter file was produced based on the methodology described to obtain a human sgp2 parameter file [189].

#### N-SCAN

The N-SCAN package [190] was used to leverage conservation between the target genome, *B. impatiens* or *B. terrestris*, and genomes of two informant bee species, *A. mellifera* (Amel_4.5) and the other *Bombus* species (Bter_1.0 or BIMP_2.0, respectively). The target *Bombus* species was masked for simple sequence repeats using RepeatMasker [191]. LASTZ [192] was run using default parameters with the target *Bombus* genome and each informant genome. For *B. terrestris*, iParameterEstimation was used to generate both a Bter_1.0-Amel_4.5 specific parameter set as well as a Bter_1.0-BIMP_2.0 specific parameter set using the training set described for AUGUSTUS, including UTR features. N-SCAN was run using each of the *B. terrestris* specific parameter sets with the respective LASTZ informant genome alignments to produce two N-SCAN gene prediction sets, one based on Amel_4.5 and the other based on BIMP_2.0 as the informant genomes. The Amel_4.5 as the informant set was chosen as the best prediction set based on Eval analysis [193] against the RefSeq and Gnomon annotations for *B. terrestris*. Insufficient *B. impatiens* transcriptome data were available to generate BIMP_2.0-Amel_4.5 and BIMP_2.0-Bter_1.0 specific parameter sets, so the parameter files generated for Amel_4.5-Bter_1.0 (where Amel_4.5 was the target species and Bter_1.0 the informant species), Bter_1.0- BIMP_2.0, and Bter_1.0-Amel_4.5 were used to evaluate Bter_1.0 and Amel_4.5 as informant genomes for BIMP_2.0. N-SCAN was run using the Amel_4.5-Bter_1.0 parameter set with the LASTZ alignments between BIMP_2.0 and Amel_4.5, the Bter_1.0- BIMP_2.0 parameter set with the LASTZ alignments between BIMP_2.0 and Bter_1.0, and the Bter_1.0-Amel_4.5 parameter set with the LASTZ alignments between BIMP_2.0 and Amel_4.5. The set using the Amel_4.5-Bter_1.0 parameter set and Amel_4.5 as the informant genome was chosen as the best prediction set based on Eval analysis against the RefSeq and Gnomon annotations for *B. impatiens*.

#### GLEAN

Gene sets described above were combined with GLEAN, also using assembled transcript sequences described above and protein homologs. Transcript sequences were aligned to the Bter_1.0 and BIMP_2.0 genome assemblies using MAKER2 v2.15, which uses WU-BLAST [194] and Exonerate est2genome [195], with minimum 80% alignment coverage and 95% identity. Protein homolog alignments included SwissProt Metazoa homologs [196], *D. melanogaster* (r5.31) [197], *A. mellifera* (OGSv3.2) [16], *N. vitripennis* (OGSv1.2) [61], and the ants: *Acromyrmex echinatior* (OGSv3.8) [63], *A. cephalotes* (OGSv1.1) [13], *Camponotus floridanus* (OGSv3.3), *H. saltator* (OGSv3.3) [9], *Linepithema humile* (OGSv1.1) [11], *Pogonomyrmex barbatus* (OGSv1.1) [12], and *Solenopsis invicta* (OGSv2.2.3) [15]. Proteins in the SwissProt dataset annotated as transposable elements were removed prior to alignment. Protein sequences were aligned to the Bter_1.0 and BIMP_2.0 genome assemblies using Exonerate protein2genome with a minimum 60% identity and 60% alignment coverage.

#### Manual annotation

The annotation consortium used tools available at BeeBase (hymenopteragenome.org) and elsewhere to manually check certain gene models. Gene models and transcriptomic evidence were viewed and edited in Apollo [198].

### Orthology analysis

Orthology assignments were retrieved from OrthoDB [69]. OrthoDB6 includes a total of 45 arthropods with the following gene sets for the selected species: *Pediculus humanus* PhumU1.2 and *A. gambiae* AgamP3.6 from VectorBase; *N. vitripennis* Nvit_OGSv2.0, *A. mellifera* Amel_OGSv3.2, *A. cephalotes* Acep_OGSv1.2, and *A. echinatior* Aech_OGSv3.8 from Hymenoptera Genome Database; *B. impatiens* Bimp_RefSeq, *B. terrestris* Bter_RefSeq, and *A. florea* Aflor_Augustus from NCBI; *T. castaneum* Tcas_3.0 from BeetleBase; *B. mori* Bmor_GLEAN from SilkDB; and *D. melanogaster* Dmel_r5.45 from FlyBase. The maximum likelihood phylogeny was built using RAxML [199] from the concatenated multiple sequence alignments of 2,294 single-copy orthologs aligned with MUSCLE [200] and trimmed with TrimAl [201]. The superalignment contained 666,462 amino acids with 215,542 distinct alignment patterns.

### Protein domain analysis

Gene sets of all species (Additional file 1) were annotated with Pfam-Scan (based on HMMR3 [202]) against the Pfam A database (version of 4 October 2012) [173]. If there were different splicing variants, only the longest transcript was used.

*Unique domains:* A domain was considered as unique if it appears only in *B. terrestris* and in no other reference species. *Expansion and contraction of domains and arrangements:* All domains were counted just once for each gene within which they appear. Arrangements are considered as the combination of domains present in one protein, where the number and order of domains are not taken into account. *Repeats:* For the detection of repeats, genes were clustered according to their domain arrangement, again without considering the number and order of domains (just for the clustering). For each cluster in *A. mellifera* and *B. terrestris* the original repeat number was extracted. Each arrangement was analyzed for the minimal and maximal repeat count for each domain. If two domains cover one PFAM model consecutively, they are counted as one domain. Only arrangements appearing in both of the analyzed species were considered, and an arrangement needed to occur at least twice in at least one species.

### Development

Orthology of developmental genes was assigned using reciprocal BLASTP or TBLASTN searches using the *T. castaneum*, *A. mellifera*, and *D. melanogaster* protein sequences. Where necessary HMMer [202] was used to identify potential orthologs of fast-evolving genes in the bumblebee genomes.

### Sex determination

Orthologs for sex determination, germline development, and dosage compensation were identified in Refseq proteins and assembly scaffolds of the two bumblebee species by using BLASTP and TBLASTN using sequences from *A. mellifera*, *N. vitripennis*, and *D. melanogaster*. Manual annotation was performed by comparing Refseq bumblebee genes against available insect genomes in Apollo [198].

### Behavior, neurophysiology, and endocrinology

*Circadian clock genes:* Putative circadian clock genes were identified via TBLASTN searches of *A. mellifera* clock gene coding sequences against the bumblebee genome assembly. These putative homologs were then manually annotated in detail by comparison with homologs from other Hymenopteran species as well as expression datasets. Multiple sequence alignments were carried out with ClustalW [203]. *Take-out/Juvenile hormone binding proteins*: Refseq proteins and assembled genome scaffolds of the two bumblebee species were searched with BLAST for homologs of the *D. melanogaster To* gene or to JHBP genes of various insects. The SMART server [204] was used to demarcate JHBP domains and signal peptides of sequences, and SignalP server [205] was used to confirm putative signal peptides. Only domains with an *E*-value <0.1 were considered significant. Multiple sequence alignments were carried out with ClustalW. *Cys-loop ligand-gated ion channel gene superfamilies:* Putative *Bombus* cys-loop ligand-gated ion channel subunits were identified by TBLASTN using protein sequences of every member of the *A. mellifera* cys-loop ligand-gated ion channel superfamily [107] and then were manually annotated. *Bioamines/neuropeptides:* To identify neuropeptides, protein hormones, and their receptors, and biogenic amine receptors, TBLASTN searches were performed, using known insect, or arthropod sequences for these proteins. *TRP channel genes:* TRP channel genes in the bumblebee genomes were identified in the same way as previously for other insects [206].

### Xenobiotic detoxification enzymes and related genes

Cytochrome P450s, GSTs, and CCEs were manually annotated by comparing the genome sequence of each bumblebee with all annotated P450, GST, and CCE protein sequences from *A. mellifera*, *N. vitripennis*, and *D. melanogaster* using TBLASTN in a method similar to that used to annotate these genes in the *N. vitripennis* genome [122]. GSTs and CCEs were additionally compared to *A. florea*, and P450s to *P. barbatus*, *T. castaneum*, and *D. melanogaster*.

### Chemoreceptors

*Odorant receptors (ORs):* The OR family in *B. terrestris* was manually annotated using methods employed before for other insect genomes [132,133]. The BtOrs were numbered independently of their AmOr relatives, because while some are orthologs, much duplication and some gene losses make using the AmOr numbers for the BtOr genes impossible. The numbering does start with the conserved ortholog of AmOr1 as BtOr1, but diverges from there, in part because AmOr2 is now called Orco. Genome assembly problems associated with this gene family are noted in Additional file 1. Pseudogenes were translated as best possible to provide an encoded protein that could be aligned with the intact proteins for phylogenetic analysis, and attention was paid to the number of pseudogenizing mutations in each pseudogene. A 200 amino acid minimum was enforced for including pseudogenes in the analysis (roughly half the length of a typical insect OR). For phylogenetic analysis, the poorly aligned and variable length N-terminal and C-terminal regions were excluded (specifically 10 amino acids before the conserved GhWP motif in the N-terminus and 10 after the conserved SYFT motif in the C-terminus), as was a major internal region of length differences, specifically a long length difference region between the longer DmOr83b orthologs, now known as Orco proteins [135] and most of the other ORs. Other regions of potentially uncertain alignment between these highly divergent proteins were retained, because while potentially misleading for relationships of the subfamilies (which are anyway poorly supported), they provide important information for relationships within subfamilies. Phylogenetic analysis of this set of 342 proteins was carried out in the same fashion as for previous OR analyses [132,133]. *Gustatory receptors (GRs):* GRs were identified using the basic protocol referenced above for ORs. Numbering of the BtGrs is complicated. The names Gr1-4 and 6/7 and 10/11 were employed for the 1:1 orthologs of these genes in *A. mellifera*. However, while *A. mellifera* has paralogs Gr4/5, *B. terrestris* only has a single gene, so the BtGr5 name was employed for the intact ortholog of a large set of highly degraded pseudogenes in the *A. mellifera* genome, represented by constructs AmGrX, Y, and Z. *Bombus terrestris* has three very recent sets of duplicated genes, related to AmGr8, 9, and 12. These were named for their orthologs and then with additional numbers. Assembly problems related to genes in this family are noted in Additional file 1. For phylogenetic analysis, the poorly aligned and variable length N-terminal and C-terminal regions were excluded (specifically from 10 amino acids before the conserved GhWP motif in the N-terminus and five amino acids after the conserved TYhhhhhQF motif in the C-terminus), as was a major internal region of length differences involving DmGr66a. Including 68 GRs from *D. melanogaster*, phylogenetic analysis of a set of 107 total proteins was carried out in the same fashion as for previous GR analyses [132,133]. *Ionotropic receptors (IRs):* IRs were identified using the basic protocol above. Additionally, iterative searches were also conducted with each new *B. terrestris* protein as query until no new genes were identified in each major subfamily or lineage. Naming and numbering of the *B. terrestris* IRs is not simple. Following the example [131], the conserved orthologs of several IRs in other insects are given those names, specifically 8a, 25a, 93a, 76b, and 68a. Assembly problems related to genes in this family are noted in Additional file 1. The *A. mellifera*, *B. terrestris*, and *D. melanogaster* IRs were aligned in CLUSTALX v2.0 [207] using default settings. For phylogenetic analysis, the poorly aligned and variable length N-terminal and C-terminal regions were excluded, along with several internal regions of highly length-variable sequence. Other regions of potentially uncertain alignment were retained, because while potentially misleading for relationships of the subfamilies, they provide important information for relationships within subfamilies. Phylogenetic analysis of this set of proteins was carried out in the same fashion as for previous IR analyses [132,133]. *Odorant binding proteins (OBPs):* OBPs were identified as above for chemoreceptors. Because their phylogenetic relationships with the *A. mellifera* proteins are somewhat complicated, they were not named for their *A. mellifera* orthologs, but rather according to their locations in the genome, although the relatively conserved OBP1 genes are orthologous. Assembly problems related to genes in this family are noted in Additional file 1. Phylogenetic analysis employed corrected distance methods (see ORs methods) and only the mature proteins with signal sequences removed, as well as the different length C-termini.

### Defense and venom constituents

Venom proteome data [143] were used to search against the au5 (AUGUSTUS) and NCBI Refseq gene predictions for Bter_1.0, and genome six-frame translation databases using Mascot (v2.3, Matrix Science). Setting the significance threshold at *P* <0.01 leads to a peptide false discovery rate (FDR) of 5.34% for the au5 and 2.88% for the NCBI Refseq searches. Mass spectra data generated from all combinatorial peptide ligand library (CPLL) flow-through fractions, and the CPLL elution fractions of the Tris-glycine- and Tris-tricine-SDS-PAGE gel were separately searched against the genome six-frame translation database resulting in FDRs of 0.86%, 0.68% and 3.17%, respectively. Significant and top ranking peptides from the Mascot output with an ion score ≥30 were retained in the final peptide lists. All peptides found in the separate genome six-frame translation database searches were merged in one list and double peptides were removed. Identified *B. terrestris* venom proteins were used in BLAST searches against the *B. impatiens* Refseq database. The mass spectrometry proteomics data have been deposited to the ProteomeXchange Consortium [208] via the PRIDE partner repository with the dataset identifier PXD001623 and 10.6019/PXD001623.

### Immune components and responses

Using OrthoDB6 [69] we identified orthologs from the two bumblebees of previously characterized immune genes from other arthropods. To complement the orthology searches, we searched for homologs of known immune proteins in the two bumblebees using BLASTP against RefSeq proteins. To confirm the absence of any proteins that appeared to be missing, we searched the genome assemblies and short reads archive with TBLASTN. Further details, including evolutionary analyses using *Apis spp.* and *Megachile rotundata* are described in the companion paper [145]. *Queen hemolymph proteome*: Post-diapausing queen hemolymph proteome data obtained from a Thermo Scientific LTQ ORBITRAP XL mass spectrometer were searched against protein datasets derived from NCBI reference sequences (downloaded August 2013), an AUGUSTUS (au6) analysis of the genome and a transcriptome assembly [50] using MaxQuant (version 1.2.2.5; [209]). FDRs were set to 0.01 for both peptides and proteins, and proteins were considered identified when more than one unique peptide was observed. The queen hemolymph proteomic data have been deposited to the ProteomeXchange Consortium [208] via the PRIDE partner repository with the dataset identifier PXD001644 and 10.6019/PXD001644. Uploaded search result files were generated by searching mass spectrometry data against NCBI reference sequences using Proteome Discoverer (v1.4.0.288) and converted to pride.xml format using PRIDE Converter 2 [210]. Identified proteins were functionally annotated using Blast2GO v2.5 [211,212] and assigned gene ontology terms relating to biological processes, molecular function, and cellular component. Functional domain analysis was performed using InterProScan [213]. BLASTP searches were conducted in Blast2Go to determine the phylogenetic distribution of the hemolymph-associated proteins of unknown function. A reciprocal BLAST search (BLASTP, E-value cutoff of 1e-10, sequence similarity >25%) against protein data for *A. mellifera* hemolymph [146] was performed to identify homologous/orthologous proteins between the two hemolymph sets. All protein coding gene models were inspected and corrected in Apollo and missing genes were added to the manually curated gene set.

### Regulation of gene expression

*RNAi:* Genes putatively involved in RNAi from other insects (*D. melanogaster*, *A. mellifera*, *B. mori*, *T. castaneum*) were used to search both bumblebee genomes using TBLASTN. *microRNAs (miRNAs):* miRNAs were isolated by high-throughput sequencing of cDNA libraries from total RNA extracted from female larvae from four *B. terrestris* colonies. Libraries were prepared using the Illumina Trusec 2.0 kit, with modifications to reduce the risk of inherent sequencing biases [214]. Eight libraries (two from each colony) were prepared and sequenced by BaseClear B.V (Leiden, the Netherlands). Libraries were combined and mapped to the *B. terrestris* genome. MiRNA precursor sequences, identified by their characteristic hairpin-loop secondary structures, are processed into approximately 22 bp mature miRNAs that have biological function. To identify the mature miRNAs, first precursor sequences were identified by their secondary structures. For this purpose, miRNA prediction software miRCat [155] was used, employing sequence data and genomic context of the mapped sequences. Predicted miRNAs in *B. terrestris* were compared to the known miRNAs of *A. mellifera* published in miRBase [154]. Blast v.2.2.15 was used to search miRNAs not previously described from *A. mellifera* against the *A. mellifera* and *B. impatiens* genomes. To identify more miRNAs, including in *B. impatiens*, and the miRNAs not expressed in *B. terrestris* larvae, precursor sequences of all published miRNAs for *A. mellifera* from miRBase were used in a BLAST search of the *B. terrestris* and *B. impatiens* genomes. The miRNA prediction tool miR-abela [156] was used to identify hairpin-loop structures in the 500 bp regions around each of the identified homolog sequences. Finally, all *Bombus* homologs of the miRNAs that had been published in *A. mellifera*, but were not predicted by either miRCat or miR-abela, were assessed based on (a) showing a high mature sequence similarity to *A. mellifera* (>85%), (b) showing a clear hairpin secondary structure in their putative precursor sequences, and (c), in the case of *B. terrestris*, had been sequenced more than 100 times in the high-throughput sequencing libraries. These thresholds were selected to reduce the numbers of putative miRNAs that were false positives when identified from the BLAST searches. Sequence data used in these miRNA analyses are deposited in the NCBI Gene Expression Omnibus [GSE64512]. *DNA methylation*: *A. mellifera* or human proteins with known DNA methylation functions were used in a BLAST search of the bumblebee genomes. Methylated genes were predicted based on CpG depletion (CpG[O/E] <1) in the *B. terrestris* (n = 3,393) and *B. impatiens* (n = 3,671) genomes. The CpG[O/E] value (=#CpGs observed/#CpGs expected) was used to predict the presence of DNA methylation in a genomic region [215]. Global DNA methylation patterns in *B. terrestris* and *B. impatiens* were measured using the MethylFlash DNA quantification kit (Fluorometric) from Epigentek. We used thoraxes of newly emerged *B. terrestris* and *B. impatiens* workers, with similar *A. mellifera* samples as controls.

### Selenoproteins

The program Selenoprofiles [216] was used to search for all known selenoprotein families and Sec synthesis machinery genes. The program SECISearch3 [217] was run with permissive criteria to scan for SECIS elements (selenocysteine insertion sequences) downstream of potential selenoprotein candidates. All results were manually inspected and compared to other available insect genomes.

### SNP production and mapping in *B. impatiens*

To reduce genome complexity and enable sequencing from the same fraction of the genome across multiple individuals, we used restriction-site associated DNA marker (RAD) sequencing [167]. Genomic DNA was isolated from 22 *B. impatiens* workers from sites throughout the species’ geographic range. Samples were submitted to Floragenex (Oregon) for library preparation, sequencing, and preliminary bioinformatics [218-220]. Briefly, samples were digested with SgrAI, uniquely barcoded, and pooled. Fragments were sequenced from cut sites using single-end chemistry on an Illumina HiSeq 2000. Samples were demultiplexed and trimmed to a length of 90 bp. RAD-tag reads were mapped using the *B. impatiens* BIMP2.0 assembly. Sequences were aligned using BOWTIE 0.11.3 [221], taking into account sequence quality, allowing up to three mismatches, and ignoring reads that mapped to more than one location in the genome. Single nucleotide polymorphisms (SNPs) were called using SAMTOOLS 0.1.12a [222] with custom Floragenex scripts. Only variants with a minimum phred score of 15, sequence coverage of 10, and a missing data rate of <15% were considered. The resulting 10,966 SNP candidates were filtered to remove variants with more than two alleles and those invariant in the 22 samples but different from the BIMP2.0 reference and those with >500 reads per individual. Remaining SNPs were tested for Hardy-Weinberg deviations using vcftools 0.1.9 [223] and loci with significance <0.01 were removed. This resulted in a final dataset containing 9,607 SNPs in the 22 diploid individuals (average coverage of 151× per individual per site). Linkage disequilibrium (LD) between pairs of SNPs (minor allele frequency >0.05) within the same scaffold was estimated using *r*^2^ on allele counts (geno-r2 in vcftools). BAM alignments were then processed using ANGSD 0.577 to estimate *θ*_*pi*_ = 3*Nμ* from per-site nucleotide diversity across all sequenced sites (1.113 × 10^6^) using a genotype-likelihood based approach that does not rely on SNP calling [224,225]. This method incorporates genotype uncertainty inherent to sequence depth and quality variation from next-generation sequencing, and analyzes all sites as opposed to estimating diversity from SNPs alone. The site frequency spectrum was estimated using SAMTOOLS genotype likelihood estimation, requiring a minimum of 20 individuals sequenced per site, a minimum base quality score of 20, and map quality score of 10. The folded site frequency spectrum was EM optimized for 22 individuals and *θ*_*pi*_ estimated across sites and individuals. RAD sequence data in the form of BAM alignments to AEQM02.fasta have been uploaded to Genbank Sequence Read Archive [NCBI SRA: SRP051027], and SNP data in vcf format are available from the DRYAD digital repository: http://dx.doi.org/10.5061/dryad.52hj2.

### Data availability

The genome assemblies and raw sequence data generated in this study are available at NCBI under the BioProject IDs PRJNA45869 for *B. terrestris* and PRJNA61101 for *B. impatiens*. Illumina RNAseq reads generated during this study include, from *B. terrestris*, queen heads (NCBI SRA: SRX090531), queen ovaries (NCBI SRA: SRX090532) and male heads (NCBI SRA: SRX090533). Illumina RAD sequence of 22 *B. impatiens* samples is available in the Genbank Sequence Read Archive (NCBI SRA: SRP051027). Illumina RNAseq data from *B. terrestris* used for miRNA mining are deposited in the NCBI Gene Expression Omnibus (GSE64512). The mass spectrometry proteomics data have been deposited to the ProteomeXchange Consortium [208] via the PRIDE partner repository with the dataset identifier PXD001623 and 10.6019/PXD001623 for the *B. terrestris* venom proteome and dataset identifier PXD001644 and 10.6019/PXD001644 for the *B. terrestris* queen hemolymph proteome. Principally, comparisons were made with the honeybee *A. mellifera* (NCBI: GCA_000002195.1), but in addition, depending on the purpose of the analysis the following arthropod genomes were also used for comparative analysis. Bees: *A. florea* (NCBI: GCA_000184785.1) and *M. rotundata* (NCBI: GCA_000220905.1). Wasps: *N. vitripennis* (NCBI: GCA_000002325.2), *N. giraulti* (NCBI: GCA_000004775.1), *N. longicornis* (NCBI: GCA_000004795.1). Ants: *A. echinatior* (NCBI: GCA_000204515.1), *A. cephalotes* (NCBI: GCA_000143395.2), *C. floridanus* (NCBI: GCA_000147175.1), *H. saltator* (NCBI: GCA_000147195.1), *L. humile* (NCBI: GCA_000217595.1), *P. barbatus* (NCBI: GCA_000187915.1), *S. invicta* (NCBI: GCA_000188075.1). Flies: *D. melanogaster* (NCBI: GCA_000001215.2), *A. gambiae* (NCBI: GCA_000005575.1), *C. cinquefasciatus* (NCBI: GCA_000209185.1). Moth: *B. mori* (NCBI: GCA_000151625.1). Aphid: *A. pisum* (NCBI: GCA_000142985.2). Beetle: *T. castaneum* (NCBI: GCA_000002335.2). Louse: *P. humanus* (NCBI: GCA_000006295.1). Waterflea: *D. pulex* (NCBI: GCA_000187875.1).

## Additional files

Additional file 1:
**Summary information relating to the** ***B. terrestris*** **genome assembly, details of gene model predictions for** ***B. terrestris*** **and** ***B. impatiens*** **, information on protein domains, bumblebee Juvenile hormone binding protein** **information, biogenic amine receptor information, bumblebee neuropeptide sequences, a comparison of** ***corazonin*** **between the two bumblebees, neuropeptide gene suites across various Arthropods, Halloween gene (p450s) list and phylogenetic tree, annotated lists, and phylogenetic trees of** ***B. terrestris*** **Odorant Receptors, Gustatory Receptors, Ionotrophic Receptors, and Odorant Binding Proteins, a taxonomic distribution of functionally unknown hemolymph associated proteins, and an overview of core RNAi genes with a phylogenetic tree of SID proteins across insects.**
Additional file 2:
**Spreadsheet tabs with genomic coordinates of synteny between** ***B. terrestris*** **and** ***B. impatiens*** **(A), a list of manually annotated genes and species-specific names (B), bee-specific (C) and** ***Bombus-*** **specific (D) genes based on ortholog analysis, protein domains relating to transposable elements (E), venom peptide genes in** ***B. terrestris*** **with proteomic support (F), venom proteins without proteomic evidence, and details of their presence/absence in the genome sequence (G),** ***B. impatiens*** **venom protein predictions (H), an ortholog-based list of bumblebee genes in immune related families (I), proteins identified in** ***B. terrestris*** **queen hemolymph by mass spectrometry (J), and miRNAs sequenced in** ***B. terrestris*** **(K), predicted but not sequenced in** ***B. terrestris*** **(L), and predicted in** ***B. impatiens*** **(M).**
