# Supplementary material for: The genomes of two key bumblebee species with primitive eusocial organization
Source: Genome Biol. 2015 Apr 24;16(1):76. doi: 10.1186/s13059-015-0623-3 (PMC4414376; doi:10.1186/s13059-015-0623-3)
Supplement: Additional file 1: — Summary information relating to the B. terrestris genome assembly, details of gene model predictions for B. terrestris and B. impatiens , information on protein domains, bumblebee Juvenile hormone binding protein information, biogenic amine receptor information, bumblebee neuropeptide sequences, a comparison of corazonin between the two bumblebees, neuropeptide gene suites across various Arthropods, Halloween gene (p450s) list and phylogenetic tree, annotated lists, and phylogenetic trees of B. terrestris Odorant Receptors, Gustatory Receptors, Ionotrophic Receptors, and Odorant Binding Proteins, a taxonomic distribution of functionally unknown hemolymph associated proteins, and an overview of core RNAi genes with a phylogenetic tree of SID proteins across insects. [file 13059_2015_623_MOESM1_ESM.pdf]

Sadd et al. The genomes of two key bumblebee species with primitive eusocial organization

Additional data file 1: supporting data

Contents

Page

1 Supplementary information, statistics and verification of *Bombus terrestris* genome

2-3 Statistics of repetitive elements in the bumblebee genomes

4 Details of gene model predictions for *B. terrestris* and *B. impatiens*

5-7 Protein domain information, including unique, expanded/contracted domains and arrangements

8-10 Bumblebee Juvenile hormone binding proteins, a phylogenetic tree of these proteins, and a map of these proteins in *B. terrestris*

11 Biogenic amine receptors

12-18 Bumblebee neuropeptide sequences, with immature neuropeptide, cleavage site, and signal peptide predictions

19 Differences in corazonin between *B. terrestris* and *B. impatiens*

20 Neuropeptide gene suites across various arthropods

21-22 Halloween gene (p450) table and phylogenetic tree

23-29 Table of *B. terrestris* Odorant Receptors

30-61 *B. terrestris* Odorant Receptor predicted proteins

62-65 Odorant receptor phylogenetic tree

66 Table of *B. terrestris* Gustatory Receptors

67-71 *B. terrestris* Gustatory Receptor predicted proteins

72-73 Gustatory receptor phylogenetic tree

74 Table of *B. terrestris* Ionotrophic Receptors

75-85 *B. terrestris* Ionotrophic Receptor predicted proteins

86-87 Ionotrophic receptor phylogenetic tree

88 Table of *B. terrestris* Odorant Binding Proteins

88-90 *B. terrestris* Odorant Binding Proteins predicted proteins

91 Odorant Binding Proteins phylogenetic tree

92-93 Hypothetical proteins identified within *B. terrestris* queen hemolymph

94 RNAi overview and phylogenetic tree of insect SID proteins

95-97 DNA methylation genes, functional characterization of methylated genes, genome wide distribution of methylation, and genomic methylation levels

98-99 Sample data and analyses of *B. impatiens* SNPs mapped to the genome

Supplementary information, statistics and verification of *B. terrestris* genome

Sequence data for the *B. terrestris* genome

		Roche 454 GS FLX Titanium	
**Mate-pair Distance**	**Fragment**	**8 kb**	**20 kb**
**Number of Reads**	13.8 M	2.5 M	1.4 M
**Pairs**	No	Yes	Yes
**Sequence Coverage[1]**	17.5 x	2.2 x	1.7 x
**SRA Accession**	SRX016989	SRX016990, SRX016992	SRX016991

[1]Sequence coverage calculated as the total trimmed bases divided by estimated genome size of 274 Mb.

***B. terrestris* assembly comparison[1] to cDNA data**

**Minimum Length of cDNA in the alignment**	≧80 %	≧90 %	≧95 %
**Fraction of cDNAs aligned to Scaffolds**	91.1 %	88.4%	84.8 %

[1] Comparison to 42,816 cDNA assembled transcripts aligned to scaffolds of the *B. terrestris* genome assembly. Most (91.1%) of the cDNAs aligned to the genome over at least 80% of their length.

Mapping of contigs and scaffolds onto chromosome linkage groups for *B. terrestris*

Linkage Group	Total Length	Percentage of Genome	Scaffold N50	Number of Scaffolds	Spanned Gaps
**B01**	17,153,651	7%	1,991,050	10	322
**B02**	13,603,873	5%	3,020,712	4	248
**B03**	14,656,165	6%	3,865,109	6	282
**B04**	14,241,696	6%	10,399,280	5	270
**B05**	11,918,102	5%	11,411,890	2	214
**B06**	12,724,418	5%	6,596,734	5	205
**B07**	18,145,390	7%	13,651,897	4	344
**B08**	9,733,834	4%	2,773,881	5	157
**B09**	15,655,298	6%	2,342,655	6	318
**B10**	13,618,662	5%	10,902,106	4	290
**B11**	17,228,712	7%	3,403,609	5	359
**B12**	12,868,931	5%	7,767,148	3	195
**B13**	9,884,808	4%	5,175,709	6	168
**B14**	11,649,563	5%	3,128,055	8	223
**B15**	11,467,329	5%	6,603,705	6	186
**B16**	5,274,633	2%	2,779,439	3	114
**B17**	3,558,169	1%	3,506,793	2	129
**B18**	3,466,108	1%	2,916,968	3	126
**Unplaced**	31,804,902	13%	626,856	5,591	844
**Total**	248,654,244				

Core Protein Set Alignments to the *B. terrestris* genome[1]

**Species**	**Total Core proteins**	**Matched in *B. terrestris***	**Percent Mapping**
A. thaliana	458	438	96%
C. elegans	458	451	98%
D. melanogaster	457	456	100%
H. sapiens	456	449	98%
S. cerevisiae	458	432	94%
S. pombe	458	437	95%

[1]Alignments to the CEGMA core protein sets from named species.

Detailed overview for the repetitive elements in *B. terrestris* (Bter) and *B. impatiens* (Bimp). For each group, the number of elements (putative families), fragments/copies in the genome, cumulative length, proportion of the genome, and features (number containing chimeric or nested inserts of other elements/ appearing complete [all typical structural and coding parts present, even if containing stop codons or frameshifts]/ with RT or Tase domain/ potentially active containing an intact ORF with the all typical domains even though they could lack other structural feature like terminal repeats/ containing an intact ORF for the RT domain or parts of the Tase domain and could be partly active). Uncategorized or those containing features of *Bombus* coding regions are shown at the bottom, and likely do not belong to the repetitive elements.

Type	Number (putative families)		Fragments (full length copies)		Coverage (kb)		% genome		Features	
	Bter	Bimp	Bter	Bimp	Bter	Bimp	Bter	Bimp	Bter	Bimp
**repetitive DNA**	123900	56712	156534 (2005)	210488 (2286)	36228.7	44598.3	14.77	17.90	88/31/106/7/13	190/16/177/6/8
**non-interspersed repeats**	123174	55517	349 (20)	372 (17)	6157.8	3607.4	2.51	1.45	na/na/na/na/na	na/na/na/na/na
SSR	94915 (na)	28931 (na)	()	()	3886.9	1221.9	1.59	0.49	na/na/na/na/na	na/na/na/na/na
low complexity	28249 (na)	26580 (na)	()	()	2048.4	1983.8	0.84	0.80	na/na/na/na/na	na/na/na/na/na
Satellite	10 (0)	6 (3)	349 (20)	372 (17)	222.5	401.7	0.09	0.16	na/na/na/na/na	na/na/na/na/na
**interspersed repeats**	726	1195	156185 (1985)	210116 (2269)	30070.9	40990.8	12.26	16.45	88/31/106/7/13	190/16/177/6/8
**class I - Retrotransposons**	541	948	105281 (1133)	155558 (1365)	20776.1	30391.5	8.47	12.20	40/17/54/2/10	94/4/121/0/5
**LTR Retrotransposon**	150	252	9917 (386)	22348 (514)	3182.8	6614.3	1.30	2.65	16/13/37/2/6	50/4/67/0/2
Copia	22 (2)	27 (7)	1404 (37)	2587 (45)	377.6	744.6	0.15	0.30	3/4/8/1/1	6/0/13/0/0
Gypsy	114 (15)	198 (76)	7704 (331)	16495 (440)	2444.0	4801.0	1.00	1.93	12/7/26/1/4	37/3/50/0/2
Bel-Pao	12 (1)	20 (4)	713 (13)	1384 (22)	330.8	624.2	0.13	0.25	0/2/2/0/1	4/0/3/0/0
Retrovirus, Gypsy-like Errantiviridae	1 (0)	4 (0)	46 (4)	213 (4)	18.0	154.3	0.01	0.06	0/0/0/0/0	1/1/1/0/0
unclassified LTR retrotransposon	1 (0)	3 (2)	50 (1)	1669 (3)	12.4	290.3	0.01	0.12	1/0/1/0/0	2/0/0/0/0
**LINE (non-LTR) Retrotransposon**	69	154	12128 (152)	16342 (245)	3557.9	4378.4	1.45	1.76	12/4/17/0/4	26/0/53/0/3
R2 (NeSL, R2, R4, CRE)	1 (1)	11 (2)	99 (6)	664 (14)	74.5	253.0	0.03	0.10	0/0/1/0/0	2/0/6/0/0
RTE	5 (1)	14 (5)	1823 (11)	3522 (16)	406.5	672.5	0.17	0.27	1/0/1/0/0	4/0/4/0/0
Jockey (Rex,Jockey,Cr1,Kiri,L2,crack,Daphne)	48 (26)	81 (57)	9072 (83)	10021 (111)	2596.8	2589.7	1.06	1.04	11/4/10/0/3	18/0/26/0/0
L1 (Tx1. L1)	0 (2)	0 (0)	0 (0)	0 (0)	0.0	0.0	0.00	0.00	0/0/0/0/0	0/0/0/0/0
I (R1, I, Nimb, outcast, Tad, Loa)	14 (7)	43 (19)	1114 (51)	1912 (97)	474.3	804.0	0.19	0.32	0/0/4/0/1	1/0/17/0/3
unclassified LINE	1 (1)	5 (2)	20 (1)	223 (7)	5.7	59.2	0.00	0.02	0/0/1/0/0	1/0/0/0/0
**SINE, unclassified, putative**	8 (0)	9 (0)	436 (90)	169 (16)	130.6	23.9	0.05	0.01	3/na/0/na/na	0/na/0/na/na
**unclassified Retrotransposon**	2 (0)	13 (0)	500 (3)	2700 (13)	117.5	578.6	0.05	0.23	0/0/0/0/0	1/0/1/0/0
**LARD**	210 (0)	357 (0)	75925 (212)	103869 (360)	11886.4	17118.6	4.85	6.87	9/na/0/na/na	15/na/0/na/na
**TRIM**	102 (0)	163 (0)	6375 (290)	10130 (217)	1901.0	1677.7	0.78	0.67	0/na/0/na/na	2/na/0/na/na

**class II - DNA Transposons**	130	153	26598 (786)	14374 (804)	5408.4	3625.4	2.21	1.45	20/14/34/5/3	22/12/44/6/3
**TIR**	86	108	17516 (417)	11789 (397)	3626.5	2856.0	1.48	1.15	15/14/34/5/3	20/12/44/6/3
Tc1/Mariner	28 (33)	38 (32)	4807 (112)	3479 (112)	1044.4	887.7	0.43	0.36	3/8/19/5/3	3/7/26/4/3
hAT	0 (2)	5 (2)	0 (0)	173 (10)	0.0	39.9	0.00	0.02	0/0/0/0/0	0/0/0/0/0
Mutator (MULE, MuDR)/Rehavkus	0 (0)	0 (2)	0 (0)	0 (0)	0.0	0.0	0.00	0.00	0/0/0/0/0	0/0/0/0/0
P	0 (1)	0 (0)	0 (0)	0 (0)	0.0	0.0	0.00	0.00	0/0/0/0/0	0/0/0/0/0
PiggyBac	43 (14)	52 (30)	10188 (262)	7533 (218)	2036.5	1720.0	0.83	0.69	9/6/15/0/0	13/5/18/2/0
CACTA (EnSpm)	0 (0)	0 (1)	0 (0)	0 (0)	0.0	0.0	0.00	0.00	0/0/0/0/0	0/0/0/0/0
Academ	4 (0)	0 (1)	120 (7)	0 (0)	55.5	0.0	0.02	0.00	0/0/0/0/0	0/0/0/0/0
Kolobok	1 (0)	3 (2)	10 (1)	56 (6)	5.0	20.6	0.00	0.01	0/0/0/0/0	0/0/0/0/0
Sola	0 (0)	1 (0)	0 (0)	10 (2)	0.0	2.5	0.00	0.00	0/0/0/0/0	0/0/0/0/0
unclassified TIR DNA Transposon	10 (5)	9 (1)	2391 (35)	538 (49)	485.2	185.3	0.20	0.07	3/0/0/0/0	4/0/0/0/0
Helitron	0 (1)	0 (0)	0 (0)	0 (0)	0.0	0.0	0.00	0.00	0/0/0/0/0	0/0/0/0/0
unclassified DNA-Transposon	6 (1)	2 (18)	7580 (6)	560 (2)	1282.0	77.6	0.52	0.03	5/na/0/0/0	1/na/0/0/0
**MITE**	38 (0)	43 (0)	1502 (363)	2025 (405)	499.9	691.8	0.20	0.28	0/na/0/na/na	1/na/0/na/na
**unclassified, putative element**	55 (0)	94 (0)	24306 (66)	40184 (100)	3886.4	6974.0	1.58	2.80	28/na/18/na/na	74/na/12/na/na
**other DNA elements**	76	103	8142 (280)	12607 (354)	2008.7	2818.6	0.82	1.13	0/0/0/0/0	0/0/0/0/0
not categorized	24 (0)	30 (0)	1711 (202)	2174 (241)	737.6	874.3	0.30	0.35	0/na/0/na/na	0/na/0/na/na
potential host gene	52 (0)	73 (0)	6431 (78)	10433 (113)	1271.1	1944.3	0.52	0.78	0/na/0/na/na	0/na/0/na/na

Table of gene prediction methods, number of gene models and database locations

Method	*B. terrestris*	*B. impatiens*	Database
**NCBI RefSeq**	10,178	10,226	ftp://ftp.ncbi.nih.gov/genomes/Bombus_terrestris/<br>ftp://ftp.ncbi.nih.gov/genomes/Bombus_impatiens/
**NCBI Gnomon**	13,390	14,758	ftp://ftp.ncbi.nih.gov/genomes/Bombus_terrestris/<br>ftp://ftp.ncbi.nih.gov/genomes/Bombus_impatiens/
**AUGUSTUS**	11262	9635	http://bioinf.uni-greifswald.de/bioinf/katharina/bombus/
**Fgenesh++**	18,340	18,918	
**GeneID**	32,231	32,616	
**SGP2**	28,356	35,368	
**GLEAN**	10,979	15,896	

Protein domains

Overview of species gene sets used in the analysis of protein domains

Species	Version	#Genes with Pfam domains
Bombus terrestris	au6	7,487
Apis mellifera	4.5	8,216
Atta cephalotes	1.2	12,767
Culex cinquefasciatus	1.2	13,198
Drosophila melanogaster	5.48	10,551
Daphnia pulex	FrozenGene Catalog20110204	14,370
Harpegnathos saltator	3.3	10,164
Pediculus humanus	1.2	7,998
Tribolium castaneum	3	10,834

Expansion and contraction of protein domains in *B. terrestris* (Bter) v *A. mellifera* (Amel). P-values are from Fisher's Exact tests. TE-transposable element related.

Domain	Counts		P-value		Function (PFAM)
	Amel	Bter			
PF03022 (down)	22	6	0.00693		Major royal jelly proteins
PF07993 (up)	0	6	0.0117		Male sterility protein
PF02949 (down)	110	13	1.15E-18		7tm Odorant receptor
PF00001 (down)	60	32	0.0156		7tm receptor (rhodopsin family)
PF08395 (down)	7	0	0.0163		7tm Chemosensory receptor
PF14223 (up)	0	6	0.0117	TE	gag-polypeptide of LTR copia-type
PF03372 (up)	7	19	0.0103	TE	Endonuclease/Exonuclease/ phosphatase
PF07530 (up)	0	26	4.23E-09	TE	Associated with zinc fingers
PF00078 (up)	0	84	7.46E-28	TE	Reverse transcriptase
PF13843 (up)	0	13	0.000065	TE	Transposase IS4
PF00665 (up)	0	12	0.000137	TE	Integrase core domain
PF01359 (up)	3	18	0.000593	TE	Transposase (partial DDE domain)
PF07841 (down)	15	1	0.00062	TE	DM4/DM12 family
PF07727 (up)	0	9	0.00127	TE	Reverse transcriptase
PF03564 (up)	0	5	0.0246	TE	Protein of unknown function
PF00651 (up)	12	23	0.041	TE	BTB/POZ domain

Expansion and contraction of protein domains in *B. terrestris* (Bter) and *A. mellifera* (Amel) to *Atta cephalotes* (Acep) and *Harpegnathos saltator* (Hsal). P-values are from Fisher's Exact tests. TE-transposable element related.

Domain	Counts				P-value					Function (PFAM)
	Amel	Acep	Bter	Hsal	Acep-Amel	Acep-Bter	Hsal-Amel	Hsal-Bter		
PF00089 (down)	60	86	59	111	0.00886	0.00637	0.00204	0.00259		Trypsin
PF01826 (up)	13	5	11	4	0.000345	0.00159	0.0256	0.0397		Trypsin inhibitor
PF13912 (up)	29	0	27	10	1.3E-15	7.89E-15	0.000609	0.00138		Zincfinger, C2H2
PF00075 (down)	1	18	1	12	0.0124	0.021	0.00417	0.00458	(TE)	Rnase H
PF07776 (up)	27	27	24	13	0.00298	0.0134	0.0106	0.0306	TE	Zincfinger, C2H2
PF12017 (down)	0	10	0	18	0.0377	0.0387	0.000012	0.000015	TE	Transposase
PF13465 (up)	121	0	107	29	5.62E-63	1E-56	2.4E-17	1.3E-14	TE	Zincfinger, C2H2

Expansion and contraction of protein domain arrangements in *B. terrestris* (Bter) and *A. mellifera* (Amel) to *A. cephalotes* (Acep) and *H. saltator* (Hsal). P-values are from Fisher's Exact tests. TE-transposable element related.

Arrangement	Counts				P-value					Function (PFAM)
	Amel	Acep	Bter	Hsal	Acep-Amel	Acep-Bter	Hsal-Amel	Hsal-Bter		
PF01826 (up)	12	2	9	3	0.000481	0.00316	0.00794	0.0368		Trypsin inhibitor
PF00096, PF13465, PF13894 (up)	18	0	15	3	4.63E-08	3.26E-07	2.06E-04	5.41E-04	TE	Zincfinger, C2H2
PF00075 (down)	0	9	0	11	0.0147	0.0313	0.00156	0.00354	(TE)	Rnase H
PF00096, PF13465 (up)	18	0	14	4	4.63E-08	8.83E-07	7.87E-04	0.00332	TE	Zincfinger, C2H2
PF05699 (down)	0	9	0	22	0.0147	0.0313	2.59E-06	8.01E-06	TE	hAT family dimerisation domain

Maximal count of a repeated domain in an arrangement in *A. mellifera* and *B. terrestris*. P-values are from Fisher's Exact tests.

Arrangement	Domain	Counts		P-value	Function (PFAM)
		Amel	Bter		
PF06582, PF07679	PF07679	7	35	1.42E-05	Immunoglobulin I-set domain
PF06582, PF07679	PF06582	2	14	0.00413	Repeat of unknown function
PF01607	PF01607	3	16	0.00437	Chitin binding Peritrophin-A domain; protease resistance,peritrophic matrix
PF00057, PF00058, PF07645	PF00057	1	11	0.0063	Low-density lipoprotein Receptor domain class A
PF00041, PF00057, PF00058	PF00057	9	2	0.0372	Low-density lipoprotein Receptor domain class A
PF02363	PF02363	58	17	1.02E-06	Cysteine rich repeat; Function unknown
PF01391, PF01607	PF01391	8	1	0.0206	Collagen triple helix repeat
PF00028, PF01049, PF02210, PF12661	PF00028	12	4	0.0473	Cadherin domain

Minimal count of a repeated domain in an arrangement in *A. mellifera* and *B. terrestris*. P-values are from Fisher's Exact tests.

Arrangement	Domain	Counts		P-value	Function (PFAM)
		Amel	Bter		
PF06582, PF07679	PF07679	7	35	1.42E-05	Immunoglobulin I-set domain
PF06582, PF07679	PF06582	2	14	0.00414	Repeat of unknown function
PF00630	PF00630	1	15	0.000511	Filamin/ABP280 repeat
PF00307, PF00630	PF00630	18	3	0.000736	Filamin/ABP280 repeat
PF00053, PF00055	PF00053	1	13	0.00181	Laminin EGF-like
PF00057, PF00058, PF07645, PF12662	PF00057	8	27	0.00183	Low-density lipoprotein Receptor domain class A
PF00057, PF00058, PF07645	PF00057	1	11	0.0063	Low-density lipoprotein Receptor domain class A
PF00041, PF00057, PF00058	PF00057	9	2	0.0363	Low-density lipoprotein Receptor domain class A
PF01391, PF01607	PF01391	8	1	0.02	Collagen triple helix repeat
PF00028, PF01049, PF02210, PF12661	PF00028	12	4	0.0463	Cadherin domain

To/JHBP: Putative genes encoding proteins from the Takeout/ JH binding proteins (To/JHBP) family in bumblebees

List of To/JHBPs in the bumblebees *B. terrestris* and *B. impatiens*.

				JH binding domain			Signal peptide	
Protein ID	Related RefSeq	Length	Genomic location	Start	End	*E*-value	SMART	signalP
Bter_GB19811	XP_003397291	251	LG B08	19	250	2.26E-44	1-18	1-18
Bter_GB17856	XP_003398030	252	LG B09	23	252	2.14E-54	1-17	1-17
Bter_GB13060	XP_003398035	246	LG B09	20	245	7.68E-56	1-19	1-19
Bter_GB10192	XP_003398037	247	LG B09	23	245	1.21E-39	1-21	1-21
Bter_GB20038	XP_003398008	248	LG B09	24	248	4.12E-56	1-20	1-20
Bter_GB16884	XP_003398032	248	LG B09	20	247	4.25E-58	NS	1-20
Bter_GB19662	XP_003398029	265	LG B09	37	264	1.04E-52	1-23	1-23
Bter_GB16869	XP_003398036	251	LG B09	22	251	1.46E-47	1-18	1-18
Bter_GB15461	XP_003398031	247	LG B09	19	247	1.92E-33	NS	1-19
Bter_GB17010	XP_003398007	226	LG B09	4	222	4.04E-32	NS	NS
Bter_GB13060_1	XP_003398034	242	LG B09	18	240	7.61E-28	1-17	1-17
Bimp_GB19811	XP_003488074	251	-	19	250	4.46E-43	1-18	1-18
Bimp_GB17856	XP_003485406	252	-	23	252	9.84E-54	1-19	1-19
Bimp_GB13060	XP_003485403	246	-	20	245	1.03E-56	1-19	1-19
Bimp_GB10192	XP_003485401	247	-	23	245	7.42E-40	1-21	1-21
Bimp_GB20038	XP_003485419	248	-	21	245	4.12E-56	1-17	1-17
Bimp_GB16884	XP_003485404	248	-	20	247	1.61E-58	NS	1-20
Bimp_GB19662	XP_003485423	265	-	37	264	7.34E-53	1-23	1-23
Bimp_GB16869	XP_003485402	251	-	22	251	8.38E-48	1-18	1-18
Bimp_GB15461	XP_003485405	247	-	19	247	8.34E-34	NS	1-19
Bimp_GB17010	XP_003485420	226	-	4	222	7.21E-29	NS	NS
Bimp_GB13060_1	XP_003485421	242	-	18	240	1.67E-24	NS	1-18

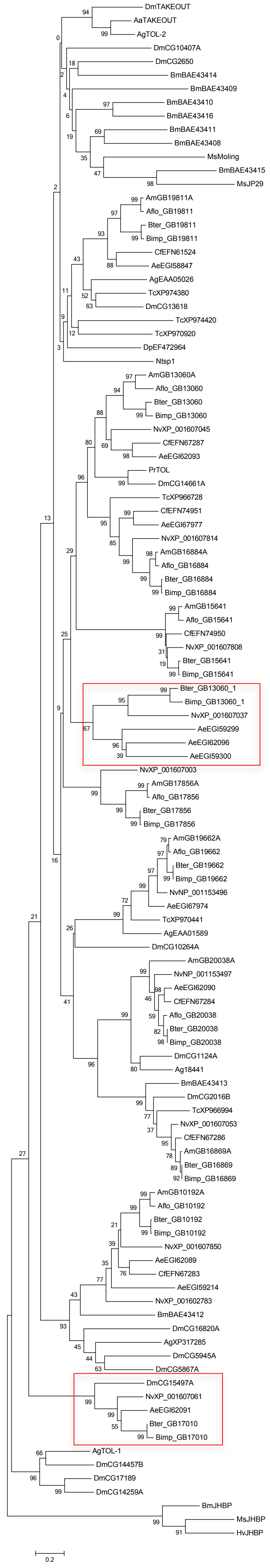

Phylogenetic relationships between To/JHBP proteins in insects. The red boxes show the two JHBPs that are proposed to have been lost in the lineage leading to honeybees. The numbers at the branching points are bootstrap values (%). Branch lengths are proportional to the sequence distance indicated by the bar below the tree. Species abbreviations: Aa—*Aedes aegypti* (yellow fever mosquito, Diptera); Ae— *Acromyrmex echinatior* (Panamanian leafcutter ant, Hymenoptera); Aflo—*Apis florea* (dwarf honey bee, Hymenoptera); Ag—*Anopheles gambiae* (African malaria mosquito, Diptera); Am—*Apis mellifera* (honey bee, Hymenoptera); Bimp—*Bombus impatiens* (bumblebee, Hymenoptera); Bm—*Bombyx mori*, (silk moth, Lepidoptera); Bter— *Bombus terrestris* (bumblebee, Hymenoptera); Cf—*Camponotus floridanus* (Florida carpenter ant, Hymenoptera); Dm—*Drosophila melanogaster* (fruit fly, Diptera); Dp—*Diploptera punctata* (Pacific beetle cockroach, Dictyoptera); Hv—*Heliothis virescens* (tobacco budworm, Lepidoptera); Ms—*Manduca sexta* (tobacco hornworm, Lepidoptera); Nt—*Nasutitermes takasagoensis* (nasute termite; Isoptera); Nv—*Nasonia vitripennis* (parasitoid wasp, Hymenoptera); Pr—*Phormia regina* (black blowfly, Diptera); Tc—*Tribolium castaneum* (red flour beetle, Coleoptera). Accession numbers for proteins indicated by abbreviated name: AaTakeout (AF458100), AgTOL-1 (AAO39755), AgTOL-2 (AAO39756), BmJHBP (AAP41112), DmTakeout (AF261748), HvJHBP (AAA68242), MsJHBP (AAB25736), MsJP29 (AAA21588), MsMoling (AAO65575), Ntsp1 (BAD91203), PrTOL (BAD83405).

The genomic arrangement of putative JHBPs encoding genes in *B. terrestris*

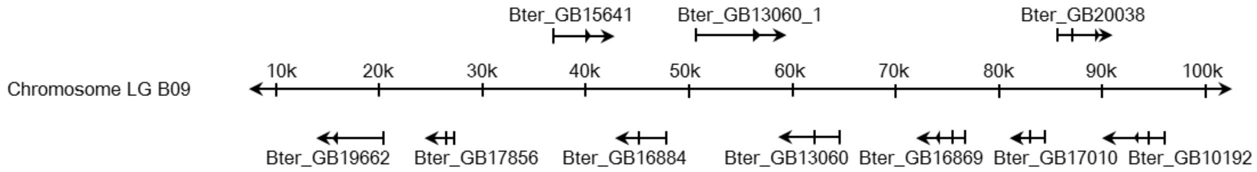

Biogenic amines

An overview of the biogenic amine receptors and their gene identification numbers present in *B. impatiens*, *B. terrestris*, *D. melanogaster*, and *A. mellifera*

Ligand	*B. impatiens*	*B. terrestris*	*D. melanogaster*	*A. mellifera*
octopamine	Bimp11589	XP_003403017.1	CG31351	Am1
octopamine	Bimp11590	on GL898805		Am2
octopamine	Bimp21653	XP_003402376.1	CG6989	Am3
octopamine	Bimp11588	XP_003397143.1	CG6919	Am4
octopamine	Bimp18006	XP_003401247.1	CG3856	Am5
Tyr./Octop.	Bimp13424	XP_003392960.1	CG7485	Am7
Tyramine	Bimp25232	XP_003393336.1	CG7461	Am13
Dopamine	Bimp16334	XP_003401133.1	CG18741	Am6
Dopamine	Bimp21538	XP_003397774.1	CG17004	Am8
Dopamine	Bimp14827	XP_003401071.1	CG9652	Am9
Dopmine/Ecd.	Bimp21034	XP_003395933.1	CG18314	Am19
5HT	Bimp17521	XP_003400301.1	CG15113	Am11
5HT			CG16720	
5HT	Bimp18142	XP_003402761.1	CG12703	Am12
5HT	Bimp12301	XP_003397807.1	CG1056	Am16
Adenosine	Bimp13953	XP_003394193.1	CG9753	Am20
Acetylcholine	Bimp16572	XP_003401764.1	CG4356	Am15
Acetylcholine	Bimp13324	XP_003397629.1	CG7918	Am14
orphans	Bimp19381	XP_003401590.1	CG18208	Am10
orphans			CG16766	
orphans	Bimp21141	XP_003398431.1	CG42796	Am17
orphans	Bimp14525	XP_003393279.1	CG13579	Am18
Total	**20**	**20**	**21**	**20**

Neuropeptides

Bumblebee neuropeptide fasta sequences. Immature neuropeptides are marked with yellow. Green indicates putative cleavage sites for prohormone convertase. Glycine residues that are converted into C-terminal amide groups in the mature peptides are highlighted with blue. Signal peptides predicted by SignalP 4.1 are underlined.

***Bombus terrestris* neuropeptide FASTA sequences**

>**Bter-AKH** (in the C-terminal part of XP_003401166.1; manual curation)
MHSSLYMHRKIRLEFLVFSVILLLCLILNSGAEAQLNFSTGWGKRSQRLEWSAVRPECPSQ
ARPSFEQLLNVYHLIQEARKMLECRKPSE

>**Bter-allatotropin** (XP_003398476.1)
MRASVIIVLAFATGIVVATSRNHNYSHFVKHHARPRVIRGFKPEYMSTAYGFGKRQSTIDV
PKLNKQERILSTLLRYFPQGIPVDWLLQQLKTNPTFATKLTQALMDGRTDFTSMIDRSNPER
ITWLY

>**Bter-Ast-A** (XP_003397004.1)
MKIRTGLLTSSVAFLYLIGIVERPVLAMEEAPPYSMNLHDYNSMLNSMEFDDSVPEKRAYT
YVSEYKRLPVYNFGIGKRWVDTNDNKRGRDYSFGLGKRRQYSFGLGKRNDNPDYPVRLN
MDYLPVDNLAYHSQENLDDLLEAKRSRPYSFGLGKRAAHPNNGQPIGPKRPNDLMSQRY
HFGLGKRMSEDEEDSSQ

>**Bter-Ast-C** (XP_003399378.1)
MSSLRTVIFLAMVLMVLIDLSIALPAADKERLLNEVDLVDDDGSIETALINYLFTKQIVKRL
RNQLDIGDLQRKRSYWKQCAFNAVSCFGK

>**Bter-Ast-CC** (XP_003399426.1)
MGNGVIPLMLVAMVPIIGGTIGQRALSKRNAPTNLDYPDYSTKYDEYPVVVPKRAALLFD
QLMVALQKVVDNQNRGELGGRTLPGPSGPHLPVGNSQKIPSTDEQTMDLQRRGQAKGRV
YWRCYFNAVTCFKRK

>**Bter-bursa** (annotation XP_003402297.1; split into bursa and bursb)
MLLYHIVGASVLICLLSETAEALIGVDECQATPVIHFLQYPGCVPKPIPSYACRGRCSSYLQV
SGSKIWQMERSCMCCQESGEREASVSLFCPRAKPGEKKFRKVITKAPLECMCRPCTSVEEY
AIIPQEIAGFADEGPFTTSAHFRRSSDLQ

>**Bter-bursb** (annotation XP_003402297.1; split into bursa and bursb)
MFIFALFFVLTAFIYMNDTVAQVTDDENCETLQSEVHITKDQYDEIGRLKRTCSGDITVTKC
EGFCSSQVQPSVASTTGFSKECYCCRESYLKERHITLHHCYDADGIKLMNEEDGVMEIKIRE
PAECKCIKCGDISR

>**Bter-capa** (XP_003397181.1)
MRNHLFVFLVVLIFSTSFSQGVKLKLNDRRASGLMAYPRVGRSDVPIPNLNFNRRHAIEPDT
DFQFYSPEFDSVSDKDYEDVRNTAPGSLGRSMNLKHADKFPKEASWLISDRIRSPKDYQLW
QKIDDGRFVYPGSLLLTRGSRNIQLNGYTPRLGRENDDDRTVCK

>**Bter-CCAP** (XP_003401568.1)
MKAINHVCCIFIFVCLVNLTQSENPQDKIEQQLLNAFSTDESLKMKRPFCNAFTGCGRKRNF
HENSMESQGMEANGSIRLPISVYKALLRAATKNIRNTIQRNTNDYQLSGIPQVYLSGRMPL
HKRFDISSTSLN

>**Bter-CCHa1** (XP_003397060.1)
MAIGSENSVAVLIRNWTFMIIFCFAGYAAGSCLEYGHSCWGAHGKRSGGHSNAYLVPLKT
SNEIQQEIPSLTKEQFILSRLIDRPSISNKYKAKWDRLLKLKTSLPESWDGDAFNAQLISDEPS
RDQNNNEDMNLGKRKQTNNMQDITGNTNGERREIPEILLIPNNEDNQHGSKPQNVELFKFL
TDTNGDFE

>**Bter-CCHa2-like** (XP_003397131.1)
MLVDGLTPCLIKSLLLLAKDFNFTSLCRLYGQSCLGGHGKRFDSHIQGNVLQENQVDAATS
DKHQEMEPLRLRNEFVIPGRKFEGQEEMLLPQTRRQDSTRFDPFTLSFIVRQWLTSQHRLH
QPDVELNNN

>**Bter-corazonin-like** (XP_003393262.1)
MDNARVLILFILSLTVMTVTCQSYHYTPDWASGKRSTSMLEEIVNFANKNAGQLDNALVN
CKLQKLRLLLQGNINNQLLQVPCDLLTSGKRSLSENTIIDHFHRQPTPTVNNNY

>**Bter-DH** (XP_003395619.1, XP_003395620.1, 2 isoforms)
MHQRMVSFYILLVVAVLFVSSNLSTNAAPQQGYWEEDPEALMEIISRLGHTMIRNPELENN
KRGLDLGLSRGFSGSQAAKHLMGLAAANYAGGPGRRRRSEQP

>**Bter-DH/CRF** (XP_003403344.1)
MTLMSLLLSLLFIAMTKCQPISYVYDERELSRDNHPLLLLVDHRIPDLENEMFDSGNDPEST
VVRTKRLESKRIGSLSIVNSLDVLRQRVLLELARRKALQDQQQIDANRRILKTIGKRSLPLY
NKDVPKAMDARIRNGIDYVFEQEEKSHDRDVMPERNPDRMQNWLRNDDSAFRERQDDQ
MRRIQANELRLL

>**Bter-EH** (XP_003395267.1)
MVMNASTRILLVLFVSYAIAFMLVAGSVPFDRAIGVCIRNCAQCKKMFGPYFLGQKCADS
CVKYKGKLIPDCEDEGSIQPFLQALDSDY

>**Bter-elevenin** (XP_003402670.1)
MVTVVAYIQRCMIIFAILLCYFHVRSERSESVNCEAFPYHQMCRGSMSRKRAMFPIMYGLG
CEGSEGNINCIKEFEERHRIPYIPLSKSKLLIALLDDDLQKDITRSARHKLRNDEMNKRKPSII
ENFLSELDSSDSY

>**Bter-ETH** (XP_003400029.1)
MTSLRSLGFSRRFIVGALTVGVLALLACENLSKADEVPAFFLKIAKNIPRVGRSEGYNDFLK
SRRNIPQVSGYNSRAQAESWAPYASDKTFSRPIKRRVDYPSIDDAWAWQHFPLAIEGPREL
WRTLAGYSKDTSDDVDNDVWKRKKRTGNEPVISEDN

>**Bter-FMRFa** (XP_003402122.1)
MKRSLTSLYVLSFICNWALIFSSILTPMKADGSLRIFKDGPNDFEYVLKRHEVDRSSEDLDS
KERRSSMGSSFIRYGRSDVDGNIERVSNSDSDGSSKVNRYPRWKSPDIIIRFGRSSFKSLNDD
THYRHGRNNLNFLRYGRNVQVYPLEIDVTAMCSDLLSNDEGNDLHPYEARLLRLCNILNN
SDIEHRNSLDFLEDRLGSKHN

>**Bter-ILP-B** (XP_003400778.1)
MRMYANRLYALMSLILVIVILIPLAEHASDTYKQRNIHGSDVFQYGQKGQSRDQSRDQSRD
QSMTEMHQYCGRILSSTLQIICGSVYNSRFKKSNQEMEMDDYMAYSYDLHPYKSIKNAKK
MIRFRRNGRGIHEECCLKSCTTEELRSYCGAR

>**Bter-ILP-C** (maker-group8.1-pred_gff_fgeneshpp-gene-10.56)
MFGRSRARTIVLVGLVLLTLLDTVNSTPYKRSLLRLCSKSLSDALYLACKDRGYNEPFSYSS
EDSPMDSVGPGLAEECCYHTCTYTQLQQYCKPEKSSSVDAVNSPVWIEKYPYLSTRSATSS
SLEERSRSDIDYVHGTIKCKIHGSKGARRKGANMDRDDDAGGCDRKNPLRRHRAGHCGC
RHRRQRRRRLGKMLERTSGVKSGAPLKKEVETETKATTREAPF

>**Bter-ITP** (maker-Group10.1-pred_gff_sgp2-gene-93.22)
...MHRQQRLRSSSSSLSSLTLPLSMSRSSIPSTTTSISSVTMPSSTTTSTSPSPSPSTPSSPLSSSSP
LSSSSIFLPLSTTWPTSNLSSYSWSVQQTSCSRLPSLPSESSIVSTVSIPCPKLVSVLAWSVTLL
LVSSCIGLTDAGILNGHPLGKRSFIDIQCKGIYDKSIFARLDRICEDCYNLFREPQLHQLCRK
NCFTSDYFKGCLDVLLLQDEVEKIQTWIKTLHGAEPGV

>**Bter-kinin** (XP_003397027.1)
MWLVFPIVSLLIRAILANSEIDDIVLPDIMDLENESNDVLTDNYTDRPGNDIWKVWKKTSFD
PWSGKRAKEYSRSKVDSQGKRTKLTHDWYDRDTPTNEFSNVKRITESVEWTPFNSWGGK
RAGKVNNRDSILALTRDPHNLHLMAEDELIAGRKLGDEKLSIEMKNWLSSMCNTCTKKHC
CSEIEKKTEQNEQYKKPRAWGPWHGKRSLKTYTFRFGNEYFTVPVDSIKAERNFSPFRKHN

>**Bter-myosuppressin** (XP_003398693.1)
MMSSTIVILFSMTTMAISYNNVLAALPTQCNPGFLDDLPPRIRKVCAALSRIYELGSEMESYI
DDKDNHISGFHESIPLLDSGVKRQDVDHVFLRFGRRR

>**Bter-neuroparsin** (XP_003395782.1)
MLAAQTIRIAVLLAIVLLHDKCSGYPSVRQKQEMALCTGCGDSCHKCEYGVVISNACGVP
QCAKGPDEPCGGRGERLGICAEGMSCICNQCFGCSSEKLKCSKIKDPCWRDFNVPVRRPAI
M

>**Bter-NPF** (XP_003400279.1)
MQSCLNSIYLVVVLFILGTGIVHGEPGPMARPTQPDIFTNQEELKKYLDHVTDFYSLNGKA
RYGKRGNVLSSVPEVNYVWDSMKTILENSQRSQQPKANRQFEKRKQEESGFLDEVEKYGP
KKSTSRIDSPPCHVLDTVEKYYDDVQ

>**Bter-NPLP1** (XP_003400507.1)
MGLTGNHFVSLLLYILVVNEIRLPLVTCQDEDNTQCMPKAAFLALLRHPEVSSNLAAYSRA
ARITQDAKSRNDMAHLRALTEEADDTEICVPGRVYLQLLKDPVMRGDLSIILNGRTQKLPD
LLGRLLDDSDEMDAREFLPESQKRSLATLAKNDDLPISIQDRIAENEDDEEKRAAISSEQPG
QNDAGISRDYLLSGGRSDLQAFARDFQMEKRNVGALARDFALPPGRRNIASLVRDYDQSK
NNNRESTLPYNGKRNVASLARTFTLPQNGKRNVASVARDYGLPYGKRYVGSLARTGDFPT
RNQRSVASLAKNSAWPVSLKRGIFLPGSVILRALSRHGRSMVDETNARNDLLDLQELSNLE
QSQRNDYETAEEKLNDSLTKIDSNIRRPKRQIGFSDEYPLPVMQNTNGFDYEEVVEALSGQ
YPNAEKRFMGRIPQMGPRPTTPPTRRQGR

>**Bter-orcokinin** (maker-Group11.1-pred_gff_fgeneshpp-gene-5.4)
MTRHCFLVLSILALSIAGCVLAVQAETSSLRREFYGPSKPEFLVEYLEGHAARKQRDDQDD
PADDRPRRVRPFARDTQARLSSGSTRYLADDYADESSAASKSIALSPIDRQLLERFVKRNID
EIDRTAFDNFFKRNLDEIDRVGWSGFVKRFADDTITGDGRINVLAQRG

>**Bter-PDF** (XP_003399126.1)
MTPVKYVLILLVMLGITYQTLGATEDSYRNLLGFNLPYGRGMNNDLQFARLLMLPPCLCH
SKRNSELINSLLGLPKNMNNAGK

>**Bter-pyrokinin** (XP_003396136.1)
MIGSVAFLSFNRLMTITVCVLLCVVYLVSCASGEYEGRDSSSSMSNDRTPSNEFGSCSDGK
CIKRTSQDITSGMWFGPRLGRRRRADRKPEIDSDIETLANALDGSRWAVITIPGSDRRQATQ
FTPRLGRESGEEYFSYGFPKDQEELYAEEQIFPPLFAPRLGRKVPWTPTPRLGRQLHNIIDKS
RQNFDDARF

>**Bter-RYa** (XP_003400572.1, corrected)
MVMCLWIACMVLTLVASQQKFYIQGRYGKRQEPRTDSFFVSGGRYGRSENTQKASKSLPK
LVEVEVPRIDRFFWGSRYGKLSPPDYRTVSSVNRFSAILNFMDQVNLDRENDESNRNDLDL
LP

>**Bter-SIFa** (XP_003400028.1, corrected)
MMSSRFVVAIVVALFILAIAVDAAYRKPPFNGSIFGKRSNAITDYELTSRAMSSVCETVSET
CNAWLARQDSN

>**Bter-sNPF** (XP_003394766.1, corrected)
MSIKFYAKSLLLFIIVGLVVAAENYIDYGDEVTEKTPIENIREIYKILMQRSALENARLGETPL
EHLMIRKSQRSPSLRLRFGRSDPRLSQFYQNL

>**Bter-tachykin** (XP_003397025.1)
MIIPSTFLLMVSVTFAIAEESMSENVLSDKRAVMGFQGMRGKKNFDSLDSEDFGILKRAIM
GFQGMRGKKNSIIADVENQLFPEETNKRAPMGFQGMRGKKASLDDEYYKRAPMGFQGMR
GKKSLEEILDEIKKTTTRFQDPRSKDMYVIDYPEDYEKRILSMEGYPNIFDKEGEFLWEKRA
PMGFHGMRGKKLILDPWQDLDKQDVMEFQGLQRRKDTFDDYLDYSMNPSDYEKRAADF
QDIESGSDSFKRARMGFHGMRGKRNVEEIYGSNSNPTQTTIGYQGVNNRGNNLAVYEIEK
RSPFRYLGVRGKKNPRWEFRGKFVGVRGKKASSLQSVY

>**Bter-trissin** (XP_003402499.1)
MNCTYVVLFFIVCALWVFGETLSCEQCGRECAEKCGTRQFRACCFNNMKKRQFDLGLKW
RMRPQLYTNWLYPNIDMY

Bombus impatiens **neuropeptide FASTA sequences.**

>**Bimp-AKH** (BIMP14855)
...HRKVRLEFLVFSIILLLCLILNSGAEAQLNFSTGWGKRSQRLEWSAVRPECPSQARPSLEQ
LLNVYHLIQMEARKMLECRKPNE

>**Bimp-allatotropin** (BIMP21229)
MRASVIIVLAFATGIVVATSRNHNYSHFVKHHARPRVIRGFKPEYMSTAYGFGKRQSTIDV
PKLNKQERILSTLLRYFPQGIPVDWLLQQLKTNPTFATKLTQALMDERTDFTSMIDRSNPER
ITWLY

>**Bimp-Ast-A** (BIMP11558)
MKIRTGLLTSSIAFLYLIGIVERPVLAMEEAPPYSTNLHDYNAMLNSMEFDDSVPDKRAYT
YVSEYKRLPVYNFGIGKRWIDTSDNKRGRDYSFGLGKRRQYSFGLGKRNDNSDYPVRLN
MDYLPVDNLAYHSQENLDDLLEAKRSRPYSFGLGKRAAHPNNGQPIGPKRPNDLMSQRY
HFGLGKRLPEDEENSSQ

>**Bimp-Ast-C** (BIMP21837)
MPSLRTVIFLAMVLMVLIDLSIALPAADKERLLNEVDLVDDDGSIETALINYLFTKQIVKRL
RNQLDIGDLQRKRSYWKQCAFNAVSCFGK

>**Bimp-Ast-CC** (BIMP21913)
MGNGVIPLMLVAMVPIIGGTIGQRALSKRNAPTNLDYPDYSTKYDEYPVVVPKRAALLFD
QLMVALQKVVDNQNRGELGGRTLPRSSGPHLPVGNSQNIPATDEQTVKMDLQRRGQAKG
RVYWRCYFNAVTCFKRK

>**Bimp-bursa** (BIMP18662)
MLLYHIVGASVLICLLSETAEALIGVDECQATPVIHFLQYPGCVPKPIPSYACRGRCSSYLQV
SGSKIWQMERSCMCCQESGEREASVSLFCPRAKPGEKKFRKVITKAPLECMCRPCTSVEEY
AIIPQEIAGFADEGPFTTSAHFRRSSDLQ

>**Bimp-bursb** (Bimp18661)
MFIFALFFVLTAFIYMNDTVAQVTDDENCETLQSEVHITKDQYDEIGRLKRTCSGDITVTKC
EGFCSSQVQPSVASTTGFSKECYCCRESYLKERHITLHHCYDADGIKLMNEEDGVMEIKIRE
PAECKCIKCGDISR

>**Bimp-capa** (Bimp23791)
MRNHLFVFLVVLIFSTSFNQGEKLKLNDRRASGLMAYPRIGRSDVPIPNLNFNRRHANEPD
TDFQFYSPEFDSVSDKDYEDVRNTAPGSLGRSMNLKHADKFPKEASWLISDRIRSPKDYQL
WQKIDDGRFVYPGSLLLTQDSRNIQPSGYTPRLGRESDVDRTVCK

>**Bimp-CCAP** (Bimp15632)
MKAINHVCCIFIFVCLVNLTQSENPQDKIEQQLLDAFLTDESLKMKRPFCNAFTGCGRKRNF
HENSIESQGMETNGSIRLPISVYKALLRAATQNTRNTIQRNTNDYQLSGIPQVYLSGRMPLH
KRFDISSTSLN

>**Bimp-CCHa1** (Bimp23716)
MAIGSENSVAVLIRTWTFMIIFCFAGYAAGSCLEYGHSCWGGHGKRSGGHSNAYLVPSKTS
NEIQQEIPSLTKEQFILSRLIDRPSIPNKYKARWDRLLKLKTSFPESWDGDAFNAQLISDGPSR
DQNNNEDMNLGKRKQTNNMQDITGNTNGERREIPEILLISNNEDNQHGSKPQNVELFKFLS
DTNGDFE

>**Bimp-CCHa2-like** (Bimp23727)
MKSNATSGLPLSICVSVCTLVILLSVSETVYAKRGCSAFGHSCFGGHGKRFDSHLQGNVLQ
ENQIDAATSDKHQEMEPLRMRNEFVIPGRKFEGQEEMLLPQTRRQDSTRFDPFTLSFIVRQ
WLTSQHRLHQPDVELNNK

>**Bimp-corazonin-like** (Bimp25646)
MGNARVLILFILSLTVMTVTCQSYHFSIPDWTNEKTSIFKEIVNAINKYISQSNNVLANCELQ
KLKLLLQGNINYQLFQVPCDLLVSGKKSFSENTITDYFHRQPTPVNNNY

>**Bimp-DH** (Bimp18864)
MHQRMVSFYILLVVAVLFVSSNLSTNAAPQQGYWEEDPEALMEIISRLGHTMIRNPELENN
KRGLDLGLSRGFSGSQAAKHLMGLAAANYAGGPGRRRRSEQP

>**Bimp-DH/CRF** (Bimp22606)
MTLMSLLLSLLFVAMTKCQPISYVVDERELSRDNHPLLLLVDHRIPDLENEMFDSGNDPES
TVVRTKRLESKRIGSLSIVNSLDVLRQRVLLELARRKALQDQQQIDANRRILKTIGKRSLPL

YNKDVPKAIDSRIRNGIDYVFEQEEKSHDRDVVPERIPDRMQNWLRNDDSAFRERQDDQM
RRIQANELRLL

>**Bimp-EH** (BIMP17811)
MNASTRILLVLFVSYAIAFMLVAGSVPFDRAIGVCIRNCAQCKKMFGPYFLGQKCADSCV
KYKGKLIPDCEDEGSIQPFLQALDSDY

>**Bimp-elevenin** (Bimp20439)
MVTVVAYIQRCMIIFAILLCYFHVRSERSESVNCEAFPYHQMCRGSMSRKRAMFPIMYGLG
CEGSEGNINCIKEFEERHRIPYIPLSKSKLLIALLDDDLQKDITRSARHKLRNDEMNKRKPSII
ENFLSELDSSDNY

>**Bimp-ETH** (Bimp11504)
MTSLRSLGFSRRFIVGALTVGVLALLACENLSKADEVPAFFLKIAKNIPRVGRSEGYNDFLK
SRRNIPQVSGYNSRAESWAPYASDKTFSRPIKRRVDYPSIDDAWAWQHFPLAIEGPRELWR
TLAGYSKDTSDAVDNDVWKRKKRTGNEPVISEDN

>**Bimp-FMRFa** (Bimp17850)
MKSSLTSLYVLSFICNWALVSSSILTPMKADGSLRIFKDGPNDFEYVLKRHDVDRSSEDPDS
KERRSSMGSSFIRYGRSDLDGNIERVSSSDGDGSSKVNRYPRFKSPDIIIRFGRSGFKNLNDD
THYRHGRNNLNFLRYGRNVQVYPLEIDMTAMCSDLLSNNEINDLHPYEARLLRLCNILNNS
DIEHRNSPDFLEDRLGSKHN

>**Bimp-ILP-B** (Bimp17968)
MRMYANRLYALMSLILVIVILIPVAEHASDTYKQRNIHGSDVFQYGQKGQSRDQSRDQSR
DQSMTEMHQYCGRILSSTLQIICGSVYNSRFKKSNQEMEMDDYMAFSYDLHPYKSIKNAK
KMIRFRRNGRGIHEECCLKSCTTEELRSYCGAR

>**Bimp-ILP-C** (BIMP11552)
MFGRSRARTIVLVGLMLLTLLDTVNSTPYKRSLLRLCSKSLSDALYLACKDRGYNEPFSYS
SEDSPMDSVGPGLAEECCYHTCTYTQLQQYCKPEKSSSVDAV...

>**Bimp-ITP** (BIMP10086)
...WPTSNLSSYSWSVQQTSCSRLPSLPSESSIVSTVSIPCPKLVSVLAWSVTLLLVSSCIGLTD
AGILNGHPLGKRSFIDIQCKGIYDKSIFARLDRICEDCYNLFREPQLHQLCR..

>**Bimp-kinin** (BIMP21628)
MWLMFAIVSLLIRAILANSEIDDIVLPDIMDLENESNDILTDNYTDRPGNDIWKVWKKTSFD
PWSGKRAKEYSRSKVDSQGKRTKLTHDWYDRDTPTNEFSNVKRITESVEWTPFNSWGGK
RAEKLNNRDSVLALTRDPHSLHLMAKGKL

>**Bimp-myosuppressin** (BIMP11018)
...MAISYNNVLAALPTQCNPGFLDDLPPRIRKVCAALSRIYELGSEMESYIDDKDNHISGFHE
SIPLLDSGVKRQDVDHVFLRFGRRR

>**Bimp-neuroparsin** (BIMP12385)
MLAAQTIRIAVLLAIVLLYDKCSGYPSVRQKQEMALCSGCGDFCHKCEYGVVISNACGVP
QCAKGPDEPCGGRGERLGICAEGMSCICNQCFGCSSEKLKCSKIKDPCWRDFNVPVRRPAI
M

>**Bimp-NPF** (BIMP23618)
MQSCSNSIYLFLVLFILGTGIVHGEPGPMARPTRPEIFKNQEELRKYLDHVSDFYSLNGKAR

YGKRGSVLSSVPEVNYVWDSMKTILENSQRSQQPKANRQFEKRKQEESGFLDEVEKYDPK
KSTSRIDSPPCHVLDTVEKYYDDVQ

>**Bimp-NPLP1** (BIMP24350)
...MVTCQDEDNTQCMPKAAFLALLRHPEVSSNLAAYSRTARITQDVKSRNDMAHLRALTE
EADDTEICVPGRVYLQLLKDPVMRGDLSIILNGRTQKLPDLLGRLLDDSDEMDAREFLLES
QKRSLATLAKNDDLPISIQDRIADNEDDEEKRAAISSEQPGQNDAGISRDYLLSGGRSDLQA
FARDFQMEKRNVGALARDFALPPGRRNIASLVRDYDQSKSNNRESTLPYNGKRNVASLAR
TFTLPQNGKRNVASVARDYGLPYGKRYVGSLARTGDFPARNQRSIASLAKNSAWPVSLKR
GIFLPGSVILRALSRHGRSMMDETNARNDLLDLQELSNLEQSQRNDYETAEEKLNDSLTKI
DSNIRRPKRQIDFSDEYPLPVMQNTNGFDYEEMMEALSGQYPNAEKRFM

>**Bimp-orcokinin** (gnl|Bimp_2.0|scf_0361 NT_176780.1)
...IALSPIDRQLLERFVKRNIDEIDRTAFDNFFKRNLDEIDRVGWSGFVKRLADDVVTGDGRT
NVLAQRG

>**Bimp-PDF** (BIMP18458)
...MLGITYQILGTTEDSYRNLLGLNLPYGRGMNNDLQFARLLMLPPCLCHSKRNSELINSLL
GLPKNMNNAGK

>**Bimp-pyrokinin** (BIMP24713)
MIGSVAFLSFNRLMTMAICVLLCVVYLVSCASGEYEGRDSSSSTSNDRAPSNEFGSCSDGK
CIKRTSQDITSGMWFGPRLGRRRRTDRKPELDFDIETLANALDGSRWAIITIPGTDRRQAKQ
FTPRLGRESGEEYFSYGFPNDQEELYAEEQIFPPLFAPRLGRKVPWIPTPRLGRQLHNIIDKP
RQNFDDARF

>**Bimp-RYa** (BIMP19768)
MCLWIACMVLTLVASQQKFYIQGRYGKRQEPRTDSFFVSGGRYGRSENTQKASKSLPKLV
EVEVPRIDRFFWGSRYGKLSPPDYRTVSSVNRFSAILNFMDQVNLDRENDASNRNDLDLVP

>**Bimp-SIFa** (BIMP11503)
MMSFRFVLVIVVVAFILAIAVDAAYRKPPFNGSIFGKRSNAITDYELISRAMSSVCETVSETC
NAWLARQDSN

>**Bimp-sNPF** (BIMP24462)
MSIKFYAKSLFLFIIVGLVVGAENYIDYGDEVAEKTPVENIHELYRILMQRNALENAGLGET
PLEHLMIRKSQRSPSLRLRFGRSDPRSS

>**Bimp-tachykin** (BIMP21639)
MIIPSTFLLMVSVTFAIAEESMSENVLSDKRAVMGFQGMRGKKNFDSLDSEDFGILKRAIM
GFQGMRGKKNSIIADVENQLFPEETNKRAPMGFQGMRGKKASLDDEYYKRAPMGFQGMR
GKKSLEEILDEIKKTTTRFQDPRSKDMYVIDYPEDYEKRILSMEGYPNIFDKEGEFLWEKRA
PMGFHGMRGKKLILDPWQDLDKQDVMSFQGLQRRKDTFDDYLDYSMNPSDYEKRVADF
QDIESGSDSFKRARMGFHGMRGKRNVGEIYGSNSSPTQTTIGYQGVSNPGNNLAVYEIEKR
SPFRYLGVRGKKNPRLEFRGKFVGVRGKKASSLQSVY

>**Bimp-trissin** (BIMP10534)
MNCTYVALFFIVCALWVFGETLSCEQCGRECAEKCGTRQFRACCFNNMKKRQFDLGLKW
RMRPQLYTNWLYPNIDMY

Corazonin

A

>**Bter-corazonin-like** (XP_003393262.1)
MDNARVLILFILSLTVMTVTCQSYHYTPDWASGKRSTSMLEEIVNFANKNAGQLDNAL
VNCKLQKLRLLLQGNINNQLLQVPCDLLTSGKRSLSENTIIDHFHRQPTPTVNNNY

>**Bimp-corazonin-like** (BIMP25646)
MGNARVLILFILSLTVMTVTCQSYHFSIPDWTNEKTSIFKEIVNAINKYISQSNNVLANCE
LQKLKLLLQGNINYQLFQVPCDLLVSGKKSFSENTITDYFHRQPTPVNNNY

B

Bombus impatiens:	pQSYHFSIPDWTNEKTSI...
Bombus terrestris:	pQSYHYT-PDWASamide
Apis mellifera:	pQTFTYS-HGWTNamide
Apis florea:	pQTFTYS-HGWTNamide
Magachile rotundata:	pQTFQYS-HGWTNamide
Pogonomyrmex barbatus:	pQTFQYS-HGWTNamide
Atta cephalotes:	pQTFQYS-RGWTNamide
Acromyrmex echinatior	pQTFQYS-RGWTNamide
Linepithema humile:	pQTFQYS-RGWTNamide
Solenopsis invicta:	pQTFQYS-RGWTNamide
Camponotus floridanus:	pQTFQYS-RGWTNamide
Harpegnathgos saltator:	pQTFQYS-RGWTNamide
Nasonia vitripennis:	pQTFQYS-RGWTNamide
Drosophila melanogaster:	pQTFQYS-RGWTNamide

A comparison of corazonin and corazonin preprohormones between different insect species. (A) A comparison of the corazonin preprohormones in *B. terrestris* and *B. impatiens*. In the *B. terrestris* (Bter) corazonin preprohormone, first the signal peptide (underlined) is removed by a signal peptidase, after which the corazonin sequence (highlighted in yellow) is excised by a prohormone convertase (PC 1/3), cleaving at the C-terminal side of pairs of basic amino acid residues (highlighted in green). The remaining peptide has a C-terminal glycine residue (highlighted in blue) that is converted into an amide group, and an N-terminal glutamine residue that is converted into a pyroglutamate (pQ) group. The *B. impatiens* (Bimp) corazonin preprohormone cannot be processed in this way, because both the pair of basic and the glycine residues are lacking. The preprohormone will, therefore, only be processed into a prohormone lacking the signal peptide, but having an N-terminal pyroglutamate group. (B) Comparison of mature corazonin sequences within the Hymenoptera. Note that the ant corazonins are always identical to the *D. melanogaster* corazonin (bottom), which represent the standard insect corazonin sequence. The five bee species (at the top) have various deviations from the standard sequence (highlighted in grey). These deviations are strongest in the two *Bombus* species.

Neuropeptide barcodes

Present (green) and absent (orange) neuropeptide genes in various arthropod species. Hymenopterans are highlighted in yellow. *A. echinator is* representative of seven ant species and *N. vitripennis* of three *Nasonia* species. *B. terrestris* and *B. impatiens* are identical, and quite similar, but not identical, to *A. mellifera*. We assume these differences may relate to *Bombus*-specific behaviour or physiology. *Dm=D. melanogaster, Bm =Bombyx mori, Tc = T. castaneum, Ae = Acromyrmex echinator, Am = A. mellifera, Bt = B. terrestris, Bi = B. impatiens, Nv = Nasonia vitripennis, Ap = Acyrthosiphon pisum, Dp = Daphnia pulex*

Neuropeptides	Organism									
	Dm	*Bm*	*Tc*	*Ae*	*Am*	*Bt*	*Bi*	*Nv*	*Ap*	*Dp*
ACP	absent	present	present	absent	absent	absent	absent	present	present	absent
ADF-b	absent	absent	present	absent	absent	absent	absent	absent	absent	absent
AKH	present	present	present	present	present	present	present	present	present	present
Allatotropin	absent	present	present	present	present	present	present	present	present	present
AST-A	present	present	absent	present	present	present	present	present	present	present
AST-B	present	present	present	absent	absent	absent	absent	absent	present	present
AST-C	present	present	present	present	present	present	present	present	present	present
AST-CC	present	present	present	present	present	present	present	present	present	present
Busricon-a	present	present	present	present	present	present	present	present	present	present
Bursicon-b	present	present	present	present	present	present	present	present	present	present
Capa	present	present	present	present	present	present	present		present	absent
CCAP	present	present	present	present	present	present	present	present	present	present
CCHamide-1	present	present	present	present	present	present	present	present	present	present
CCHamide-2	present	present	present	present	present	present	present	present	present	absent
Corazonin	present	present	absent	present	present	present	present	present	absent	present
DENamide	absent	absent	absent	absent	absent	absent	absent	absent	absent	present
DH (Calc.-like)	present	present	present	present	present	present	present	present	present	present
DH (CRF-like)	present	present	present	present	present	present	present	present	present	present
EFLamide	absent	absent	absent	absent	absent	absent	absent	absent	absent	present
EH	present	present	present	present	present	present	present	present	present	present
Elevenin	absent	absent	absent	present	present	present	present	absent	absent	present
ETH	present	present	present	present	present	present	present	present	present	present
FMRFa	present	present	present	present	present	present	present	present	present	present
GPA2	present	present	present	absent	absent	absent	absent	absent	present	present
GPB5	present	present	present	absent	absent	absent	absent	absent	present	present
ILP-A	present	absent	present	absent	absent	absent	absent	absent	absent	absent
ILP-B	present	present	present	present	present	present	present	present	present	present
ILP-C	present	present	present	present	present	present	present	present	present	absent
Inotocin	absent	absent	present	present	absent	absent	absent	present	absent	present
ITP	present	present	present	present	present	present	present	present	present	present
Kinin	present	present	absent	absent	present	present	present	absent	present	absent
Myosuppressin	present	present	present	present	present	present	present	present	present	present
Neuroparsin	absent	absent	present	present	present	present	present	present	absent	present
NPF	present	present	present	present	present	present	present	present	present	present
NPLP-1	present	present	present	present	present	present	present	absent	present	absent
Orcokinin	absent	present	present	present	present	present	present	present	present	present
PDF	present	present		present	present	present	present	present	absent	present
Proctolin	present	absent	present	absent	absent	absent	absent	absent	present	present
PTTH	present	present	present	present	absent	absent	absent	present	present	absent
Pyrokinin	present	present	present	present	present	present	present	present	present	absent
RYamide	present	present	present	absent	present	present	present	present	present	present
Sex peptide	present	absent	absent	absent	absent	absent	absent	absent	absent	absent
SIFamide	present	present	present	present	present	present	present	present	present	present
sNPF	present	present	present	present	present	present	present	present	present	present
Sulfakinin	present	present	present	present	present	absent	absent	absent	absent	present
Tachykinin	present	present	present	present	present	present	present	present	present	present
Trissin	present	present	present	present	absent	present	present	absent	absent	absent

Halloween genes

Halloween genes identified in *B. terrestris and B. impatiens*. Genbank IDs of *D. melanogaster*, *A. mellifera* and *T. castaneum* orthologs are also presented. In the case of *T. castaneum* CYP307A1/2 and CYP307B1, contigs were given on which the gene is found, also for *A. mellifera* CYP307B1. For *B. terrestris*, the NCBI gene ID is provided and for *B. impatiens* the official gene set ID is given.

Name		*D. melanogaster*	*A. mellifera*	*T. castaneum*	*B. terrestris*	*B. impatiens*
**CYP307A1**	***spook***	AF484415	-	AAJJ01000951	not present	not present
**CYP307A2**	***spookier***	NM_001110990			not present	not present
**CYP307B1**	***spookiest***	-	AADG05005080	AAJJ01001163	100642619	BIMP19752
**CYP306A1**	***phantom***	AF484413	XM_391946	XM_963384	100642897	BIMP23510
**CYP302A1**	***disembodied***	AF237560	XM_001122832	XM_969159	100649492	BIMP10969
**CYP315A1**	***shadow***	AY079170	XM_395360	XM_965029	100644009	BIMP17298
**CYP314A1**	***shade***	AF484414	DQ244074	XM_967606	100649449	BIMP18931

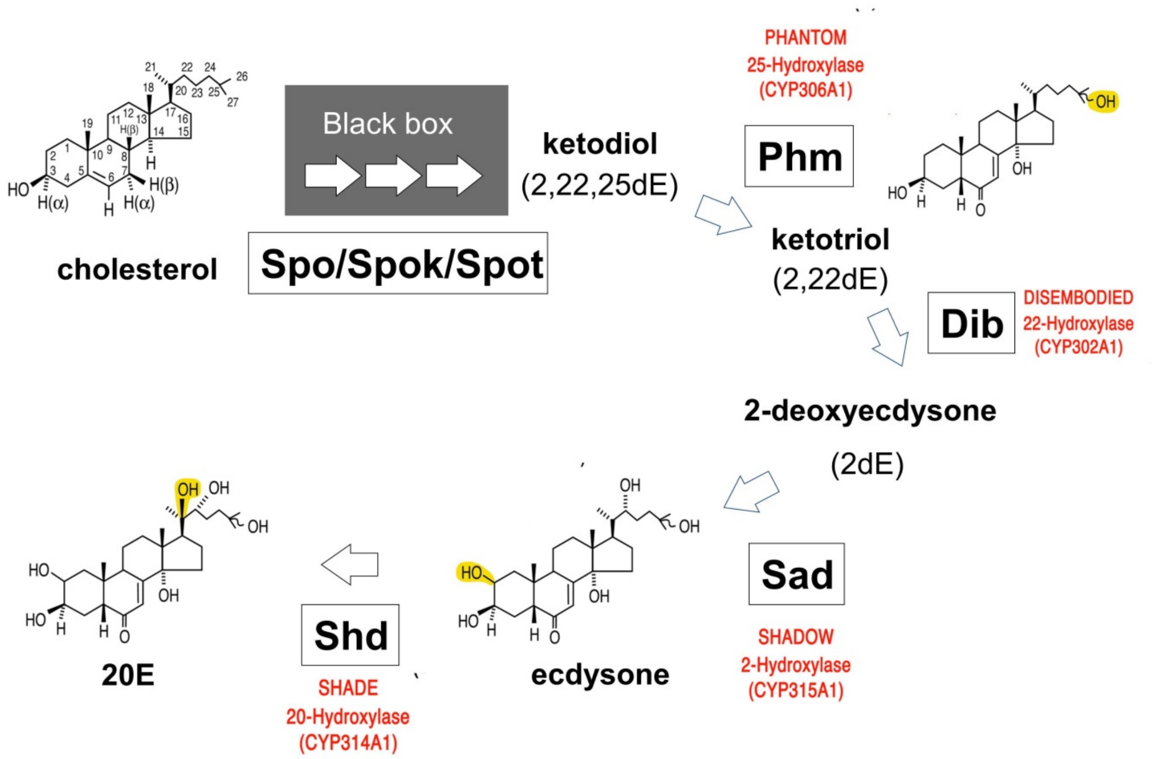

Summary of the biosynthesis of 20-hydroxyecdysone (20E) presenting the different Halloween genes (boxed) and their function in the ecdysteroid biosynthesis pathway. The intermediate products and their structure are also presented. The changes for which the enzyme is responsible are highlighted in yellow.

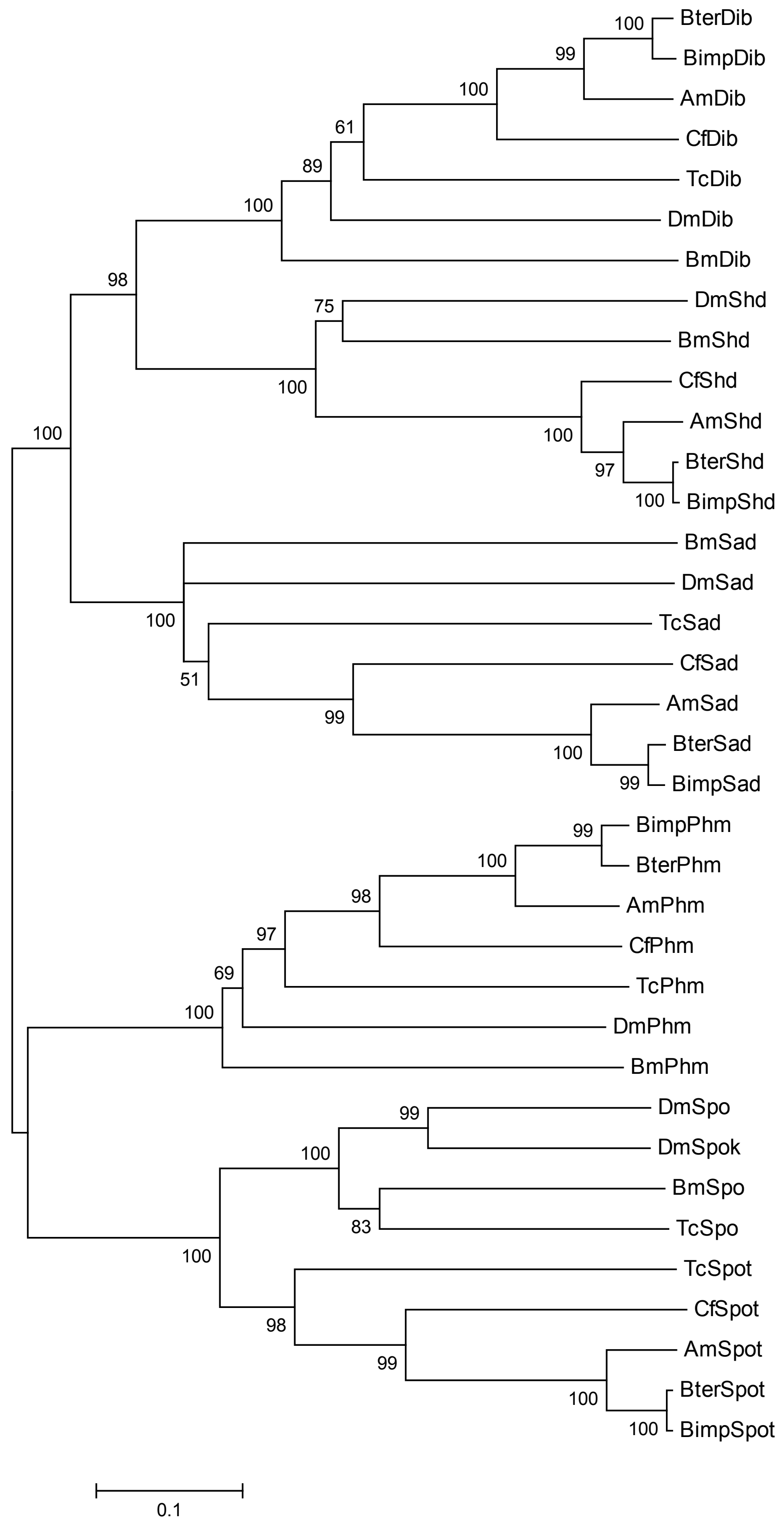

Phylogenetic tree of the Halloween genes. This tree was constructed with the neighbour-joining method using whole sequence amino acid sequences. Bootstrap values as percentage of a 1000 replicates are indicated on the tree. Halloween genes full amino acid sequences are from *B. terrestris* (Bter), *B. impatiens* (Bimp), *D. melanogaster* (Dm), *T. castaneum* (Tc), *A. mellifera* (Am), *C. floridanus* (Cf) and *B. mori* (Bm).

Odorant Receptors

Details of *B. terrestris* OR family genes and proteins. Columns are: Gene – the gene and protein name we are assigning (suffixes are NTE – N-terminus missing; INT – internal exon unidentified; FIX – assembly or frameshift fixed; JOI – joined across scaffolds; PSE – pseudogene; TRA – trans-spliced model; multiple suffixes are abbreviated Fine as is to single letters); Refseq protein number (prefix is XP_00); Scaffold – the genome assembly scaffold ID (format is GroupX.X, amongst 5678 scaffolds in assembly v1.0); Coordinates – the nucleotide range from the first position of the start codon to the last position of the stop codon in the scaffold; Strand – + is forward and - is reverse; Introns – number of introns in the coding region; ESTs – presence of a spliced EST contig in ILLUMINA RNAseq from male (MH) or queen heads (QH), or queen ovary (QO), or multiple life stages (MLS); AAs – number of encoded amino acids in the protein; Comments – comments on the OGS gene model, repairs to the genome assembly, and pseudogene status (numbers in parentheses are the number of obvious pseudogenizing mutations). Suffixes after the gene/protein names are: PSE – pseudogene; NTE – N-terminus missing; INT – internal sequence missing; FIX – assembly repaired; JOI – gene model joined across scaffolds; TRA – trans-spliced gene model; multiple suffixes are abbreviated to single letters.

Gene	Refseq	Scaffold	Coordinates	Strand	Introns	ESTs	AAs	Comments
OrCo	3402775.1	Un633	553161-561881	-	7		479	Fine as is
Or1	3393987.1	2.3	4649752-4652343	+	4		412	Fine as is
Or2	3393988.1	2.3	4653527-4655914	+	4		403	Fine as is
Or3	3393991.1	2.3	4656694-4658840	+	4		404	Change N-terminus
Or4	3393992.1	2.3	4660888-4663020	+	4		404	Fine as is
Or5	3393901.1	2.3	4667219-4669080	+	4		398	Fine as is
Or6	3393993.1	2.3	4670239-4671916	+	4		399	Fine as is
Or7	3393902.1	2.3	4673961-4675822	+	4		407	Fine as is
Or8	3393903.1	2.3	4676857-4678627	+	4		401	Fine as is
Or9	3393994.1	2.3	4680576-4682671	+	4		409	Fine as is
Or10	3393995.1	2.3	4683642-4685608	+	4		411	Fine as is
Or11	3393996.1	2.3	4686553-4688243	+	4		403	Change N-terminus
Or12	3393904.1	2.3	4689169-4691043	+	4		411	Fine as is
Or13	3393997.1	2.3	4691865-4693744	+	4		405	Fine as is
Or14	3393905.1	2.3	4694422-4696139	+	4		405	Fine as is
Or15	3393998.1	2.3	4696844-4699153	+	4		409	Fine as is
Or16	3393906.1	2.3	4699858-4702380	+	4		405	Deletion in model
Or17	3393907.1	2.3	4702914-4704625	+	4		403	Fine as is
Or18	3393999.1	2.3	4706421-4708168	+	4		403	Fine as is

Or19	3393909.1	2.3	4709060-4712056	+	4		405	Deletion in model
Or20	3393910.1	2.3	4713557-4715341	+	4		406	Fine as is
Or21	3394000.1	2.3	4718202-4719901	+	4		398	Fine as is
Or22	3394001.1	2.3	4720605-4722317	+	4		404	Fine as is
Or23	3393912.1	2.3	4723673-4725381	+	4		404	Fine as is
Or24FIX	3394002.1	2.3	4726944-4728666	+	4		406	Fix frameshift
Or25	3394003.1	2.3	4730494-4733168	+	4		409	Fine as is
Or26	3394004.1	2.3	4733882-4735636	+	4		407	Deletion in model
Or27	3393913.1	2.3	4736765-4738491	+	4		405	Fine as is
Or28	3393914.1	2.3	4739300-4741712	+	4		406	Fine as is
Or29	3393915.1	2.3	4742531-4744305	+	4		405	Fine as is
Or30FIX	-	2.3	4747469-4749277	+	4		411	Fix frameshift
Or31	3393917.1	2.3	4750427-4752262	+	4		411	Fine as is
Or32	3394005.1	2.3	4753075-4754956	+	4		407	Fine as is
Or33	3393918.1	2.3	4756102-4758151	+	4		406	Fine as is
Or34	3393919.1	2.3	4758861-4760750	+	4		410	Fine as is
Or35	3393921.1	2.3	4761664-4763201	+	4		411	Fine as is
Or36	3393920.1	2.3	4764251-4766076	+	4		405	Fine as is
Or37FIX	3403429.1	Un5358, Un3730	<1->941, 1051->1758	-	4		406	Fix assembly
Or38FIX	3394006.1	2.3	<4771050-4772860	+	4		406	Fix assembly
Or39	3394007.1	2.3	4773619-4775362	+	4		405	Fine as is
Or40	3394008.1	2.3	4776241-4778655	+	4		411	Fused gene model
Or41	-	2.3	4779534-4781267	+	4		405	New gene model
Or42	3394008.1	2.3	4782152-4784336	+	4		410	Fused gene model
Or43	3394008.1	2.3	4786038-4788418	+	4		410	Fused gene model
Or44	3394008.1	2.3	4789162-4791442	+	4		410	Fused gene model
Or45	3394008.1	2.3	4792303-4794092	+	4		413	Fused gene model
Or46	3394008.1	2.3	4794814-4796922	+	4		410	Fused gene model
Or47	3403027.1	Un975	2257-4556	-	3		394	Fine as is
Or48	3401114.1	15.5	1989652-1991921	+	3		394	Fine as is
Or49	3401175.1	15.5	1994455-1996889	+	3		394	Different final exon

Or50	3401176.1	15.5	2000732-2003508	+	3		400	Fine as is
Or51	3401113.1	15.5	2004311-2006835	+	3		394	Fine as is
Or52	3401177.1	15.5	2008201-2010824	+	3		394	Fine as is
Or53FIX	3401116.1	15.5, Un4668	2015289->201617, <1-6617	+	3		394	Fix assembly
Or54FIX	3403422.1, 3403233.1	Un4605, Un995	<1-1006, <1-645	-, +	3		394	Fix assembly
Or55FIX	3403234.1	Un995, 15.5	<2020697-2021523	+	3		394	Fix assembly
Or56	3401115.1	15.5	2023109-2025428	+	3		394	Fine as is
Or57	3401178.1	15.5	2028000-2030265	+	3		394	Fine as is
Or58	-	13.5	4730302-4733416	+	4		371	New gene model
Or59	-	13.5	4734462-4736834	+	4		370	New gene model
Or60	-	13.5	4737691-4740007	+	4		372	New gene model
Or61	-	13.5	4741197-4743349	+	4		373	New gene model
Or62	3402693.1	Un586	200-2381	-	4		373	N-terminal extension
Or63FIX	-	Un3418, Un3602, 13.5	<1-327, <1->566, <4747131-4748198	-,-,+	4		371	Fix assembly
Or64	-	13.5	4750858-4753038	+	4		373	New gene model
Or65	-	13.5	4754985-4757201	+	4		373	New gene model
Or66	3400291.1	13.5	4758685-4760704	+	4		371	Fine as is
Or67	3400342.1	13.5	4761733-4763633	+	4		364	Part of another model
Or68	3400342.1	13.5	4764721-4767033	+	4		370	Part of another model
Or69	3398625.1	10.4	212240-214970	+	5		403	Fine as is
Or70	3401565.1	15.6	623438-625960	-	5		403	Fine as is
Or71	3401564.1	15.6	619917-622765	-	5		403	Fine as is
Or72	3401563.1	15.6	616976-618984	-	5		427	Fine as is
Or73	3401562.1	15.6	613310-615833	-	5		410	Fine as is
Or74PSE	-	15.6	606932-609414	-	5		402	Pseudogene (5)
Or75FIX	-	15.6, Un124	<603748-606062, <1-635	-,+	5		406	Fix assembly
Or76FIX	3402395.1	Un124, 15.6	1451->3396, 602134->602306	+,-	5		410	Fix assembly

Or77	3401556.1	15.6	598583-601409	-	5		409	Fine as is
Or78	3401567.1	15.6	593001-595284	-	5		403	Fine as is
Or79FIX	3401561.1, 3403388.1	15.6, Un2327	<590785-591896, <1-1428	-,+	5		409	Fix assembly
Or80	3401561.1	15.6	584441-587522	-	5		400	Fine as is
Or81	3401560.1	15.6	579911-582573	-	5		402	Fine as is
Or82	3401521.1	15.6	576848-579134	-	5		407	Fine as is
Or83	3401559.1	15.6	573872-576107	-	5		408	Fine as is
Or84	3401503.1	15.6	570653-572284	-	5		404	Fine as is
Or85	3403340.1	Un1188	450202-452973	-	5		409	Fine as is
Or86	3397911.1	9.5	1069013-1071323	-	5		389	First half of model
Or87	3397911.1	9.5	1061346-1063764	-	5		389	Second half of model
Or88	-	9.5	1051910-1054157	-	5		389	New gene model
Or89PSE	-	9.5	1049777-1050881	-	4		237	Pseudogene (3)
Or90PSE	-	9.5	1045048-1048089	-	5		390	Pseudogene (1)
Or91FP	-	9.5	1041867-1044343	-	5		388	Fix assembly; pseudogene (1)
Or92NTE	3397949.1	9.5	1036933-1039608	-	5		396	N-terminus unidentified
Or93	-	9.5	1033816-1035955	-	5		394	New gene model
Or94	-	9.5	1026671-1028792	-	5		393	New gene model
Or95	-	9.5	1022653-1025011	-	5		389	New gene model
Or96FIX	-	10.1, Un980	8542326-854268, 2139-3497	+, -	5		412	Fix assembly
Or97	3400864.1	14.7	1090557-1092303	-	4		401	Fine as is
Or98	3400880.1	14.7	2355789-2357517	-	4		401	Fine as is
Or99FIX	3400879.1	14.7	2353569-2354931	-	4		402	Fix assembly
Or100	3400841.1	14.7	2348101-2349850	-	4		402	Fine as is
Or101	3402378.1	Un51	23434-37459	+	5		380	Add N-terminus
Or102	-	Un987	30542-41440	+	5		384	New gene model
Or103	3398618.1	10.4	168327-172295	+	5		387	Multiple changes
Or104FIX	3398619.1	10.4, Un4996, Un2069, 10.4	173353->175348, <1->778, <1->655, <177910-178024	+,-,-,+	5		374	Fix assembly

Or105FIX	3398620.1	10.4	181128-184269	+	5		376	Fix assembly
Or106	3398622.1	10.4	186136-189207	+	5		386	Multiple changes
Or107	3398624.1	10.4	198983-206364	+	5		375	Fine as is
Or108	3397556.1	9.1	340763-343912	-	6		408	Add N-terminus
Or109	-	14.4	188105-189918	+	8		394	New gene model
Or110	3401155.1	15.5	862306-868203	+	4		438	Add N-terminus
Or111	3395372.1	5.1	6136242-6139048	-	6		389	Fine as is
Or112	3393433.1	1.8	565127-568007	-	5		390	Fine as is
Or113PSE	-	1.3	2692343-2693558	+	3		262	Pseudogene (6)
Or114	3396270.1	7.1	1526990-1528796	-	4		400	Part of another model
Or115	-	10.4	93829-98365	-	4		398	New gene model
Or116	-	2.1	1006702-1008643	-	5		377	New gene model
Or117	3397790.1	9.4	1150178-1152426	-	5		373	Remove first exon
Or118	3397835.1	9.4	1147545-1149228	-	5		371	Fine as is
Or119	3397834.1	9.4	1144442-1146409	-	5		371	Fine as is
Or120	3397789.1	9.4	1141593-1143119	-	5		380	Add N-terminus
Or121	-	9.4	1136276-1137784	-	5		374	New gene model
Or122	-	2.2	1155133-1158704	+	5		374	New gene model
Or123	-	2.2	1165659-1167791	+	5		374	New gene model
Or124	3395945.1	6.1	2461465-2463691	-	5		375	First half of model
Or125	3395945.1	6.1	2458431-2460596	-	5		377	Second half of model
Or126	3396148.1	6.3	6319634-6321414	-	5		374	Fine as is
Or127	3402379.1	Un51	48634-58988	+	5		374	Fine as is
Or128JOI	3403225.1	Un706, Un987	40915->18304, <1-7812	+	5		372	Join across scaffolds
Or129NTE	3399230.1	11.4	<3933522-3935970	+	9	QO	397	N-terminus unidentified
Or130NTE	3399231.1	11.4	<3939190-3941259	+	9		397	N-terminus unidentified
Or131	3399232.1	11.4	3943207-3945910	+	9	QH	403	Fine as is
Or132FIX	3403290.1	Un1121	1304-4046	+	9	MH	403	Fine as is; Fix assembly
Or133	3399233.1	11.4	3954125-3956822	+	9	MH, QH	403	Fine as is
Or134NTE	3399234.1	11.4	<3958722-3961754	+	9		401	N-terminus unidentified
Or135NTE	3399235.1	11.4	<3962937-3965186	+	9		400	N-terminus unidentified

Or136NTE	3399237.1	11.4	<3966381-3968592	+	9	MH	401	N-terminus unidentified
Or137NTE	3399238.1	11.4	<3969831-3972027	+	9		401	N-terminus unidentified
Or138	3399239.1	11.4	3972875-3976764	+	9		404	Intron splice changes
Or139	3399240.1	11.4	3977675-3980188	+	9	MH	405	First half of model
Or140NTE	3399240.1	11.4	<3982614-3984703	+	9	MH, QH, QO, MLS	403	Second half of model
Or141	3399599.1	12.1	3536724-3542428	-	8	MH	390	Fused model
Or142FIX	-	12.1, Un872	<3532917-3534948, <1-1310	-, +	8		390	Fix assembly
Or143FIX	-	Un872, 12.1	2790->4726, 3528615->3529889	+, -	8		390	Fix assembly
Or144	3399526.1	12.1	3521012-3526168	-	8	MH	390	Multiple changes
Or145	3399598.1	12.1	3516576-3519938	-	8	MH, QH	391	Fine as is
Or146	3399597.1	12.1	3512014-3515217	-	8	QH	389	Fused model
Or147	3399596.1	12.1	3508203-3511225	-	8		390	Needs N-terminus
Or148	3402807.1	Un706	18016-22266	+	8	QH, QO	390	Needs N-terminus
Or149	3403249.1	Un1054	31073-41514	-	8	MH, QH	390	Needs N-terminus
Or150FIX	-	Un1054	21328-29754	-	8	MH, QH	390	Fix frameshift
Or151	-	4.5	9129684-9132673	-	8		388	New gene model
Or152	-	4.5	9121295-9124201	-	8		388	New gene model
Or153	-	4.5	9115463-9118461	-	8		388	New gene model
Or154	-	4.5	9110023-9113027	-	8		388	New gene model
Or155	-	4.5	9102467-9105462	-	8	MH, QO	388	New gene model
Or156	-	4.5	9097265-9100289	-	8	QH	388	New gene model
Or157	-	4.5	9092287-9095717	-	8		396	New gene model
Or158INT	-	4.5	9084314-9087496	-	8		357	Internal exon missing
Or159INT	-	4.5	9080343-9083575	-	8		379	Gap removes exon
Or160	-	4.5	9075988-9078996	-	8		389	New gene model
Or161FIX	-	4.5	9069120-9072462	-	8		401	Fix frameshift
Or162	3394499.1	3.4	1486491-1493341	+	8		396	Needs N-terminus
Or163FT	3392906.1	1.1, 1.1	5810->5968, <1->4854	+, -	8	MH, QH	409	Trans-spliced model, extended assembly

Or164FT	3403003.1	Un2033, Un687, Un800	1219->1833, <2032->2286, <1-1162	+	8		416	Trans-splice model, Fix assembly
Or165TRA	3398760.1	11.1	168721->168864, 165538->167766	+, -	8	MH, QH	407	Trans-spliced model

Protein sequences of predicted *Bombus terrestris* odorant receptors from the table above

>BtORCO
MMKFKQQGLVADLMPNIRLMKATGHFMFNYYTDNSTKTIHRIFAVVHLILMLMQFGFCGINLIFEKEDVDDLTANTITMLFFTHSVVKVVYF
AVRSKLFYRTLGIWNNPNSHPLFAESNSRYHQVAVRKMRILLLAVLVTTMLSAISWTSITFIGDSVKKVIDPITNETTYVEIPRLMLRSWYPYN
ASHGMAHILTLIFQFYWLVFCMADANLLDVLFCSWLLFACEQIQHLKNIMKPLMEFSATLDTVVPNSGDLFKAGSAEQPRDHDPLPPTTPTA
PGENMLDMDLRGIYSNRTDFTATFRPTAGMTFNGSVGPNGLTKKQEMLVRSAIKYWVERHKHIVRLVTAIGDAYGVALLLHMLITTITLTLL
AYQATKINAVDTYAASVIGYLLYSLGQVFMLCIFGNRLIEESSSVMEAAYSCHWYDGSEEAKTFVQIVCQQCQKAMSISGAKFFTVSLDLFAS
VLGAMVTYFMVLVQLK
>BtOr1
MKKVISVDMSEKIGNSTNYRNVHYESDAEYTIHVAKTLLKLIGIWPRRDTFLDNVKMYIQTTIVFSLMCFLLVPHVIYTYFDCENLTKYMKVI
AAQIFSLLAVIKFWTVILNRKEIRFCLDQIEIQYRDVKCEEDRLVMTNCAKIGRFFTMMYLSLSYTGALPYHIILPLISERIVKADNTTQIPLPYL
SNYIFFTVENSPIYEITFVLQIFISSIILSTNCGIYSLIASITMHCCGLFEVTSRRIETLHKWNKCDLHDRVVDIVQYHLEAIGYSALIGKSLSIVFLS
EMVGCTIIICFLEFGVIMELEDHKTLSTLTYFVLMTSIFVNVFIISFIGDRLKQESERIGETSYFLPWYDFPVDVAKNINTIMLRTSLPSCLSGANIL
ELSLQAFCDVCKTSAAYFNFLRAMTV
>BtOr2
MSEKIKNIIYRNEHYKSDSKYVIHIAKTLLTLIGIWPRKDTFLDNVKMYTQTVTIFILMCFLLIPHVIYTYFDCEDLTKYMKVIAAQVFSLLAIIK
FWTLIFNRREIRFCLNEIEIQYKDVECEEDRMIMLNCAKIGRYFTMVYLALGYSGALPYHIILPLISERIVKADNTTQIPLPYLSDYVFFVIEDSPI
YEITFVVQMCISCIIMTTNYGIYSLIASITMHSCCLFEVTNRRIEKLRNWDKRDLHDRIADIVQCHLKAIEYSAVIGKSLSFVFLSEMIGCTIVICF
LEFGVIMEWEDHKTFSTITYFVLMTSMFVNVFIISFIGDRLKQESERIGETSYFLPWYDFPLNEAKNVRTIILRTRLPSSLSGAKILDLSLQAFCD
VVKTSAAYFNVLRAMAA
>BtOr3
MMKIKEAKDKKGIEKDLKQSLRYVEPFVVALGAWPLPPESSLWMKILQRVIRFIIIFLALFFVSPGFYYVLFKEKSNKRKLQIVTTYINSFVQLI
KYIIILCRMKDIRILSEEIRNDWLHSTEENRRLFRENSKIGERVVFIVAFTLYSGGFCYRTILPLTRSSIVLPNNITIRILPSPTYFPFINEQITPYYEII
FVLQVLSGFCIYTVFSGAIGITMMICLHACSLIRILIDKLVDLTDKSNTSEEIIQEKIVNIVGYHRKIKKFLSNGQQLSEYISFLELFNGAGIICLIGY
AVIVEWENLNTITVIVCFTLLTTFTFTSYTICSIGQLLLDESNNLAQTCVTLNWYRLPMKQARYVVLMMIMSNDPIKLTAVKVMDVSLSTFSDI
MKASMGYLNMLRNVT
>BtOr4
MIKTKEESNKEEREKDLKQSLRYVEPLLMVLGAWPLPPESSLCMKIFQRVISVISIFLALFVICPGFFYIFFKAGGTRRKMELLSAFINSLIQLIKY
IIILNSMNDIRTLLREIRNDWLYGTEENRRLFRENAKIGDRVVSIVAITMYCGGLCYRTILPLSRGRITLPNNTTIRLLPSSTYLPFINEQITPYYEII
FVLQVLSGLFIYTVFSGAIGIMMMICLHTCGLIRILTDKLMDLIDKSNTSEEIIQEKIVNIVEYHRKIKKFLSNGQQLSEYISFLELFNGTIIIGLLG
YCVILEWESHNTIGLMVYITLLTTFTFTSYTICSIGQLLLDESNNFARTCVTLEWYRLPVRKTRYMIMIIIMSSDPIKLTAAKMMDVSLLTFSDI
MKGAMGYLNMLRKVN

>BtOr5
MSRLGNKEENLKHFVKFIYPPMKLIGAWPKPTATSTFSKAIKWGLIVSAYFLQLLVFVPGVLYLFLKEKNGKKRIHIMIPHINGFSQLCKYTILL
RRTSEFSKILDQLSDDWVDATEDSRHIFRMRANIGHRMVLTVAITMYTTGLFYRIIIPLSTGRIVLPNNTTMRMLPCPVYFVFFNEQSTPYYEIIF
VLQIMGGFLNYTILCSTMGVCLMLCLHLSSLLRILMNKMIELTSQLDTSETAVQKKISDIVAYQTKVKEFANSVEEITPYLYFFEIFNYAIEACI
VGYCIIVEWEESNAASIIVYLMFQGICIFCNYAMCYIGQLLINESENVRRMSITLNWYRFPMKKARSLILIIIMSNYPIKLTAGKIVDISLATFTDII
KASVGYLNVLRKVT
>BtOr6
MSRIRNAEEELKHSLRLLYTLLRIVGAWPHFVSTSVFSTISKWCLISICYFLQLLLLIPSLIYIFGKETNSRKRLKLLVPHINGLVQLTKYTFLLYR
MHDFKELMETMKNDWLNATEENRKIFRENTEIGYKMLLGLAILMYSTSFCNRIIVPLWKGRIILPDNTTIRLLSYQSHFTFFDVHRTPNHEIIFT
LQILGAFVIYTILCGSLGLISLLCVHMSSLLRILANMMIELSDQPDTSENAVYKMIKDIVEYRTKVKKFSDKVEHIMSYISCLEIFNGTCIACLVG
YCIIMEWENSDTVAVIIYIMFQTICMFIVFTMCYIGQLLINESENVRQISVTLNWHRFSSKEVRCLIPVMIISNYRIKITAANIIEISLATFTDIMKA
SIGYLNVLRNSVK
>BtOr7
MSKSSAMKNTSQLTKREEDMKYATRYVKPILGAIGAWPVSFSSSFMSKILLRTEHILTYFLFFLIIVPTIMYVFFKEKNNKIRLKLMGPIINCTM
QFCKYTILLWRTNEVQKGLDVMKQDWITATDENRLIFRSKAKIARRVVLTAAITMYGGGLCYRTILPLLKGPIITPDNITIRPLPCPSYFVILDE
QKSPNYEILFLVQILAGFVIYAVISGSCGLSALFVLHACSMLRILVDKMKALVDMRDMSDTMVQRRIMDIVEYQTKIKRFLKNIETITEYICLT
EMMGGTCLVCLVGYYILMEWENNNIAAVLIYVTLQISCTFCVFILCYIGQLLIDENQIVGQASCMINWYHLSTKHMRSLILIIAMSNYPMKLM
AGKMIEMSLATFTGVMKLSMGYLNILREVI
>BtOr8
MENISALAKAEEDMKYATRFVKPILGIVGAWPVSSSASSFSKILTRIEHILTYFLFLLLMVPTLMHVFLKETNNKIRLKLMGPIINCSMQFCAYT
ILLCRASEIQNGLNVIKQDWVTATEENRLIFRSKAKIARRVVLTVAITMYGGGLCYRTVLPLLKGTIVTPGNITIRPLPCPSYFVILDEQKSPNYE
ILFVLQVMAGFVIYAVISGSCGLSALFVLHVCSMLSILVDKMKALVDMRDMSDTMVQRRIMDIVEHQTKIKGFLKNIETITQYVCLSEMICGT
SLVCLVGYYIVMEWENNNATAVLIYSTIQISCTFSVFILCYIGQLLVDENRIVGQASYMINWYYLSRKHMRSLILIIAMSNYPMKLMAGKMIE
MSLATFTDVMKMSMGYLNILRNVI
>BtOr9
MKRGNIVETSNNASYKSDLRFNVRLNVLTLRTIGTWPRFLDDSWWETIEHVFLNLLCYGLLGFILIPGFMCLALEIVGFYNQMRLGSALSFFL
MAVMKYCVFIMREDDIRRCVKLIEDDWRNVKHNEDRKIMLKNASFSQRLIIICSVFMYGGVVFYYIALPLTRAKIVEEGGNLTYMRLVYPFP
RTIVDARRSPINEIFYTIQLLSGFVAHDITVAACGLAALLAMHACGQLQVLMSWLEKLVDGRENDDESLDQRLANIVEQHVRIINFIALTEDLL
REISLVEVVGCTMNICFLGYHSMMEWDFNHPVTGFTYIILLISVTFNIFIFCYIGEVLAEQTVKVGQKSYMIDWHRMPWKKSLVVPLMISMSR
STTKITAGNIIELSISSFGNVIKTSFAYLNMLRTFAI

>BtOr10
MQTSDIKNTKFRITDYEYKRNVNLSIQWNLWLLKSIGLWPYSNSISRIRRYFYWFINIACYSLISFLFIPCVLYIVLDIEDTYGKLKQFGPLIFCA
MAFAKYYSLIVHKADIRECLERIKWDWKNMTNREDREIMTVNASFGRKLVVVCTLFMYSGFVFYYIAIPISVGKIAAENESLTFIPLVFPFSRFI
VDTRYSPTNEIVFSIQLVAGCLMHGITSAACSLAAAFAVHACGQMQVLMNWLKHLVDGRSDMSERVDGRIADIVCQHVRILKFLTLIENTIQ
QISFAEFLGCTLDICLVGYYVIMELKSNDVTSALTYMILLISITFNIFIFCYIGEIVTEECRKIGETSYMIEWYRLQGNKKLCCVLIIAMSNCTIKLT
AGNIVNLTINTFADVVKTAVTFLNVLRKTT
>BtOr11
MSSTKVDQDYKKGVNLSIRSSRWILKLIGVWPNSRNVSSVKKYFHVLLNAIYYALIMFLLLPGSLYVILEVEDVYDRIKLFGPLSFCVMALLK
YYLLILHEEDIRECVERIEWDWQNITYPKDRELMMTNANFGRKLVIACTFFMYSGFIFFYIAVPMSVERIPVEGTNATFIPMVFPFSRFIIDTRYS
PTNEIVFSIQFLAGALMHGITSAACSLAAIFAVHACGQMQVLMTWLNHLIDGRLDMHDCVDQRIAKIVSQHVRILKFLSLIERALQQVSYVEF
LGCTLDICLLGYYIIMEWNSNHLTDVMTYSVLLVSLTFNIFIFCYIGELVADKSRKVGEMTYMIEWYRLYGKKKLCCVLIIAMSDSSRKLTAG
NIVELSMSTFSDVVKTSVTFLNVLRTLT
>BtOr12
MQKSVTNDASYLTTDHDCKRNVDLSIRYSRWILKPIGVWPTSPNILMAEKYFYRFINTVCYSLICFLLVPCSLYLILEVKDVYNIIKLFGPLSFC
VMAFLKYYLLILHEDDIRECIKRIEWDWKNITYSEDREIMITNANFGRRLVIICAFFMYSGFAFYYIAIPISVGKISAGDVNVTFIPLVFPFSRFIM
DTRYSPINEIVFSLQLVSGCLMHSITSAACSLAAIFAVHACGQMQVLTSWLKHLIDGRSDMYNNVDSRIAIVVSQHVRILNFLALTEKTLQQVS
FVEFIGCMLNICLLGYYVIMEWSSSHLTSAITFFILLISLTFNIFIFCYIGEVVAEQCKKIGETSYMVDWYRISGTRKLCFVLIIAMANSSIKLTAG
NMVVLCLTTFSDIVKTAVAFLNVLRTLT
>BtOr13
MNKKKPVMTEHDYKRNVDLSIQWNRWLLVPIGTWPNLRKSILGKYFSSLINVICFGLIGFMLVSCTLYLVIEVEEAYHKIEMIGPLSFFLMTF
MKYYLLILQENHISEGCERIEWDWKNIEHHEDRNIMAQNANYGRRLVAICTFFLFSGFAFFYVVVPASVGQVVTEDGNLSFVPLPFPASRLIA
DVRSSPSNEIIFSIQTLTGVVMHTVTSAACSIAAVFAVHACGQMQVLMNWLEHLVDGRSDMSNIVDERIASIVTQHNRILRFLALTESALQQIS
FVEFLGCMMNMCLLGYYFIVEWSSNDIMHSITHMILMISFSFNIFIFCYIGELVAEQCKKVGEMTYMIDWYRLTGKKKLGCILIIAMSNSSIKFT
AGNMIELSISTFSDVVKTAVAFLNMLRALM
>BtOr14
MQKQKSVVTEHDYEKDVGSSIQWNRWLLIPIGAWPNFRKSMIGKYFSSLINILCFGLIAFMFVSCGLYALIEIEELYHKIELLGPLIFFLMTFIKY
YLLILHENDIREGCKRIEWDWKNIEHQEDRNIMTVNANYGRRLAAICTFFLFSAFAFHYIIVPISMGKVVDESGNLSFASLPFPVPGFIADVRYS
PCNEIFYFIQTLTGAVMHTVTSAACSIAAVFAVHACGQMKVMMNWLEYLVDGRPDMSNTVNGRIAKIVIQHNRILKFLALTENALQQISFVE
FLGCMLIMCLLGYYLIMEWNSKDIMLSITHMALMTSFTFNIFIFCYIGELVAEQCKKVGEMTYMIDWYRLTGKKKLGCILIIAMSNSSIKFTAG
NMIELSISTFSDVVKTAMAFLNMLRALT

>BtOr15
MLDLTSEPRISNVIYARDFEYSIQVNRWLMQPIGAWPKLAKTTRTQRLFVRLLNFICHSLIIFMIVPCILYIAYEAESSKTRMKAIGPVSHWLMG
ELNYCCLLSRINDIVRCIKHVERDWQMVENESDRELMLKNAKVGRFIALVAALCMHSGVLAYTVTNGFKKMVFHLGNDSYSMYPLPYPFY
TNLLDVRFSPANEIVLTLQLLSGFVVTSVTVGACGLAAVLTMHASGQLNVVMARLDNLVNTKEEEKQEEQKAAQRKLGIIVEHHLRTLSLM
ASIEKVMNMICLVELVGCTMNMCMIKYYLLTEKSKDVIIAYAILYASMVFNIFIFCYIGEIITEQGERVGKKVYMTEWYRLPPKTALGLVLVIS
RSSMVVKITAGKFVQISIATFGAVFKTSFAYLNMIRTMM
>BtOr16
MNESMIIRTDAKSNSDYSLQLNRWFLKPIGAWPYFSTTSTLEKVISVSLIIFCYIVILFSIIPCVAHLIFEDDSFYRKVRVFGPLGHWFIGGINYTN
LLFRSKNISDCVEHIETDWQIVTKEKQQQVMLKHAKFGRYVSAICAIFVHSGIMSYCIVSASSTQIIKVGNETRTMRSLPLGVYNRMIPVDTSP
ANEIVLVMQFLSAFIADSSGIGFYTLASVLAAHACGQLSVLTICISDYVNEAGNRKEDTSFRKIGTIVEHHLRTLDFIARIEEVMSWVCMTELFR
CVLAICMVGYYIVTEWSDHDVRSLTSYFIIFASVTFNTFLLCYIGELLTEQCMKVGEIVYMTNWYILPRKRILELILIIARSSVVIEITAGKLIHMS
IHTFGDVMRLAFAYLNILCQMT
>BtOr17
MTNQSVTIGANAKANSDYGLQLNRWFLKPIGVWPSSPSTTRFEKIISITLNVICYTSIILTTIPCLVRMFLEDESIYLKVKSLGPVSHWIVSGANY
TTLLLRSNDIRQCMEHMEADWQTVTREKDQWVMLKNAKFGRYVAASCAIFMQGGIMCFIFVTAMDTVEIQIGNETRILHVLPCAVYKKLIN
VDESPTNEIVLFLQAWSTIIANSSTVGIFSLAAVLAAHACGQLNVVMVWITEFVNEPKKANGPKGIGVIVERHLRTLNFIGYIENLMNRIYFLEI
FRCTMDICILGYYILSEWDDQNVQSLVTYVMIYISIGFNVFLICYIGEILTEQSKKVGEVVYMTNWYYLPDKTILDLILIIARSSVVVQITAGKM
VLMSVYTFGDVVKTGFAYLNLLRQIT
>BtOr18
MTNQPVTIGANAKTNSDYGLQLNRWFLKPIGVWPSSPSTTRFEKIVSITLNVICYTSILLTTIPCLLRMFLEDESIYLKLKSLGPVSHWVVSSAN
YTTLLLRSKDIRQCMEHMEADWQTVTREKDQWVMLKNAKFGRYVAASCAIFMQSGVMCFIFVTAMDTVEIQIGNETRILHVLPCAVYKKLI
NVDESPTNEIVLFLQAWCTIIANSSTVGMFSLAAVLAAHACGQLNVVMVWITEFVNEPKKADGPKGIGVIVERHLRTLNFIGYIEKLMNRVYF
LEIFRCTMNICILGYYILSEWADQNVQSLITYIMIYISIGFNVFLICYIGEILTEQSKKVGEVVYMTNWYYLPDKAILDLILIIARSGVVVQITAGK
MVLMSVYTFGDVVKTGFAYLNLLRQIT
>BtOr19
MSESAIIKANAKSHSDYSLQINRWILKPIGAWPYFSTTSTRERVISLFLIVLCYVVILFNLIPCVAYLIFVGDSFYRKVKVFGPLTHSFLGGVNYT
NLLFRSRNISDCVERIETDWRMVKKEDEQQVMLKHAKFGRYVSTMCAIFVHSGIMSYCIASASNVQIIKVGNETRTIRTLPFDVYNKMIPVDT
SPANEIVLVVQFLSAFIADSSGIAFYTLASVLAAHACGQLGVLTIWINDYVNEARNRKQDTSFRKIETIVKHHLRILDFIARIEEIMSWVCMTEL
FRCVLAICMVGYYIVMEWSDHDVRSLTSYFVICASLTFNTFVLCYIGEVLIEQCMKVGEIVYMTNWYFLPRKRILELILIIARSSVVIEITAGKLI
HMSIHTFADVMRLAFAYLNILCQMT

>BtOr20
MTNEPVITEGDSNSNSDYSLQLNRWFLKPIGAWPSSPSTTRLEKIVSFILNIICFTSVIITAIPSLLLMILEDESIYMKLKTLGPVSHWFVSSANYT
ALLMRGRDIRHCVEHIEADWRTVTREEDQHVMMKNAKFGRYVAASCAVFMQGGVLCFCFVTALTTTEIQIGNETRIIHVLPCAVYKKLVNV
DESPANEIMLFLQIWAALIANSSTVGIFSLAAVLAAHACGQLDVIMMWITKFVKEAKQRKKTSSFREIGVIVERHLRTLNFISCIEDVMNRIYFL
EMFRCTMDICVIGYYILSEWADHDIQNLSTYFMMLISICFNIFVICYIGEILTEQCQKVGEVVYMTNWYYLPDKIILDLILIIARSSVVVHITAGK
LVHMSVYTFGDVVKTGFAYLNLLRQMT
>BtOr21
MSNEAVIIEADSNSGYCLQLNRWFLKPIGAWPSSPSTTRLEKIVSFILNIICFASVIVTAIPSVLLLILEDESINLKLKTLDFLSHLFVSSFNYSTLLL
HSKDIRQCVEHIEADWRSVTRVEDQHAMMKNAKFGRYVAASCAIFMQGGILCFCFVTALTTVEIQIGNETRVLRLLPCAVYKKLVNVDESPA
NEIMLSLQIWSALIANSSTVGIFSLAAVLAAHACGQLDVIMAWITEFVKETSSFQEIGVIVERHLRTLNFISCIEVVMNKIYLLEMLRCTMNICLI
AYYILSEWDEHDIRNLTSYFMMFVSICFNIFVICYIGEILTEQSKKIGDAVYLTNWYYLPNKEIHNLILIIVRSSMVVQLTAGKMIQMTINTFGN
VVKTGFAYLNLLQQMM
>BtOr22
MPNEAAVIEADSNSNSDYSLQLNRWFLKPIGAWPSSPSTTKLEKILSFLLNIICFGTVTVTVIPSLLLIILEDESINFKLKALGFVSHWIVSSLNYT
ALLLHSKDIRQCIEHMETDWRTLIREEDQHVMLKNAKFGRYVAAFCAIFMQGSVLCFCFVTALNTFEIQIGNETRVLHVLPCAVYKKLVNVD
ESSRNQIMLFLQVLSAIIANSSTIGIFSLAAVLAAHACGQLNVVMLWINEFVNEARGEKTSSIQIGAIVERHLRTLNFISYIENVMNKICLLEMLR
CTMDICVTGYYILSEWTEHDIQNLSSYFMMLVTICYNIFVICYIGEILSEQCKKIGEVVYMTNWYYLPGKTILDLIMVIARSNVVVQITAGKLV
HMSVYTFGSVLKTGFAYLNLLQQMI
>BtOr23
MSNEAVIIEADRNSDYCLQLNRWFLKPIGAWPSSPSTTRLEKIISFLLNTICYSTVIITTIPSVLQLILEDESINLKLKSIDFVSHLIVSSFTYSVLLL
HNKDIRRCVEHMKTDWRAVTRKEDQQVMMKNAKFGRYVAAFCAIFVQGSVLCFCFVTALNTLEVQIGNETRILHVLPCAVYKKLVNVDES
PTNEFMIFLQIWSTFIANCSTVGIFSLAAVLAAHACGQLNVVMLWIVEFVNEAKVERRADGFMEIGIIVERHLRTLNFISYIEGVMNKICFLEM
LRCTMDICVIGYFIVSEWAEHDVRNLTSYSMMFVAICYNIFILCYIGELLTEQCKKIGEVVYMTNWYYLPGKTILDLIMVIARSNVVVQITAGK
LVHMSVYTFGSVVKTGFAYLNLLQQMT
>BtOr24FIX
MTTEVVIIEGDSKRNSDYSLQLNRWFLKPIGAWPSSPTTTRLEKIVSFTLNIVCFSTLILTAIPSLLVIILEDQTFNFKLKTFGFVSHWFVSSFNYA
VLLMHNKDIRKCVSYIEADWQTVTREEDQHVMLKNAKIGRYIAAFTAIFVQSSVLCFCFVTALNTVEIRIANETRILHVLPCAVYKKLVNVDE
IPTNELMLFFQIWSTVIANFSTIGIFSLAAVLTAHACGQLNVITLWIVEFVNETRVEKKTGGFIQIGVIVERHLRTLNFISYIEDVMNKICLLEML
RCTMDICVIGYYILSEWDEHDIRNLASYFMMFVTICFNIFIICYIGELLTEQCKKIGEVVYMTNWYYLPSKTILDLIMVIARSNVVIHITAGKLV
HMSVYTFGSVIKTGFAYLNLLQQMM

>BtOr25
MVDMSSEPPISNAFYAHDYEYSIQVNRWLMQPIGAWPKLTKTNRTQRLLTKLLNFICHSLIIFTIVPCILYIVYEAESSKTRMKIIGPVSHWLMG
ELNYCCLLSKTDDIIRCIKHVERDWQVVENASSREMMLKYAKVGRFIAFIAAFCMHSGVLAFNVTKGFKKMMFLVGNDSYFMYPLPCPIYT
NLLDARFSPANEIVFVLQILSGLIVTSVTVGACGLAAVLTMHASGQLNMVVARLDNLVDTKIEEKQEAQTVAQKKLGIIVEHHLRTLSLIASIE
KVMNMICLVELVGCTINMCMIKYYFLTEKSKDMRIVYAIVYASMVFNIFIFCYIGEIVIEQGERVGKKVYMTEWYRLPHKTALGLVLVISRSS
MVVKITAGKFVQISITTFGIVFKTSFAYLNMIRTML
>BtOr26
MMNETVIVESDSKSNSDYSLQLNRWFLQSIGAWPSSPSTTRLEKIVSFILNIVCLSIVILTVIPSLLLMILGDESFNFKLKMFGYVSHWIFSGINYT
ALLTQGKNIRQCIEHIEADWRTVTREEDKNVMLKNAKLGRYIAGFSVIFVQGSVLCFFFVTALNTVEIQIGNETRVLHLLPCAVYKKLVNVDD
SLTNEIMLLLQMWSTIIANSSTSGIFGLSAVLAAHSCGQLNVIMVWITEFVNKARERKKTGSFIEIGMIVERHLRTLNFISCIEDVMFKIYFVEMF
RSTMDICVIGYYILSEWADHDFQNLITYFMMLISICFDIFVICYISEILTEQCQKVGEVVYMTNWYYLPNKVILDLILIIARSSIVVQLTAGKFIQ
MSVYTFGNVLKTGFAYLNMLQQMTR
>BtOr27
MTNKTVAIKIDFDGRSDYSLQLNRWFLKPIGAWPSFPSTSRLERIISFVLIVICYIILICTIIPCLLHIILENESFRIKLRLIGPASYLCVGSINYTTLLL
RGKDIRCCVEHMQADWRTVKREKDQQVMLKSAKFGRYVTASTAAFMQGGVFCYCFMTALSTEVIQVGNETRIVHQLPYVTYKELIDINESP
TNEIILFMQFLTGFIVSSSTLGILSITVVLIAHACGQLNVVMTWITEFVNESRKEKIAPFENIGIIVERHLRTLSFVSSIEETVNRIFFLEVLRSTLHM
CMLSYYIVTEWSDSDIQILTTYSMLLASICFNIFVICYIGETLTEQSRKVGDVVYMANWYYLTEKRILELILIIMRSSVVVEITAGKIIHMSIQTFS
TVIKTAFTYLNLLRQVT
>BtOr28
MTNKSAVVEKTFDSLSDYSLQFNRWLLISIGAWPASTSTSRRERIISFILIALCYGFILFTVIPCIFHFILEDESIYMKLKVLGPLSHWFVGGINYT
NLLLRSNDIRDCIHHVQTDWKIVTRPEDSQVMMKYARIGRFVAAFCATFMQGGVLTYCVVTAFATQTIEIANETRIIHMLPCAAYKNLIPVDT
SPMNEIVLATQFISGFIVNSSAVAAITIGAVFTAHAYGQFTVLMRWINEFVNRSEDQKKDVEFNEIGEIVEHHLRILSLIAGIENVLTQFCFMELL
KSKLDISMLGYYIITEWAEHDIRNLTTYFMIMASMSFNIFTVCYIGDILTEQSKKFGDVVYMTKWYYLPNKDMFDLILIISRSNSAIKITAGKIT
HMSINTFGDVMKTAFAYLNLLRQVT
>BtOr29
MTESVRIEKNLYSLSDYSLQLNRWFLTPIGVWPLSASTSRLERIISFVLIFLCYFFVLFTIIPCLLHLILEDENTRTKLKIIGPFSHWLIGGINYSTLL
LRSREMRVCIEHVQNDWKIVTRQKDQQVMMRYARIGRYTTVFCATFMQVGVLSHCTMAAFATKIIEIGNETKIIRLLPCGFYKSLISIDVSPTY
EIILASQFVSGFIANSSAVGAVGMAAVFAAHAYGQLTILMIWIKEFVNRSKDYSRNVGLNEIGEIVEHHLRVLSFIAGIEDVMNEICFMELFKC
TMNMCMIGYYILMEWRDHDIQYMPAYLIILFSMTFNIFVICYIGETLKEQCEKVGEVAYMTNWYYLPYKDILNLIQIILRSSMMIKITAGKLVH
MSIFTFGNVIKTACTYFNLLRQVT

>BtOr30FIX
MVVLLNPSTHSWDKDWMYSIQINRWLLKSIGVWPISLCITPTEKSNSIILTLISSFLISFLLVPCALCTLLVKTDDPEAKIKMVGVLSFCVMAAI
KYYILASRGVMIGKCIENIRSDWDRIHAQLRQEDREIMKENAKIGRSLAIFCAGFMYSGGFFYTTVMPLCTERTEIIDNETVRTQAFPIYRGLLD
PRTSPSFEIVQFMQCLAGFVIYSVTVGACSLAAVFVMHVCGQFGILVKKLQRLVGSLEEEKNLNVHEQRLGDIVEHHLHILGFISQIEDLLNEIC
FVELIGCTVNICFLAYHLLTEWEQNDTIGTLTYCTLLISFTFNIFILCYIGEILSEQCRNISISAYMIDWYRLPQKKALGLILIFAVANSSTKLTAGK
IVELSLASFCSVLKSAFAYLSLLRTLTI
>BtOr31
MVVLLNPSTHSWDKDWMYSIQINRWLLKSIGVWPISLCITPTEKINSIILTLISSFLISFLLVPCALCTLLDKTGDLDTKIKMIGPLSFCVMAAIK
YYILVSRGAKIGKCIENIRSDWDRIHAQLRQEDREIMKENARIGRSLAIFCAGFMYSGGFFYTTVMPLCTERTEIIDNETVRSQAFPIYRGLLDP
RTSPSFEIVQLMQCLAGFVIYSVTVGACSLAAVFVMHVCGQFGILVKKLQRLVGSLEEEKNLNVHEQRLGDIVEHHLHILGFISQIEDLLNEIC
FVELIGCTMNICFLGYYLLTEWEQSETIGTLTYCTLLISFTFNIFILCYIGEILSEQCRNISLSAYMIDWYRLPQKKALGLILIFAVANTSTKLTAG
KLVELSLASFCSVLKSAFAYLSLLRTLTT
>BtOr32
MTDDPVTVERKFGSLSEYSIQVNRWILKPIGAWPSSNSTTNREKFISWILNVICWCFSLFTIIPGVLNILLEKEDLYLKLKMFGPLSHWCVGGFN
YAVLLLRQGDIHYCIKHIRTDWKTITRLEDQRVMLKNAKLGRYIACFCAAFMHGSVFCTCLVLGAFKRTIEVRNETILVYTLPCPAYKLPVQT
NPGHDIILGTQFLSAFIATSSAAGAFSLATVFASHALGQLNIMVTWINEFVNQHSKDQNNNARANKIGVIVEHHLRALSLIARIERIMSPICFME
MFKCMLGMCMPSYYILVEWSEHNIQNITAYVLIIISMTCNIFLVCYIGEIIMEKCKKIGDMVYMADWYHLPDQDIINLIMIISRSSMEVKITAGK
IIDMSVLTFANIVKTVFGYLNMLCQTTMT
>BtOr33
MADELVAAKKKYGNLGEYSIQLSRWYLQPMGAWPNSPSTTRREKILAQISIVICWCIVLFTIVPGFLHIILVKEDIYLKLKTLGPLSHWCVDGF
NYAVLLLRQEDINYCIERVRSDWKMITRVQDRHEMWKNAKLGRYVAGFCAGFMQGTMIYTCIVLGAFRRTIKVGNETMDIYTLPCPAYKFA
VQTNPTHDIILGTQFLSALVVSSGAAGSFSLATVFASHALGQLNIMVTWVDEFTNQTKQQNKQAQINKIGVIVEHHLRVLSLIARIERIMSPICF
MEMFKCMLGMCMPCYYILAEWSEHNVQAMIIYVMVFLSMTFNIFLICYIGEVLKEQCQKVGDMVYMTNWYQLPDKNILSIIMIISRSHMEV
KITAGKIITMSVYTFGNIIKTVFTYFNMLRQTTMI
>BtOr34
MRSSNDVNDHPQNNHYKSDIYHTFQFCHWILKPLGIYFFLHDHVNKFEKIVSVLLIIICCSILQFVIVPFGYYILFYEKDMNMKIKFLGPLAFCV
TALFKYGYLGVKSSELGRCVKHVKKNWKMLQDKDHRAIMVRYVTMGRNLITLCAAFMYTGGLSYHTIMPLLSKKKINENITIRPLTYPGYE
AFFDIQESPTYEIVYCMHCVYVLVTGNITMAAYSLTAIFTTHACGQIKIQTLRLENLKKDGKALENGIEDHLAVIVSEHVEILRFTKNIEIALQG
LFLIEVMLSTLLICLLEYYCMMEWETSDSAAISTYIILLTSFTFNILIYCYVGELLLGQGSEIATALYDIGWYNLPGRKARDIVLVLAISKYPLKL
TAGKILVLSMNTFGVVLKSSLVYLNMLRTVTEF

>BtOr35
MHDRSHATVDDQLRNVHYENDIHYTLQMCQWLLKPIGVWPLVNNHTSRLEQLVSVILMIMCFSSLLFIILPSFYHIFFVEKSVHVKVKLLGPV
GFCLSSTIKYCYLGVKGAFFERCIQHVEKDWKMVEDPNHRTIMLKYATVSRKLITLCAIFLYTGGMSYHTVMQFLSKERNKKNYTFRPLTYP
GYDSFLDTQSSPTYEVVFFLQCFAAMIMYSVTTVAYSLAAIFVTHICGQIQVQIARLQDLVENEKRKNNGHDSMSVIVHDHVEVLRFSKNVEE
ALREICLAEIVESTIIMCLLEYYCMMEWQNSDAIAILTYVTLLISFTFNIFIFCYIGEILTEQCSQIGTTSYEIEWYQLPAKRAYNLILLISISQYPPK
LTAGKIIDLSLNTFSSVAKSSLIYLNLLRTVTDW
>BtOr36
MYDRSYTIVDDQLKNNHYKNDIHYTLQMCQWLLKLIGIWPLVNNHTSRLEQLLSIVLMITCYSSIFFIILPSGHHFFFVEKNLYMKMKMLGPV
SFCVFATVKYSYLARKGTFLQRCIRQVKNDWKRVQDPYHRQIMLKYAGVSRKLITMCAVFIYTGGMSYHTVAQFLSKDNTKENSTVKPLAY
IGYDPFFDTQSSPTYEIVFFLHCFAAMIMYSITTVAYGLAAVFVTHVCGQIQIQIARLQNLVGSKDRDLFTVIVHDHAETLRFSKNIEDALYQIC
LTEIVECTICMCILEYYCLMEWANSDLIATLTYLTLLTSFTFNIFIFCYIGELLTEQCSQIGTTSYEIEWYQLPAKRAYDLILLISISQYPPKLTAGK
IIELSLNTFSSVAKTSLVYLNLLQTVTD
>BtOr37FIX
MYDRSYTIVDDQLKNNHYQNDIHYTLQMCQWLLKLIGMWPLVNNHTSRLEHLLSIIVMIMCFCSIFFIILPCGHHFFFVEKNIYMKMKMLGP
VSFCVFATVKYSYLALKGAFLQRCIRQLKNDWKRVQDPSHRAIMLKYAGISRKLITMCAVFIYTGGMSYHTVAQFLSKERTRENYTVRPLAY
IGYDPFFDAQISPTYEIVFFLHCFAAMIMYSITTVAYGLAAVFVTHVCGQIQIQIVRLQNLVESKDRDLFAVIVRDHVKILRFSKNIEDALYQICL
TEIVECTINMCMLEYYCLVEWANSDLIATLTYMTLLTSFTFNIFIFCYIGELLSEQCSEIGTISYEIDWYNLPAKEAYDLILLISISQYPPKLTAGKI
IELSLNTFSSVAKTSLVYLNLLQTVADW
>BtOr38FIX
MYDRSYTIVDDQLKNNHYQNDIHYTLQMCQWLLKLIGVWPLVNDHTSKLEQLLSVVVMIICFCSIFFIILPSGHHFFFVEKNLYMKVKMLGP
VGFCVFATVKYSYLALKGAFLQRCIRQLKNDWKRVQDPSHRQIMLKYAGISRKLITMCAVFIYTGGMSYHTVAQFLSTDKTRENYTVRPLT
YIGYDPFFNTQSSPTYEIVFFLHCFAAMIMYSITTVAYGLAAVFVTHVCGQIQIQIVRLQNLVESKDRDLFAVIVRDHVETLRFSKNIEDALYQI
CLTEIVECTMNMCMLEYYCLMEWASTDLIVTFTYITLLTSFTFNIFIFCYIGELLSEQCSEIGTVSYEIDWYNLPAKEAYDLILLISISQYPPKLTA
GKIIELSLNTFSSVAKTSLVYLNLLQTVADW
>BtOr39
MHLSVRQPQNPNYEEDIVYVTKHNKWILSTIGMWPTVVKGIGKFVPKIIIGLSNFVSSLNVLQFILHIILEEKNTTLKVRFLGLICFASTNLMKY
WALIARKSNIEYCIEQVQIDWKQVDFQRNRMLMLKYGKIGRDLTIYSAVFMYGSEMLYITIMQYALGSMLKENNRTTRVLVYPTYSGLLDV
QKSPIYEIVYVLQCMCTLLFNSVTVSCCGLAALFATHACGQIDIIMSQLDDLVDGKFAKKNSNPDTRLTEIVKHHIKILKFSTMIETVLQEVCFF
EFVGTTLVVCFLEYYCLTDWQSNNKIGVATYSMLLVSLTFNMFLLCYIGNLLLEKSSDIGISCYLIDWYRLPPKTVQDLMLIIAMSNTPVKISA
GRIFLLSLPTFGNILKTSFAYLNFVRNATM

>BtOr40
MNMHLSVRYQTDQPLNPKYEEDIIYVTKHNKWVLNSIGMWPAVLEGIGKFVPKIVIGLSNFVSFFSVVQCVLHIILEEKDPLLRLRLLGLACY
SSTNLMKYWALILRKPNIEYCIEQIQTDWKQVEFPRNRLLMLKYGKIGRDLTICSAAFMYTGLVCYVTIMQYAMGLNMKANNRTIRVLVYP
TYSGFFDAQKSPIYEIVYVLQCMCTFVFNSVTVGCCALAALFATHACGQLDVVISQLNDLADGKFSGKNSNPSTRLIEIVEHHIKILKFSAMIES
VLQEVCFFEFVGSTFVICLLEYYCITDWQQNNKIGLATYSMLLVSLTFNMFLLCYIGNLLLEKSTNIGISCYMIDWYRLPVKTVQDLMLIIAMS
NSPVKISGGRMFLLSLPTFGNILKTSFAYLNFIRNTLM
>BtOr41
MHLSVRQPRNPNYEEDIVYVTKHNKWVLNSIGMWPAMLKGIGKFVPKIVIGLSNLVSVFNVLQFVLYVILEEKDTSLKLRFLGLICFASTNLM
KYWALIARRPNIEYCIKQVQTDWKQVEFQRNRTLMLKYGKIGRDLTIYSAVFMYSSEMCYITIMQYAMASVLRENNRTTRVLVYPTFSGFFD
AQKSPVYEIVYVLQCMCTLLFNSVTVACCGLAALFATHACGQIDVVISQLNDLVDGKFANKNSKPDTRLIEIVENHIRILKFSTMIETVLQEAC
FFEFVGSTLVICLLEYYCITEWQDNNKIGVATFSMLLVSLTFNMFLLCYIGNLLIEKSTNIGISCYLIDWYRLPPKTVQDLMLIIAMSNNPVKISA
GRIFLLSLPTFGNILKTSFAYLNFVRNATL
>BtOr42
MHLSVRHQTDPPQNTNYEEDIVYVTKHNKWVLNSIGMWPAVLKGIGKFVPKVVIGLSNIVSFLNVVQCVLYITLEENDPLLRLRLLGLACFA
SINLMKYWALIARKPNIEYCIEQVHTDWKQVEIQRNRILMLKYGKMGRDLTIYSAVFMYSAEICYVTVMQYAMGLNMKENNRTIRLLVYPT
YSGFFDAQKSPVYEIVYVLQCMCTFLFNSVTVGCCGLAALFATHACGQIDIVISQLDDLVEGKFSEKNSNPNTRLMEIVKHHIRILKFSAMIET
VLQEVCFFEFVGSTFVICLLEYYCITDWQQNDRIGLATYSMLLVSLTFNMFLLCYIGNLLIDKSTSVGISCYMIDWYRLPIKTVQDLILIITMSNS
PAKISAARIFILSLPTFGNVLKTSFAYLNFIRNTIIY
>BtOr43
MHLPVRRQTVPPQNTNYEEDIIYVTKHNKWVLSSIGMWPAVVEGIDKFLPKIVIGFSNLVSLFTVVQCILHIILEEKDALLRLRLLGLACFASIN
LLKYWAVIVRKPNIEYCIKQVQTDWKQVKFQKNRMLMLRYGKIGRDLTIYSAMFMYSAGMCYITIMQYAMAMSLKANNRTIRVLVYPTYS
GFFDAQKSPIYEIVYVLQCMCTFVFNSVTVGCCGLAALFATHACGQIDVVISQLNDLVEGKFAKKNSDPNTRLMEIVKHHIRILKFSAVIETVL
QEVCFFEFVGSTFVICLLEYYCITDWQQNNKIGLATYSMLLVSLTFNMFLLCYIGNLLIEKSTSVGISCYMIDWYRLPVKTVQDLILIIAMSNSP
AKISAGRIFLLSLPTFGNVLKTSFAYLNFVRNTIMY
>BtOr44
MHLSVRRQTDQLQNPNYKEDIIYVTKHNKWILNCIGIWPTVLKGIGKFLPKVVIGFSNLVPFFTIVQCVLYITLEEKNPLLRLRFCSLAWYSSIN
LMKYWALIARKSDIEYCIKWVQTDWKQVKFQKNRMLMLKYGKIGRDLTIYSAVIMYSAGMCYTTIMQYAMRMSLKENNRTIRILVYPTYS
GLFDTQRSPVYEIVYVFQCVYAFMCLSVTVGCCGLAALFATHACGQIDVVISQLDDLVDGTFSKKSSNPNTRLMEIVKHHIRILKFSAMIETV
LQEVCFFDFIGTTLLICSLQYLCITDLQYNNKIGLATYSMLLIGFTVNMALLCYIGNLLMDKSTSVGISCYMIEWYRLPGKTMQDLILIIAMSNS
PAKISAGRIFLLSLPAFGNILKTSFAYLNFVRNTIVI

>BtOr45
MFTSTHLSTRSQTDQLRNPNYKEDIIYVTKHNKWVLNSIGIWPAMVEGIGKFLPKIAIGLSNLILFFTLVQCVLHIVLEQKDPLLRLKILGLTCFS
FISLMKYWALTIRKPKIEYCIEQLYADWKQIEYQRDRKLMLKYGKIGRRLTVYSAVFMYSGGIIYHTAMQYAIGSYVDEFNRTIKLLVYPTYS
GLYDVQKSPVYELVYILQCMCGYVFDTVTVGACGLAALFATHTCGQIDVIMSRLNDLIDGKFSKENSNTSVRLMEIVEHHIRTLKFSAMVET
VLQEVCFLEFIGTTFVMCLLEYYCITDWQQNNKIGLTTYSLLLISLTFNMFLLCYIGDLLIEKSTNVGISCCMIDWYRLPAKSVQDLVLIIAMSS
NPAKISAGRIVNLSLSTFASVLKTSFAYLNFLRTALV
>BtOr46
MHLSMKHKCDYTPRNLYYKKDIAYVTKHSKWILQSIGIWPAVVGDVKKFLTKISIALSNFVLLFAIIPCILHIIFEEKDTIMRLKLSGLLSFCCTS
LMKYWALTVRKPRIKGCIEQVWIDWEQVELHKDREIMLKYGRVGRNLTIICAVFMYTGGTIYHSILQYAIGTFVDEHNRTIKPLVYPTYSALY
DVQSSPIYDLVYVIHCMCGYVMYSITAGACGLAALFATHTCGQIDIVISRLNDLVRGEYMKETLNLNARLIEIVERHLRILRFSAAVEMVLQE
VCFLEFIGSTCMICLLEYYCITDWEQSNTISLTTYTMLLISLTFNIFILCYIGELLIEKSSSVGTSCFMIDWFHLPTKTIQGLILVIAMSNNPAKISA
GKIADLSLSTFGSVLKSSLAYLSFLRTAVM
>BtOr47
MKVPSDKDFTYAMTPLKMLSWPVGTWPLQEYDIFSGIRAIIAISFLLLMLMVMQMELYLDISDAKKNLDALILINCSILSLSKIIRFRIQPDSLIL
NFVSAVKDYNELKDQEKRVIMRRHAYMGRLVSASMIFSSYIGSTLYMTIPMMAGDKEKDIVNVTKESTTDYPIPSECVIALIQLPDNLYFMVF
IIEYLMLLFTSTGNLGSDTLFLGIIFHLCGQVEILKLEFSKLGNENERTMERFVVLIKRHVYLLNLAKMLNETISTILAVQLFSSCVLICITGFQLI
LDLSVGNIVMTIKEFIILNAMLVQLFAYSYVGEYLKLQMEGVGDSLYFCSWYDIPTSITKDIIYVIMRSQDPVFLKAGRFFIVNMETYMSIVKT
SMSYLSVLRVMVNA
>BtOr48
MKESSDEDFAYAMTPLKIMSWPVGTWPLQVYDIFSAIRAIIAILLLLFMLIIVQMELYLDRSDAKKNLDALLLITCGILALSKIIRFRIQPDGLVS
NFISVIKDYNELKDQEKRVIMRRHAYMGRLVSASVIFSSCIGSTLYLTIPMLSGDEEKDIVNVTKESSTDYPMPSEYVIALMQLPDNLFFMVFII
EYLMLLFTSTGNLGSDTLFFGIIFHLCGQVEILRLEFNRLGNKNERTMERFIVLIKRHIYLLNLAHMLNETISSILVMQLFSSCVLICTTGFQLILA
LSIGNIVMMMKGFIVLNAMLVQLFAYSYVGDYLTRQMKGISDSMYFCNWYDISKSMAKDIIYVIMRAQYPVSLKAGNFFIVNMETYMSILK
TSMSYLSVLRVMVNA
>BtOr49
MKESSRKDFAYAMTPMKIVSWPVGTWPLQNYGIFSAMRGIIVISLVLLMLAILQTEMYLDSGDAGKNVDALILITSGVVALSKIIWFRIRPAGL
ISNFTSAVKDYEELEGQDKRLIVRRHAYIGRVASATVIFSSYIGSTLYMTIPMLAGDEEKDIANVTEESTTDYPIPSEYVMAVIQLPDNLYFMVF
IIEYLMLLFTSTGNLGSDSLLFGIIFHLCGQVEILKLEFNKLGNENERIMERFIVLIKRHVYLLNLAKMLNETISSVLVVQLFTSCILICTTGFQFIL
DLSVGNIVMAMKTFIVMSCLLVQLFAYSYVGEYLKRQMESVGDSVYFCNWYHIPKNVAKGIVFVIMKSQDPVSLKAGKFFIVNMETYMSIL
KTSMSYLSVLRVMVNP

>BtOr50
MREKVAIEEYVRNFLVQETVLKVVGIWPTRNESFFGRWIFAMMTQISTIYSLSLEVYRHCLDIDDTMDAFVMDLSSIVSLAKLFIMRRNSKHA
YVLINSVLKDWSAVNDSRHEDIMTEYYKKGRIVSLTILYLGYASGLSFIVKALPFGEVLPFKMFQNSANSSANLKGSLEMNYFLASYCLFGPL
PLLPQICVLILQAMHIFVNAVAHCANDGLFFSLTMHLCGQFEVLKMNFTKFELQEFGCHKKLRFLVKRHCQLLMLANDLEQTFNMIILVQLL
MSALLICIEGFVFLVCLATRDNVGALKSVVLMVTLLIQLYLYAYAGDVLESRSNEIAHGVYDSPWYQPRGHVARDLMMIINRGHRSYHVTA
GKFLSMNIFTFKEILRSSASYLSVLKVMMDT
>BtOr51
MKVSTSKDFAYAMTPLKILSWPVGTWPLQDYDVFSGIRAITATFFLLLMIMIVQSEMYLDNSDAEKNLDGLVFITCGSLAASKVIQFRIRPAA
LISNFTSAVKDYNELRDEEKRVIVRKHAYMARVASASMIFFAYFSSILFITVPMLAEEEEKDIVNVTEESTSEYPIPSENVMALVKIPENLYFIVL
IIEYLMLLFTSTGNLGSDSLFFGITFHLCGQVEILKLDFQRLKIEGERTREHFNVLTKRHIYLIKLANMLNETISSILVMQLFTSCILICTSGLQLIL
ALNIGNIVMVIKTFIVLSTLMVQLFAYSYVGEYLRRQMEGIADSMYFCNWYDIPKSVAKDIIYVIMRAQEPVFLRAGQFLVVNMETYTSIIKTS
MSYLSVLRVMVNA
>BtOr52
MKAALNKDFAYAMIPMKIMSWPVGTWPLQDYNIFSAIRVIITSFLLLLMVTIVQSEMYLDSKDAEKNLDALVILSCGILALSKIIRFRIRPAALI
SNFTSAVEDYNELWDEEKRVIVRKHAYMTRVASASMLFFAYFSSIVFITVPMLADEEEKNVVNATEESTSEYPIPSENVMALIKIPENLYVIIFII
EYLMLLFTSTGNLGSDTLFFGITFHLCGQVEILKLDFQRLKIEGERTREHFNVLTRRHIYLIKLANMLNETISSILAVQLFTSCILICTSGLQLILA
LSIGNIVMVIKTFMVLSALMVQLFAYSYVGEYLRRQMEGIADSMYFCNWYDIPRSVAKDIIYVIMRAQEPVFLRAGQFLVVNMETYTSIIKTS
MSYLSVLRVMVNG
>BtOr53FIX
MRATLNKDFAYAITPMKIMSWPVGTWPLQDYNILSAMRAIFTIFLVLLMLMIVQLEMYLDSSDAEKNLDGLVLITCGILAMSKILQFRIRPAG
LISNFTSAVKDYNELNDQEKRVIVRRHAYMGRVAGISVVFFAYFGSTLFMTLPMLAAEEVEDIVNVTEDNTPEYPIPSEKVMELIKMPDNLYF
IVFIVEYLMLLLTSNGNLGSDSLFFGIIFHLCGQVEILRLDFRRLSNDNERTIEHFIALSKRHVYLLKLAKMLNETISSILAVQLFTSCIVICTSGLQ
FIIALSVGNIVMTIKSFIVLSTLLVQLFAYSYVGEYLKRQMEGIGDSAYFSIWYDIPKSVAKDIIYVIMRTQDPVFLKAGKFFIVNMETYMSIIKT
SMSYLSVLRVMVTA
>BtOr54FIX
MKVTLNKDFAYAMTPMKILSWPVGTWPLQDYNIFSAMRVIITSFLLLLMLTIVQSEMYLDSNDAEKNLDALVILSCGILAVSKVVRFRIRPAG
LISNFTSAVEDYNKLYDQEKGVILRRHAYMGRVAGIGVVLFAYFSATLFMSVPMLAAEEVKDVVNVTEDNTPEYPIPSEKVMALIKMPDNL
YFIVFIVEYLMLLLTSNGNLGSDSLFFGIIFHLCGQVEVLRLEFSRLSNENEKAKEHFNVLSKRHVYLLNLAKMLDDTISSILAVQLFTSCILICT
TGLQFIIALSVGNIVMVIKTFIVLSTLLVQLFAYSYVGEYLKRQMEGIGDSVYFCSWYDIPKSVAKDIIYVIMRTQDPVYLKAGRFFIVNMETY
MSIMKTSMSYLSVLRVMINA

>BtOr55FIX
MKTTSNKDFAYAMIPFKILSWPVGTWPLQHYDIFSAIRAIITSFLLLLMITIVQSEMYLDSSDAEKNLDAVVILTCGYLAVSKVLQFRIHPAGLI
SNFTSAVKDYNELNDQEKRVIVRRHAYMGRVAGISGVLFAYFSATLFTTLPMLAAEEMENMANVTEESIPEYPIPSEKVVALVKIPEHLYFIV
FIVEYLMLLLTSNGNLGNDSLFFGITFHLCGQVEILKLDFKRLRNENERTKERFSVLTKRHVYLLNLAKMLDDTISSILAVQLFTSCVLICTSGL
QFIIALSVGNIVMTIKTFLVLSTLLVQLFAYSYVGDYLKRQMEGIGDSIYSCSWYDIPNSVAKDIIYVIMRTQDPVYLKAGRFFIVNMETYMSI
MKTSMSYLSVLRVMIST
>BtOr56
MKASRSKDFAYAMTPLKILSWPVGTWPLQDYDIFSATRAIIAISLLLLMLTILHIEMYLDSSDAEKNLDGLALITCGILAVSKVIRFRIRPGGLIS
NFTSAVKDYDELKDQEKRVIVRRHAYMARLACGSVISFAYFTSTIMMTLPMLVEEEDEGIVNVTEESIPSYPIPSEYVMAIIQLPDNLYFIVFIV
EYLMLIFLSTGNLGSDSLFFGIIFHLCGQIEILRLEFDRLNNENEKAMEHFTSLTKRHIYLLKLAKMLSETISSILAMQLFTSCILICTSGLQFIIAL
KIGNIVMTIKTFIVSTTLLLQLFAYSYVGEYLKRQMEGVGNSVYFCSWYNIPKCVAKDIIYVIMRGQDPVFLRAGKFFVVNMETYMSIIKTSM
SYLSVLRVMINA
>BtOr57
MKTSMSKDFAYAMTPLKILSWPVGTWPLQDYDVFSAIRATIATFFLLLMVTVVQSEMYLDNSDAEKNLDGLVLITCGSLAASKIIQFRIRPAA
LISNFTSAVEDYNELRDEEKRVIMRKHAYMGRVASASVICFAYFSSILFITVPMLAEEEEKDIVNVTEESITEYPLPSENVIAVIKMPDNLHFIVF
IVEYLMLLFLSTGNIGSDSLFFGIIFHLCGQVEILRLEFNRLNNENEKAMEHFISLTKRHIYLLKLAKMLSETISSILIVQLFTSCILICTSGLQFIIAL
SVGNIVMTIKSFIVSSTLLLQLFAYSYVGEYLKRQMEAVGNSVYFCSWYDIPKCVAKDIIYVIMRTQDPVFLKAGKFFVVNMETYMSIIKTSM
SYLSVLRVMVTT
>BtOr58
MSVLQPAFNILIVCGCWIPPSCRTFYGKLLYAAYTAFVIFLLCSFCISQFLNVILNVRTANELSDSFYMFIASILSCCKIFTLLVNHKAIRILSRKL
DEEPCKPVDEQEITIRRRFDKSIGSITIYYTIMVMLTVACMILFSFLTDFGDRKLAYKAWLPFNYSVSNCYYIAYAHQIIALIGTALLNVACDML
VCGLLVHVCGQQEILKHRVKELKKESRPDIGKIVRFHDYLYGYVSMIQQKFQEIIGVQLLSSTFVVCFILYELSNAPVNSKYLQFVLYLTCMM
TQVFFYCWYGNQLKLKSVEVANAIFEADWISFDNSSKKSLINVMRRATKPIELTCAYVFTMDLKTFVDILKMSYSTYNLLQRTKES
>BtOr59
MHLLRLTYRFLTICACWRPPLLSPLKNVAYTVYRYYVILLVYGGSICQFIDLIFIVETLDEFCDNIYLTLTFFISCLKMYSILSNRKNIIVITDMLE
STPFQPETKEEIEIREKCEKQARSNAFYYALLVESSVVVMSVLTLLGALYKGEHHKLAFRMWLPWNHTSTATYSFIYSQQILIHAFNGLLHVA
CDSLIWTLLMFICNQIEIFGYRLRKIEQGTNDCKLCIRYHNLIYRFATMINEEFKLMIFVQFAVSTLTICMNLYILTGTNVSLEMIVKIIMFSSCM
LTQIYILCWYGNEVELKSLEISNMIFEIDWLALKETTKRDLLMIMMRARSPIQMTSVYVVTMNLKSFVILLKTSYSAYNLLQGM
>BtOr60
MHTLRWTFALFTLTGIIRPSTWKYLWKRVLYDVYTIVVLLLLFSFETSLILDLVINVDNQDDFSENLYVTLVLFSSCCKALVLLIYRGNIEILMG
VLLEKPFAPVNDEEIEIRTKFEERIEWNSKAYSFVLHFFVAWLWIAALLTDFRHGRLKFRAWIPYDYNSPLLFLFSFIHQILATIFSTNLNIVCDC
LFSGILIHIYCQFEILEHRMKNITTDKNYSAKFCAHHHHRIYKFASMVNDNFKMIMSMQFLISTGAVCFNLYRLSVMEFGPKFMETATYTLCL
LMQVFYYCWYGNEVKLKSLEIPNAVLESNLPFLDDSSKKILLLIMRRALEPIEFTSCHVISMNLESFAILLKTSYSAYNLLQQSKLND

>BtOr61
MHVLRWTFKLFIASGYFLPPTLKSPTKRFLYNLYTVFVTLYMWSYCLTLIMHICYDVETQDDLSENFGISVTAVITSCKLINLVIGRKTIIHMLD
LLKKKPFVPENNGEVEIHAKYDNLIEKVAMFYTIQVVCCVLALVGTTLVSDFKLKKLMFPAWFPFDFTSSWSAFSITFIYQLVGLMIIATGISM
FDTFFAGLLLQICCQFEMLMNRLHNIEGNEIQSLKNCVWHHNTIFRFAEIVNKFFNKMMFVQFMVSTVAICFTLYQLTEANDSLQIIGWASFM
FSALMQTFYFCWFGDAAKVKSLDISNTVYNSDWANLSNNARKMLVIIMARSLTPVEITSAYILPLNLESFKGLMKTTYSAYNMLVQNKSSR
>BtOr62FIX
MTVRTLQVSRVLLSIAGCLPPSSWTSPFTKSLYKFYTLFVWLLILSLVSAQILDIIINVENQDQFSDNFYITLVVFVSGCKLSIILKHRESILSLIES
LEREPFSPMNDEEEKIQMKFNRTNERIAICYTILVEVAAIWIFVRAFLTDFKKRKLVFRAWLPYDYSELLPYTFSYTYEVATSLLCSCQNVASD
TLFAGLLIQINGQFEILEERLRNIEEDSNYSAKQCVKHYQQIYKFSKTVNEKFKIILFLQFCTIAFTLCFNLYRMTNITMLSKFLEASLFLIRIIAQI
LYYCWFSNEVKLKSLQVPSMIFKSNWASWDTKTKKILLVIMTRATHPIEFTSGYLVTLNLDSFVALMKTSYSVFNLLQQTK
>BtOr63
MHVLRWTLKLFIASGYFLPPTLKSPTKRFLYNLYTVFVTLYLWSYCLTLILDICFNARTRDDLRDNFSISVTILITGCKFVSLVLGRKTIINMLD
LLKKKPFVPENNGEGEIHARYNNLIEKVAVGYTIQVIFCVFSLLGTTLMGDFQSKKLMFRAWFPFDITASWFAFCMTFLYQFVGLVIVSNGVC
IFDTLFAGLLLHICCQLEMLVYRLHNIEGNEIQSLKHCVWHHNKIFRFADIVNNFFNKLMFVQFVESAISICFTLYLLTDIEDTAQLIGWSSFMF
AAIFQTFYFCWFGDVAKVKSLDISNMVYNSDWPNLSNDARKMLVVIMARSLTPVEITSAYILPMNLESFKGLMKVAYSAYNMLLQSKSAE
>BtOr64
MHVLRWTFKLFIASGYFLPPTLKSPMKRFLYNLYTVFMTMYLWSYSLTLIMDIFYNVETQDDLSENFSVTVTVLITSCKFVSLVLGRKTIINIL
GLLKKKPFVPENNGEVEIYAKYDNLIERIAMFYTIQNAFCVLSLIVATLMTDFKFKKLAFRAWLPFDFTSSWFAFSMTFIYQFVGSMVISAGISI
FDTLFAGLLLQICCQFEILVNRLHNIGGDEIQSLKHCVRHHNAIYRFAEIVNNLFSKMMCVQFMVSAAAICFNVYRVTESNAGSQLIGSVLFIFS
ALLQTFYFCWFGDVAKLKSLDIPNMIYHSDWTNLSNDAKKMLLIIMARSLTPVEITSAYILPMNLESFKGLIKTTYSTYNMLLQSKSSQ
>BtOr65
MHILRWTFLLFTLCGCYPPLSWKTPLQKFLYEIYTIFSFLLLNSFLLFQILDMICNVENTDDFSDNFSVTVVVFVTCLKLFTILIHRANFLLLCNT
LQKEPFLPMNEEEFEILLKFEKITDWNTLGYMTLLVISDFFILVVSLLANFKNRKLAFRAWIPYDYSSLSAYLLTFFYQSLFTTICTFGCVASDSL
YSGLLIHINCQFEILEHRLKNIESNQNYSVKLCVHHHDHIYKFGEMVNEEFKMIMFFQFGTSLTTICFNFYRITQIEMDSRFVGTLLYMACSLM
QIFYYCWFSNEVKLKSMELSDMIFRTDWTSLNNNVKKAFLMLMRRAMRPIEFTSIYVISVNLESFMTLLKTSYSAFNVFQQSRES
>BtOr66
MHRLPLSFALLTYCGYWRPTKWPHTSLKYQLYNVYSVLMILLLYFFAFCSCVDSFTSKNLNAMTDKFSLCVSVVGVCLKVANLFIRRGKIIN
VMNMLLNENCIARDDQEERIQRENDDYARKLAIYCEILNESAVFFATVGQYKKLMSMRELPVSDWMPYDLSSQKVYTISLVYQTVGLLICA
NTSVANETLIAGLMIQVGTQFEIFCHRARNLSFSLTSARRNTMSNEEFKIRCNKIIGNLIRHHHEIYKFAETVNSVFQYMIFLQFCISSIVLCLSVY
QFSTVDPFSMNFLWSGFYLCCMLMQVYLYCWFGNEVTLKSNKVRDAIYDMDWTMLPTDVMKNLLLIIIRTDKPVKMTSGHVVVLSSQSFV
SIMKMTYSSYNLLSSSTSK

>BtOr67
MHILRWTFLLCTLCGCFPPSSWKTPCRKFLYKIYTVFSLLVLNTFFLCLIMDMIYNVENIDDFSDNFHVTVGSFLTTIKVSVMLMYRESFVRLR
DTLQKEPLLPMNQQEFEILLRFDKVTDWNTLGYMTILMTSNFYLFMESLLTYKKRQLTYRTWVPYDYSSASAFLLTLLYQSLFTTICSFGCVA
TDSLYSGLLIHITCQFEILEHRLKNIESNQNYSVKLCVRHHNHIYKFGEMVNEEFRTIMFFQFYTSLCMICFNLYQITQMEMDSNIIGRILFMNFS
LMQIFYYCWFGNEVKLKSLELSDMIFRTDWTSLNHNVKRAFLMLMRRAMKPIEFTSIYVISVNLESFMTLLKTSYSVFSVFQQSRES
>BtOr68
MHVLRWTFKLFIASGYFLPPTLKSPTKRFLYNLYTVFVTLYLWSYCLTLIMHICYDVETQDDLSENFGITVTALITSCKLMSLVIGRKTIINMLD
LLKKKPFVPENNGEVEIHAKYDNLIEKVAMFYTIQVVCCVLALVGTTLVSDFKLKKLMFPAWFPFDFTSSWFAFSMTFIYQLVGLVIIAIGVS
MFDTFFAGLLLQICCQFEMLMNRLHNIEGNEIQSLKNCVRHHNTIFRFAKIVNKFFNKMMFVQFMVSAVAICFTLYQLTEANDSLQIIGWTSF
MFSALMQTFYFCWFGDAAKVKSLDISNMVYNSDWANLSNDARKMLVIIMARSLTPVEITSAYILPLNLESFKGLMKTTYSAYNMLVQNKSS
R
>BtOr69
MHTGQYTDVSIKMSGFLLKITGILKATNSSAERRRKFMVVYTVAALVYGVYVNVVDIYHNLHNLDHCIFLASNTLNIILAMFKLSVLHFYKT
EFSDIIVFAQKHFWHINYNDDEKILFAECRHFCKLWTVFILLVIESSLSFYAITPISANIGNNGSERLLPFKMWVNLPLTVTPYYEIMFAIELLAV
QQIGASYLCPEQFLCVLNLHVVYQFRMLQKTLLNLWSNIDEQTDIADYSKKYYIILKKCIRKHQSLIQFNAKLEQIFTLPILSHMVIFSVLMCFD
TYEIVLANISSGKRLIFFFHMIGSFAHIIFFTYICHGLVEESTNVSIASYSGWWVCLPMSKTGKKIRKDMKMMMMKAMRPCQLTAGGFFPVTL
ETSTALISSTMSYFALMRESSMRLTEQ
>BtOr70
MPAERYTDVSIKMSEFLLKITGLSRTTNSAEEIRRKLTIVYTIAALVYGVYVNVVDIYHNMDNLDHCIFLASNTLNIVLAMFKLSVINFYKTEF
SDMIVYAQKHFWHFNYDDNDKILFAECGRFCKLWTVFLFFVTQTALSFYAVVPISANIGNNNSERLLPFKMWVNLPLTVTPYYEIMFAIELLA
VQQIGASYICPDNFLCVLNLHVVYQFRMLQNTLVNLWSNIDERTDIVEYSNKCYVMLKKCIRKHQSLIEFSAKLDDIYTLPILSHMVIFSVLMC
FDTYEVILADVSPGTRLIFFFHMIGSFTHIIFFTYICNGLVEESTNISTASYSGWWTILPMTETGRKIRKDTRIMIMKSMRPCYLSAGGFFPVTLET
STALISSTMSYFTLMRESSIKAAAE
>BtOr71
MMRNNDISIIWASFLMKIVGLWLATNRNEQRRRDFALIYTVGALFISICIAIRDIYHTWGNFSDSVFICCNILYVTIVFLKIGVIYKHKIEFFNLIT
FTQKNFWRPYHDPQEILVVADCKRICNIFIILMIFCTQGTCAGYMVTPLIANIGRNESDRILPFNLWVDFPVGMSPYFEILFTIQILCVYHVGVC
YICFDNLLCIVNLHVACQFRILQHRLRSIDNATKDQIEEYESDAKLSCYSNMCYTKLKNCVQQHQMLIEYCKKLENIFTLIVLAQVMFLAMVI
CLVGFQLLLVDTPTSKKASLVLNLCGVLCQLLMFTYSCDDLMRQSVNVGNASFSGPWPILPMNEAGATVRKNLLIIIMRSHKICCITAGKFFP
VSLQTFTGVLSTAMSYFTLLRNTSLDATNS

>BtOr72
MDKDSKRTKVSPTSARLKLENEASYTKDFALLMTSFLMKIVGLWLTNNKKEERIRQLTLMYTVVAILFGVWVQFRDFYYSWPNFGDCAYT
ACNILCLIMVLLKLSVVFIHKKEFIELLVYTHENFWHTNYSYNELLLLQNCRKISIVCISLINICAQGTVFGYVLTPIVENIGRNHSDRVLPFRM
WLDLPLSVTPYYEILFVLQVLSLCHVGICYICFDNLLCLINLHVATQFRILQYRLLYLGETIEKQPYEYAIKEILPSDYLKNYHTLFKCCVREHQ
DRINYCQRLNNIFTYIVLGHIVVFSLLLCLVGFQVLMANSPPTRRLIFVFHIVGSSSQLLLFTYSCDTLIRESTNIGTAVYSGPWTHLTMNKIGKL
LRKDLTMIILRSSKPCCLTASGFFPVSLETCTKVLSTAMSYFTLMRQSFTN
>BtOr73
MSFLKSHDISISLTSTFMKLVGLWTSKNQLERRVRLITQIYAFAIILFALWVEITDTYYSFGDLSTCIYNVCNILAILMPLLKMTVLLAHKHEFFR
LIAYTQRRFWHENYDEYEKRIYMNCKRKCTVFVCFVIFTTKATLICYALSPILENIGRNESDRELPFNMWIDLPLTATPYYEITLLIQLMTLYHI
GVGYFCFDNLLCVMNLHLATQFQILQYKMSRMTDLTNKGKGETNLQLFSASSANKCYTVFKMYVQQHQALIAYCGKLESVFNLPVLAQVL
AFSLVMCLDGYQILMPGAPTRTRFIFSFQLIACLCQLLMFTYSCDCIIQESASIALAVYKGPWSFLPPTKSGMMMRKDLILVTIRSGVPCCITAY
GFFVVSLETYTRVLSTAVSYFTLLRQTTQETLYS
>BtOr74PSE
MHTEQYTDISIKISRLLLKIAGIWKSANKTEDRQRKFAVFYTMITSAYSMYVNVADICRNLNDLDXRCIFLAANVLNTILAVFKISIINLYKTEF
SYILYTPKNIFWYLNYDLDEKILFAECKKVCKLWSLFAILVIQIIVSFYAVASICANIRSNSSERTLPVAMWVDLPIYVTPYYEIMFVIQLASVQQ
ICVAYMSCDNFLCILNMHVICQVRILHNKLLNLWKIIDQQIDKIDYTDKCYAALKKCIRQHQSLIKFYERLEYVYRFPIFGHIVIFSLLVCFDTY
EILLXVSPGTLIFVFYMFGSFIHIIFFTYTCHGLIEESSDISLATYSGWWTILPITETGRMLQEDVKMMIMKTMRPCYLTAGGFFPVSLETSTALM
STTMSYFTLIRESSLKMKN
>BtOr75FIX
MSSRQVKDLSIIITSFYMKFVGFWLTNNYVEKRRRNVALSYTLFAVLLSLSTEARDLYFSWGDLGDSIYVICNVITIVLVVVKIFILLIYNEELL
DIIDYAKINFWHLNYDSHEQMIVDNCRRTCTIFVCVFTFFAQGTVIGFIARPILINYGKNESERILPFNMWLPECHLSMTPYFELMFMLQVVCS
YHVGVCYHCFDNVLCILNLHTAAQFRILQYRLTNMCNMNDCAEFYEVSKKSSYSIHRYAKLRTYIQQHQALTDFCKKLEDVFNLIVLGQVSL
FSLLICLDGYLILMDDAPATRRFTFAFHITGCMCQLLMFTYSCDCLIRDSANVANAAYKSLWSHLPMDQFGKILRKDLILVIMRSATPSCLTAC
GFFTVSLETYTGILSSAVSYFTLLRNQSNDN
>BtOr76FIX
MRFLKSHDISISLTSIFMKLVGLWTSKNQLERRVRLITQIYTFAIILFALWLELTDIYYSFGDLSTCLYNVCNILAVLMPLLKMTVLLAHKQELF
HLIAYTQRRFWHENYDEYEKRIYMNCKRKCTVFVCFVIFTTKATLICYALSPILENIGRNESAREFLFNMWIDLPLTMSPYYEITFMLQLMTLY
HIGVGYFCFDNLLCVMNLHLATQFQILQYKMSRMTDLTNKGKGETNLQFFSASSANKCYTVFKMYVQQHQALIAYCGKLESVFNLPVLAQ
VLAFSLVMCLDGYQILMPGAPTRTRFIFSFQLIACLCQLLMFTYSCDCIMQESASIALAVYKGPWSFLPPTKSGMMMRKDLILVTIRSGVPCCI
TAYGFFVVSLETYTRVLSTAVSYFTLLRQTTQETLYS

>BtOr77
MQTELDLDISMVLSKFFLRSIGLWISENSAEERRMKIMIIYTVWHSIFATVEITRDFYFTILYKGDILYVTTNILTVTMGLIKICIIMTHKEEFINLI
VYVQQHFWNVKYDFREKEILDNCKKTCTFFVCSVTIMGACAIVAYLTTPVIANAGKNNSERVLPFNIWLNLPVSMSPYYEMIFTLQMINMYQ
IAVTYFCFDNIFCILAIHLAGQFRILRYRFSKLCDIEHQINEKDMESMITNYAHTFYEKLKMYVRHHQTLINFCDRLENVYTMLILGQVLVFSV
LICLFAYQGLLAAAPLARRSIFIFHLIGSMALLFMFTYSCDGVIEHSEKVAIGAYSALWTIMPMNKSGKMLRNDLIMVIERSRRVCCLTANRFF
PVSLETYNKILSTAASYFTLLRNHLENEIEK
>BtOr78
MHTTRYTDISITLSQFLLKLAGVWMTVNNAEERRRRLTMAFTAVIHVYGLYLNLGDAYYTWNDLSHCTFLLSNTLCIVLAMFKLLILNFRRT
EFKDLVLFAQQNFWHFKYDHDEKILFMKCRKLCKLWTITACSFTQASLAFYIITPICANIGKNKSDRVLPFKMWVDLPLSVTPYYEIMFVIQL
ATVQQIGVTYLCSDNFLCILNMHVICQFRILHNRLLNLWKIIDQKTDKIDYADKCYIALKKCIRQHQLLIKFCEKLEYVNTLPIFGHVVVFSLL
MCFDTYEILLANVSTGTRLIFVFHMVGSFIHIIFFTYTCHGLIEESSNISLATYSGWWTILPMTETGRMLREDVKVMMMKSMRPCCLTAGGFFP
ISLETSTALMSTTMSYFTLMRESSMKNGDK
>BtOr79FIX
MQMQRDLDISMVLSTFFLRNIGLWITDYPTEERRMKIMILYTIWNSVFATIVITRDLYFTVLYKGDILYVITNILTVTMGLIKICIIMKHKEEFIK
LIMYVQQNFWNVKYDFREKEILDNCKKTCTFFVCSVTIMGTCAILAYLTTPLIANVGKNKSERAFPFNMWLNLPLSVSPYYEMIFTLQTISMY
YVSVSYFCFDNVFCILAIHLAGQFRILRYRFAKLCNMERRMKENDMESALTKHAHVFYEKLKSCIRHHQALITFCDRLENVYTMLIFGQVLVF
SVLICLFAYQGLLAGAPPARRSIFVFLLVGSMALLFMFTYSCDSVIEHSEKIAIGAYSALWTIMPMNKSGKMLRNDLIMVIERSRRVCCLTANK
FFPVSLETYTTILSTAVSYFTLLRNRVEDEIVN
>BtOr80
MSSKKGRDLSITLVSFYMKIVGFWLVKNHVEERWRNFAMIYTTFAIFVAIVVEMRDLYFTWGDFSGTVYIVCNLVTIILVLFKILVCFVYKKE
LLSLIRYAKTNFWHSNYNSHERMIVDRCKRTCTVLVCVFTFFAQGTVISYVIGPIQANIGRNETNRILPFNIWLNAPICYMTPYFEVEFIIQVLCL
YHVGVCYLCFDNILCIINLHTAGQFRILQYRLENMCGVNNNGKLSYSVCKYMKLKTYIQQHQMLIEYCKKLEQVFNLIVLGQVSLFSLLMCL
DGYLVLTEDAPLTRRLIFLFHITGCMCQLLMFTYSCDCLIRDSTNVANAAYKSLWSFLPMDKYGKILRRDLVLIIMRSNIPCCLTASGFFVVSL
ETYTGILSTAASYFTLLRSHANDMS
>BtOr81
MSSKEVKDLSITVTSFYMKFVGFWLTNNYADKRWRNIAMSNTVFFIFVAITIELRDLYFTWGNFEDTIYTACNVVTIVLVLLKTFVIFIHNDEL
LYLINYAKTNFWHSNYDSHEQMIINTSKRICTFLVCSFAFFAQGTVASFILRPILVNYGKNESDRIHPFNMWLDDSLMLSPYFEIVFVIQIFSACL
VGTCYHCFDNLLFVINLHTASQFRILQYRFSNMCNINDREHYTTLESYTVDKYATFKTYVKQHQMLIEYCNKLENVFSVIALVQVTLFSLLIC
LDGYLILMEEIARIKRLTFIFHVMGCMCQLLMFTYSCNCLIQDSECVMNATYKSSWSPLPMDKYGKMLRKDLMFVMMRSRAPCCLTACGFF
AVSLETYTGILSSAVSYFTLLRNHAGDT

>BtOr82
MQTEQELDISMRLSAFFLRHIGLWIANDPADERRMKIIFTYTIWNLIFGMIVLSRDLYFTWFYNGDTLYALANTMSLILTLVKVCVIVMHKKE
FMNLVVYMEQHFLAVKYDFHEKEILNNCRKICAFFISSVTTIGICAILSYISTPFIAQIGNNESTRELPFNMWIGILSQSPYYELIFFAQIMSLSYIG
ICYFCFDNVFCVMAIHLGGQFRILRYRFSELCNIKYQISEQDKMSILTKHVHRTYETFRKYVRQHQALINYYNTLENVYTVIILIQVLVFSVLIC
LFGYQVLLANTNSARRSIFVFLLIGALSLLFMFTYSCDDVIEHSDNVAIGAYSALWTIMPMNKHGKMLRNDLIMTIERSRRVCCLTANGFFPV
SLETYTKILSTAMSYFTLLSNNVTKNET
>BtOr83
MSFIKTDDISVRLTSIFMKLVGLWMATERSEQRIRDITVGYTLVAIIFAMWLQTTDMYYSWGDFSACLFAACNILSLTMPFIKILVLLAHKEDF
FRLIFYLQRKFLHGNYNDYERKIVISCKRKCTFFICFFTLFTLATVASYIINPLVANIGRNESDRVLPFNMWIDLPLTVTPYYEIIFVLQVLSLYHI
GISYFCFDNFLCIMNLHVAGQFRVLQYRIANMPDLMDKVKQSGDKILNTGSSCLANECYSIFKKYIRQHQALIAYCGKLEEVFSLIILEQVLMF
SLLICLDGYQVLMADASTSIRVIFICHILACLCQLLMFTYSCDCIIRESASIATAAYKGPWLVLPMSTSGRMMRKDLTLIILRSHIPCCLTGKGFF
VVSLETYTSVLSTAASYFTLLRQRETP
>BtOr84
MSLKYHKDVSFSVAVFYLKIVGLWLSTNLVEKWFRNALVTYTVLAIIFNMWMQLRGLYFSWGDFSVSTYIACNSLGLFMDLFKLLIIFIHKK
KFLYLVMYMQKNFWHFNYNQYEKSVLADAKRMCIYFVCVFSFLSQSTIFSYIFRPLISNIGKNESDRVPIFHMYLDLPLNVSPYYEMTYLIQA
LTLYQVGVCYLCVDNIFCIMCLHVASQFRILQYRIANVLSLKDKVKFDQDTNLDSSGEFYAIFKKCIQQHQALIGFCTTLEEIFTVIILGQVLTFS
ILICFVGYQALLVKLSLSWRISLVSFLTTNICQLWIFTYSCNALVQESMNTANAAYATPWIFLPMDKFGEMARKDLQLVLMRSRRACYLTAC
GFFPISLETFTKIMSSAMSYFTILKQRTVDT
>BtOr85
MQTEQDLDISVIFSTFFLRNIGLWISDNRAENRWMKIKLMCTLFNSIDSSIVIMRDFYFTWLYKGDILYVTTNTLTVTMGLIKICIILIHKREFISL
ITYTQQNFWNVNYDTREKEILKNCRKTCTFFVCSVSAIGVCAMLAYLSTPLITNAGKNKSERILPFNVWLNLPVSISPYYEIGFLIQTISLYYIAV
SYFCFDNVFCILAIHLTGQFRILRYRIAKLCDMDHQITEKDVELMITKHVHTFYEQLKLYIRQHQTLIDFCNRLEDIYTMLIFGQVLVFSVLICLF
AYQGLVVAAPFARRSIFIFLLIGSMALLFMFTYSCDGVIEHSEKVAIGAYSALWTVMPMNKPGRMLRNDLIMVIERSRRVCCLTANKFFPVSL
ETYTTILSTAVSYFTLLRNNVEDKNVD
>BtOr86
MIDRDIDLNNIYAWNYNLLKFMGIWPEERKWNRSSSYLVLFPFLTMVCFACGPQTINLSIIAGDSDLVIENLSNNITFMVSIMKTLTVWINGIPL
KSLLGYMANDWDAVTNNIERETMVNTARITRKITIGSTLMVNIVILAFVPARLSSMKNNDITLFLRGYYPYNTSISPNFELTMIAQYVAAIYAA
NTYTTVDTLVVLLIFHVCGQLAILRQDLGKIHSYDKKNIEMKMQKIVEKHEYINRFAGRIENSFNMMLLFQMLSCTIQICSQFYQVIMSLGENT
MEDMILQISFLLIYVAYVMLQLFLYCYMGEKLAAESTEIANIAYSTKWYNLPPKNARWLVIIMCRARSSPLQITAGRFCSFTFALYCQVLKTS
MGYVSVLHAMKNQ

>BtOr87
MIDRDIDLNYMYGWNYNMLKFMGIWPEERKWNRPYSYFVLLPCIVMVCFICAPQTFNLSIIAGDSDLVIENLSTNITITISLMKTMAVWIKGK
PLKSLLRCMANDWNTVTNNADREKMVNIARITKKITIRSILLANIVIVAFLPARLSSMIYNDKELFYRGYYPYNTTISPNFELTLIGQLMAALY
AAITYTTVDTFVVLLIFHVCGQLSILRDDLRKIHSYDNKNVEMKLQKIVQKHVYINRFAETIEDSFNMMLLFQMLGCTTQLCSQTYQVLMSL
GEEAIEHMILQITFLLIYVIYVMLQLLLYCYMGEKLTVESTEIANTAYSAEWYNLTPKNARWLVIIMCRARSSPLQITAGRFCCFTLVLYSQVL
KTSMGYVSVLLAMKNK
>BtOr88
MIDRDIDLNYVYGWNYNMLKFIGIWPEERKWNRSSSYFILLPCIVMVCFICAPQTINLPIIAGDSDLVIENLSTNITITISLMKTMAVWIKGKPLK
SLLKCMANDWDTVTNNADREKMVTIARITKKITIKSILLANIVVAAFLPARLSSMIYNDKELFYRGYYPYNTTISPNFELTLIGQLMAALYTAI
TYTTVDTFVVLLIFHVCGQLSILRDDLRKIHSYDDKNVEMKLQKIVQRHVYINRFAETIEKSFNMMLLFQMLGCTTQLCSQTYQVIMSLGEEA
IEHMILQITFLLIYVIYVMLQLLLYCYMGEKLTVESTEIANTAYDAEWYNLPPKNARWLVIIMCRARSSPLQITAGRFCCFTLVLYSQVLKTSM
GYVSVLLAMKNK
>BtOr89PSE
MLSFLFPRLQSNTQSVSEADLGALHPATFPYDISKSQNFEIAWPGQFMSSVLTAICYSCFDTFLAVLVLYLCGQLTVLRMAHENPTYATKKNN
YAKFYERLRFTVNQDNQLSRFAAILEDRFNFAILIRVIICTSLYCLTGCRMITSVNQVQADLPIVGMIFLIIHVIYTALLLLTYDHVGEMLRGQS
AGVGQSVYDCNWHLPPKDIISLIIVVCRAKVSFQGTAGKFSSFSLEFDT
>BtOr90PSE
MTDSDIDVKYMCGWNYYMLRFMGIWPEERKWYRAYSYLVLLPCIMMLCFVCAPQTINLPMIASDSDLVIENLSTNITITLSLMKTIVIWFKG
KSLRPLIKCMVNDWDTVTNEAERETMVNISIITRKTTMRSTLMVNIVVLAFLPARLSNVRYNDSALFFRGYFPYNTSISPNFELTMIGQFVATV
YAANTYTAIDTFVVLLIFHVCGQLSNLRDDLRKIHTYSKKDVETKLQKIIQKHEYINRFAATIENSFNMMLLLQMLGCTIQICSQSYQIIMSFEE
EAMEYVIFQITFLLLYVVYVMLQLFLYCYMGEKLAAESTEIANTAYYAEWYNLPPKSAKWLVIIMCRARSSPLKITAGRFYWFTLALYTQVL
ETSMGCVSVQYAMENKQ
>BtOr91FP
IDRDVDLNNVYAWNYNLLKFMGIWPEERKWNRSSSYLVLLPCIIMLCFACAPQTINLPIIAGDSDLVIENLSTNITVMVSLMKTLTVWINGIPL
KSLLRYMANDWDKVTNNTERETMVNIARITRKITIGSTLMVNIVILAFVPARLSSMKNNDITLFLRGYYPYNTSISPNFELTMIGQYVAAIYAA
NTYTTVDTLVVLLIFHVCGQLSILRQDLEKIHSYDKKNIEMKMQKIVEKHEYINRFAGRIENSFNLMLLLQMLSCTIQICSQSYQLIMSLGENT
MEDMILQISFLLIYVAYVMLQLFLYCYMGEKLAAESTEIANIAYSTKWYNLPPKNARWLVIIMCRARSSPLQITAGRFCSFTFALYCQVLKTS
MGYVSVLYAMKNK
>BtOr92NTE
LKTAIGWNRWNMELIGIWPEPRRTDERLSHFKALFYLSIITIFGTGPQSANLFFIWGNLELVTENLSTANIPGINAMIKLIFAWYYKDTFKPIMK
SFYDDWRSAKTEEEKAAMLKMAKPANFISIWCSILTLTMVTAYLSLRSITVYLSDRLHENQDRLSLYPGYFPYNIRPVPILLMTNFAQVIAGYS
ATICYTTVDTFIAMLVLHICGQFEILRKKLSRLMDGEGGNRSIDEFQKELVWIITKHEHLNWLAETIEKCFSTLLLLQMLLCTIEICFQGFLFFNV
LIKNENEIFNFQLVFFVLFVCFILVHVYLYCYIGEMLLIQSREMSNCAYESNWYNVSPSETKCLLFIMNRSTRPLCLTAGKFGIFSMELFSTILKT
AMGYLSVLLTVANND

>BtOr93
MDFAMGWNRFNLTLLGVWPEPRKVSRGSRLLSSIIFWFTTIVTFTFICAPQTANLVLKSTNLDEVIENLSINIPIAFALVKQIVLRYYKKALTLL
LSQMFDDWTEPIANQDRQMMLKNARISRLISIVCSTLTYLMLFAFISLQIWSNMQSASEADLGGLLHPATFPYDTSKSPNFEITWLGQFMGTV
LTAICYSCFDTFLAVLVLHLCGQLTVVRMALKDLANTMKKDNNYERFHERLGFIVNRHNLLSRFAVIVEDCFNLTLLIQTVICTAMFCLTGYR
MITSVDQEEADVPIVGMIFFIIHVIYTMLHLFIYCYIGEMLLGESNGVGQSAYECEWYDLPPKNAISLIIVICRAKVSFQITAGKFSPFSLELFNAV
LKTSAGYLSVLLAMKD
>BtOr94
MDFAMGWNRFNLTLLGVWPEPRKVSRGSRLLSSIIFWFTAIVTFTFICAPQTANLILKSTNFNEIIENLSINIPIAFALIKQLVLRYYKKALTLLLR
QMFDDWTEPIANQDRQMMLKNAQISRMISIVCSTLTYFMLLAFISMQIWSNMQSASEADLGGLLHPATFPYDTSKSPNFEITWLGQFIGTMLA
GISYSCFDTFLAVPVIHLCGQLTVLRMALEDLANATKDDNYARFQERLGFIVNRHNLLSRFAVTVEDCFNLTLLVQTVICTTMFCLTGYRMIT
SVDQEQADVRIVGLIFFIIHVIYTMLHLFIYCYIGEMLLGESTGVGQSAYECDWYNLPPKNAISLIIVICRARVSFQITAGKFSPFSLELFNAILKT
SAGYLSVLLAMKD
>BtOr95
MIDRNIDLNYVYGWNYHMLKFMGIWPEERKWNRPSSYHVLLPFIMMVVFACAPQTINLLLIAGNSNLVIENLSTNITTTISLMKAMAVWIKG
KPLKFLVKCMANDWNTTTDKAERETMVNIRRITRKTTIRSTLMANIVLLAFVPARLFSMRYSDNMLFYRGYFPYNITISPNYELTMIGQFMAT
FYAATTYTAVDTFVVLLIFHVCGQLSNLRDDLRKIQSYDTKDVEKKLQKIIQKHEYINRFANKIENSFNMMLLLQMLSCTIQICSQSYQVIMSF
GEEETEYMILQLSFLLIYVVYVMLHLFLYCYMGEKLTSESAEIANTAYNAEWYNLPPKNARWLIIIMCRARASPLKITAGKFCSFTLVLFSQVL
KTSMGYVSVLHAMKNK
>BtOr96FIX
MDIFGEKQTKFKKIHPNKHLQNSLSIIYYLGFWPEWAKNKYLYNIYTTFNLTFLLGIIIVSNIVYIIINWGNIEAMMASLSILMTYSTYGTKVIYII
CRRRRIKDLIDITNSEMFNRDNDKYEHITAYYTWQAIFHQITFQTFGFIAVISWGSTPILYQISGTSKQLPMVGWYPYNVTSTPVFEFTSLHQFM
VVCTSCVNNIAIDTLATGFIVTACCQLTILNYNISSIRCIIEKKYTLLNDNVSIENSTLKVYSKMYEDLKHCIEHTIMIFDFSKQIQDVFGTLIFLQ
LLVDCIVICAILFNLSQMKDYVTSEFLGTLLYTCCVIYQIFIYCWHGNELHFHSMRICSSAYANNWWDNSKDFKHALLIIMARAQWPLILIVGN
VMELSLQNFVLILRTSYSIFTVLRTSTAT
>BtOr97
MKELSNTFDYYILPNKILCSIVGMWPIEQESSTCTKIFSYVRLTFTLVSLISVFVPEIMLIAVNWGDIEILAGAGSIVTTLGQTLFKMFYLIARRER
SYMLYYEIKSLWDTANDSKEIQSYTQFAYWARICTIVFYSSCVCNVITFSSAAAVDYFRFDYNASGTSNNRHLPFIVWYGTDISASPKFEIAFIC
QLLSAALSITSIAGLDCSFMTTMLHVSAQFKLINTWISNIGTEINCNPNYKQKIKVDLTRCIRHHQRIIHVVNEVNNLLTPIVFLQILTSGIEVCLS
GYAILDTGKAEADLVKFICYFISMAVHLLLWCWPGEILVQESQEIGHAVYFNVPWYDLPPIYQRYLCLMIVRAQQYSSISALTFQTLSIHTLTA
VFNTAASYFTLLQQIQQT

>BtOr98
MKELSTTFDYYILPNKIFCSIVGIWPIEKRSSTYSKIFAYFRLIVSLIAISNFFVPEIMAVAFYWGDMETVIGIGSNLMSATQLFFKMIYLVARRER
VYRLYNEIRILWDSTDDPNERKSYEQIAYRARIVTITFSSCFLCNLTTFSIATIADYFRFAYNGNDTNDNRHLPFLVWYGTDISASPKFEIAFAG
QIMTAMIGLSAITAIDCTFMTMILHVSGQFILIKTWINKIGFEMNHKSIDMDKFEEDLFKCIRHHQRMIHVVNDVNNLLTPIIFMQLLTSGLEICL
SGYAMLDNGTKITDILKFTSYFISVTVQLLLWCWPGEILVQESQEVGQIVYFSVPWYNLPPIYRNHVCLMIIRAQQYCSITALTFKVLSIQTLTA
VFNTAISYFTVLQQMQQN
>BtOr99FIX
MKELPNTSIDYYILPNRIFCSMVGMWPIEEKSSTCSKIFAYIRLILALTAINSVFVPEIMMIVSSWGDITILAGVGCVLTTVGQLLFKMIYLIVRRE
RSYRLYYEIRSLWNTANDSKEMQSYVELVYWARICTIVFYSSCMCNVITFSIAGVVDYFRFEYNASSADNNRHLPFVVWYGTDISASPKFEIA
FICQILSSMVCATTISGLDASFMTTILHVSAQFRLINTWISNMGIEINCNPNYTRKIKIELMRCIRHHQRMIHVVKEVNNLLTPIIFMQILTSGIEIC
LSGYAMLDSGTAKADLVKFISYFISMGIQLLLWCWPGEILVRESQDIGQVVYLNVPWYDLPPIYQQHLCLMIVRAQQYCSISALTFQTLSIHTL
TAVFNTAASYFTLLRQIQEK
>BtOr100
MKELPNTSIDYYILPNRIICSMVGMWPIEEKSSTCSKIFTYIRLILALIAINSVFVPEIMMIVSSWGDISILAGVGCVLTTIGQLLFKMIYLIVRRER
SYKLYYEIRSLWDTANDSKEMQPYIRLAYWARICTIAFYLSCMCNVITFSIAGVVDYFRFEYNASSTDNNRHLPFVVWYGTDISASPKFEIAFI
CQILSSMVCATTICGLDTSFMTTILHVSAQFKLINTWISNIGTEINCNPNYTRKIKIELMRCIRHHQRMIHVVNDVNNLFTPIIFMQILTSGIEICLS
GYAMLDNEAAITDILKCTSYFISVTVQLLLWCWPGEILIQENQEVGQVAYFNIPWYNLPSMYQQHLCLMIVRAQQYCSISALTFRTLSIHTLTT
VFNTAASYFTLLRQIQEK
>BtOr101
MINRPLEYSLRLFGVWPDSSYPILKIIIWTTVMLTFLVFQYWYCITHIKSSLIELLDGLSITLSNSLFFLKFTIIWLHKRTFYEILTTMFEDWNVDN
STARNKRIMSDKAILSFRISNFLIGYFAITFFVYAGLALTLFDEDQLASDSKQRKFLIRMEFPFAATISPRYEVILAIQFIFESVMVYGAATSIALIA
ALILHVGSQIDLLCCKLTEISHNCDKEKLQKHIIDDIVGKHQRIIQFSKNIEKIFTYISLCQFVSNMLVICFISFILVVSSHMDQATVIIMKCFPYYI
AVNCEAFILCYTGEYLTSKSEDITKSVYNFLWYELKPQNARVILLMILRSQGKLTLTAGKFLCLSLEAFANMLKASASYVSVLYAMY
>BtOr102
MAIKGIISYPVEITLRLIGAWPNSSCRIFKYIIWTTVMSTFLIFQYSYCLTHIKAADLTDLLDGLSVTFSNTLLLLKFIIVWFHKQTFSEILIIMAED
WDNCKSEWNMDVMMQKAILSYRIAKVMLIVFTCSISLYAVTTFFVPDNIEASHSTEKKFLLRMEFPFEATFSPIYEIIVTIQLVIQPIFTLMAGM
FMALIATFVLHLASQIDILCDRLTEILNNHSEEQLRITVIKNLIVKHQRIISLSENVESVFTLISLLQFFFNTVVICFVGFLLVTSLSSGQAPAVITKC
FPYYIAVNFEALILCYTGEYLSSKSEDIGWTVYNSNWYRLSICETRALLLLILRSQKPLNLTIGKFMNLSLETFANMLKASASYVSVLHAME
>BtOr103
MILSRSISRPVVIGLRLIGIWPGSSYEIIVRCIWVANMMSAQVFQYRYIIKHINSGNLADIVDSVSTTLPYSLLFFKIISFWIKRGIFKNILIGMSRD
WIDTSTAKLNVHVMINKAELAYRCSNLIIGVYASAVCIYGGMFLEFSRQDQDDGFNITSRQLLIKMDLPFAYYESPMYEYVFVVQFLQLLAT
GISIAMLDALIITLILHIGGQIEMLHEALENISIKDEKHGSLRNVIKSLTDHHYRIILNSEYIEKLFSYIALMQLLCNTIVICCIGFLIIVAVNSYGDIK
ILMKILLFYIAITLEAFIFSYAGEYLSSKSLSVSSSAYGSSWYLLEPRNRRVVILLMIRSQRSLTITAGKFMDLSMIGFATILKASASYVSVLYAM
Y

>BtOR104FIX
MILNTISPSVKFGLHFCGVWPGTPFQFIHKLCWVIAIITLQIYQYKYIVIHYNTDTLMNIAENLSITVPFSLVLIKFFITWTNYGLFCDILSTMEED
CQKYANMDINNLITKAGVVSFYATSMIMSFYLATTIYYLGGTIAFQGTNDSMSRELLLKMDLPFETSESPNYELVVTTQFIIHISAAFAFATFTA
LLFMTIVHVGCQIDIMCQNLTYAFPKNENKLKFFIGRYQEIIAFAEKIEKLFTYIALSQLISNTVSTCCEGFLIVLAVNDQSGLVVLIKCVLFYIVI
CSEVFIYCFAGEYLRIKSQLIGDTAYNMLWYDLRPNKSRLLIPVILRSQRGFTLTFGKFSNLSLESFTGIMKVSASYMSVLLALY
>BtOr105FIX
MILNTISPSVKFGLHFCGVWPGTPFQFIHKLCWVIAIITLQIYQYKYIVIHYNTDTLMNIAENLSIAVPFSLVFIKLFVTWTNYGLFCDILSTMEE
DCQKYANIDINNLVTKTGVFSFYVTSMIMSSYLVSAVSYLGGAIAFQGTNDSMSRELLLKMDLPFETSESPNYELVVTTQFIIHFSAALAFGTF
TALLFMMIVHVGCQIDIMCQNLTYAFPTNENKLKFFIGRYQEIIAFAEKIEKLFTYIALSQLVSNTVNTCCEGFLIVLAVNDENGLAIAILIKSVV
FYIVICLEVFIYCFSGEYLRIKSQLIGDTAYNMLWYDLRPNKSRLLIPVILRSQRGFTLTFGKFSNLSLESFTGIMKVSASYMSVLLALY
>BtOr106
MIPSKSISRPVVIGLRLTGIWPGTSYEIIARCIWVAIIMPAQVFQYRYIMKNVSSGNLADVIESASTSLPYTLLLFKLISFWIKRGIFKNMLVGMY
YDWINSSADKANVDVMMNKAELAYRCSYSIFGVYIISVFMYAGVFLQFIRQDQDDFNITSRQLLMKMDLPFAYYESPVYQYVFLVQFLQLL
AVGIGMAILNALIITLIFHIGGQIEILHEALENISIKDEKHGSLRNVIKSLINRHYRIILNSEYIESLFSYIALLQLLCNTVVMCGIGFLIIVAINSHGD
VGIVVKIVLFYIAIMLEAFVFSYAGEYLSSKSLSISTSAYGSSWYLLEPRNRRVVILLMIRSQRRLTITAGKFMDLSMFGFASILKASASYVSVL
YARY
>BtOr107
MAPINTISRSVKYGLHYAASWPGAPLSVLCKLFWMIVLGVGQTHQYNYIIKHYKVQTLIEIIDNISICLPFSLVCIKLVIAWTHQGLLHSILSTM
EEECQTYAVMDTNNLISKTAHWCYRLTNIIISTTIASTVFYVIGVFTSEGVNATAPRELLLKMDLPFDTSKSPTFELVIIVQYFYQASSAFIFAVF
TGLLLMIVLHIGCQIDVMCQTSSAISYKNEKQLKFFISRHQEIILFAEKIEKFFTYIALSQLITNTLIICCLGYLIVLIIHIENGFPMFMKCVVFYISV
CSEAFVYCFAGEYLNIKSKLIADTAYEFLWYDTHPSKSRLLIPVILRSQRGFSFTLGKFANLSMSTFAAIMKASGSYISVLLAMT
>BtOr108
MRKHFYSLSRGRTSYVKMAQEPGHEWMKPFRKLINLEISYIKYSGLGEIDSSSAFLNRAYFVYKIWMLIAMYIFAITLFLDIYVNRDNLPISTD
NGCIFAGIFVVIYKAMNLQFQVEYIKEIIGKYQVQSKMIFYGFTALGSILGTALLFFSPMEDGLLIRAKYPFNTTISPWHEISLAIETCAVFGGLL
GIIGIDSFVVMICTLLTVLFDMLNVNFENCGIETKEHTVEIYDERIHANIRFNRKKLNNRFLYRYKTCFQFYQRLVCMTNDYNKLFSLSMFIQM
LSSTSIICLSGFQAVVVGGQSSDVMKFGIYLSAAISQLLYICWIGNELNYATWTLDRSQWLSGWNNERLTNIVKMFTLSTMFTRRSITLKASVF
YVLSLETFITIIRRSYSIYTLLNNMQVTDH
>BtOr109
MNIRNYVFINQLVLKFVGLYPISIVRYVICISCIMLITIPQIIMIYTNWNDLNIVMEIGSTLLTISLAMLKSAIWMFNRKNLELFIEFMLTDYWKIIE
ANVFEYLQKYALYAKTITKGYLISMCNALLFFFSLPIIEILITKHEDSDNFTMNFPFAASYPMAFYKFPLYEIAYASQILATSICCLVMLATDGLI
ATALLHTCGHFAVLRKNVKQLDSYIYRITSLKTNSKHINANLYEIKIQIIHVIKHHQVVLWFCDNMEKNFHLILFLQAMISSLLICFVGFQVSAT
LMEQSKMIKFASHLIVAFFQLLLFCFPGDMLIRESFSISTAVYSIQWSQLATFVKNELCMIIARSQRPSYITAGKLYIMHLENFTAILSTAFSYFM
MLQSFNSES

>BtOr110
MSDDETKREFEKTIDLNLALLRLTGVVSSERASRDTLASLAFVCMTINTISYVYEFATSSYTLATVLESFAMIIPLVAGQTRLVILLWLRDSCQT
MLNICESFWSTLNSREKKIVRSYTNKAKLLSRCYLFSCVSTIFFYVLLIEFGSLIFSEPKHFANVTIHGNDSSSVPSEAVKSLEVTDDKRRYLPYA
FFIDVQETPWYEIAYVVQLGSMVSVGLTCVGVDTTGPLLILIACGYFDTIQSRIENSYSFESPSSPPILASLSIATKAKTATETTLVTGIESSSRNV
GMKNLRTCLNHHQLLLKLCEDIENLTNIMFLIQLITSTYNISLIGFKVVEDNPGKVKFVTQLGIFIIQLFLCNWPPDLLRSKSEAIGHAGYSMPW
YRYPRNLQNPVNMLMVRGQKPVRLTAGKFIELSLETFASMISTAASFFTMMRSMN
>BtOr111
MEMSDQQFSGKEYDELIKPIMITAKIISIWPLEEDSSKGTILFRRFHLFCMFFLAVVMSFAVTADVVHNIDDLNEATECALICTAFYLCVVRLLV
YSLHQKDMFYVVKTMKEDWILSSHEDRKILAKKTMFAFRLAKYFISTVAMTIVLFMCIPFLEIYVFGNNERVLPFRGYFFINHTISPVFECLYF
FNVTAGGFGGSMIAGATSFNLVVIMHGSGKFAVLRKRLEALSGEDPNSTAIMNNYVIRHQKAIEYADALERIINVLALGQFVTSTGLICFAGF
QITSMMKDKGRLMKYSTFLNSAILELFMFSFSGNGLIDESGAVGDSAYGSGWIGSRFNQNLQIMMMRARSPSKITAAKFYAMSLESFSAVLST
SFSYFTVLTATEGD
>BtOr112
MKKPISSYVELFYDENVISWSKRLLNLSGLWPHNRNDVRFFFYVTYVVIFTWLEIVTLLQNIHDLEKTLKNITLSFPTILIVLKAMMFRLNMHL
VLPLLTVVKRDVKLGLYRSEEERRTVVWYNVAATLFSTSSVLSLFFVPTLFYAKPIVSCLLSKFNNCTLPYELPMKVNNIYEVTGVQSYALLC
VYLIPASMLLTIGATGADSLLVALTFHLCSQLSILAHRIRNISIEPQIYSPEMRALVERHIELLQLASILAKAFSSLMFIQTLGLIFSLCIVVYQLLM
TTDSGEDINTIHFIIYSCAVILLAFCYCFLGECLINESSEMQVACYFINWYDLPEQYTRSLIFCIARSQKPSYLTAGKFYVFSLETFGVIVKASMA
YLSVLKSII
>BtOr113PSE
LARTVNIFSYILVYILIRLLMILPRFSIVIRYVTNGTDAGKLLPLPTYYSYNVSKTLYFEIMYVVQCINLLIASFCYIGVDNFFGVLILHIFDQLENL
RFHLANMKDSRYGYETSNHVLGATIEDHIRLIXAINVIENISTLLFLVLLINFNICACIYRLLLITASELIYRIMKDNCETNRKVNDSDIILIFLKYS
EGVHNTAYECEWVSLKFTKAKSLIIIMTRSKRPLHLTTGKFPLTMLKXILKISFSYISFLLIMLH
>BtOr114
MSKELKIYRKYASFVKRFLLLSGMCPITKERDVFYRCISIWSIFSSFISLCVVGNFCSQNVQNIALLTASFSLFCAILNTTTKACCFFIYQNKLQQ
ANDILSSMLEQALSETDIRSIAFSWVRTFYRLIYLQFTLMTINSTIHAFKPLITRILYDANNTTNLQYPLPFPASYPWTIDSMLVWQLHYLFDLNI
VWNIISVSTGVDGFFSFCLFRISVMLRLLGFEFEMRFSTDEKDKANKEYEENRKRIFQECVNKHALLLKCRDIVQEAYGPVILLTTITSATSLCT
LIFQVLQVRGQIIDKIIICIVFIFMKLLQTFLYAWPADVILTESDRFRRKVYFSDWYKHKDISFAKGFTLILAQRSIVLKACDLMQVSLDLFVKVL
NTAVSYYFLLETIDKDK
>BtOr115
MDFRDLNPFNIFLSTLSANVLPMSSRETKLPIFFKIYSIIIWLIELTYFIGCVVGILTVSREKALKDGTVNLVIAIEVLVLMIYMHNRKNLLRGLIG
KLNHLIEGNKILRNMIVSALEPMEKPLKIYTVASVGSLALWISLPLIEVFRKNEFRYSDYRVPFVLSKEPFSLNVFVGGVIFQIVGGTYTLIRKIS
LDIYTMYIILLTTAQYKYLRMKFATIFEQKPETLNGSYNDSTRQNVSFRKKRMIQEMRVLTRHYETVVEIAVMLKTLLFLNVGVHYINNVFRF
CFLSFMFSTNMFSEKCLIILYTIGALIQFYILCYCIQELLDASNAVADDVIYEKWYLHDVPLQRALLMIISANKLECKLSNSRHIDLTLSSFMSIL
NQAYSVCLLFLKSRPD

>BtOr116
MGKRVAQLERAILFTKLSVALTCSWPPSPLATKNRLLLFNALWCTAFASSVALFLPLLAAIYEYYKSPIILGKTVSLASAVAQVVIKMIICRLQ
QRRFQMLYFDMENFCKHATKTERMVLERYVHKYKYFHCIYILWSFITTAFVICGPLYSSQTFPTHAIYPFSVKHQPYNSLIFFHQSLVGFQASS
GMGIDTQVALLLRYATARFELLGIQLRNAKTNSEFNVCIQKHIDLLRYTKEIRLSIKYLVLATIATTTAVIFGSLNLIANQPLILKTLYATVVFS
ASVELFMYAWPADGMMRMSERTATSVYGTAWYNRDISVQRKVLRIILRSQKLETIGISGIVPQLSLSHYAKYLYTSLSYFNALRIMVGDPSPL
>BtOr117
MSMFNVNIRQVLYIVELAGMFTCTWPIDPNSSKKQIFFRNIRWSFAILNVVFLTISLVFAIFHFRHDIPILMKTVSEVTALLEVLFDLILCRRNNA
HLQVLTGKVKAFLEVASEHETKAIERYMDRYKQFLSVTAMGYITTAISFSLAPFFSAQELPADGWLPFSTESLGIYCMVYVNQVYCIFQTAFCI
GVDFTIAVLFSFSAARLDILRMKLRHVNSSDILVSCIKEHQEIIGFVEDTKATVETLLFKTNATMGSAVICGAFPLIYNQSLAVMSQYLSFVVSG
CTRLYVISWPADDLKESSVQFAKSITDVQWLGKPKDMKSSMLIMMQRSQKPLLIRMSGLLPPLTLEYFANFITTVSSYFMAMRSMIES
>BtOr118
MLKQITPEKVIHITWLSVVISFCWPLPINSNRNKIFCFKILQICSGISGCLLFMPILYSIYLHLNDIVITSSCMCLLLGVSQNIFQTLICFIKHDSLQR
TVEEMMTYVREAQQYEKKIFYKYIKKCYVFCGGSMACTYVTATCFSLRPIVLPVSLPFDAEYPFPINYTSMYVIIYMHHACLCIQTAAHICVSS
FGALLLWFTVARFECLAAELEKSTDIDALVVCVKKQLHLKRYAEEVFNSFRFLVLYAIGVATFGITLCFLMMLVNVPLFVKLQFIGVCVTVL
MEIYLYAWPADYLKDMSIEAPQSIYNSTWYEQRLGMQKSLLNVLIYRRPLTLSIACIVPELSLRYYCSYLSNAFSIFTTLRVMIHD
>BtOr119
MTKQITPEKVIHITWLSVVITLCWPLPINSDRNKIFCFKILQICSIISACLLFLPMLYFCYVHFDDLIVTSSCVCLTIGVSQNIFQTIVCSVKHVSLQ
RIVEEMIVCIKEAEQYEREIFYKYIKKCYVFCGCSIVCTYVTAVGFSLRPAILPVPFPFDVEYPFPVNYTSVYIIIYMQHVCVCFQSAAQICISSFG
GLLLWFTAARFECLAVNLERSTDTDTLIDCVKKQLHLRRYGEEVLNNFRFMVLYAVGVATFALTLCCIMMIVDVPLFVKIQYIGACVTVLTEI
YLYTWPADYLMDMSMGIPQSAYNSTWYDRRLEMQKNLLNMLTYQKPLVLSIRCVVPELSLRYYCSYLSNAFSIFTTLRVMIQN
>BtOr120
MLRNVTPETAIAFTQFILGLSCCWPLPSTATKSQILCFKILRSVLFLNSLLLFCPLLYAIYVHREDTAMFCKSVSLALAVVHVPLHSTYCFSQHD
RYQRLIEEMKSCCEKGNSYERQIFQRYVDKYAIYYAASAVWFYWSPSIILIGTFFISDPFPTNAEYPFPVDFEPVRSIIFLQQSLVGMQCASLLCT
NILCALLLLFAAARFEILMTEIRAVNSVKSLIKCVKKYYTLKRYAEEVANTARYTTLITLCICGIESVFAGIIFIGRQPFTVKLQFVTVSITVLLAV
FMCAWPADNLMDVSESTMRSVYQSKWYEQPLRIQKFILFMMIPQSPVILRIRCIIPAFSLNYYCSFITNVLSMFTALRVVMLQDEDGILEDI
>BtOr121
MFRNVAPKTAIAFTQFILGLSCCWPLSSTATKFQILCFKILRSVLFLNALLLFCPLLYAIYVHRYDTVMFCKAVSLALAVLQVLLHSFYCFNQY
DRYQRLIEEMKSCCKEANSYERQVFQRYVDKYAVYYAASAAWFYWCGAIVPIGTFFLPDPFPTNAEYPFPVDFEPVRSIIFLQQSLVGMQCSS
LLCTNIFCALLLLFAAARFEILMTEIRAANSVKSLITCVKKYYTRKRYAEEVANTARYTTLITLCICGIESVFAGIIFIGRQPFTLKLQFVTVSVTV
LLAVFMCAWPADNLIDVSENTMRAVYQSKWYEQSLRTQKFILFMMIPQSPVILRIKCFIPAFSLNYYCSFITNVLSMFTALRVVMYQDED

>BtOr122
MPKRMTPEKVIDIIWFSVALTFCWPLPINSSGTRILVHKILQISSVISACMLLLPLLYSIYLHLDDIIIVSECICLFLGVSQAVVQSIICLINHDSLQH
VVEEMIICVKQAREYERDIFSKHIARCSVFYASSIMCIYLAATAFSIGPAILPLSFPSEAEYPFRVNYTPVYVIIYMQQSILSYQCVAHICLSMFGS
LLFWFTAARFQCLAMELKSTADVSTLIVCVEKQLHLRRYAKEVVNNFRFIVLYAIGVSTSALTLCGIILLVDVPPMVKIQFVTVCFTVLTEIYV
YAWSADYMKDMSINVSRSAYDIIWYKQKLEMQKNLLTVLECQEPITLSVSCIIPELSLRYFCSYLSNTFSIFTTLRIVIEDNAE
>BtOr123
MPKRTTPEKAIDIVWFSVALSFCWPLPINSSGTRTLVHKILQISSVISACMLLPPLLYSIYLHLDDIIIVSECICLFMIVSQIVVQTVICFINHDSLQH
VVEEMIICVKQAREYEMEIFSKHIARCSVFYASSMVCIYLTATAFSIGPAALPLSFPSEAEYPFRVNYTPVYVIIYTQQSILSYQCAAHICLSMFG
SLLLWFTAARFQCLAMELKKTSDVSTLIVCVEKQLHLRRYAKEVVDNLRFIVLYAIAVSTSALTLCGIILLVDVPPMVKIQFVTLCLTVLTEIY
VYAWSADYMKDMSINVSRSAYDTIWYKQTLEVQKNLSIVLVYQEPVTLSVSCIIPELSLRYYCSYLSNTFSIFTALRVVIEDNAE
>BtOr124
MSKQITPEDVIEFTRLSVALSFCWPHADDSGRSRGSYKIAQICIVINAFLILLPSLYSIYLYLVDISLHLEALFKCINLTIYTMQLIIQTCICWKKRD
SLQRIIDEMVKCVNEAQKFERDIFMAHIVKYNIIYVGYVVAIYATLTFFIFGPLVLPIPTLVEVEYPFQVNYMPVNFIVYLHHSSVCLAVTAHLC
IGVFGALLMWFAAVRFECLVVEIQKTTNIRMLVVCIKKQLHLRRYAEEVVGCFRFIILYVLGMTTFTITLCGILLIMDSSVTTKVELIDASAFCL
LLTFMYAWPADYLQDASINVSRSVYYMEWYKQPLEIRKYILTVLVHQKPVTLSVGCFMPELNLRFYCSFVSNAFSFCTALRTMVQDH
>BtOr125
MSKQITPEDVIQFTRLSVALSFCWPSADNSDRNRGSYKIAQICIVINAFLILVPSLYSIYLYLGDISFYLDALFKCITLTIYSTQLVIQTCICWKKR
DSLQRIIGEMVKCVNEAQQFEREIFIAHIVKCNVLYSAYVVAVYASLTFFMVGPLVLPIPTLVNVEYPFEVNYIPVNIIIYLHHSSVCLTVTAHL
CIGVVGALLMWFAAARFECLVVEIGKITNIRMLIICIKKELFLRRYAEEVVGCFRFIVLYVLGMTTFTIALCGILMIMDSPITTKMELVNSSVFC
LILTFMYAWPADYLQDASVNVSQSVYDMDWYEQSSEIRKYMLNVLVHQKPVTLSVGCFMPELNLRFFCSFVSNALSFCTALRAMVQDESR
>BtOr126
MSKQITPEGVIRFTWFSVALSFCWPLSDDSSRSQVLGFRILQISAVVNAFLMLVTSIYSFFLNLDNMTALFRCIFQVIYTAQLILQTFICWKKSDS
LQRIIREMMKCINEAKQYEREIFDAYIAKCNIFYGSYVVILYIATLCFMLGTLFLSSVLPLSIEYPFPINYTAVSVVINLHYIILFITCAAHVCMCV
FGALLLWFTAARFECLAVEFEATTNISMLIYCIKKQLYLRRYAEEVIGCFRFMVFYFLSLATFFVTGLGIILVIDTSTITRMEFVSHSSFSLMHVY
MYAWPADLVQDISENASRSAYDMKWYEQSLEMRKYILNVLVYQKPVTLSVGCLMPELTLRFFCSFMSNALSFCTGLRAMVQDEPE
>BtOr127
MLRQVTPEKVIHIVWLSAALSFCWPLPISSSRTRVLGFRILQISAIISACMLLLPMLYSIYLHPDDLVVVFKCMCMSTALCQLIVQTTMCWINQ
DSLQRTVREMMTYVKETQQYEKEIFYKYITRCDILCICTIVCMYATATGFSLGPAILPISFPADAEYPFRVNYTPMNIIIYAHQSILSYQCAAHSC
VSIFGALLLWFTAARFECLAMELEKSTTINTLIVCIKKQLSLRRYAEEVLNNFRIMVLFSIIISTLVLTFGGIILIVNSPLLLKLQFVIVSLTVLTEV
FMYAWPADHLREMSTNVSRSAYNLIWYKQTLEIQKNLLNVLVFQEPITLSVSCVLPELSLRYYCSYLSNAMSIFTALRAAIGDNST

>BtOr128NTE
VFQKNERRTSTVFDISYYKVFKKYLLFLGQFPNQSHWSKKLNLTVMIGSLLTLHYPGFVQIYTSLYAKDVASFLEAVPVITTLTAALIKLLNH
VIYKENFEKMFHIIKKDWELLNDKSQTHILEEITKEGNKIGEIYRTFMLSCMLGFIIIPLTPVILDIISPLNETRHREQMFRVTYFLDENRYFYPIYF
HSLWCAFVITIIAITIDSLYIQIVHHDSALFAICGQALITARKSTDVDTNGTYTEWLRQCLTMHNDALQFFEMLDDSSRRSYFFQILLTMVGMT
VTAVQAVMNLHQPEEALRIGLFLVAQQFHLLIITLPGQVITDYSFELTNDIYRSMWYNMPINDQRILHMMQMRSSKPCKLTAGGIYEMNIENF
GITFKTCVSYFTVLLSLGD
>BtOr129NTE
VFQKNERRTSTVFDISYYKVFKKYLLFLGQFPKQSRWSSKFNVTVMTGSLLTFYFPAFAQIFTSLYEKDLGGMLEGMPVVASVSAVLIKLLNH
EIYKENFEKMFDVIKKDWKLLNDKSQIHILEEITKQGNKIGEIYRTFVLSCMSGFIVIPLYPAFLDIIIPLNETRQRHQMFRLTYFLDENRYFYPIY
FHSLWCSFVTVMIAVTIDSLYIQIVHHDCAIFAICGQNIITATGSTGVRINETYTERFRQCLTMHKNALQLFEMLDDSSRRSFFFQILLTMVGMT
ITAVQAVINLHRPEEALRIGLFLVGQQFHLLIITLPGQVITDHSFELTNDIYRSMWYDMPINVQRILHMMQMRSSKPCKLTAGGIYEMNIENFGI
TFKTCVSYFMVLLSLKD
>BtOr130
MDVFQKTGEQYPNIYDIPYYNTIKKYLRFLGLDPHQKYGLIIVIIMVISMTSGLVPMSIVLYGSLCTKNLDMVLECLPHLGALLTSVVKILNVH
LNRENFKKLFDSITKEWQQLKLSQDLYILEEVTIRGSKMAKLYRNTLLICMVLFLLVPLLPPMLDIVLPLNETRPRQQILNVNYVLFDSDNYFF
YVYLQLSWTSIVVSIIIVTVDSLLMLIVHHNSGLFIVCGHQIQKSTRHLNSFTNEVMSERYTYKQIRNCVIMHNKAIDFYDILDENNRISYMIQIG
LNMIGITTTAVQTVINLDRPGESIRSAVLCGANQFHLFMLSLPGQILIDHCTELTKQLYNSTWYGVPVKVQRMLYMMQIRTRRPCTLTACGLY
EMNIENFGTTFKTCMSYITMIMSLKG
>BtOr131FIX
MDVFQKTGEQYPNIYDIPYYNMIKMYLRFLGLDPHQKYGLIIVAIMVTSMTSVLIPMSIVLYGSLCTKNLDMVLECLPHLGALLTSFVKVLNV
HLNRENFKKLFDSITKEWQQLKLSQDLYILEEVTIRGSKMAKLYRNTLLICMVLFLLIPLISPMLDIVLPLNETRPRQQLVNVNYVLFDSDNYF
FYVYLQLSWASVVVSISIVTVDSLLMLLVHHNSGLFIVCGHQIQKSTRHLNSFTNEVMSERYTYKQIRNCVIMHNKAIDFYDILDENNRISYMI
QIGLNMIGITTTAVQTVINLDRPGESIKSAVLCGANQFHLFMLSLPGQILIDHCTELTKQLYNSTWYGVPVKTQKMLYMMQIRTRSPCTLTAC
GLYQMNIENFGTTFKTCMSYITMIMSLKG
>BtOr132
MDVFQKTGEQYPNIYDIPYYNMIKKYLRFVGLDPHQKNGLITVIIMMTSITTVLIPMSIALYESLYTKNLDVMLECLPHLAAIVTSFVKIMNVY
LNRENFRKLFDSITKEWQQLKLSQDLYILEEVTIRGSKLAKLYRNTLMTFMVLFLFVPLISPMMDIFLPLNETRPRQQLLNVNYVFFDSRNYFF
CVYLQLSWAAIVVSISIVTVDSLLMLIVHHNSGLFIVCGHQIQKKTRHLSSFTNEVMSEHYTYKQIRNCVIMHNKAIDFYDILDENNRISYMIQI
GLTMIGITTTAVQTVINLDRPGISIKSAVLCGANQFHLFMLSLPGQILVDHCTELTKQLYGSTWYGVPVKTQKMLYMMQIRTRSPCTLTACGL
YQMNIENFGTTFKTCISYITMMLSLKG

>BtOr133NTE
VFQITGGPEIFQVPYYSKLERYIQLLGQDPRHKSSTRNIIVVIVVTSIASITIPATTELLVSLHKKDMDGVIECLPHFVTSSASVVKILNMHFNRRS
FNKMFQLVAKQWEQLKLNGELHVLEEVVKQGNRMAHFYRNTLISFMVLFLLVPLVSPILDIVHPINGTRTRQQLLRVNYIFFNDDDYFIYIYL
QLFWSSVVIVFTIISADWLYMLIIHHSSGLFAVCGYRVHKATVNPNNFTGESMSENYTYEKIRSCAIIHNEAIRFSDILKQSSQGSYLIQVGLNM
LGISATAVQTVINLDRPEEAIRSAVFCGACQFHLLLLSLPGQVLLDHCSDLADNIYSSKWYKAPMQIQKVLYVMQIRCKRFCSLTAGGLYEM
NIENFGITFKTCMSYITMMMSVKD
>BtOr134NTE
VRQKTGIPEILHVTYYNKLERYIQLLGQDPRHKSSTRNMIVIIVVTSIASITIPTTIELFASLHKKDMDGVIECLSYFVTSSTSAVKVLNMHFNKR
NFSKMYQLVAKQWEQLKLNNELHVLEKVVMQGNRMAHFYHTLVSFMVLFLLVPLTSPILDIVHPINGTRKRQQLLRVNYIFFNSDDYFFYIY
LQLFWSTVVVVLTIVGADWLYMLIIHHSSGLFAVCGYQIQKATANLNYFSGEKDSENQTYEKFRNCVMMHKEAIQFYNILDESSQSSYLIQV
GLNILSISTAAVQTVVNLDRPEEAIRSAVFCGACQFHLFLLSLPGQVLLDHSSDLADNIYSSKWYKAPVQIQKVLYVMQIRCRRFCSLTAGGL
YEMNIENFGITFKTCISYITMLMSMKD
>BtOr135NTE
VRQKTGNPEILHVTYYNKLERYIQLLGQDPRHKSSTRNMIVTIVVTSIASITIPTTIELFASLHKKDMDGVIECLPHFVASSISAVKVLNMHFNK
RNFSKLFQLVARQWQQLKFNNELHILEEVVMQGNRMAHFYRSTLVSFMVLFLLVPLISPILDIVHPINGTRTRQQLLRVNYIFFNDDDYFFYIY
LQLFWSSIVVVFTIIGADWLYMLIIHHSSGLFAVCGYQIQKATANPNYLTRESIFENATYEKFRNCVIMHKEAIQFYNILNESSQGSYLIQVGLN
ILSISTAAVQTVVKLNRPEEAIRSAVFCGACQFHLFLLSLPGQVLLDHCSDLANNIYSSKWYKAPVQIQKVLYVMQIRCRRFCSLTAGGLYEM
NIENFGITFKTCISYITMLMSLNG
>BtOr136NTE
VLQINEGSEIFHVPYYNKLERYIQLLGQDPRHKSSTRNIIVMIVVISIASITIPATIELLASLHNKDTDGVIECLPHFLASSISAVKVLNMHFNRRN
FHKLFQLVAKQWQELKLNDELHILEETVMQGNRMAQFYHNTLVSFLGLFLLVPLIFPILDIVHPINGTRTRQQLLRVNYIFFNGADYFFYVYV
QLFYAAAVVVFTIISADWLYMLIIHHVSGLFAVCGHRIQKATLNSNNSTGTAVSEKYTYEKIRNCAIMHSEAILFYNILNGSSQGSYLIQVGLN
MLGISVTAVQTVVNLDRPEEAMRSAVFCGASQFHLFLLSLPGQVLLDHCSDLADNIYSSTWYGTPVQIQKVLYVMQIRCKRFCSLTAGGFYE
MNIENFGITFKTCMSYITMLMSVND
>BtOr137NTE
AFQETDRQSPDIFDVPHYKMLEKYLQLLGQDPRQKNGFRNFIVTAVVISIIGNIIPTSLELYTSLCDKDMDAVIDVFPHFMAATISAIKILNIQIN
RQNFNKLLHFVAKQWEQLKLNNELYVLNQTVMQGNKMAQLYRRSLLTALILFLLVPLVSPILDVVLPLNETRPRQQLLKINYIIFDDADYFF
YVYMQLAWGSIMVVVTIISVDSLYILIIHHNSGLFAVCGNQVQRATTNLDTNQIISESYMYKQIRDCVITHNEAIQFYNILNESSRTSYLIQVGL
NMLIISATALQAVINLDKPEEAIRSVVFCGANQFHLFVLSLPGQVLLDHCAELGNNIYGAMWYKTPVQIQKILYMMQIRSGKLCSLTAGGLYE
MNIENFGTFKTCMSYFTMLLSLK

>BtOr138NTE
AFQKVKTRSTDMFDIPYYKMLEKYLQVVGQDPRQRDIFRSIIVTIMVTSITGILVPTSLEVYRSLYDKDMDGVIECMPHLIAAITSIVKILNVHF
NRENFRKLFEFVRKQWEELKLTNDLRVLEEVTIQGSKMAQLYRNTLLIFMVLFLLVPLLFPILDVVLPLNETRPRQQLFRVNYIFFDHEEYFFY
IYLQLAWGSVIVVMIIVTVDSLYILIIHHSSGLFAVCGHQVHKATSSSIIFSTEAMTETYTYEQIKNCVITHDKAIEFYNILNENSRSSYLLQVGL
NMMGISMTAVQTVANLERPAEAVRTGIFLGAEQFHLFVISLPGQVLLDHCSDLANNIYGSTWYKIPVKIQKMLLMMQIRSKKLCTLTAGGLY
EMNMENFAITFKTCMSYFTMLMSLRE
>BtOr139NTE
VFQANSKQDFNVFDISYHKPLKQYLSICGINPYQEDRTSNIIVIAIMCSFMSFFAPTSMQLYDAIRDKDFDNVILNLPHILTVLVSVTKILNIYSN
KTLFRKLFNSLEEDWKLLESKNELQMLDKFTKHGNKVACLYRRTLLIALVIFLSLPLCNPILDIIPLNETRQRNNVFQVHYGILDNEEHFYIVY
VHLSLCAIIIVVTIISVDSLYITIIYHACGLFAVCGSQVQKTAENNFVEKNGINIRNIGYDAFKECVMMHYKCLQLYDALEKCCRNLYLITMVL
NAVILSVTAVEVIVFLDRPAEAIRAIVYLIAQQFHLYMFSLPGQTLLDQSVELANKIYDSDWYKIPTKAQKVFYLMQVRSNKPCILTAAGIYK
MNIESFGITVKACMSYFTMFLSLRE
>BtOr140
MDKATVVDEYMKVVKICTMIIGIWPDQSKSSKLVMRTIIYIISVLSFVTQIANVVHFFNMNVLLNQICFLTALSGVLLKEGLYIIKATEYKMLL
TAIWKDWSTDSHSNEFHIMAEYAKKGALLSWFDCGIGVVCIVIFLQLFLTPLVLDIISPMNETRDLLTIYPAYYYIDDRKYRTIINLHMVYTYIL
GIIVYVGCDTSYMCIVQHACGQLAVAGHRFKNAIFDLSIVNETSAIQDEIHERVLRSIRQHQYAIDYLKTIQSIHSTYLCICIGLVMVTFSVTLVK
VAAQSEISAEFIKDVVFLSSQLTHIFLLTVQGQFVQNANDEVTESIYDALWYNSNNKTKLLFVLALRNCLNPPTLSAAGLIILNLKSFSEILKTS
VSYFTVLKST
>BtOr141FIX
MDKATVVNEYMKVVKICTMMGGIWPDQSKVSKLVMRMIIYIVIVISLVTQMANVVRFYSLHTVVEQVCFFNAMAGALLKQGNYIVNAAEY
KMLLTAIWKDWSTDRLIDEFEIMVEYAKRGAFFSRLYCGLGVFCAICFIQLSLSLYILDIISPNNETRDLIYIYPAYYYIDDRKYRTFISFHMTYT
VISTFFVFVGCDASYIYMVQHACGQLAVAGHRFKNALSDLSIDNEKGGMQDKSYERVLHSIREHQYATNYLKSIQTSHSVYLCICIGMSMAS
FTVSLVKVASQSEISGVLIKDCVFLLSQLIHILLLTVQGQFVLNSNDEIIESIYDASWYNANKKTQLLFVLSIRSCLSPPILSAGGLLDLNLKTFAE
IIKASVSYFTVLKST
>BtOr142FIX
MDKATVVNEYMKVVKICTMMGGIWPDQSKVSKLVMRTIIYIVVVISLVTQMANVVCFYSLQTVVEQVCFFNAVATVLIKQGNYIVNMAEY
KMLLTAIWKDWSTDRLIDESDIMVEYAKRGAFFSRLYCGLGVFCSISFIQLSLSPYILDIISPNNETRDLIYIYPAYYYIDDRKYRMFISVHMTYT
VISTFFVYVGCDASYIYMVQHACGQLAVAGHRFKNALSDLSIDNEKGGMQDKSYERVLHSIREHQYATNYLKTIQTSHSVYLCICIGMSMAS
FSVSLVKVASQSEISGVLIKDSVFLLSQLIHILLLTVQGQFVLNSNDEIIESIYDASWYNANKKTQLLFVLSIRSCLSPPILSAGGLLDLNLKNFAE
IIKASVSYFTVLKST

>BtOr143
MDWQTIEDQCLKANKFFGQLVGVWPNQEKFTKIIIRFVIFIIVITTITAQISRVVVFYSLEVLSDQMPYLDIGFVILIKQYNYILNEKKLKGLLND
IVADRLIERPKEELEILDIYSKKAIFLSCLYQVSISFCAFMFVLLPTIPPILNVVAPLNESRSREFIYPSYYFVDEQQYYYPILVHMISVAVILTSVYI
ACDINLVHVVHHGCALLAISGYICSKCLVTSIYIFRFVSNTYCYYHDNFVALHYSILHICIKIYVDKVDACHVHYFFIILGLIIVTFTSTFVRLSTM
EVGVRYFTFCAFTISQMTHLLFLTIMGQFLINSNDETFQTICEANWYNGSSKAQSLYLLVLRKCLSPPKLTGGGIISLNLESFVLKASFSYYTVF
RSS
>BtOr144
MNRVIIEKRFLKITKIFAILSGIWPGQNKIKFILWALVHITMLSSVIVQVARIIHIGTLEVVLEQSSFIGAIILMIIKHGNYILNAKKLKSLLNDMSE
DWATDRLKEEFAIMTTYAYRGTTLAMFYFVNACICTVLFIQMPWTVRLVHMLKPHNTSSPILYTIPAYYFVEDDRKYYYYIQMYLALSVYV
VLIVFVGCDTCYMVLVQHACALLTVAGYRFKNAINDLSYNGRNPEKGAKEIYKKLRFSIQGHQRAIMFLRNIESTHVTYLFMCMGIIVLCVSI
TMVEIATMDPCWDFYKFIGFLIIQLLHLFCLTMQGQFIINSSDDIYDAIYEAQWYNANPEMQAFYVLALRRSLTPPRLTAGGLIQLNMQSFSEV
MKLCVSYYTVLRTTS
>BtOr145
MDVEAMEKRFLKINRMFGVLTSVWPYQKPFPRLIQRIIVLTILFTSFVTQTAYLILFPSIRGIVTSMPYYILVLGTFVKMGNYFINETKLRSMLN
HIFTDWATIKSKEENDIMITYSQRGLLLTLSYALHALITGILMISWPLVPPILDILMPLNESRQRMFIYPAYYFVDHEKYYNILAIHMVTVMCMT
GFVYCACDANYVYAVQHACGLLAITRYRFRNVTEGVLDHYKNDAKLSKFNYRNVCKSIQAHQHALRYLKLIETNHHTYLFISVGMLIMCIC
VSLLQVANEKNDSWLVQCIFLFAQLFHTLILTGQGQFVINGLDSVFNSIYESPWYTFPAKIRALYILALRSCLSPPLLTAGGLIVLNLRSFAEIIK
AAVSYYTVMKTK
>BtOr146
MNRATFEKRFLRITKIFGKLSGIWPDQNKVKFSLWAMVHITMASFVFVQVARIVHIGTLKVVIEQSSIIGTGIVMVVKHGNYILNAKKLKSLLN
DMSEDWAIDRLKEEVAIMTTYAYRGTTLAMFYLVNACICAFLFLQLPWTVRLMHMIKSHNTSLPMVYAIPAYYFVEDDRKYYYYIQMYLG
LSIYIVVIVFVGCDTCYMVLVQHACGLLTVAGYRFKNAINDLPFNARNPEKEAKEIYKKLCFSIQGHQRAIMFLTKIESAHVIYLLLCMGIIVLC
LSITMVQITTMEICLDFYKFVSFLILQFLHLFCLTMQGQFIINSSDMIYNAIYEASWYNANPKTQALYILALRRSLTPRYLTAGGLIELNMRSFSE
VIKLCVSYYTVLRST
>BtOr147
MNRAALEARFLKITKIFAKLNGVWPDQNKVKFILWAMVYITMGSSIIVQVARVIHIGSLEVVIEQSSLIGAGFLMIIKHGNYVLNAKKLKSLLN
DMSEDWAIDRLKEEFAIMTTYANRGSILAMLYFINACICAFLFLQLPWTARLVHMMKPHNTSSPMLYTIPAYYFVEDDRKYYYYIQMYLGLS
IYVVLIVFIGCDTCYIVLVQHACGLLTVTGYRYKNAINDLSFNTKDSEEMAKETYERVRFSIQGHQRAIMFLEKIESAHVIYLFMCMGIIVLCV
SITMAQIATMEICVDFYKFISFLMIQFLHLFCLTMQGQFIINSSDEIYDAIYEASWYNTNPKTQALYLLALRRSLTPCYLTAGGLIRLNMESFSEV
IKLCVSYYTVLRST

>BtOr148
MNRATLETRFLKITKIFAKLGGIWPDQNKIKFILWAMVHITMGSFVVVQVARVVHIGTLEVVIEQSSLIGATILMIIKHGNYILNAEKLKSLLND
MSEDWAIDRLKQEFSIMTTYANRGSILAMFYFVNACICAFLFLQLPWTMRLVHLIKPHNTSPPMLYTVPAYYFVEDDLKYYYYIQIYLGLAIY
VVLIVFISCDTCYMVLVQHACGLLTVAGYRFKNAINEFSFNTKISEKKVKETYDRVRFSIQGHQRAIMFLEKIESAHVTYLFMCMGIIVLCVSI
TMVQIATMEICMDFYKFTSFLIIQFLHLFYLTMQGQFVINSSDEIYDAIYEASWYKMSTKTQALYILALRRSLTPCYLTAGGLIQLNMQSFSEVI
KLCVSYYTVLRST
>BtOr149FIX
MNKTAIEKRYLKVVKTCSMIAGIWPDQSKFSKLFTRIIIYIISVLSLITQMANVICFFSLNTLIEQVCFFNAIIGALIKQGNYIINAAEYKMLFNAV
WKDWSTDRLSDELDIMIEYAKKGAFFSWLYFGVCICCAISFLQLSLTPIVLDIISPMNETRDLMYIFPAYYYVNDQIYRTSISFHMMCTIISTCL
VYIGCDTSYMYIVQHACGQLAVARHRFENAILDFSIVSETSVVQDKIYERVVHSIRGHQYATNYLRTIQSSHSAFLFITVALGMTTLSVTLVKV
ASQANVSGQFIKDIVFLLAQLIHIFFFLLQGQFVLNANDEFAESIYNTFWYNTNTRTKLLLVLVLRSCSSAPNLSAGGLLVFNLKNFSEILKTSF
SYFTVLKTT
>BtOr150
MDVFYRQYNIYRILLSVLGLWPYHKLIYSKIHRISISVIMLAYIVFQVLSLFKSGITFRGCIVTLSATCPVAIYFMRYVTSVAMFPVTKYIFDNLR
TINTTLKDQLEVQILMKYVDYSTYIISIFLLLCCLWILFATSFVFTPITLDLLLPLNESRRRYFSYFTMFSHDRIEYLDIACVNILVVHTIGMLSLA
GTELMLVLFAHYMCGLFDITSYRMRKTIAGLSSSRQIDPNFRDFRHVVDSHTNTLQLIDYALSNYMVHYLPPCSVFLVSFSVCLHRLTNAITN
ANDQTEIFICSMFVIIHMVILYLCHYSGQILIDRSLDVFKETYNSTWYCMPVEAQKLLLFIMLRSSTESVIDLFGFFAASHVGFSKMLSTSFSYFT
MIYSLQ
>BtOr151
MDVFYRQYNTYRILLNVLGLWPYHKSIYCTIHRISISVIMLAYIVFEVLSLFKSGITFRGCIVTLSTICPITVFFIRYVTFIALFPVTKYIFDNLRTIN
TTLKDQLEVQILMKYVDYSTYILFIFLCSSCSWTLLAASYVFTPITLDLLMPLNESRRRYFSLLTTFSHDRIEYVDMVCVNILIVHTIGLLCLAGT
ELMLAAFAHWMCGMFEITSYRLRKTIADLSLSRQIDSNFWNFRHVVDCHRNTLQLIDYALSNYMVHYLPPCSVFLVSFSVCLHRLSNAVTNV
NDQTEIFISSMFVISHIVLLYLCHYSGQILIDHSLDVFKETYNSTWYCMPVEAQKLLLFIMLRSSTESVIDLFGFFAASHVGFSKMLSTSFSYFTM
IYSLQ
>BtOr152
MDVFYRQCNTYRILLSVVGLWPYHKTIYSTIHRILISVIILAYIVFQVLSLYKSGITFRGCIITLSATCPITAILMRYVSSVAVFPATECLFDYLRTT
DTMLKDQFEIHILMKYVDYSTSIIHIFLCLCCSWTLLAASYVFTPITLDLLMPLNESRKRYFSLLTMFSYDRIEYVDMVYVNILVVYTIGLLCLA
GTDLTFVVFAHYICGMFEITSYRMRKTIAGLSSSRQIDPNFRDFRYVVDHHRNTLHFINYLLSNYTIHYLLPSAVAVVSVSVSLHRLSKMITDA
KDEKEIFMFIFFLISHLAFLYISCYSGQIIIDRSLDVFKESYNSTWYRMPVEAQKLLLFIMLRSSTESIVNIFGFFVASHAGFTTLLSTSFSYFTVIY
SSQ

>BtOr153
MDVFYRQYNTYRILLSVLGLWPYHKSIYSTIHRISISVIMLAYIVFEVLSLFKSGITFRGCIITLSATCPITAILMRYVSSVVVFPATEYLFDYLRT
TETMLKDQYEIHILMKYVDYSTSIIPIFLCLCCSWTLLAASYVFTPIILDLLMPLNESRKRYFSYLTMFSHDRTEYVDIVYMNILVVYTIGLLCV
AGTEQIFAVFAHYMCGMFEITSYRMQKTIAGLSSSRQIDPNFKDFRYVVDNHRKTLHFINYILSNYPIHYLLPSAVAVVSVSVSLHRLSETITD
AKDEKEIFICFFFLIGHLAFLYICCYSGQIIIDRSVNLFKESYNSTWYCMPVEAQKLLLFIMLRSSTESIVNIFGFFVASHAGFTTLLSTSFSYFTVI
YSSQ
>BtOr154
MDVFYRQYNTYRILLSVLGLWPYHKSIYSTIHRILISAIMLAYIVFQVLSLFKAGITFLGCVVTLSATCPIMVFFMRYVSSVATLPVNIYLFDNL
RRMDTMLKDELEVQILMKHVDYSTYIISIFLCIWCSWTLFTASYVFAPITLDLLLPLNESRRRYFSLLTTFSHDRIEYVDIVYVNILVVYTIGLLC
LAGTELMLAVFAHWMCGMFEITSYRLRKTIADLSLSRQIGSNFWNFRHVVDCHRNTLQFIDYVLSNYMLHYIPPCSVIVVSFSVSLHRLSNAV
TNANDQTEIFISGMFVISHIVLLYLCHYSGQILIDRSLDVFKETYNSTWYCMPVEAQKLLLFIMLRSSTESVIDLFGFFAASHVGFSKMLSTSFS
YFTMIHSLQ
>BtOr155
MDVFYRQYNTYRILLSVLGLWPYHKSIYSTIHRISISLIMLIYIVFQVLSLFKSGITFRGCIVTLSAICPVMVIFMRYVTSVAMFPVTIYLFDNLRT
IDIMLKDQLEVQILMKYVDYSTYIISIFLCLCCSWSLFGALYVFTPITLDLLMPMNESRRRYFSYLTMFSHDRIEYVDIVYMNILVVYAIGSLCL
AGTELMLAVFAHYMCGMFEITSYRMQKTIAGLSSSTQIDPNFKDFRYVVDNHRKTLHFINYVLSNYPIHYLLPSAVAVVSVSVSLHRVSKTIT
DSKDEKEIFICIFFLISHLAFLYICCYSGQIIIDRSLDVFKESYNSTWYCMPVKAQKLLLFIMLRSSTESVINIFGFFVASHAGFTTLLSTSFSYFTVI
YSSQ
>BtOr156
MDFLGSKYYNINQQLLLLTGLWPYEKSKLTVILNFYMLAVFMVGLIIQLTNIFTSVYNIDDFISYSAYMIITAMYILKYCTFCCQTKKIKILFEL
VKHDWNDIQHNENELDMLRSRTSTGKLITIVATTIIVMTSILFAISQLLPVIIDIMIPAKESRSIHSANMTFIIIRYKNSSYFALFSLIIVNILGAIVM
TTTETTYVILCQHLVGMFTVAGNKIEHALDCDKFELSPTRKNIVIFLRIVNAVNTHKRCLTFIKGLTNTFVLTYTILLFLGMVSLIVHLIYVYELI
FIAFNIKKALIISVVIILHFGILYFGNFTSQNIRDHHAAIFFYAYSAKWYEAPVWIQKLYLFLMIETTIDIKIILGFYSPSVEGFFRLVNLSVSYLMA
FHSMQRTK
>BtOr157INT
MVTSELNVLEPSQKDDIKKGYYRYTTIHLLITGLWPYKHTKFTRVHRIICASSFFNNCILQLIIYINTSFTSSLFSHDLLDVVISLSFCLLYSTLWH
KQDIIKEIMDIVQQDWRTVRNLEEFNILTKHAKEGIRHSKYFCLFMYSTMMIGTVFYAINFTELLTVPDLHLLPIWMQTIVNQKFLRYISYCIFC
TVMFLANATSIAAESISTMTFQHLRSLFEIASNRIKEAFIYNSLHDSTSSKTFKMTESLKSSIHLHVKCMINYVLLYIIISMIEIVCLFRVNYTTQT
MLDAYNNYNIEWYEAPLHVQKIILFMMLKYSFPLSFNMFGVFSACMEGFSSLAKMTVSYFMVIYSTMR
>BtOr158INT
MVETLAYYSFSKTLLRGLGLWPYHNKRFKVLQNSFISLNLFLFIMAQCFVFKTREFMLKDVSTLIMSFSYFVRYNINWYQLPTAKKMMDKIK
EDLETKNGEVLQIMKDHASIGRRYGIRLSLIIYVSLTFVILYPLLICIQDKSTSLNQICFTRFYIATEYLMLEEKCFLLTVLHSYLSTVIFVTIFLAT
ESLVIMWLQHAVSLCKITRYYVKRATLRNAKNHFDRRTKEDIAKVVFAQSRSVQFFQELQGNNVLTYGVILVSVIISLGINLFCLSQSLLSLSEI
EEEAITYCLFIICQLTFMFYINNMCQQLLDYSNFHTNWYETSLTTQKLVLNIMLRSSKPLLFTISGFYDGTLNGFLLFLKASISYFMMISSIQ

>BtOr159
MVEPAAFYKRSIFLLSWVGLWPYNNTKFRWLHNNIICFIFLSVLFALKTAEFTLYVLSNRLFPIILCSYVVVRYNMFWYQINVVMELMEQVK
YDWKMNQGEPLKILKKYAALGKRYSVVLIALIYLGAFIPITCHVFSYILCIIVSTNITCQYKFPMAMEYFVDEKKYSFFITSHQYFLLILDATILA
GIETLMVTWFQHASSLFAVVRYHIKKAVFEDCVQSHVSRRYLNRNSKRNIEIVINAHRRAMEYAYNLRDRYMFSYGTFLLFVTICLSINLFLL
TQSAFLYRNMEETIIHLVVLSCMLTCMVFFHYMVQFTSDAADNIVTIIYNTNWYEASVPLQKLLLIIMIRSINSFTCTFFGFYSASMEGVSMILR
KSISYFMVMMSMK
>BtOr160FIX
MRSDFQKMDNFAGSHYYSLNRILISAIGLWPFNRTIYTKLLNVLVILLFLLCVFGQLVKLIMAKLSTALRWKIVSYIAYSCIFVVRYIGYLYYIK
DVKWIFQRITYDWNSLKNEAELLIIHEYTKTMKSLSIGFGLILGPMILTFFLKTFSNDILDLLIPMNYTRPRVLPMMIEYFIDQEKFFYYIAIYINV
TISLGALSVFVTEVNSMAVVYHICGLLKIVCYRINHIYVSNIPGVSPAQRSMIIHKQIISIVKLHTTIKHYVDFIQLKVMPLYNVFCVFGLITFILA
LFQFAKAMSIRNDIEEFVASLCTVITSVLYAFTVIHFAQKLTDHSSDIFQQLYNIEWYNLPVVEQKVVLFLMRNSLKDLTFIIGGVIVASHDMFT
MYVRTAFSYFMVVYSFD
>BtOr161
MDSAQYEYLRLNKYLLFTIGIWPYQTTLQRSLVAIVFIPIIVAQVILQGGGMVTAITANDIDSLLEGFAPFMISLMCLAKYINFFYNFKQMKRLL
DIMREDWEIYKKLRIEYDLLSEQYAIGKKITISFVAFLFGLITPFAAMPLLLNAADALGLCNISDDRPLAFRVEHFVDVDKYYFPLLVHSYIGTL
AYTTIVLAINSIIAVYVLHESGLCEILRFKLENFVESDVMDVKLHANKRDDKWYQNARDCVLLHEHIIEFANILEDANTTSYLLQIGFNMICVS
FTQFQAVINVQENAALAFRYVSVTISLLCDLLFVSWPGQQLSDSTERIFEFTTNGKWYLSSINCRKLLMMMLSKSITPLKLTAYKFYTLNLESF
SAVARTSFSYTMVLCSVQ
>BtOr162FT
MIIGMDFFEQPAFRLNKRLLSFVGVWPFQSTLPKLSRQSVVLIPIFVLLTAEIRGLSNAWSVNYSLAIECMPPIIALFLSMSMYLNAIFNTKKMR
DVLLFIKNNHHYYANRPEKLILRYYDVQGRKITLYYALYVYTTVVAYITIPAISLLIDFIIPSNHSEEKSFPIELDYGVDTQQYFYYLFIHSYMTI
AMIANLIASCDTTYMLYAQHGCALFAIVSYELRTVHILDASSLINLKDHRLFENYKNTELLPEEEKKISTKLFLCIKEYQNAIRYCNLVESLFAK
SIFVQLFFNVVCLSIAGTVMKLGNAADTIRFGSFTFAQAAHIFVLCLPGQRLLNHSEEVYNAACEAMWYIFPKKCHNLYKFLLARTLVFSKIT
AFKVATMSMETFLAIIQTAMSYFTVLLSTT
>BtOr163FT
MSRNSERNAITNMDFFEQPAFRLNKRLLSTIGVWPFQRTLTKLSRQGIVLFLNITLFIAEVRGLVNVWSISVDSIIECIPPMVTIFLQVASYFNLIF
NTEKFKDILLFIKNDHNYYMNRPENMILQYYARQGSKIILYYVSYIYITITAFVLIPTVSLINELINHSEERSLPIEIDYGIDIQEYFYYLFIPLYISM
FIVPHVIASCDSTYLLYVHHASALFAIVSYELKTIHIFDTSSLINLKDYNLLEKYKNVELSPDEQKKIFKKLLFCIRRHQNAIRYSNLVESFFTKSI
LAQMFCNVVSLSIGGVETVLNLGNTRNVMRFGALALAQAIHIFILCFPGQTLLNHSEEVYAAACEVVWYIFPKRCHNLYMFLLTRTMVFNKI
TAFKLSVMSMETFLSIIQTAMSYFTVLMSTT

>BtOr164TRA
MNFFEQPGFRLSKRFLSIIGVWPFQSTLSKMLTQGIVYLFCVSLFIAESRALSNEWNVNKNSRIECIPPLMTISFCLSSHLNLIFNKEKVKDVLLFI
KNDHNYYMNRPENKILQFYTAQGNKVVLCYLTYICSTVLAFLMIPTISLVTDLIVPSNHSKEISLPVIADYGVDTQQYFYYVFICICITVFIIGVV
IFTTYSAYMLYVQHACALFAIVSYQLRTMHILDTSSLINLKDRHLLEKYKNVELSQEEKEKIFRKLLLCIKEHKNAIRYSNLLESLFTKFILTQIF
FHIICLSIGGVGIVLNLGNTDEITRFGSLALVQVIHIFILCLPGQRLLNHSEEVYVATCEIVWYMLPKKFHNLYMFLIARTMIFSKITAFKIAVMS
METFLAIIQTTMSYFTMLLSTI

B. terrestris and _A. mellifera_ Odorant Receptor (OR) phylogenetic tree

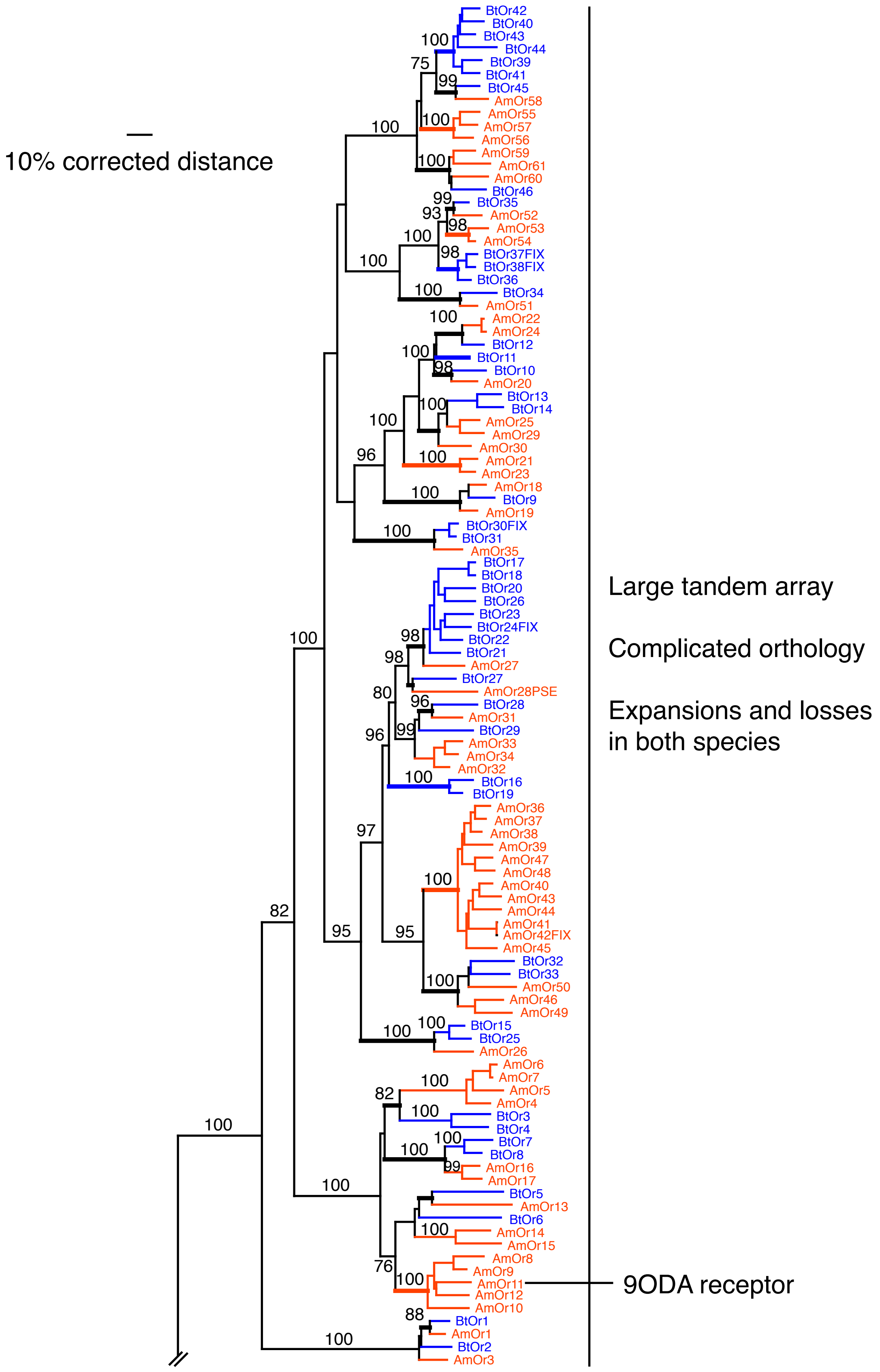

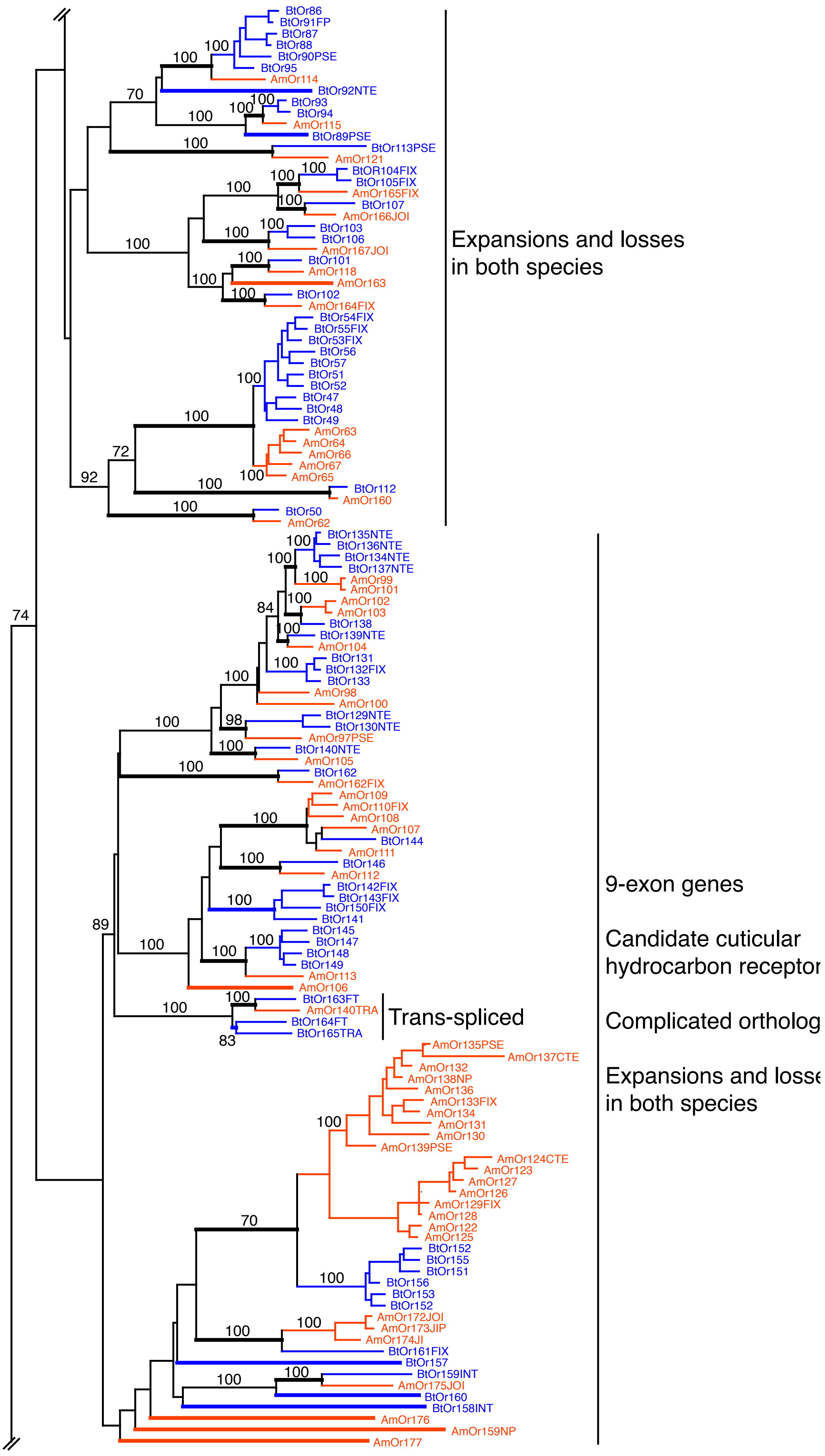
BtOr86
BtOr91FP
BtOr87
BtOr88
BtOr90PSE
BtOr95
AmOr114
BtOr92NTE
BtOr93
BtOr94
AmOr115
BtOr89PSE
BtOr113PSE
AmOr121
BtOR104FIX
BtOr105FIX
AmOr165FIX
BtOr107
AmOr166JOI
BtOr103
BtOr106
AmOr167JOI
BtOr101
AmOr118
AmOr163
BtOr102
AmOr164FIX
BtOr54FIX
BtOr55FIX
BtOr53FIX
BtOr56
BtOr57
BtOr51
BtOr52
BtOr47
BtOr48
BtOr49
AmOr63
AmOr64
AmOr66
AmOr67
AmOr65
BtOr112
AmOr160
BtOr50
AmOr62
Expansions and losses
in both species
BtOr135NTE
BtOr136NTE
BtOr134NTE
BtOr137NTE
AmOr99
AmOr101
AmOr102
AmOr103
BtOr138
BtOr139NTE
AmOr104
BtOr131
BtOr132FIX
BtOr133
AmOr98
AmOr100
BtOr129NTE
BtOr130NTE
AmOr97PSE
BtOr140NTE
AmOr105
BtOr162
AmOr162FIX
AmOr109
AmOr110FIX
AmOr108
AmOr107
BtOr144
AmOr111
BtOr146
AmOr112
BtOr142FIX
BtOr143FIX
BtOr150FIX
BtOr141
BtOr145
BtOr147
BtOr148
BtOr149
AmOr113
AmOr106
BtOr163FT
AmOr140TRA
BtOr164FT
BtOr165TRA
Trans-spliced
AmOr135PSE
AmOr137CTE
AmOr132
AmOr138NP
AmOr136
AmOr133FIX
AmOr134
AmOr131
AmOr130
AmOr139PSE
AmOr124CTE
AmOr123
AmOr127
AmOr126
AmOr129FIX
AmOr128
AmOr122
AmOr125
BtOr152
BtOr155
BtOr151
BtOr156
BtOr153
BtOr152
AmOr172JOI
AmOr173JIP
AmOr174JI
BtOr161FIX
BtOr157
BtOr159INT
AmOr175JOI
BtOr160
BtOr158INT
AmOr176
AmOr159NP
AmOr177
9-exon genes
Candidate cuticular
hydrocarbon receptor
Complicated ortholog
Expansions and losse
in both species

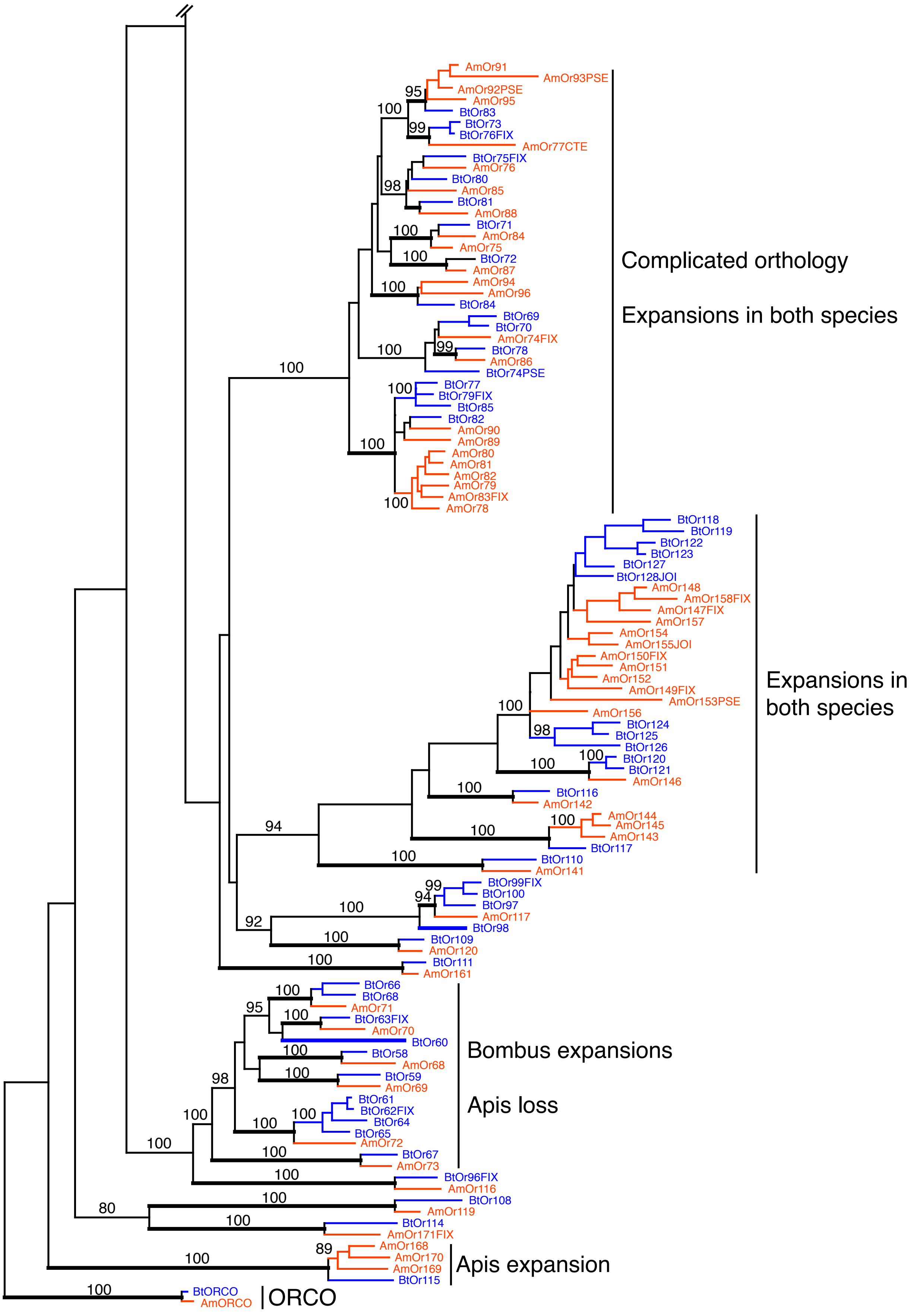
AmOr91
AmOr93PSE
AmOr92PSE
AmOr95
BtOr83
BtOr73
BtOr76FIX
AmOr77CTE
BtOr75FIX
AmOr76
BtOr80
AmOr85
BtOr81
AmOr88
BtOr71
AmOr84
AmOr75
BtOr72
AmOr87
AmOr94
AmOr96
BtOr84
BtOr69
BtOr70
AmOr74FIX
BtOr78
AmOr86
BtOr74PSE
BtOr77
BtOr79FIX
BtOr85
BtOr82
AmOr90
AmOr89
AmOr80
AmOr81
AmOr82
AmOr79
AmOr83FIX
AmOr78
Complicated orthology
Expansions in both species
BtOr118
BtOr119
BtOr122
BtOr123
BtOr127
BtOr128JOI
AmOr148
AmOr158FIX
AmOr147FIX
AmOr157
AmOr154
AmOr155JOI
AmOr150FIX
AmOr151
AmOr152
AmOr149FIX
AmOr153PSE
AmOr156
BtOr124
BtOr125
BtOr126
BtOr120
BtOr121
AmOr146
BtOr116
AmOr142
AmOr144
AmOr145
AmOr143
BtOr117
BtOr110
AmOr141
Expansions in both species
BtOr99FIX
BtOr100
BtOr97
AmOr117
BtOr98
BtOr109
AmOr120
BtOr111
AmOr161
BtOr66
BtOr68
AmOr71
BtOr63FIX
AmOr70
BtOr60
BtOr58
AmOr68
BtOr59
AmOr69
BtOr61
BtOr62FIX
BtOr64
BtOr65
AmOr72
BtOr67
AmOr73
BtOr96FIX
AmOr116
BtOr108
AmOr119
BtOr114
AmOr171FIX
Bombus expansions
Apis loss
AmOr168
AmOr170
AmOr169
BtOr115
Apis expansion
BtORCO
AmORCO
ORCO

Phylogenetic tree of the *A. mellifera* and *B. terrestris* odorant receptors (ORs). This is a corrected distance tree. The ORCO orthologs were declared as the outgroup to root the tree, based on the basal position of this gene in the OR family in analysis of the entire chemoreceptor superfamily in *Drosophila melanogaster* (Robertson et al. 2003). The *A. mellifera* and *B. terrestris* gene/protein names are highlighted in orange and blue, respectively, as are the branches leading to them to emphasize gene lineages. Branches inferred to represent orthology are in double thickness, as are those to genes whose orthologs have been lost. Support for major branches, and all potentially orthologous relationships, is shown above each branch as a percentage of 10,000 bootstrap replications of uncorrected distance analysis. Comments on major gene subfamilies are on the right. Suffixes after the gene/protein names are: PSE – pseudogene; NTE – N-terminus missing; CTE – C-terminus missing; INT – internal sequence missing; FIX – sequence fixed with raw reads; JOI – gene model joined across scaffolds; TRA – trans-spliced gene model; multiple suffixes are abbreviated to single letters.

Gustatory Receptors

Details of *B. terrestris* GR family genes and proteins. Columns are: Gene – the gene and protein name we are assigning (suffixes are PSE – pseudogene, FIX – assembly was repaired; JOI – gene model spans scaffolds; NTE – N-terminus unidentified); Refseq protein number (prefix is XP_00); Scaffold – the genome assembly scaffold ID (format is GroupX.X, amongst 5678 scaffolds in assembly v1.0); Coordinates – the nucleotide range from the first position of the start codon to the last position of the stop codon in the scaffold; Strand – + is forward and - is reverse; Introns – number of introns in the coding region; ESTs – presence of a spliced EST contig in ILLUMINA RNAseq from male (MH) or queen heads (QH), or queen ovary (QO), or 454 RNAseq from multiple life stages (ML); AAs – number of encoded amino acids in the protein; Comments – comments on the OGS gene model, repairs to the genome assembly, and pseudogene status (numbers in parentheses are the number of obvious pseudogenizing mutations). Suffixes after the gene/protein names are: PSE – pseudogene; NTE – N-terminus missing; FIX – sequence fixed with raw reads; JOI – gene model joined across scaffolds.

Gene	Refseq	Scaffold	Coordinates	Strand	Introns	ESTs	AAs	Comments
Gr1	3401991.1	17.2	793766-800484	+	10	-	455	Change first exon
Gr2	3402054.1	17.2	804565-809758	-	8	-	458	Fine as is
Gr3FIX	3401879.1	16.3	2691534-2693608	-	6	-	437	Fix 454 frameshift
Gr4PSE	-	1.4	1673377-1674647	+	3	-	366	Pseudogene (6)
Gr5JOI	-	Un1992, Un16	<1-970, <38365-48937	-	6	-	427	Join across scaffolds
Gr6	3394407.1	3.1	6224665-6226760	+	5	-	419	Multiple changes
Gr7	3397918.1	9.5	1302132-1304956	-	6	MH/ML	422	Needs N-terminus
Gr8	-	13.3	2818921-2820242	-	2	-	391	New gene model
Gr9FIX	-	13.3	2816689-2818550	-	2	-	403	Fix 2 454 frameshifts
Gr10PSE	-	4.5	1496273-1498668	-	5	-	342	Pseudogene (5)
Gr11	-	15.6	909933-911488	-	2	-	426	New gene model
Gr12	3397919.1	9.5	1319950-1322519	-	6	-	451	Part of large model
Gr13	3399602.1	12.1	4239925-4241137	+	3	QH	310	Multiple changes
Gr14FIX	-	Un1870	>1547-492	-	2	-	392	Extend contig
Gr15NTE	3400069.1	13.3	2813014-2814688	-	2	-	362	N-term misassembled
Gr16	3400068.1	13.3	2810767-2812107	-	2	-	392	Part of large model
Gr17PSE	3400068.1	13.3	2808643-2810439	-	2	-	401	Pseudogene (8)
Gr18	3400068.1	13.3	2806510-2807834	-	2	-	392	Part of large model
Gr19	3400067.1	13.3	2804362-2806228	-	2	-	402	Change last exon
Gr20	3400066.1	13.3	2802207-2803528	-	2	-	391	First half of model
Gr21	3400066.1	13.3	2800060-2801895	-	2	-	402	Second half of model
Gr22	3397919.1	9.5	1316550-1318999	-	5	-	451	Part of large model
Gr23	3397919.1	9.5	1313191-1315672	-	5	-	452	Part of large model
Gr24	3397919.1	9.5	1309273-1312305	-	5	-	451	Part of large model
Gr25	3397858.1	9.5	1305830-1308244	-	5	-	447	Multiple changes

Protein sequences of predicted *B. terrestris* (Bt) Gustatory receptors from the table above

>BtGr1
MRSFGVHIVDRDASRRRSPKSSSQFGKLDNLKTEIDTIGMSEPVAASVSAFNPKTDSLHASMRPIIMLAQCFSLFPVSGINNSDASYLRFTWRSP
KFAYCVMSIFGSSMMTIFNILRIVATGVNSTKMTTFVFNGTNLIASLLFLKLSIQWPCLMVTWEKLEKELSYRHRKISRITLATKFSIVTIVVMM
IALVEHGLSIVYGYIQAKECAVHKAEEDILGVYFQMQFPQIFSRIQYSLWKGVLVDICNILSTFSWNFVDLFLILISIALTDQFRQLNSRLNSIRG
KAMPEWWWAEARSEYNHLATLTRQLDSHISVMVLLSFATDLYFICIQLLFSFTPMRGIIEKIYFGFSFGFLLARTTVVSLCAAMIHDESLLPAPI
LYSVSGSSFSTEVMRFLVQVTTDNICLTGMKFFSVTRGLVLTVAGTIVTYELVLVQFNTTQQSEVSNSTVCEVK
>BtGr2
MLKSKGTLCDVPVRSKASSLRVWSSFTYRRNDNNVGELSTNQENSVSVGRPRDGRNYSADFNAIESFHCAIGPVLSIARVFGLFPVAGVRSVS
PSKLQFKTFSFVTFYSAFIASTIFFMTIVSVLHMVKTLNANTIQTRGGIASATVGAVFYGNSLLGSILFFWLSSRWVPLQSEWRAMERCIDSNCI
EPTRLRWKFFFLSSVILMLALIEHILSMFNNVDGYDWNASNSTFRSFLETYTLRSHSFIFDTLNYNFAFGLYIFVVSKLATFTWNFTDLFIMLVA
TALAERYKSLNKKLAVTVTKYRPIFYWRELREHYAILSCVVKKVDEHISPIILLSFANNLYFICLQLLNGLSIAEKSSPLSAVYFFGSFGFLIIRTC
AVTLLTARIHDQSKEALPYLYNCSTSSYGIERLQYQLATDEVALTGLRFFSITRNFMLAVAGAIITYEVVLLQFVDGK
>BtGr3FIX
MELKRIEKRQKAPYNGDLCRAIFPIYYLGKLCGVVPVRFYVHASEGCQARLNIIDLVYSLCALILLLGAEIWGLWRDLKDGWEHSTRLKSRT
AMIATWSDVLGVMSLTVVCIVGSPFRWKSLQLVINKLIEVDRKIGVSSAKKARRFTICLTIYSLVLLWFNSCLDFYSWSRKTKNETLTDKGPIN
YAPLYVMYTVIISTGIQYSVATYNIGQRFVRLNSSLKSMLDTNNSNNDDAIGYFRKFSERAANDIGDKKGWNITPKRQLVLGSYRLSRKMDE
SKTYVSSISELIMVHSLLCDTVSLINAAFGVVLLAVTISCLLHLVITPYFLIVQANEKHEWIFLVVQVGWCIFHISRMLIIVQPSYSTVAEGKKTA
ALVSQLLSCCFEADARRELEIFSLQLLHRPLEFSACGLFSLDRNLITSIAGVITTYLVILVQY
>BtGr4PSE
IEFLLFFKFGGLATISLKNQRTLKKNCSEILVFFTGFKLGVFYNLFXCLVIALIFHIPTNHIYNAEYAFMTIVTQIIEISQAVLGSFVFFSILLYYCIN
ESTFVKIGNYLIYLEGALCRLKQSLNWKHISYVLFFMYSFNFSLFVSVFVTDIISFNXSCFIFESILPAIFVSFCFIQYFSALSLTNAISIXCAIQDFC
RSKFGDIIPNTLFETRHVFVSYTKIYLVVRIRDVHGQFCDITNEISRFYYLPILITLTLISYILLYNVCYLFEPIMVRNVGVDYVIIVNTLLRIISML
YPLGLLTSKATKVTNEIEKAENLVHALLNCTIDRZTKVEILSTVTTYMVILIQFQVGTSVPDAYPCNCTQ
>BtGr5JOI
MFLDKTLKTMKIKVFTWKPSTQHASLLAIIWFNEIFGLRIFESAGRLRYSWSIIYVSTCTILYFTLLYTTIVSNYADWPAYQEISYKFALYVNVI
VAGNCVLFGFIYTGCSKNITARCEQIDNILESFGVEKNYTKIVVYTVRVTSVWGILMISLFVLAWLWWSNGADIMKALCLSLLVSLPVTIICLM
SLTFNIFIRILRDKLHKMNIAMSEVRQLPNENDINIRYKMQPQRAHKLVFVRDYSNQKHYLQHFLQITRQVHFEIVKISRELNHIYGYQLLLEL
AMQFTIIISTLYNVYFEIISSQNVIEIFHSKMAVSAMWWALISSAKVITTNHHCTCFHREAAVTAETLQELATSCSYNSIKDEVQQFSLQLILHPL
HLSAGRCVRLNNEFTTKFFGTITTYLAILVQVSSTPTAMRSLIHTLNR

>BtGr6
MNFSKLVGKELTRYPRREVLPSSKIRVKPFDVESTLAEVTEARKCHARKYHGPDSLLYSAIYPIVSIMKVLGLAPYDFTGDQLVPSNCYLLFSV
VFMGTYSYIIRNVCARFLAVQREKVILNVVETAKVMMNYLIAMYDLTLVIFTRKTFCRIWNAQQDFDERLSQLGYPRKEVKTRIAAWVLLIS
QIVIWTVVNQSGMFAYNETWLFNMSYMCLYVTNATSVYKFFGMTSFLGQRFHRLNQIARDNLPSRVGYKSTSVSKKTIQELHDELMIFGESL
GSLYSWSLFFWLGNLSIHIISNLYLIIDWIIATNGYNLSWPLIFNMSSWLVGYVGQLLALHIVCDYTITEANFMAVTLVEWDARIVERYPHNDT
VLSTLHFLNRRLHFSAGGLFDVKLSLLSSIVGMMSTYLIILLEFPSS
>BtGr7
MFKPKTLRQTIVPSLVMNFLFGMGISDAFSKKPSKLIEYAYTLCVLILIDVVGILMIPYFNKYYIISMLSLSRIAFKLLIYANLWTLSTLIIASRLN
AQKLRALIALIESNDEAMEKIGLPRMYRTLFMYQIKEFVFYGFITVVFVAITSKWHFATSAPTIMKLCLSFVAHVPFIVIYVSSATFGFWVTCLR
LKLHQLNQLLHSMLATMTPESSLYKRFLQMKNDFEDNKFWSFRNDQGSHGNTNKIRSIKQMHLEIIKIVKVVNDTFGMQILLLMTTSVIFTIT
FLYILYRIIWLDLTMDELVKELVSVICWFLVYASQILYVNHVCAKTNLEVTIMGDVICDLYEPSTSNEFRAEIRNFTLQLIQNPVVFTAHGYFN
LDHTFIQAVIGTITTYLIIMIQVGDLTKSRTKTLNNESNSILF
>BtGr8
MYNRSTKFERRSVKKRKSWSSYFGSYISLISPTASINRFLGFLPYKLESSKLVYSKSYSIFSTISIIIYLTCASFGLYEVNVVLENLLTVTTKLHINF
ILCCGLMIFITNYIKSRSMIPVMNHVSNVSRILSSEIFCGVATRILIKDTLFLMPLVSHIPQVMYTNYIYSYICWYTSFGVVILTNLYTNNVYVLN
ACFKHINDSLMKVKVILVNDEPHLLRRVYHMQKNPILLTKLRALKKQHLETSEVVQLLNNTCSMQIEAMVTLMFIDITFNMYKYLILFNEIGK
MRSFSFALMFTILYIVHIIITVSIVETTRSQMAKIGSSLHRILVHTFDEQVTTELELFSLQVLQKGNAFVMKGLVIDATLLAKMACGITTFLLILIQ
FLFVESC
>BtGr9FIX
MMLKAKVKECGKVKTRKTWELFRATDFESLMYPCVCTCWLLGYFPYKYQPPVYSLSKQRFAISTSIMFIFVFLLFFVIYEINIGTRINYSIPELI
HGNMYAFLDGLVIVVMYLLTDARLSVIQNLSRTSCILSTKDFKDLSKLIHTKDILGSLFLAIHIPNCFKHNIFVTVRNLTNIYIMMANFSMDIFYI
NCVCILKDCFKKMEESIRQLKKFRINDHMSTQTFMHHEQISPLVVMKLKNLEEKHLEISDGVELLNHTFMIRITIAAITTFIVITFDIYFYIIYSHS
EFPVNSKFWYKPYVMPAAFYFIKFWMMIWACETATNRAREMKTTLWDVFSATNDPFVKREVEIFSLQIMHTTNIFTAKTFDMNSSFLTKIVG
GIIMYILILFQFLLNYVVCTLHM
>BtGr10PSE
MFVGDINPIFSTGLLLGCTVLVATKNDIKSRRFRHMIFIVFSMILYLISLVVSHVYVFSREDLDLVLFSYSSLFVWNSLWNWKIZSIPSQICNSDR
STKFDDSSRRIKLRIICRITMVTSFSHWAIIVYLTYRIGLRLPVFRGMIYFVMDASVNTQILIFVCILFLIEERFRHLCNMMPFSKXPTDRSIEQFD
LRQIWWLHCSLVNATEMLNSVYAIQLLLWITAMSFNLLSRIYSLKVHKLSVHWKIRKLMLVTDCAWKLILIITMCHMTANPTNHVGQLILSP
YSSVSMKRVFLQESVEAAAYFQLRKARIAAIAGIISVYFPLLLSIFSAVTTYLVILT

>BtGr11
MEEEINVQVESNPTKPENTLRPIMYISWLLGVGVARPKNCPKWITVILRVIHFSVCSVIIAFSAMDFANFGKQFTSKLFEIMYCMNETVCHLAA
YYYVYHIIRHYDKWPELMKMLESLNQNISKGLSINDECIKISQILAIIVALLCGPVSLAVHILYYYFIAPDQIFVSDLFLYYTIAQTLVNSFVFDVI
VYMICHKFRAINEMIKQLDEKLTSYWIALKIRRIRRLHNEICSIIMTVNEIYGIDLLLCSTNAFIMVVAKLFRIYMSAAEQKSGFIFLNNVIWIIYV
FQFILMCWLCTLTHREIDKIGLYISKFILNTQHSTKFNKLSCFGNKEPEQLGIFDNECRDNVALNLNGFGMESLLRSAFEQDCVRNEINDFSLQL
KQYRITFTACDFFEMNNSLLTRFISVITTYLIILVQFYKPEGF
>BtGr12
MNNYKATTKIITILNTIAILRNFIDPVKHKFKFICSWILTFLVAVFLSIIIYIQLSHIKQHMAYNIESVTYMFQYIIHDLGYVSIVIIGQYQSKNAMT
LIERIDKVDEIFKELGIHIEYRSLFRHTMIVGSLWLLNAVIIFALFIKMLIQHPALYTTTFLIFIYCYFTNAQSIVLYDYNTAVYWLGSRFKNINQL
MKTSILKNSQVKTEDSDTESIFEPSHNFRNTFELDNWLPQRTVKVLQVNSTNNTRKLKQRSLSDDKIRLLQRIRFVHLQVCNVSKMVNQIFNA
QILIYTTVILLNCSISIYFLYMQFSRPSNSIHSIELIMMYVLNCVLAALKIPLMSYDCENTTRQANKTIGLLHACPVDEGNAELIDEILQFSWQISY
TQLERTKSVDYILNYGFVRYCLNFVVSNLVIMVQWSQHLIKNDPMIITNKTTTFQTLQENTSIVVQ
>BtGr13
MKLFCPSNIYQESWLLLSITKLLGLYPIKFFRIAFPNYLYVSILVALYWFCHWQFLYYIFDMFDKFRSINDTFLLRYVRVYLNVLSYPIIIISSMY
KCSKVKEAFEQLDTVDESAKFLNIEIDHSLCMKNDIVRITTAIFVVLTCNLMDYYGLLDNDSSFIYLLIWVIDRLPDFVCVIVICSFTVFMYKIEI
RFIQINTILNIITKGKHFISISEMSDMNNVSRYRLLKWLRFELHKTMSLLNEAYSFRFKLMTIIYIGYICLHVCIIYNHTFNTFYAPDVILSFTWSTI
DLIKLVYLIHIYGNLTLEHN
>BtGr14FIX
MFSQPQKLEPRTNKNLKSSPLYFRSYTSLINPSASINRLMGILPYKLESSKLVYSKSYFVFSTISMIIYSICVIFSLFQINFSSVQLPTLANKLHITLV
FLCGPVIFISAYAKNQSMIRVIDGISNVSRILSSETWHKVAARIVIKDILILIPHMCYVPATIFYANFIFGYVCWYTFLGVIALISLYTNNVYVLNA
CFKHINDSLVQVKEILVNDEPHLLRRVYHMQKNPILLTKLRALKKQHLELSEIVQLLNNTCSMQIEAMLTIIFIDITFNMYNYLSMREEMGEAK
SLTLTLGFAIYYIVHVFITVSIVEITRDQMQKTGRDVHRILAHTFDGQVKTELELFSLQVLQKGNTFVMKGLVIDATLLAKMACGITTFLLILIQ
FLFVESC
>BtGr15NTE
GYFPYKYQPPVYSLSKQRFAISTSIMFIFVFLLFFVIYEINIGTRINYSIPELIHGNMYAFLDGLVIVVMYLLTDARLSVIQNLSRTSCILSTKDFKD
LSKLIHTKDILGSLFLAIHIPNCFKHNIFVTVRNLTNIYIMMANFSMDIFYINCVCILKDCFKKMEESIRQLKKFRINDHMSTQTFMHHEQISPLV
VMKLKNLEEKHLEISDGVELLNHTFMIRITIAAITTFIVITFDIYFYIIYSHSEFPVNSKFWYKPYVMPAAFYFIKFWMMIWACETATNRAREMK
TTLWDVFSATNDPFVKREVEIFSLQIMHTTNIFTAKTFDMNSSFLTKIVGGIIMYILILIQFLLNYVVCTLHM
>BtGr16
MFSQPRKLEPRTNKNLKSSSLYFRSYTSLISPGASINRLMGFLPYKLESSKLVYSKSYFVFSTISMIIYSICVILSLFQVNFSSVQLPTLANKLHITL
VFLCGPVIFISAYAKNQSMIRVIDGISNVSRVLSSETWHKVATRIVIKDILILTPYMCYVPVTIFYVNFIFGYICWYTFLGVNALISLYTNNVYVL
NACFKHINDSLVQVKEILVNDEPHLLRRVYHMQKNPILLTKLRALKKQHLEMSEIVQLLNNTCSMQIEAMLTIMFIDITFNMYNYLSMRKEM
GEVKSLTLTLGFAIYYIVHVIITVAIVEITRDQMQKTGCDVHRILVHTFDEQVTTELELFSLQVLQKGNAFVMKGLVIDATLLAKMACGITTFL
LILIQFLFVESC

>BtGr17PSE
MSENTAEVKELKKHRIZTLLRATGFELLMYPCFCIYZFLGYFSYMFETFRXSLSKGCSAFSTFMIFIFVFSLLFTIYKINIGTRINYSVLESIRGNIY
TFLDGVVILIMYLLIGARISLIENRSKTSLIFSTKDFRYSSKAIQTZDILCFLFFAIHIPNCIKHSIFLTSWDFISMYIMMANLSMDMLYINCTCILK
DCFKKMDESIRQLKKLPMNNNIWMQTFVHHRQLSPLLVMKLKNLEKKHLEISDGLELLNNMFMIRVIIAAITFSVVTFDIYFYIISLHSKFSASS
KFWYKPYVTPAAFYFIKFEMMIWTCEAATNRARKMKTTLWDVFSVTNDPFVKREVMLFSLQFVHRAIIFSAQTFNANSSFLTKVIECITIFILT
LFQFLLYYVVCIFHSH
>BtGr18
MFSRPRKLEPRKNQKHKSSSSYFGSYTSLMRPSASFNRLMGFLPYKLESSKFVYSKSYFVFSTISIIIYLICVILSLYQINFSPMRFRELVYQLQFA
LIFLCGSVIFISAYAKNQSMIRAIDGISNVSRILSSETCRKVVKKILMKDISILLPLIFCIPYNLFYVPYIFCYTYWYTFIGAMVLTSLYTNNVYVL
NACFKYINDSLVQVKEILVNDEPHLLRRAYHMQKNPILLTKLRTLKKQHLEMSEVVQLLNNTCSMQIEGILTIMSIFIIFTVYNYLSMHKEVGE
VKSLTLILGYAIYYIVHVIITVAIVEITRDQMQKTGRDVHRILAHTFDEQVTTELELFSLQVLQKGNAFVMNGLVIDATLLTKMACGITTFLLIL
VQFLLVESC
>BtGr19
MMLKNTGKIEELKKRTAWKLLSATDFESLMYPCFFICWLLGYFPYKYEHQVYSLLKGRFAFSTSMMFIFVCSLMIAIYETNIGMKINDSIPKTI
HENMYAFLDGLVIVVMYFLTDARLSVIQNLSRTSCILSIKDFKDLSKMIHTKDILGSLFLAIHIPNCFKHNIFVTVWNLTYIYIMMANFSMDIFY
INCVCILKDCFKKMEESIRQLKKFRINDNMSTQTFMHHEQISSLVVMKLKNLEEKHLEISDGLELLNHTFMIRVIIAAITTFSVVTFDIYFYIISV
HSKFPANIKYWYKPYITPAAFYFIKFGMMIWACEAATNRARKMKTTLWDVFSATNDPFVKREVEIFSLQIMHTTNIFTTKTFDMNSSFLTKIV
GGIIMYILILIQFLLNYVVCTPHM
>BtGr20
MFSRSRKLEPRTNKKLKSSSLYFGSYTSLISPGASINRLMGFLPYKLESSKLVYSKSYFVFSTISMIIYSICVIFSLFQVNFSSVQLPTLANKLHITL
LFLCGPVIFISAYANNHSMVRVIDGISNVSRILSSETWHKVATRIVIKDILILILPMCYIPVTIYTNLIFAYISWYTFLGVTALISLYTNNVYVLNV
CFKHINDSLVQVKEILVNDEPHLLRRVYHMQKNPILLTKLRSLKKQHLEMSEIVQLLNNTCSMQIEAMLTILFIDITFNMYNYLSADKKVGEA
KSLTLILGLVIYYAVHIIITVSIVEITRDQMQKTGRDVHRILVHTFDEQVTTELELFSLQVLQKGKAFVMNGLVIDATLLTKMACGITTFLLILV
QFLLVESC
>BtGr21
MILKSTGITEELKKRRTWKLLSATGFESLMYPCFFICWVLGYFPYKYEHQVYSLSKGRFAFSTSMMFIFVCSLMITIYETNIGMKINDSIPKMIH
ENMYAFLDGLVIVVMYLLTDARLSVIQNLSKTSCILSTKDFKDLSKMIHTKDILGSLFLAIHIPNCFKHNIFVTVWNLTNIYIMMANFSMDIFYI
NCVCILKDCFKKMEESIRQLKKFRINDNMSTQTFMHHEQISPLVVMKLKSLEEKHLEISDGVELLNHTFMIRVIVAAITTFSVVTFDIYFYIISIH
SKFPANIKYWYKPYVVPAAFYFIKFWMMIWACETATNRAREMKTTLWDVFSATNDPFVKREVELFSLQIMHTTNIFTVKTFDMNSSFLAKIV
GGIIMYILILFQFLLNYVVCTLHM

>BtGr22
MKNYETTTSIITVLNYMMALRNFINPKKHRFKFICSWIWNTLLIVLSVFTIHAQIENIKYHMVYNIESVTYIFQYITHVLGYISIVIIGLYQSKNVS
ALIKQIDKVDERLKKLGMKIEYRSLLRHITIVGSFWLLNAVVASAIFIRILIQNSLAPLEAFITFAYYYITNAQSIVLYDYNTAIYWLGSRFKMIN
ELLETFLLEKYKVEAEESNTETIFEPSHNFRNRFELNNWLSEGSLNVSEVSTTGSVSNVKQKILQEKVHILRQIRFLHLQVCKVSKMVNDIFNA
QILIHTITTLLHCTICVYSLYMNFHRQSNFVIDQLDLTLIYMLDSVVGTLKIALMSYDCEYTMGQANKTIGNIHAYPVHEGNAELTDEILHFSW
QISYTQLEETKSVHYILNYGFIRYCLNFVISYLVIMIQWSQHLIKNDPMIITNKTTTSQTLQENTSTVVQ
>BtGr23
MKNYETTTRIITVLNYMMALRNFIDPRKHRFKFICSWIWCMSLIGVLSVFTIHAQIENIKYHMVYNIESVTYVFQYTTHVLGYISIMIIGLYKSK
NVPALIKQIDKVDERLKKLGIEIEYHSLLRHITIVGSFWLLNAVILSAIFIKILIQNLLPPVTIFTIFNYYYITNAQSVVLYDYNTAIYWLGSRFKMI
NELLETFLLEKYKVKAEESNTETIFEPSHNFRNRFELNNWQSEGSLNVSEVSTTSGLSNVKEKILQEKVHILRQIRFVHLQVCKVSRMVNDIFN
AQILIYTITTLLYCTICVYALYMMLHRDPNFIIDRLDLTLIYMLDSVVGTLKIALMSYDCEYTMGQANKTIGNIHAYPVHEGNAELTDEILHFS
WQISYTQLEETKSVHYILNYGFIRYCLNFVISYLVIMIQWSQHLIKKDPMIIANKTTTFQTLQENTSIVVQ
>BtGr24
MKNYETTTRIITVLNYMVALRNFIDPRKHRFKYICSWIWYMLLISVLSVFTIHAEIKHIKYHMMYNIESVTYIFEYTTHVLGYISIMIIGLYKSK
NASTLIKQIDKVDERLKKLGIEIEYRTLHRHITIVGSFWLLNAIVESAIFIKILIRNSVAPLEIFIAFVYYYITNAQSVVLYDYNTAIYWLGSRFKMI
NELLETFLLEKYKVKAEESHTETIFDPSHNFRNRFESNNWQSEGSLNVSEVSTTSGISNVKEKILQEKVHILRQIRFLHLQVCKVSKMVNDIFN
VQILIYAITTLLYCTISVYSLYMIFQRGRSCVVDRLDFILIYMLDSVVGTLKIALMSYDCEYTMGQANKTIGNIHAYPVHEGNAELTDEILHFS
WQISYTQLEKTKSVHYILNYGSVRYCLNFVISYLVIMIQWSQHLIKNDLMIVTNKTTTFQTLQENTSIVA
>BtGr25
MKNYETTTRIITVLNYMVALRNFIDPRKHRSKFICSWIWNTLLIVLTVSTIYPQIVNIKYRMTYNIESVTYVFQYITYVLGYMSIMIIGLYQSKNI
STLIKQIDKVDELLKKLGIEIEYRTLHRHITIVGSFWLLNVVVESAIFIMMLTQYSVTPLEVFITFVYYYITNAQSVVLYDYNTAIYWLGSRFKM
INELLETFLLEKYKVKAEESNTETIFEPSHNFRNRFKLNNWQSEGSLNVSEVSTTSGVSNVKQKILQEKVHILRQIRFLHLQVCKVSKMVNDIF
NAQILIYAITTLLYCTICVYNLYMNLHRGSNSAVDLANLILISILDGVVGILKIALMSYDCEYTMGQANKTIGNIHAYPVHEGNAELTDEILHFS
WQISYTQLEKTKSVHYILNYGFIRYCLNFVISYVVIMVQWSQHLIDNDPMIITNETTTLYENTSIVV

B.s terrestris, A. mellifera and _D. melanogaster_ Gustatory receptor (GR) phylogenetic tree

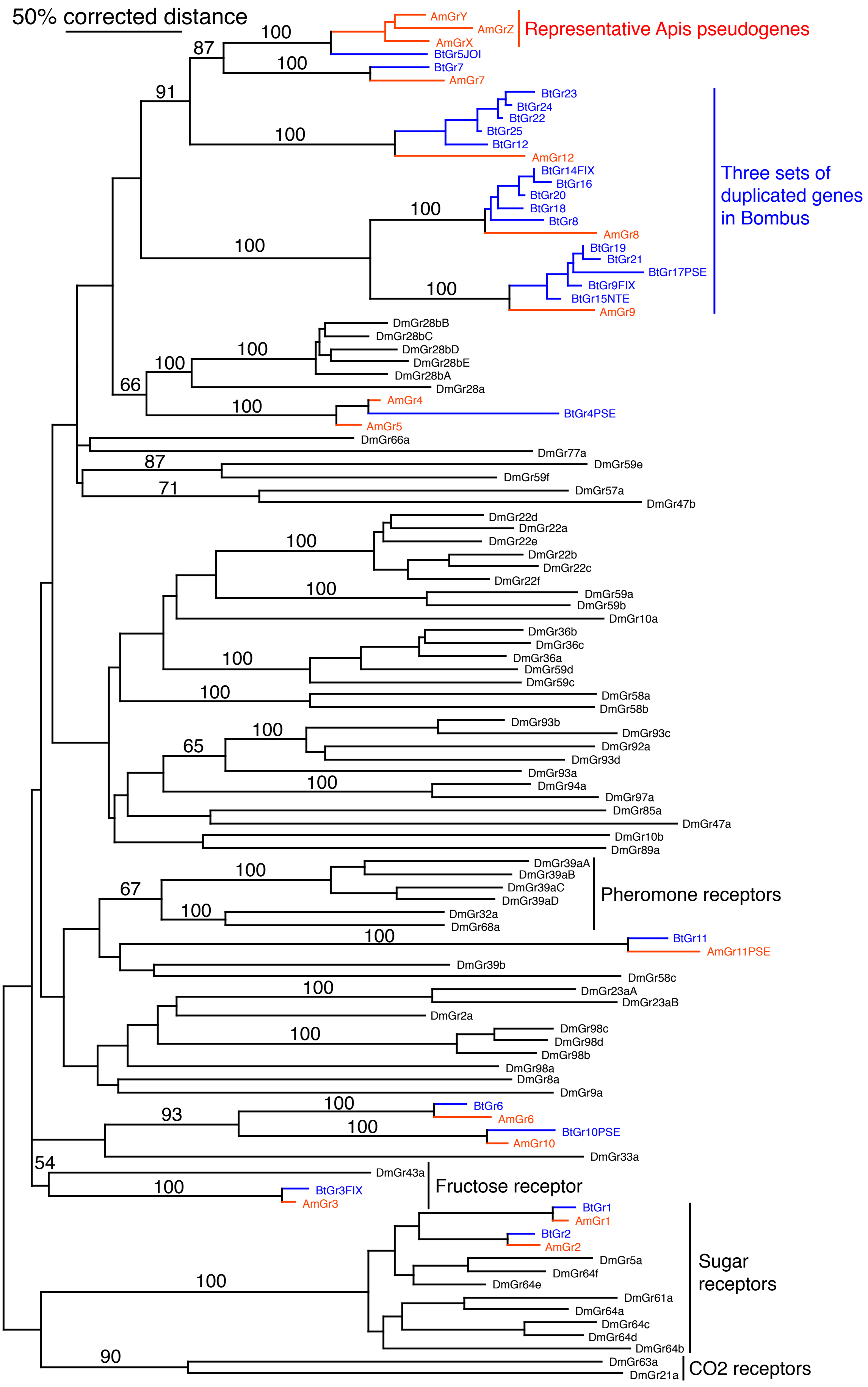

Phylogenetic tree of the *B. terrestris*, *A. mellifera* and *D. melanogaster* GRs. This is a corrected distance tree and was rooted at the midpoint in the absence of a clear outgroup, an approach that clearly indicates the distinctiveness of the sugar and carbon dioxide receptors, as well as their putative distant relatedness. The *B. terrestris* and *A. mellifera* proteins are highlighted in orange and blue, respectively, as are the branches leading to them to emphasize gene lineages. Branches inferred to represent orthology are in double thickness. Bootstrap support levels in percentage of 10,000 replications of uncorrected distance analysis is shown above major branches. Comments on major gene lineages are on the right. Suffixes after the gene/protein names are: PSE – pseudogene; NTE – N-terminus missing; FIX – sequence fixed with raw reads; JOI – gene model joined across scaffolds.

Ionotrophic Receptors

Details of *B. terrestris* IR family genes and proteins. Columns are: Gene – the gene and protein name we are assigning (suffixes are INT – internal exon unidentified); Refseq protein number (prefix is XP_00); Scaffold – the genome assembly scaffold ID (format is GroupX.X, amongst 5678 scaffolds in assembly v1.0); Coordinates – the nucleotide range from the first position of the start codon to the last position of the stop codon in the scaffold; Strand – + is forward and - is reverse; Introns – number of introns in the coding region; AAs – number of encoded amino acids in the protein; Comments – comments on the OGS gene model, repairs to the genome assembly, and pseudogene status (numbers in parentheses are the number of obvious pseudogenizing mutations). INT – internal sequence missing

Gene	Refseq	Scaffold	Coordinates	Strand	Introns	AAs	Comments
IR8a	3402208.1	18.1	1896769-1905248	+	15	894	Fine as is
IR25a.1	3392973.1	1.2	169823-173788	-	4	943	Fine as is
IR25a.2	3392975.1	1.2	190156-192742	+	3	476	Fine as is
IR93a	3401385.1	15.6	3559364-3564574	-	15	862	Change an intron
IR68a	3397511.1	9.1	61620-65919	-	8	693	Fine as is
IR75f.1	3396144.1	6.3	6184986-6188033	+	7	552	Multiple changes
IR75f.2	3396145.1	6.3	6189137-6192524	+	8	597	First half of model
IR75f.3	3396145.1	6.3	6193102-6196198	+	8	655	Second half of model
IR75u	3398764.1	11.1	420735-424186	-	8	705	Multiple changes
IR218	3393505.1	1.9	1558190-1564301	+	11	602	Fine as is
IR328	3395261.1	4.5	7984098-7994889	-	8	807	Fine as is
IR329	3401158.1	15.5	927156-934618	+	9	621	Fine as is
IR330	3393979.1	2.3	2587368-2589967	-	7	610	First half of model
IR331	3393979.1	2.3	2583969-2586708	-	7	659	Second half of model
IR332	3397340.1	8.3	1908987-1913669	-	6	455	Change final exon
IR333INT	3397302.1	8.3	1915646-1919868	-	5	406	Multiple changes
IR334	3395389.1	5.1	7290378-7293889	+	3	697	Fine as is
IR335	-	5.1	7297781-7299913	-	0	710	New gene model
IR336	-	14.8	1307351-1309099	+	0	582	New gene model
IR337	-	2.3	1080706-1084316	+	3	582	New gene model
IR339	-	15.5	2781105-2782973	+	0	623	New gene model

Protein sequences of predicted *B. terrestris* (Bt) ionotropic receptors from the table above, and also corrected *A. mellifera* predictions (Am).

>AmIR8a
MSTTLVAVCALLLTFLGVSICQISMNMLIVIEEPDKSILNILNEALPQAEKNYGNDIISVHISTIEVERSNTDASFKKVCAALFKGISIVLDMTW
TGWDTLRNKANENGIIYKRGDSNINPYIQAIDDLLMLKNATDVALIFEDERELNQSLYYLIGNSILRLIVIDEFTEKTVSKIKSMRPSPSYYAIY
ASTAKMEDYFRTAVQGGLVKRNGIWKLIFTDNNYKDFKYINGDLQLNVSITVLWMKMDVCCRLIGESLCNCPSNVKIFSNYFKRLVGLIVS
LMSELQASGVSVEPKSVKCSSNANQSSNVTIEAFNKNIVAKLGGNDTFEYWPEKGMITYKAEIELKILENGLLEPLATWTRNGKIKEAENKK
ILPAKRFFRIGITPVIKFLPWIIPKIDPATGKVMKNENGNDMWDGYCIDFVKKLSEEMQFDYDLIIPEDRQFGKKLPNGQWNGLIGDLAKGET
DIIVAALTMTSEREEVIDFVAPYFEQSGLLIVMRKPVRKPSLFKFMTVLKVEVWLSIVGALTLTGIMIWILDKYSPYSARNNKQLYPYPCREF
TLKESFWFALTSFTPQGGGEAPKALSSRILVAAYWLFVVLMLATFTANLAAFLTVERMQSPVQSLEQLARQSRINYTVVANSSQHQYFINM
KNAEDKLYTVWKEITLNNTSDEVEYRVWDYPIKEQYGHILQAITQVGPVANSVEGFRKVIESENAEFAFIHDSSEIRYEVTKNCNLTEVGEV
FAEQPYAIAVQQGSHLQEEISRKILDLQKDRYFEMLASKYWNQTQKAQCLNSDDNEGITLESLGGVFIATLFGLALAMITLAGEVFYYRKR
NTETEKSTKDKKRKVKNKIIQNLTKMSLQMKPAPINPFFEKTNNPPRVSHISVYPRNLPFKE
>BtIR8a
MSTTVVAASVFLLASFLGPTISLTPMNMLIVIEEPDKAILSILNDAVPQAEKNFGQNIIAVHISTVQVDRSNVDSSYERVCAALFKGISIILDMT
WTGWDRLRNLADENGIIYKRGDSNINPYIQAIDDLLMLKNATDVSLIFEDERELNQSLYYLIGNSIIRLVVIDEFTEKTVSKIKTMRPSPSYYA
IYASTAKMEDFFRTAIQGGLVTRNGVWNLVFTDNNYETFKYINGEHKLDVSITILSMKKDICCRLIGESDCSCPSDIQVFSHYFKRLVGLIVSL
MSDLQASGISVEPKIGRCPSPNTSPASNLTSEAFNNNILAKLGSNDTFEYWSEKGMITYKAEIQLEEFNGILEPLAIWTRHGKIKEAKKNKIKP
ARRFFRIGTAPSVPWTVPRLDPVTGKIMKDENGNDMWDGYCVDFIKKLSEEMEFDYDLVVPQDHQFGKKLSNGQWDGLIGDLAKGETDM
VVAALTMTSEREEVIDFVAPYFEQSGILIVMRKPVRKPSLFKFMTVLKVEVWLSIVGALTLTGIMIWILDKYSPYSARNNKQLYPYPCREFTL
KESFWFALTSFTPQGGGEAPKALSSRTLVAAYWLFVVLMLATFTANLAAFLTVERMQSPVQSLEQLARQSRINYTVVANSGVHQYFMNM
KNAEDKLYMVWKEITLNSTSDQVEYRVWDYPIKEQYGHILQAITQVGPVVTSREGFQKVIESENAEFAFIHDSSEIKYEVTRNCNLTEVGEV
FAEQPYAIAVQQGSHLQEEISRKILDLQKDRYFEMLASKYWNQSLKAQCQNSDDNEGITLESLGGVFIATLFGLALAMITLAGEVFYYRKR
NTQDKQKDKKKVKTIDNEKLIMQKLASKLQMKPAPTNAFFEKTMNPPRVSHISVYPRNFTFKE
>AmIR25a
MTGAGFNEKYMSAPPRKLRYRVIINDEANKVANQSITTALETIKENYPNHLGNVWSVQVNESDINNTLDRVCNNWDSAVEKGGAEVPDLV
IDTTTAGLAAKISNSFTAALGIPTLSAQYGQVGDLQYWRKLSADQQDYLIQVMPPTDLIPEVIRQLSIQLNITNAAILYDYNFVMDHKYKSLL
LNVPTRHVINETSQQIIEMKRQLLRLRDLDIVNYFILGNENTISIALEAADALNFTDKKYGWFLLTPDINIWPRCECRDISVLFMKPEFDKRNN
SDSVEFSLPKPILLSAFYYDMIRLAVLAMKSALDDGEWPMEPRHITCDEYNNTNTPERKLNFFGKLKDAYKNITPTYAGIKWGSRNGEHQA
KFVMSVHLVTIKDGVVSNTVDSGSWNASISSPLQLTNNDVMNTTAVKSYRVVTVIHPPFVMYNEEKNEYYGFCIDLLNEIKKTVGFQYEIR
ETDDKKYGSLNLDGSWDGMMRELIEKRADIALGSLWVTAERERVVDFTVPYYDLVGLSIMMLKTKTTTSLFKFLTVLENEVWFCILAAYL
FTSVLLWIFDRWSPYSYQNNREKYKNDDEKREFNLRECFWFCMTSLTPQGGGEAPKNLSGRLVAATWWLFGFIIIASYTANLAAFLTVSRL
EIPIETLEDLSKQYKIQYAPVINSSAYIYFKRMANIEWKFYEIWKEMSLNDSLSDVERANLAVWDYPVSDKYTKMLQAMEEAGFPASTEEA
LRRVRRLDSNNEFAYIEDSTTIKYLTMTNCDLIQVGEDFSRKPYAIAVQRGSPLKDQFNNAILILLNKRKLEKLKDKWWKKNPNKKDCDAE
NSQSDGISIHNIGGVFVVIFLGIIFACFTLAFEYWYYRHRNAQDTASIWAAPTLSIPSGPLWPTDTTETIFTLSTSLYGYATDTPYQESIVDDSPL
IPWDIDLISIEDYADEYSNKNKSGDRSDM

>BtIR25a.1
MRLVGFTIAVCGFLQFCGESAVYAQRNVVGNRGSTKTRAVNLYPLTSNDFNFNLFFTPSFSLDLINDEANKVAKSSIIAALETIKEKYPNYLG
EVWSVQVNESDVNDTLDRICKPWDSAVKEGGTRVPDLVIDTTTAGLGAKISNSFTAALGIPTLSAQYGQEGDLLYWRNLNTDQESYLIQV
MPPTDLIPEAIRQLCIQLNITNAAILYDHNFVMDHKYKSLLLNVPTRHVINEASQQVMEMRTQLPRLRDLDIVNYFILGDENTINIALEAAEA
LNFTGKKYGWFLLTPQLNVWPRCECRNMNILFMKPEFNKKSPIESSLSKPVISSAFYYDLIQLGVRAMKSALDDGEWPIEPRHITCDKYDKT
NTPERKVNFFNRLKETYKNMTPTYAGIKWGSKNGEHRANFEMSIHLVDIKDGIVSNTIDSGSWNASISAPLQITNNDVMNTTAVKSYRVVTI
IHPPFVMYNEENGTYYGFCIDLLDEIKDTVGFQYEIRETEDRRYGSLNPNGSWNGMMRELIDKRADIALGSVWVTAERERVVDFTVPYYDL
VGLSIMMLKTKTTSSLFKFLTVLENEVWFCILAAYLFTSVLLWIFDRWSPYSYQNNREKYKDDDEKREFNLRECFWFCMTSLTPQGGGEAP
KNLSGRLVAATWWLFGFIIIASYTANLAAFLTVSRLEIPIETLEDLSKQYKIQYAPVINSSAYIYFKRMAAIEWKFYDIWKEMSLNDSLSDVE
RANLAVWDYPVSDKYTKMLQAMEEAGFPASTEEALRRVRRLDSNNEFAYIEDSTTIKYLTMTNCDLIQVGEDFSRKPYAIAVQQGSPLKD
QFNNAILILLNKRKLEKLKDTWWKKNPDRKDCDAENSQSDGISIQNIGGVFVVIFLGIIFACFTLAFEYWYYRHRTKITKINLNSTTKGKVTQ
VKPLRFNLQPAPTHGFQNSQLRPRF
>BtIR25a.2
MTIGSRPIYRAVTGIAPPYVMYNNTSGAFYGYCIDLLNDIGSLAGFDYTIRKSFDEKYGDLDPKTGLWNGMIYELTSNTSDIAVGPIWITSSR
AQVVDFAVPYQRPSGFTIMMLKKRRQVPFLRFLMILEIDVWIGFAFAFLFTIFLLYSLERYSPFSYRNNRAKYRNEADDRFSSLKECFWFAFT
SITPHGGGEMPKNLSGKIVAGTWWLFGFVMVAAYSANLAAYETLERLERNIESMDDLRKQYRVQYSTVANSSAFRYFLAMRRTEEWFYA
NWRRMALDENTPEAKRSDYAVWDYPLEDKFTKMYLAMEDTGFVRSVEEAVKMVREANRTYEFAFIAEAITVKYLMLTNCDFRQIGEEFG
KKPFAFAVQRDSPLRTKLNDAITKLAIERRLNALEKKWWDENPSRANCPADKDFNAGFDVDNLAVTFLLILLGILLAVLILCLQYCWYCYW
LNRRIKKVGLYATTRYISRGK
>AmIR93a
MISVLLLVWWINYGSSYNNFPSLITSNATMAVIIDKGFFSNKDEYQNATKVIQDLITDAVKKEMNLGSISIRVFRDMNVNFKDYTILLSVATC
YLTWRLHEVAQKEELTHFAITDPDCPRIPDTDGITVPSIVPGEELSQIFLDLRMTDILSWNVINILHDDTFDRDTISRVLKAISNKLPNKRMNLI
SRSIFSLRHGNTGSGRKSSVKKMLNDFHVEQLGHCFLVIATVDMVADVMSVANSLNMVHPGSQWLYVITNSVSGNLINTSFINLLAEGGNV
AFMYNATNLDGFYKIKLKCYMKDLIEALAKALEYSLKNEIELFKRMNEDEFEMIRLTKSKKRAELLKNVRIHLSRNTSASNSVCEQCLLWR
FFSSITWGNFFSHDRNMAHLLDIGTWTPIIGVNLTDVIFPHIVHGFRGINLPIATYHNPPWQIISMSKTGKKLYEGLIFDAINYLSMKLNFTYTV
IMLETSQISRSWNTSQFAKLGEKIKEMTMSTTKKVPLEIIDLVRQKKVLLAACALTVNECGNTTFNYTVPIFVQTYSFLTAKPSQLSRVLLFA
SPFTKETWACLAVSIIIMGPILYLIHKYSPYSTKASGLNSSWQCVWYVYGALLQQGGMYLPQNDSARILIGMWWLVVMVLVATYSGSLVA
FLTFPRMDTSILSVEDLIAHKDSISWGFPNGSFLEMYLQNAEEPKYHVLFSRAERHNDTEEERLVERVKEGKHALIDWRSSLRFLMRKDFLL
TGSCHFSLSMDEFLDEPIAMIIPYGSPYLSVINAELHRMLESGLMNKWITEKMPMKDKCWEAPGSNQMVNKRKVNVTDMQGIFFVLFIGIT
LAFFFLFCEFYCHRRKIAKERKLIHPFVS

>BtIR93a
MISVLLLLWCVNYGDSYNNFPSLITTNATMAVIIDKSFFDNNGDHRNVMGVVHDLIINTVKKEMHIGGIVVRIFRDADVNLWQGYTILLSV
ASCCITWRLHEVARKEELIHLAITDPDCPRIPETDGMSMPVVVPGEELSQIFLDLRMMNILPWNVINILHDDTFDRDTISRVMTAISDKLPNK
QVNLISRSIFTLKHETTRSERKSSVKKTLNDFHVEQLGHCFLVIATVDMIADVMGVARSLKMVHPGSQWLYVITDSASKNMTNMTAFVDLL
AEGGNVAFMYNATNLSNYCEIKLICYVEELIQALAKALEYSLTSEIDLFKSMEEEKFEMIRLTKRERRAELLKNIRIHLSQNAFASEGFCGRC
LLWRFSSSITWGNFFSRGRNMAHLLDIGTWSPGFGVNLTDVIFPHIAHGFRGTNLPIATYHNPPWQIISVSKTGQKLYEGLVFDAINYLGSKL
NFSYTAITPEVTRNSNSWNTSRYAKLGEKIKEMTMSATRKVPKEVIDLVREREVLLGACAITVNENKKDAINFTVPIFVQTYSFLTSRPKQLS
RALLFASPFTKETWACLAVSIIVMGPILYLVHKYSPYSIKTSGLKSSFQCVWYVYGALLQQGGMYLPHCDSARILIGVWWLIVMVVVATYS
GSLVAFLTFPRMDASILTVDDLLARKDGITWSFPNGSFLEMYMQETDEPKYHTLLSRAESHNDTEEEKLVERVKDGKHALIDWRSSLRFLM
RKDLLLTGVCHFSLSMDEFLDEPIAMIIPHDSPYLPVINAELHRMLESGMMNKWITERMPIKDKCWEVPGSNQAVNKRKVNVTDMQGIFFV
LFMGIILAFFFLFCECYCHRRKISKERKLIHPFVS
>AmIR76b
MAINKDNEEGNSIPSQITVTSWNDMPFSGIVQKNGKWIGQGYAFYIFDLISSKLNFTYTIIPPKEHILGNESSGILGLLYEKKVDIAVAFLPMLP
EMRRYCSFSTLLDETKLTAVMKRPQESATSSGLLAPFEKTVWLLVLTSLIFVGPIIYLFANMRAKLWHDPTSENFSLSSCFWFVYSSLLKQGT
NIIALTDSTRMLFATWWIFILILTSFYTANLTAFLTRPQFTLSISSLEDIVHKEYNWITYKGRIVDFLLSQNQQNDLSLLNISKQQGKGIFKYYE
PSRPILELVSTKRLFLEETHYLESLIFKDYVNKTRDHLEHNLRCTYVIMPGNILVTSRAFGFSHGSTIEKHINKMLLRLRETGITQFKKKEDLPL
AEICPVDLRSTERQLRNTDLLLTYKVVIGGYTIATIIFLFELIYACISYRLQNRKRQFINGRSYYVITNPVGDRKLIPIRTPSAFLFQYTT
>BtIR76b
MHTLYLLLLIASFFIQEYTRYVHGEDNVTNNTNNEEMPSQLTVTSWNDMPFSGIVEENGKWVGKGYAFYIFDLLSTKLNFTYTIIPPKEHILG
DKSSGILGLLYEKKVDVAVAFLPVLPEIGQYCTFSAPLDETKLTAVMKRPQESATGSGLLAPFERTVWLLVLVSLIFVGPIIYLFASMRAKLW
HDPSSENFSLPSCFWFVYSSLLKQGTNIVATTDSTRMLFATWWIFILILTSFYTANLTAFLTKPQFTLSISSLQDIVHKGYSWITYKGRTIDFLL
SQNQENDLSLLNVSKLQGKGVFKYYEHSRAILESVTTKRLFLAETHYLQTLIFKDYMNKTRHHLQHNSRCTYVIMPGSILITSRAFGFPHGST
FEKRINRLLLRLIETGIIHRTKKEDLPLAEICPVDLRSSERQLRNTDLLLTYKVVVGGYTIAAIIFLFEFIYAFILYRVQNGKRKACCYLPYCGR
NEKPQNPLNTNLPAQNYMMMLKRSPPAIYQHPDNVLIQKKQQFINGRSYYVVTNPYGDRKLIPIRTPSAFLFQYAA
>AmIR68a
MLLVTLFLQFIVLASSKRVLYKLHQCENNEANLKSLAEEIVEEIIEQTNCIIFITDSTYQNLIDIKNIKGSSNVSKYEILLRDNEQFSRPRRRIQRI
LVDGRTVDCNAYIMLISNGYLTAEFLQYTERERLINTRGLFLLLYDLRLFQLNLYYLWKKIINVVFIRQYNAYKHRSGEISFKERIDLNTVYF
PPRKRRLTATKYIDTWYQGKLRYGTNHFTEKTNNLQKKHLQIAVFEHIPAVTEKSKLYYNKQPNNIIQGLGIEFELIQIISKAMNFKPKYYIQ
QNIPLKQKDIEGSNQTDTGLISKVIEENAAFYLGDLHYTLQNLNYLDLTIPYNIECLTFLTPESLTENSWKLLILPFKQFYTWIALILTLILGSIV
FYFLSLSYKKHISSYKSQNTSIKNETKGLYLFTEIGNSILYTYSMLFQVSLPHLPSPWAVRILIGWWWIYSILVAVAYRASMTATLANPVARV
TIDTLAQLAKSSMEVGGLNEESKNFFLKSSDLSSQEIGNKFIIIKHEDEAIEKVANGSFCYYENSYFLQYARVKRQIFEKEKKRNETANNRSSK
HNLHIMEECIINMPIALGMEKNSPLKPKVDILIRRMIEIGLVKKWLNDVMEWPKIMEIRQEAESEKALVNLHKLKGAFFAIIFGYLLAFMILIG
EILYWKYIVLKDPKFDKYHLDIFYNSNNNSKI

>BtIR68a
MILVTLFLQIILVSGKSVFYKEHQCENSGNNLKSVVEEIVEEIINQRNCIVFVSDSVYRNLVDVKNIKGSSTVLKYEIALRDNEQFLQPRRRVQ
RILIDGKAVNCSAYIILIANGFLAAEFLQYTERERLINTRGLFLLLYDSRLFRSHLHYLWNRIINVVFIRQYNAYKYRSGEKASKERIDLDTVY
FPFRKRKSIVTKYIDTWYKGKLLYGTNHFTEKITNLQEKHLQIAVFEHIPAVTTKSRTYYNKQPNNNTEGLGIEFELMQIISKAMNFKPKYY
MPDNITLEKWGINEDNQTHVGLVGEAIQGKAAFYLGDLHYTLHHLNYFDLTIPYNTECLTFLTPESLTKNSWKLLILPFKFYTWIALVLTLIL
GGVVFYFLSISYKKHISLYKNQMHFQNTSMKKEIKGLYLFTEIENSILYTYGMLFQISLPSLPSSWAVRVLIGWWWIYSILVAVAYRASMTA
TLANPVARVTIDTLEQLAKSSIEVGGWNKENKNFFSMSSDLSSQEIGNKFKLIQEEDKAIEKVANGSFAYYENSYLLQHVRVKRQILEKEQK
ENITTVDISSKHNLHIMEECVINMPIALGLEKNSPLKPRVDTLIRRIIEIGLVEKWLSDVMEWSKIMEIRQEAESEKALVDLHKLQGAFIAIIVG
YILAFMVLIGEILYWKHIVLKDPKFDKYHLDIFYSINNPKI
>AmIR75f.1
MYTTYISMLNSTVELRLDVKKILRAEYHRLGIFLDSRCDRSRYRRILIDATKYSMYDEMHKWLILGSNLSHVLEILNDETFSVSTDVIIAVPS
ADNYILYDVYNPCKDRGGSMNVTYFGMWNFKTGLNVNLNQSKFARRSNLHGMKLKVGIVVNFKPENMSLHDMMLQYSMKSKYGRSKF
LYILLQHMSDIFNFTMKIVQINAQRRFDNSGPIFAAFKKKLIDFSANPVAMKVDRLHNGDIIRPIWPIRSCFMFRTISSTKVKPDQFLKPLSVK
VWYVILAMIGVVTTILIIFLKLENIGTPTEIYGLSVLLTIGALSQQGSAFIPTRCASRIALLQVLFFSLLILNYYSASVVSSRLKNKGEKMNDSLI
NLAKSNLKVAVEPTPYIRSFLQVSDKEVKYFYDNCWTKIPELYKYLPLEEGLNRVAEGSLAYHTMTDSAYPYIEHTFNYRSICELTEVHLFR
AVLAFYARHHSPFTELMKVGLTKIHNVGIQKRELIRWTARKPFCPNNLLIAEPLSIHEAAPIFIFLCISIILSILICIIENMIFCLFRPR
>BtIR75f.1
MYTTYISILNSTDKSRLDVKKVLRAEYNWLGVFLDSRCDRKRHTNVLVEATKYSMYDEMHKWLILGSNLSHVLEILNDDAFTISTDVIIAV
PSANSYILYDVYNPCKDRGGSMNVTRFGTWSNKTGLNVTLNESKFERRSNLHGMKLKVGVVIGFKPENMTFHEMMLQYSMKSKYGRSKF
LYVLLQHLSDIFNFTMEIVQISARRRFDNSGPVFAAFKEKLIDLSASPVAMRIGRLDNGDIIGPVWPIRSCFMFRTISSTKIKPGQFLKPLSVKV
WYVILAMIGIVTTILVILLRLEGVQTSTEIYGLSVLLTIGALSQQGSAFVPTRCASRIAFLQILFVGLLILNYYSASVVSNRLKNRGEKMNDSLI
SLAKSNMKLAVQPTSYIRSFFRVPDKEVRYFYDNRWSKIPESDRYLSLEEGLNRVAEGHLAYHTMIDSAYPYIEQSFTRRSICELTEVHVLRA
VLAFYARHHSPFTELMKVGLIKIQNVGIQKRELKRWAARKPFCPINLLIAEPLSIHEAVPIFVFLCISLVLSILICIVENIVFCLFPAR
>AmIR75f.2
MHPIFFILLQFLSISNAQDHIFIRDYFLYKKVRNVVGFSCGDTIGDFNLLKTLSTTGIYTIIRESSVKIDFQRFMKSETWTVGVVIDLRCHNETA
ASSKYRMYDYSYNWLILGSNYNHSISLLNDTAYNIITDVALAISNRNGFHLYDVFNHCKYRGGILNVTELGTWHIYSGLKIFLTQPLIIRRAN
MHGMRLKISGVIQYRPKDMRLEDYMQDINTRSLDSMHKFVHAMILHIGELFNFSVHASEIIYWDRHSVHGLIFEFLRSNYIDFASNPRIMVSE
RLDYASLIGAAWPIKPCFMLLSTSTNKIKLEIFLKPFTRQTWYVFAAFGIFFIFIMKIIMNREDVGKKEKYSGAIILSVGILAQQGANFLPKRLP
SRIALFQITVHGWIMYNYYSASIVSARLSEPLDMMEDSVTVLADSNLKIAAEAVPYLNYFLYNLNWESDYFRKKRWDPLPESKRYLPIEEGI
RQVGQGILAYHTDPNTAYPYVERMFDSNKICELTEIHLFKQSLMGMYASHNGQFIEIAKIGLTKMFNTGLRNRQIKYWSSRKPECQLDTLST
RSITIYEIAPALILLAFGILVAGIICIMENIIYNRFMK

>BtIR75f.2
MYTVLFVLLQFVLISNAQDHVFIRDYFVYKKVRNVVGFSCGDIVGDFNLLKTLSTTGIFTIIRKPSVKIDFRRFMRSETWTVGVVIDLRCRNE
TASSKYRMYDYSYNWLVLGSDYNNSIPLLNDTAYNIVTDVVLAITNRNGYDLYDVFNHCKYRGGALNVTELGTWHRESGLKVFLTQPLIN
RRANMHGMRLKISGVIQYRPKNMRLEDYMQDINTRSLDSMHKFVHAMILHTGDLFNFSVHASEIIYWDRHSVHGLIFEFLRSNYIDFASNP
RIMVSERLDYATLIGAAWPIRPCFMLLSTSTNKIKLEIFLKPFTRQTWYVFAVFGLFSIFVMKMIMNREDIGKREKYSGAVVLSIGIVSQQGA
NLLPKRLPSRIALFQIIIHSWIMYNYYSASIVSARLSEPLDMMEDSVTVLADSNLKIAAEAVPYLNYFLYKLNWESDYFRKKRWDPLPESKR
YLPIEEGIRQVGQGILAYHTDPNTAYPYVERMFDSNKICELTEIHLFKQSVMGMYASHNGQFIEIAKIGLTKMFNTGLRNRQIKHWSSRKPQ
CQSDTLSTRSITIYETAPALILLAFGMLVAGIICIVENIIYNHKMK
>AmIR75f.3
MYVQNIFYLQLIVASYASNIDIIRDYFIFKNVPRVAGFSCGNIENDYQILKLLNEVGIGVSITQFTSIINIPQFLHTTYWNLGIFVDLECLVSDENI
VKLFYETSTYYMFDHLHQWLILEKNMTHILQLLNDNMFSIITDVTIAISKDNDYILYDVYNHCKNYGGLLNITKLGTWTKNNGLQIILETNK
FSRRWNYHRMKIKVAGLVVKRPKNQSLIDYLQEENLYEHTDNWSKFGYAIMKHIKQLFNFTFELIELNHWEKNDSNGPLIAGLKNGIYDLG
YFPSILTKERFNYADVILQVWPIRTCFMFLTVPSLKVDMDIIFRPFARNVWYMILILIVAIILGLWIIFKLEENDSAYGSTILIIIAALCQQGLPFF
NNQFSSRIAFLQTMIFGLLVYNYYSAAIVSSRLNAPLDKMNDSLYSLVNSRMKLAAYKDIYFNILLHSSVEEVQYFKKYWEKIPEKKRYLSI
QDGLKKMTTAKFAYHADPMNVYPFIERVFDKQMICQLTEVHLLRPSSLGLWSTRHSQFQEITKIGLIRISTSGIRKREVIRWTYRKPYCDKD
KHYVSSITIHETIPILLVLCFGIILSIVICFIENIIFHTIRKKQRQIKESEHRLKKNNKKNVAKNKTMIKIKKLMYKKTSNKKN
>BtIr75f.3
MHFYNFFYLQLIVALYASKIDMIRDYFVFKNVGRVAGFSCGEIENDYRIVKLLNDAGIGVSVSQFASTIDISRFLHSIYWNLGIFLDLQCSISD
KDVMELFYETSTHYMFDHLHQWLILGENMSYTVNLLNESTFSIITDIAIAIPRDDEYILYDVYNHCESCGGLLNVTELGTWSKNDGLRITLRS
NKFSRRWNYHRMKIKIAGVVTAKPKNQDLVEYLREKNAIRTDNWSKFGFAIMEHVGDQFNFTFEVIKLDHWNKNDSSFGPLIAGLRDDIY
HMGYFPSILTVERLNYVNVITQVWPARTCFMFLTVPSSKVDMNLIFRPFSRNVWYMILLLIIVIIFAMWTIFKLEKDAVYSDHGSTILIIAAAL
SQQGLPFISNQSSSRVAFFHIMIFGLLVYNYYSAAIVSSRLNAPLDKMNDSLYSLVKSKMSLAAFKDIYFSVLLQSPAEDVQYFKKHWETIPQ
KKRFLSIEDGMKKMKNLRFAYHAEPDNVYPFIDRNFDKEMICQLTEVHLLHPCSLGLWSTQHSPFQEITKIGILKISTSGIRKREVSRWSYRK
PYCNKDKYHVSSVTIHEAIPVILVLCFGIILSAVICFIENVVFCKLSTKQEQIKKFGSRLKKRNQKNIAPGIKTTVKTKVAIRVTNKKKFFLINK
NFN
>AmIR75u
MLLVPRILRSFAAIAIVRGTLAFDSLDRLLEDAIPILFPPVRMSLHLCRLEREDAIELSKKMSSKRFIHEIHRELDGFTARMHDIWEHRDLYVL
DLNCDYAIPLLRAANETGMLSAPLKWLLLRDRSSVDGDWTDERETLKGMAVYPDSEVIVARKRRNGVVEIRSVYRPSPFHEAIEEDRGNW
TIEHGVRMPNLYPSSQRRRDLRRTPLKSCLVMTDPDTINHLTDYENKHIDPVTKANYPWIMHIANRMNATISFRVTDSWGYRSENGSWSG
MTGMLQRREIDIGGTGTFFIPQRIGVVDYIQLYTHTRACFIFRQPLLSTVSNIFTLPFQRSVWIAIAVFLLLLLVLLCFSTKWEYRRGASANTA
RYWQQFNPAEQTVSDNLMVVLGAIAQQGYSYEPYRVPSRIVTLMLLIAALNLYASYTANIVALLQSTTDSIKTPADLLHSPLKLGAQDVVY
SRHFFKSFQDPIRRAIVDQKIEPKGQNGSWMSVEEGARRVRNELFAFHAERGALYKIMQETYYEEEKCGIMEIDVMNMLDPLLVIQTRSPYL
EIVKNAALLLRETGLKVREDIRLYTNKPKCHERKSFVRIGFTECYFALVALGYGTLLSLVVLAIEVVWHKKIQQIRWDRTSRNNDAMTIAT
WSKAVPFEFEFEEEDEHPMTREGIRSITRLDSMNLRKNVNE

>BtIR75u
MLFFARVLGIVAILAGTRRAVAFDSWFVDLIDDSITVLFPPVRVSAHLCRVDQDGAIELSRRMSRKGLVHDIHREFQGFELRTRHDGWDHRI
LYVVDLDCDYAIPLLRAANSSGQFSAPMRWLLLRDGTIAGNSMAHVDQTFRNMAVYPDSEVIVATKLRVDDNLAEITSVYRPSPYHGVITE
NRGNWTMDGGVRAANLHAASRRRRNLRQTPLKSCLVMTDPDTINHLTDYENKHIDAVTKANYPWMLHIASRTNATIDFRVTNTWGYRQ
ENGSWTGLTGMLQRREIDIGGTGTYLIQQRIGVIDYVQLYTRTKAKFIFRAPLLSTVSNIFTLPFQRSVWIAIGVFLLLVLGLLYFSSKWEYRR
GASAKSAAYWQQFHLDEQTLSDNFMVVLGAFAQQGYSYEPYRVPSRIVTLMLLLAALNLYASYTANIVALLQSTTDSIRTIADLFYSPLKL
GAQDVVHVRHYLKSFQDPLRKAIVEKIEPKGHNSSWLPVEEGVRRVKDELFAFHSELGAFYKIMQETYREEEKCGITEIDILNMLYPLLVIPT
RSPYLEIVKNAALVLAETGLQTREDSRLYTKKPKCQGQTSFVSIGLTECYFALVAVGYGALLSLVVLAVEHLWHRKGSLIDLRAVKRFASD
ALPFPGETANDNATPRSEADPFEFIEESRRFPSKETENGSVSEDVDDRRRQGTRSTIGDSVNSVQ
>AmIR218
MRIYNIFIHSLVSSVIGFSEDLLLAMNVKSWITDHNFTEIINLSFSISKCCNVLLSDSTEDSEIFFDRFRTIYPYDYFLKRNTNEYEEECDGYLLL
GSRDHEIMEFIRKIPSLLWKTEILIIVDNDISIDSNILDNSIYGIASVNIVSTSGIWKLSDNYLSPRVFYKLDRYEEIKIDENINFRGQELQVCSIYN
PPMAYMNHTIRKTINGFEAEVYAMDNDLDWDGIEMRLFLIMAEKLNFTWTIRKPEGNYTYGKRFNETYWIGGIIQMLVDQKVDIAFASIW
MTLDQNKFVTLSMPWYDVYLHFLVPRPHRTTSFWALKKPFSKKIWCLLLSALLLHSLYTYVRSWIDSKFPKRYRNFLITFIDLIGYLLSSSVP
KTAVPNRVQILLWQTVGWLIIAAYCSSLAARLATWEYESRIDTFKQFVEANLSWGKSGQPPPFDDYFDLSDPHSAQLRNRYRQIENNTQLE
KFIMEGNYAILGKIIETCFVPTDYITTESLKNYRLMRESLGHFYASFAIQPRLLKPINKMILWLKESGIVIWHLRDVIRRRGNYNFREVFIERDR
YDGSVQVLGLTPLGAGFSLLLVGFFIATLVFCLELKHFVGKMCDLRILINRKHDS
>BtIR218
MADKRNIFLSKGKSWIADEYFTKLIKLSFTSSGCCNVFLSDTTEDSETLFNQFRSIYPSNYLLKSDTHDCHEFFLLGSSEEGIMESIKKIPLLQW
NTEILIIVNNDISNDSCLLNNSVYGIANINIVSMSGMWILSKDYIKPRVFTKVDRYKEIQINDKVNFHGRELQVCSDYIPPMTYLNHTIQKTIN
GIQAEVYTMDSDLDWDGIEMRLFLIMAEKLNFTWTLRKPEGNYTYGKRINDTYWMGGIIQMIRDQKVDLAFASIWLILDQERFVQLSEPW
YQLHIHFLVPRPRRTTDFLALKRPFSEEVWFLLLSVLLLHSFYAYVRTWIDPKFPKRYRNFLIILTDLIGCLLSMSVPKSVGTTMNKLQILFWQ
TAGWLIIAAYCSSLAARLASSDYEDRIDTIEQFVQANLQWGKMGQPPPFADFFDLTNPHAAQLPNRYRQIQNSTQLKQFITQGNYAIPGKIL
DTYFFPTDYISNEDLKNYRLMRKPVGHFYAAFAVQPWLLKPINRIILWLKETGIVIWHLRDVIRRRDNYNLREVFVEHDRYDGHVQVLGLM
PLGAGFFFLLVGMSIATFVFYLELKRTAKTASVHDCLRHMDKIHDA
>AmIR328INT
MKTKFFILPFYMVTSTCILPSPLTLQLILEFAKWKTWDQIVLFENLTSLDCVFTYTRPLIACLSDEGIGMSIQSATNPNIPDALTIQKHRIGSIVL
LDGLNLTAPDNILYVASQKFQFNYYISWLMITMRNMDTTIDTVLRHLNIGIDSDVIVATPSTTYEMIQKLSRIYWNNNENASINLNYAVLEN
GTLLFYFIHVYKIRYSDNTSLVTNFLGSWNLNSWSLNNPISVKLRNEFKGQPIIFGVLNETIDGQMDINEEEMNDIAPLLDFANFVANSVNASI
ELVSHEKLGTLNNKIWSNLLGDVVSGEVDIGLGYITVNKERQAEMSFSHPLIRYMRNIYYHPLESGTMRDIFRQPFNNYLLSCVAFTYFMILI
SMGLIIYTAKTVLHYEEAKRVGIGEAALWCISIMCMQGSPWTPCNPSGKTILLFTLIFALVMYNAYAGFITSILSVQASGIKSITDILSHDFKLG
YSITDDEYIRNVNDSNLRQLYIRAYNSRESKLDTSSGLMKAVKGHYGFFVSATLARRTLRSTLIQERCTLKELSLPQTFTMVALPMANSCPY
KKIINLNILKIRERGVLNRITEQMLPEMPRCKSSTTFHSARLADVYSAFFILIAGGVVAISIWIAERIWHKRRQMKETIVQRVRQRRLIPSHLPH
LPRFKSFSHFPFQSQLDFHNDARNEFSHDYCKHNSSINNDCFASKLSISTKHSKEKISLKKRKEKNDYVDSDEDIDRSRYESKFFTWKRYSNL
KRTNWNQFLEFPKSKLDRNKIDRLKDNIVFPFHH

>BtIR328
MRVEFFMAATFYMVTSTCILPSPLILQLILEFAKWKDWDQVVLFQDLTSFDCVFTYTRPLIACLSDEGIAVSIQSAANPNIPDVLVIQKHRIGS
IVLLDGLNLTSPDNILHVASQKFQFNYYVSWLMITMRNTDATIDTVLRHLNIGIDSDVIVATPSSTYEVIQKMSRTYWNRTCVSLQHYGTSF
KFGEPPVRRFDENASLNLNYAVLENKTVLYHFVHVYKIRYSDNTSLVTNFLGSWYLDSWSLNSPISVKLRNEFKGQPIVFGVLNGTIDGQM
DVNEEETNDIAPFLDFAKFVASSVNASIELVPHEKLGTLNNKMWNNLLGDVVTGDVDIGLGYITVNKERQAEMSFSHPLIRYMRNVYYHPL
ETGTMRDIFRQPFNNCLLSCVASTYFAILVAMGLIIYTAKTILHNEEAKRVGIGEAALWCISIMCMQGTPWTPRNPSGKTILLFSLMFALVTY
NAYAGFITSILSVQASGIKSITDILSHNFKLGYSITDDEYIRNVNDSNLRQLYIRAYNNRESKLDTTSGLTKAVKGHYGFFVSATLARRALRST
LIQERCTLKELPLPQTFTMVALPMANSCPYKKIINLNILKIRERGVLNRITEQMLPEMPRCKSPTTFHSARLADVYSAFFILIAGGVSAVSIGIV
ERIWHKRRQMKETIVRGMRQHRLMPSHLPHLPHLPRFSHFPHLSHLHFRDDRRNDFSLDRDSRSNLTINNECVAANPSDSTQESREQITQRN
YNQERYVHSDEDINRPRFEPKFFNWRRRSNFKRTNWSPFSGLPRTRLGRSQKSRSKDNTVFPFHQ
>AmIR329
MTNFQFHTTLLLLCVSSCVYLDILPVKAESILIRPYYVYENLIKGVHDYFNNTCIILFHGSSKLIEEEGLQEMDGLLTLQTRFSKYLHIRTVIMD
FHMFKNRVGSFWITMAYPTWLLFLRDETRFEEFLSDVYIPFDCVFMVAQRDRQGSEIIQDVYRIGKEDYLRSMTFGTWNSSHGFQGPLLGL
YQRRHDLHGHNIRVVAINDPPISRISRDKAGQPFGITGFFGEVIQLLQEGMNCTFSYKEAETWGVQLLNGSWTGSIRMLIEDETDLIATEMM
MSSDRLDVLKFTTPIYTSKCRVFIKRPDTTAVKWNAYLAPFAWNIWNVIALTIVIVTLTIAGIDAFSRRIEWLSSIGGGTPRPSNDLFEILFHVF
GVFCGQGMDQSLLDPTRMVHLSVHLTAVVVIAAYSAALISYLAIKTFVMPFTTMKGLLEDGGYRFAVVANSAEYSFFQNTTDTVLSIMFEE
LLTRETDLPINYLDGLQRVCREKNYAFMTLDNMASVLQGKVDCVLEPLDVIMQVTIAMAVPFQSPYRGIIDTNILLLRDSGILQRLLKIEWS
NDEIRRIKKGWTSVELEDAAPLLLFLIAVYTIVCLLLLVERLIDRNRERRSKTN
>BtIR329
MLRFRREFCATLLLLCVSRSTRLCASLADAEPILIRPLKVYESLIKGVHDHFNNTCVILFHGTMNVIEKKGLEDIDGLLALQRYLSGSLNIRTV
IMDFSMFKTRVGLPRNVSKWITMSYPTWLLFLRDSTKFDEFLSNVYVPFNCVLMVTQRDSKGTGEIVRDVYQISKEDNLRSMNFGEWNAR
EGFQGPQLGLYQRRNDLNGRNIRVVSVHDPPVSRIIRDKAGQPSRIGGFFGEVIQLLKEGMNCTFTYMEASSWGTRLPNGTWTGSIRMLVE
DKADLAATELMMSSDRLEAIKFTTPVYSTKCRAYIRRPDTTAVKWNTYLAPFAFNVWNAIGLTVVVVALTITGIDALSRKVNWFPIDLRSN
SRSTLSEILFFVFGAFCGQGMEPSPLDPTRLVHLNVHLTGVVVLAAYSAALISFLAIKTFVMPFTTMEGLLKDGTYRFAVVGDSADYSFFQN
TSDTVLTVMFEELLARELDLPVNYLDGLNRVCQEKKYAFMTLDNMATVLQGKVECAVEPLDAIMQTTIAMAVPIHSPYRGIIDTNILLLRD
SGILQRLLKLEWSSQVRWAKSGWSSVELEDAVPLLLFLISSYLVTCLVLLVERIVCRNREQRSRSDQRFTGN
>AmIR330FIX
MKLILYLNFLLVSFYLTRANISGALYVPLIKWIRNYYSTSSICFLSSFKESKHFDNFQIKHLSYTWSRLLQREQIATLTTSFKNIHTLKNNHLRS
LIIVIIPGPYILFEFSKITKSLKMSSFSWFIVFIPNIEDIKKYCYEPPGNIFNLLYDTQMLVMCQNDPVLREWYSIDENKTEILDIVKWYPEKQEL
GFPAAVDLLTNLSLYERRKNLKGKVLRAVIVKNSLLFSIKNDKMQGYFSLAITELENALNFTLDIVAEKREFGSYNMTTKHWTGAFSLVAS
GEVDIGISDFSMTNIRLNFVDYTIPIITTKRCLFLKQPEIFTVKWFAYYKVYNFMLWISLIVTMIISLFVLAFIRSRIESNNMIHEIFHEFIRIWGIF
CQQGISGELPRNLSLKLAYFTVLMTALVVFTAYSASMISFVTACIRNVPFHTVEEFIDDSSYSLIMLKGSSDYDMLIYSKDSTSKYLMSKLLPI
DKLPMDVQSGFKIICDNSKIGSYTGYSKKIQKITHSWPIPCEVYCIDIGPIDSLSLILSKDNQFTSIINYYLQKLLNSGILNRFKNEETFVEESKFD
PVAIYSVASIIIIFFGSALLAVVILFIEIYYKKIKSKFF

>BtIR330
MKIVIYLNVISIFFHLTGTEMYNNETLYIPLIKWIRSYYSASAVFLLHSSNENKNFDDLKLTYLSHTWSRLLHREQIATLSSTFKNIHALDRNNI
LRPLAIVFISGSDAVSEFSKYSKSFRISHFAWFVIFVPTAMNAESYCYNPPGNPFNLLYDTEMLVMCPGDPVLREWYSVDGNNTVISDLVKW
YPKKQLKSLTAVVDLLSNLTLHERRNDLKGKVLRAVTIKNSIFATKDDKFEGYFSRAVNELEKYLNFTLDIVTEELEFGSFNVTTKCWNGA
FRLVASGEVDIGLSDFSMTNIRLDYVDYTVPIITTRDCLYFKQPEMSAVKWLAYYKAYSFALWISVLMTIIIAQFTLAFIRSRIESTDLTVELY
HEFIRIWGIFCQQGITEEFPRYTSLKLAYFTVLVTGIVLFAAYSASMISFVTAYVHNLPFRTIEEFVNDGTYDVILNKDSADYDIFALSKDPVSV
HMMAKLRPIHTLPITIEDGFQKICDDPTLVYYSGYGKKMQGIANFHIPCDIVCIDSGRVDSLSLILPKNSQFTSILNYYVQKLLNTGLLNRFKN
EVSFTNKNKFQPVGFYSVASVLIIFFGGVLLALVILIAEMYCNRYKNSQ
>AmIR331
MARVTCYAIILLFLLPVPFSFSMDVNDALKYILLIKDVHKYYKTTCIIIVHSDSDTELDQTSLAYIWSRAFSQQGILTMIASFSELSHERKFQDY
TTRPLCVIILATKENLIEFSTITRYIDVSFFVWLVMFLPYQENSMRNFCQNPIGNPFNLIFNTEMLVLCYDHPVLREWYALRDDKTRVFDLAT
WKSGQGLNVTTRNTLYARRNNLFGQTMRISIVNESLFIEMKNGVLSLFWGAVVRELSKSMNFKIEVTSIMSEYGSWNEEEKIWEGVIGELA
SNKSDMGVAEFSMTSHRLDAVDFSLPLIMSHKRIYFKKPDSSSVHWSAYLKTFNIDIWMVIVCLIVSVPIFLTVIKTRGRVKMNVLTDNYMH
VWGIYCQQGLSEFPTETSMRLGFLSIFVSSLIILSAYSASLTSFLTVSTVSLPFSTMEEFVDHGSYGLITFRNSADYEIITSNNRSITLKLKKLIKE
KHELPLTAQDGFVQVCNEKVGFYLTKAIMNAMTTIPCKTVYIKASGIDSLALILTKRSPYTGLVNYFIQRYKDNGVMNKLKRMYFVKKQF
YDTGYNSVTLSGIAPILSVLAGGILFSCLILIFEKAYHQFCSCESKRTYQFVFWQKFFDREIGTKEGRRDQNLAFKFKKNQLSEILRKNDFLT
>BtIR331
MTRMTRYAIIILLLLSVSAFSVDINDANKYVLLIKDVHNYYKTSCIIIVCSNSHTNFNQTSLAYIWLRAFSQRGILTMILSFSQLSREARKFQDY
LTRPLYVVALATKETMDEFSTATRQIDMSLSVWLLMFLPYRENPMRDICQNPVGNPFNLMFNTEMLVLCYDLPLLTEWYALRDNQTRVFN
LATWQTGQGLKLKTTSSLYARRNNTFGQTMRISIVQESPFVEIKNGILSHFFGRLIRELSKSMNFKIEVTSSMLIYGDLNKDDNTWTGVVGE
VASDRADFGVAEFSMTNHRLDVVDFTLPLILSRNRIYFKKPDGTYIQWSAYFKTFKADVWSMIIFVIVTAPIFLTLMKTRGRIVMRILADNYI
YVWGIYCQQGLSEFPTKFSMRLAFLSIFLSSLIILSAYSASLTSFLTVSTVTLPFSTMEEFANYGSYKLITFRNSADYDMIIAANSSLFEKVKKLI
KDKEDLPLTAHDGFLQVCNEKVGFYITEAIKNAISIPCETSFIDADRVDSLALVLQKHGQYTGVVNYYLQQFKDNGVLARLKNTFLVTGDS
PEKGNVTVNLHGIAPILSVLAGGAIFSCLVLLLEKIYYNVWRNNCRGIFHCVLWRKFLNRQLVVDSVDKKKRIENLSENILQSQENQRSDIFR
NQSSFTQYK
>AmIR332
MKKLMLLVVTIHIFTKPVHSVNGVIWNKKLENFVPIFSVPTFATVDYEIKNQSRKEEITTFQGRRFRFACYEELNMIITEANGTRITGIIGEIWN
ILSEYLNFTLIPVVINDRSVGITNSQGKYERGLLKFMQENKTDVISKMGAYNVRRKISQFTIPLWKTYRLYVQQEVIHLPTWMLKLFSQKVW
YAILITYLSLSMCSYLSQAINSIIMRKNLKTDLRDHLFYNFGMICGQSYFPKNSYKSFRMIELWLGLFSCLIRTAFGALLIGYMSQTIFIPPFQD
LDSLLDETTYDILTLNGSLPYYLFNQKIFSVYEKASRLKRYIVMNSIEEMYKTICLSEKLYALYESEDVKMAREIYFCRLNPVGFSLFSSWIIS
GLSLNFKYKRSIDIGLLKLYEVGIMDLLKYRWIKSKNEEKEIIKISEPIILEQIYLILLIFGAGFLISFIILVFENLIFYCKN
>BtIR332
MIHQLLRPVRSVNGVIWDKKLQDFVPIFSIPAFAAVNNEVLSQSRGEEIYDFQGRIVRFSYYEEKNLINSVGNGTRITGIIGEIWNILSEYLNFTI
KPILTDEKSTGASDLDGMFRTGLLKYIADNKTDVIPRMEAHPKRLTITQMTMPLWKAEYRLYIEREVKHVPTWMVKLFSKRVWHAVLITY
LLLSMCSYLSHKVETRIMLKKLQTDLKDHFFYNFGMICGQSFFPSSSTRSSRIIELWLGLFSCLIRTAFSALLIGYMTQTTFTPPFNDIESLLDN
TSYKILTLNGSIPNLIIERGTSPVYKKVLDMKRYIVMDTIEEMYTKICTSNKPYTIFESEDLKKARGMYFCRLNPVGIPLLYSWIVPGISKTFTH
RRSIDIGMLKLYEVGFIKLLKHRWIESKNVEKETLNVTEPIILEQVYLTLMIFIGGLLISFVILLFENIIFYCKIKKIS

>BtIR333INT
MCVAKANTSKMLVTLFIIILRYCRVKCQMEKSIIWDKNQQDFVQLYNDSRFRDFRRQTISTRQDLHMKDISGKVLRASYYEEQDLVMFYEN
DTKVTGICGDIWNLLAYHLNFTWLLSMFTRETLCTHIFLILIFAVAGYIFQYGSRKNRKRNKQSKSRSGYFNFADHIFYTYSIMCCQGYLPRG
FCDQSKILSTSKFLFAWLMLLIFSSNLIYRMTNRSMTPPFVDFDTLLKQSKYNVLIFEGSIIYEFVKNAIHLPMYDNYKLSERIFFEKNLSFIYK
EVCSGKKLIATLENENKAYSRSKDFCPIVPVGKNYFQTWIGFGVPKRFPYKRSIDTSIIKLHEVGLIDILKDRWMNYRRNNIERSPFKRIDINQ
VYLIFEMLFIGIVLSLIILSLENLIFFCRKELT
>AmIR334
MSKLLLATLLHLTILFRVACKLSESDESTDSYSNLYPFLEKWARTSHTKTLTVVFDDYDLERHPFDPIPAEMLIDLNVSTKLVTLKHIISLKHK
DRRRDYRTIEAENCVLLLFSDVDHLKDILSSPHLISFWHPENFYILREQGGSSVSLFERERFCQWAFERLWRMRRVYKLLLFTGDKVIRYDPF
DYNRLRARYRVGGRDCDWYCDKSSEEAGFLLINRPNITDVSDFFEEEMIDFKRYPLKISIFETSTISVKDGRYFGLDFKYLDEVCKMMNVTR
SLIKSKDRFGWEENGTFFGTIGHLVYGFADVSFNQFFVKDYLTRQVEFTVSITSDKLCVLVPKAAPLPDYLVIVKIFTGRAWLLVFAAHFVIA
MIYTVLKIEKYRNMLEAVRKSGQAFFCCEYIPDSYFLEDRNGVVFNVVREETGEEDDERASYDGNTRKYPRFANNFFLFDGSENVRSTASK
TERNDSKRFYRAYLSLLITLARYLMKVIFQLMQPFKLGQPWFPERLLLICSLLLSLILNGIITSQLASSFSKRMYYEDINTLEQLEKSGITILTDA
KDIISDAFTDVSSPLIKRLHERLEYANRSEVHRRLFEVKDAGYLHRIATLPLKYDEYQRNSLHIVKECPKDYIIANVMKKGSPFGRRINTILLR
LNNGGFYKIWYQAMYQSLKRKEMMTLDDSSIHRKITIRHLFIPFGILYLGLATSVIVFIYEYRQNNV
>BtIR334
MRSKFPVMLLTATILFRVACELFEPNYSGNYSNLYPFVEKWAMTSYTKMLTVVFDDYEYHRAFDVPRDILIGLNVSTKLVSLKHAISLKYK
NMRRDYQTIETESCVLLLFSNVEHLRDILSSPHLMSFWHPENFYILQEQGLQFFDSSVYEKFCKWAFERLWRFRRVYKLLLFAADKVIRYDP
FDYAARHTGYSVNTSCDWNCVKSNKDGFLLINGPNTTDISDFFIKDRRDFKLSPLKISIFETSTISFQNGQFSGLDFKYLEEVCKMMNVTFVLI
RSKDRFGWEENGIFFGTIGHLVYKFADVSFNHFFIKDYSTRELEFTTPITSDKLCVLVPKAPPVPDYLVIFKIFTEGAWLLVFATHFVISMIYTI
LKNERFEAIRQSGTVLFCCEYLTGFYFLEKRNGGVVKIAPKFTKKRAYDEAHGIYLVEKTPNVRSTIKTKKGRSKRFNEICLSFVTWLGRYL
TKVVFQLMQPFKLGQAWFPERLLMMCSLFLSLILNGIITSQLASSFSKRLYYEDINTLEQLKESGLAILTDTRDVLDDALTDITSPIIKQLNDR
LIYANKSEVYRRLFKAKDAAYLHRLETLPFKYSEEEMESLHVVSECPKEYILANIITKGSPYGGRINNILSRLNNGGFYGKWYQSIYQSQKRS
ALVLNGSTMHRKITIRHLFIPFGILCIGLAMSIIVFIYECLQNNVR
>AmIR335
MHPIYFSFLILIEPCSSTRHSWFHDKLKSIENDNQLSKREIDNLGLVLNFIRQSPEFQPLAISVILTKFQSGPIDAVLRYVRENSIQTYKVSHAAR
DRPLKSFLRRKNDVEGITWLFTIDRFDVIFVYWEWHVWNSNNQYLIVFTERSQVGGIVVWRDVFRTLWKRYHAYRVIVIRADDDFRCMLR
YKPFERYGEDEYGVVYETCLGEGSKVEGDGRRLFENFQNLNHYPVNVSVFESLLMRISYDRGNRLKLDKIDAKALFVLEKAMRASFNIKA
VRKRNIKRDPYSFLINDIERGITDMCITGFFVKTYGRFQKFQFTPSLYEDKICFVMTNYGFIPNMYVFFFPFRARLWLVLITYNVVITLLWRLV
RHLSSLFRRRPTSSNDHRVNGNRAKGVNRGESVKPPEIPRIVEKFFTFFEYLCYPIRDSKYAAERALLTGILFFNLIISGLYQSFLVSSLNKPFH
YYQYHTVDEVVRSGKTMITKYENLKQAFVGSDLWEKIQVIEFQRSTKNIVLTEDKISMTRLYNMQLLPHRHDRGKSSLYMVDECAMTYRI
SYILKLHSPYAERVNFVLLRMAEAGLPEHWIDEMRYFVTLYNRGEMEDFVNIHKLSLGYYVLAFLLLFVGLLLSTLIFFCELQAAKRNRKR
R

>BtIR335
MLYSTYLIPLGILLLLKSSTLAEHSRNHDKFYSSIESKPLSSQETDNLRLVLNFIRQSPEFHPLTFSVILPEFQSSFIEFLLSYIGQNSIEAYKVRA
RDLSLKATWMNRLRLTWIFVVQDLSSFNIFVYWQWNLWKAGNQYLIIFTGKVVTTFWTEAFTNLWRKYDVHRIVVVDDDFRCLTRYMPF
EKRSNNGFGRVYKSCLEKNHFTKRSSEYDLLQNSPKDYNSGYPGEILRLNRDTRLFENFQNLNHYPLNVLVFESLLMNVSYDTRNRLKLSK
PDANVAFALEKAMGAKFRIKAMRKADFMDDPFTTSLTDIETRNVDMIITGFFVKVYSKFRKFQFTCAMYEDKLCFVSPDSGLVPKAYMPFL
PFQKSLWFLLIAYNITVTFLWCLVKHISESLRRQYKCANPSRRLEINRSTRKPHAWYLPKSGIKNYFPIKEHRSSDVSNYKEPPEIPRYIKNPIIF
IEYLTYPFQTSEIPAQRALLLGTLFFALILNGLYQSVLVSSLSKPFHYPQLHNLEDVVDSGKIVITKYANLKNVFLDDTELDATLAQRIRLINT
QRSTKDIVAYEDKIAITRYYSLELDDSDYFDKEGNPLLNLVDECPMNYRVSYVSRLHSPYAEKVDFVLLRLREAGLLNFWFDDMLYRIKIV
KIKKRRLKKDMAAMKSWMSDKIMLSLDHYLLTFLLLLVGLFGSTVMFFVELYMAKRSTRKC
>AmIR336
MIPPIYLLTLQQMNYLYQLPIIERSVKHLLDTSQFLNVLFVVQNSLDPLGDLDHLIDEIYKTVSRTIPTNLVSFNDSIPIELPEIDATESTLILYSY
VAKDFPDRRRREDIARFIEHCQNRSKVLLVTRLEEMNCNFEGFLKQIWYDELIDMTVLELSPSKRSITMNVHRYNPFANVYDRSPYTPTLD
WFPNKMTDLHGHPFRATVLEREGYINLTVDSRDYPVSYKGPDVKLLRTLARIMNFTIVMQPNNDALTSLMDGDLDIIIPKLPLFPDQRFDLL
DHTLPFEYEKWCPVVPITYQVNAIETRAFAAIIANIVILLAFWAVSALLRFERRLWQPLKIFGILIAASVSMRPGRTLERIVFFLVVLASTVYSA
NLYVDLTSVTMADTMETEYKSYEDLDESGLTPVVLHMIFNVTFFTDDQAFNSLKRKAIADEDMDDCTEVLSRYRNVTCFMELRGINALIYS
QARRNTATMKVCRNLCYAEPHATYFLRKHSPFRGKFDAIISRLEAAGIRKKWHYDFVGKFFPKKGRPVKRNLYESSLVWNLVYIGVIGFLA
SIVAFFCEILVYRAHKRRKNRA
>BtIR336
MMLPVYLLMLQQINYLNHLSNVEQTIIDVLRRIKFSDVQLVVQNPYGNNFGIINEIYKIVAQTIPTSYILVNDSDPLGLPSSQVSQATLVLYIY
VAKTSPNFEQTENITRAIVSKNRPKILLITMMEETDCNFEPLLKQTWHNHLIDIAILELSKLNRHTIATKVHQYNPFTDVYDQRPYVSGIELFP
NKMDNLHGYPIRIGMVKRAGYLWYMKNSQGYPVMYRGPDVKVIKTLARIMNFTIAVYPSQEVFVDIINEKIDMIIPTLSIFADANAVKCDFT
LPFGFESWCPVVPVKYKSNHAEIRGLIGIATNFCVLLIFWGLSVVLKFQSDLWQPLKIFGLLIATTISSRPKKTTERVIFFLIVLASSMYSANLFI
DLTNISMKEHTVIDYNSYKELDDTGLTPVILTGLLNVTFLNEDESSSRLKRNAIQMDNAESCANYLEQHKNITCFLETRSVNMIMYSHAREG
KRVLKICEKLCYAKPPSAYFLKKHSPYRDQFVKIIIRLDAAGIRMKWLNDYIGKFRPRKTHIMEVNSSYTLPLAWNLAYITCSGFLMSLLVFF
GEIIIHYLRRDKGTKKFLNRM
>AmIR337
MVQSSDALLLAGEYEDFFQRSLLDDIPVALISEAKSTAFELFTLSKYSFEIYTQFSFLYLIWKLNQFVIVASSQPLLRLLLQKTKDSGWSNFTG
FHILIDRRTVERGCVNAYNFLWAAWEYDRLSTIFLCIDPVEGLVLYTYNPYSSVAPRAWKNVGHFKGRSGHPWILLKQRYRDDLRLCENL
MFDKTRDLNGYEIRLNAISFEPHLQIDQSKPGLQKFTGDNSEIVKLVLVKLNASLNVRVYTGSVYDLGGVGKHGSMVGMMADVANGEVD
MGMNARSLNNMWKIEHTYPHGDDGLCVITQRAGEIAEFVKLWSFTAPIIHIGNFAIFTIALVILAKYQGFCPAFMNIIRMMTFVSIRKLPRTSS
CRIFFCSVFVLYLIVNALVQSHWASLLTIPVPLPNIRTAKDLKNSGYQIYGSMFHKLELQDPILRSRFHEDTYYACKEHVLRSRYAACLGDCQ
HQYVRIQNDYLYRSKKIQQNLQIYVARENWPLLPSVTEMIHRTVESGISSMWKKFNKRNMVRAWKKRQMNHKRSFRILEIKHIAFTFYALA
IGNFFAIVIFILEIIVGRKRVR

>BtIR337
MFHADDALLLAGEYDEFFQRSLLNDLPVALISESKSTAFEMFTLGKYSYTIYTQFSVIYLLWKLNQFIVVASSQPMLQLLLQRIKDSGWSNF
QGFHILIDRKTEQRGCVNAYNFLWTAWEYDRLSTIFLCIDPIEGIVLYTFNPYSSMAPEVWSNVGHFHGRSEHPWILLKKKYHDDWRTCED
LMFDKTKDLTGYEVRTNAISFEPHLQIDSTKRGLEQFSGDNSEILKIVFKKLNASLRVRVYTGSPYSLGGIGSHGTMVGMMADLATGEVDIG
MNARSLYNTWKVEHTYPHGDDGLCVFTQRAGEISEFVKIMSFLSPVIHAANAIVFVIALLVLTKYQGFVKASMNIIRLMTFGSVHRLPGTNS
TRIFFSSTFILYLIMNALHQSHWASFLTIPVSLPNIRTSEDLKKSGCQIYGSIFHGQELQDPELQSRFHKDTYYACKEHVLRSQCAACLGDCLH
HYVRIQNEVRLYRSKKIQQNALVFKTREDWPLLASVTQMIQRTVESGIIGKWQKASIRKTRWAWKKRQLNKNKSFKTLEMHHVLFSFYIL
GSGYLLGIVAFVVEIFMGRKRIDKSSRNRKH
>AmIR338
MKYTTKYIFLILIAQWQMLIVLSKLRITLDQFEILTKELSLVLSACKKIEVNNIGVVTDSRTFVQPSLKAVPLININDVDHLSRVERDQNLHTF
VVIPSTVEHLESTITKLERTTWWNPSALYVILDDNVARNGCKNARPFLKTAWNKDLLSSVFFCIMEHRNTEFRMYTFNPFTSRAPKSWQKM
REGTNETNKNRDHSWTLFTRTFHPGRTTCGNLDFAKTGTLGGYRMKGMGLHNPPSLTIDPSKGSGGKLGGFNGIITEILLSKLNGSMTVTGI
TNDTRSYKFLHLVASGKYDVFLNTQYVFNKPNITTTYPHVNSGISILTRYPDNEAVYVKVLKFMNPVFILCCAVVGVITVIILEVFVGRGMIH
ASLEMIRVALNNSMTRFPEQGALRVYLITVFLLFMLTSSTFQSNLSSLLTSSIPRLTIDSDEELKTSGFEIYAYHGYRNAVFDDVLFTRVKMV
DHWDCSEYVRKDRNVACAADRTTLLKIAFEKGLHLSKHRINTLFSAYVVRPNWPLKDRFTSMLLHLSETGLIDHWWEKVMAKYVHKWL
KRTEKRTEDEKRNFTVMTLNDLDFAFYILAFGLFASALSFLIEISTRRLRVTLERREKNDFPIFPFTL
>AmIR339
MESLFLRFLPLLLSASLCPSASAVVVVSSYHGLKTSYHGKGRKWGDRGFIVEGVTSAEKEREEFSRSMACIASFLDRATDPARTSFMISDDT
VDQAARSLARHLQKRRFAFLKYSGDSEFDTRELALNAVVLVANAASLRDDLYVIDPCTRDCPFFVVLARPFRSERAFLAEADALTRSMWT
RRIFTVVVLARVGESVLAAGATSFRPGQPCAPSPPIILDKCGTRSWNLTRSAIRPPKWNKCVLKVAYFVEPPYVVVATNGTTEESLLGFEGM
LTEEILKGESIEREEVAWTENATYVEQIRTILYQADADLVVGRVLLQPQEDIDYSSSYDVLKVVWLIPKVANVSLKGLIQPFHPYVWAALGC
TLVLAVLVKIFLIPDLAYLDIFALIIGASIANRPGKLSTKIQYISWSVFGLFLMEVYVDALADQLINTSDTKIATTEELVSSSFGIGGTSAFKSLF
EEDTSGVVSRIREKFVTFDQNEYVGLFNDVMEGRNSSIALMVLLNSSRSEDIDTLHAYTMSTDVIYSFPLALAVWKGFPKLKRINAKIHDYI
DMGIFDHMIKLAIQKGRLAMMFEFAEEQEYKNNLHLQHFAPAFLVMIIGFSVGFLSIIVEIALYPSDLFFPRKRREEGN
>BtIR339
MSLSRSCLRRFTVLLFVFTFLHLTRAIVVPQTYSDVYEKKNESGHRRLIIESMLDEENQREEFLTSIPCINSFLEKYIHRPGRISFVAIDNEESIQ
NIVQSFVRSLHNYFAIFKHTVDLDFEAHESALSTIVLMQNANSLEDNHYILEPCDRACPFITILISSFQDEESFLEHAGVVTQLMWSRRIATVVI
LAKVGDSVLAAGSLTFQPGKMCTVSPPVILDKCEDKSWSKLKKISAPKTNGCILKVAYFEQSPYVIVGNDSKRLSGFEGALTEEALRGQEIE
REKVVWNDNTSYAEQVQMYIYNDINADFVIGRILQQSYEDIDYSSSYDTSKVVWLVPKVPNVSLKGLVQPFQHYVWAAVGGSILLGCVVK
IFLFRDLSFLDIFALIIGVSTARQPTRLSTKIHFLAWCIFGLFLTQLYVDSLADQLINMSDLKFETMKELISSSFQIGGTAAFARLFEIFDQDEIVK
SVRKKFVVFDQDEYVRQYYDLLDGTNSSFALVIVLNSSRSGAIETTQAYTITTDVICSFPLALATWKGSPHLEHLNGEINKYIDFGILDFLIQI
ALEKNLRAMSSQTAQDEEYKTELHLQQFVPAFLLVAIGFSSGFLFIILEVVLYPSKLLQ

B. terrestris, A. mellifera and _D. melanogaster_ Ionotropic receptor (IR) phylogenetic tree

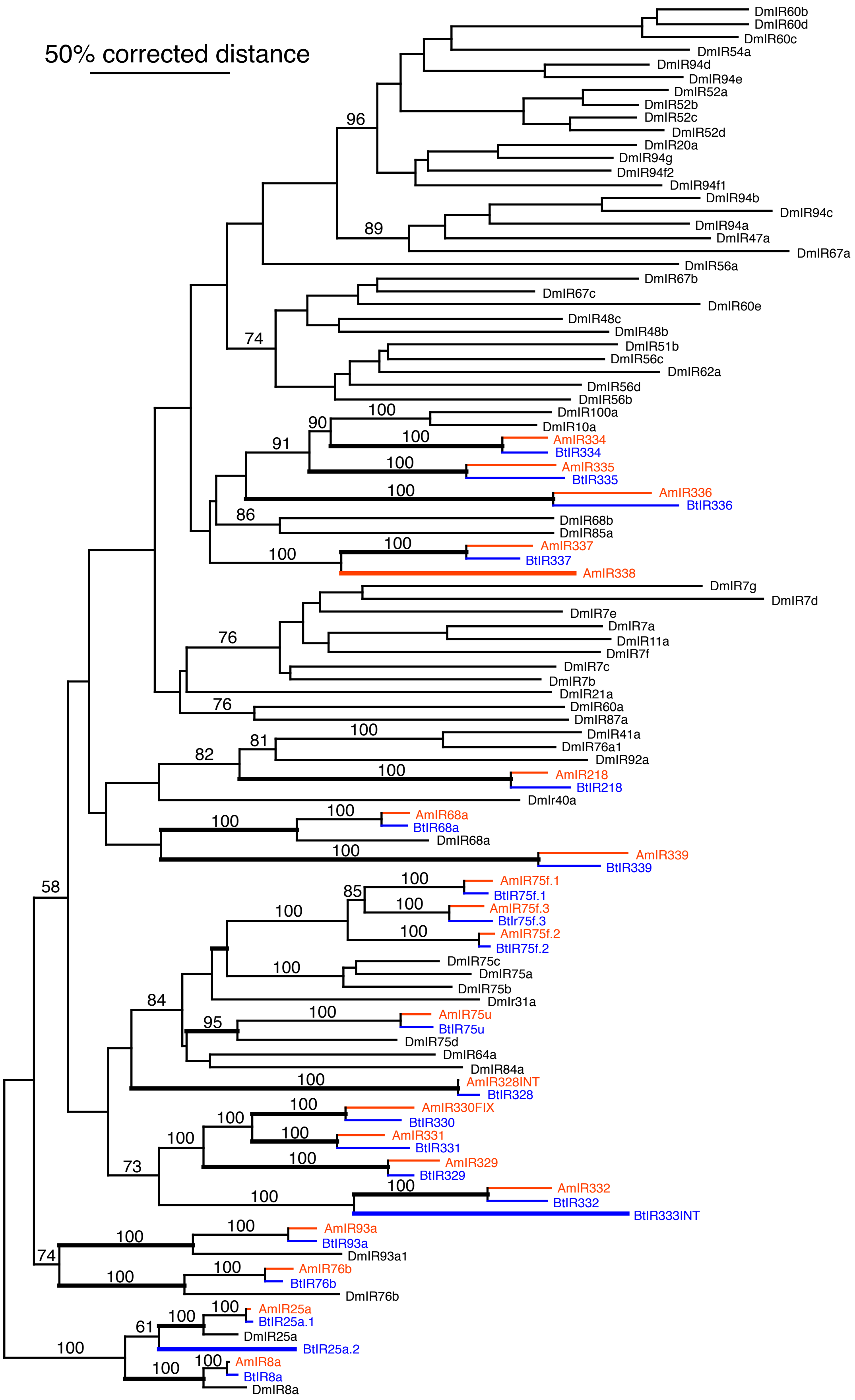

Phylogenetic tree of the *B. terrestris*, *A. mellifera* and *D. melanogaster* IRs. This is a corrected distance tree and was rooted with IR8a/25a as the outgroup, based on their highly conserved sequences and ancestral position in the family. *B. terrestris*, *A. mellifera* and *D. melanogaster* gene/protein names are highlighted in blue, orange, and black, respectively, as are the branches leading to them to emphasize gene lineages. Bootstrap support level in percentage of 10,000 replications of uncorrected distance analysis is shown above major branches. Inferred ancestral and orthologous lineages are highlighted in double thickness. Suffixes after the gene/protein names are: INT – internal sequence missing; FIX – sequence fixed with raw reads.

Odorant Binding Proteins

Details of *B. terrestris* OBP family genes and proteins. Columns are: Gene – the gene and protein name we are assigning (JOI indicates a joined gene model across two scaffolds); Refseq protein number (prefix XP_00); Scaffold – the v1.0 genome assembly scaffold name (prefix GROUP); Coordinates – the nucleotide range from the first position of the start codon to the last position of the stop codon in the scaffold; Strand – + is forward and - is reverse; Introns – number of introns; AAs – number of encoded amino acids in the protein; Comments – comments on the OGS gene model and repairs to the genome assembly. JOI – gene model joined across scaffolds.

Gene	Refseq	Scaffold	Coordinates	Strand	Introns	AAs	Comments
OBP1JOI	3393962.1	UN544, 2.3	<1-7047, <1-2506	-, +	5	141	First exon in separate scaffold
OBP2	3397921.1	9.5	476155-477709	-	4	142	Fine as is
OBP3	3397912.1	9.5	472699-474392	-	5	146	Fine as is
OBP4	3397906.1	9.5	468253-470320	-	4	136	Fine as is
OBP5	3397877.1	9.5	465406-466652	-	4	136	Fine as is
OBP6JOI	3397894.1	9.5, UN3981	462424-464218, <1-965>	-	4	142	Second exon in separate scaffold
OBP7	-	9.5	457824-460037	-	4	146	New gene model
OBP8	3397865.1	9.5	455718-457043	-	4	137	Fine as is
OBP9	3397885.1	9.5	451528-452709	-	4	135	Fine as is
OBP10	3401479.1	15.6	5517044-5519625	+	4	147	Fine as is
OBP11	3401480.1	15.6	5521216-5522898	+	4	133	Fine as is
OBP12	3402792.1	UN637	1234-3000	-	4	136	Fine as is
OBP13	3401135.1	15.5	2870640-2871523	-	4	132	Fine as is
OBP14	3398556.1	10.1	9186283-9187517	-	5	152	Fine as is
OBP15	3398555.1	10.1	9184397-9185245	-	5	143	Fine as is
OBP16	3399482.1	12.1	2267493-2268250	-	4	152	Fine as is

Protein sequences of predicted *B. terrestris* odorant binding proteins from the table above

>BtOBP1JOI
MGGKQALLYLGLVCLQTIFVNAVPDWLPPEIFDMVAEDKARCMSEHGTTQAQIDEVDKGVLKDDTSITCYMFCLLEAFSLVDDEGDIDAD
MLLGLLPDQLQARAESVMSKCTPAPGSDKCDKIYNLAKCAMAEAPDVWFIV
>BtOBP2
MHVKLLLVAMLFAFVAWKPAESATAEQMDKMARSLRRSCLQKIDISEDKVEGMRRGEFPDDPNLKCYTNCIMKLIRTFKNGNIDFAMVIK
QVELSMAPEEAAVMKEAILKCSHMEYTGDECQKSYTFVKCTYDTNPERFFFP

>BtOBP3
MKGVGVCLLVSLMLVLLAIKDTESKKMNLDELKKSIKNLRKPCSKKNDTPKELLDGQHNGEFPKDERLMCYMKCVLTQTKAMKGDEIM
WDFFVKNARVMILDEYFPRITHLVETCKNSVTATEGCEAAWQFSTCTYATDSELYIIP
>BtOBP4
MRVSLAICCLFFVAGAICDDKAEEFIKAFKEDIIPCLQEVEIPEDQIAKFVDNDLEGEERIKWGCVKACVMKRINIMSDGQMQMDNIKEIMD
KRFDKDSESTTEDMDQIKKCVEEVAGKTDECEIAFEFSRCMTPA
>BtOBP5
MKSLLVGICLLLTITVIQADFIDTYIELSKVPTLKCMKTVGYTETDPKVIFEQEVKLGVDKATCLRSCILKSMNMLKDSKINLDMINEFIKIVH
NEEPEKIEPMKKNAVECLDKVKDMSDDCKMAYAFIQCYVDKY
>BtOBP6JOI
MKTLVTFTCLLAAVTIVRGMDQDAVIGKYMEYLMPDIKPCADELNIAEDTATNIQQPKADADIRKMGCLKVCVMKRMNVLKGTDFNMEP
VYKMIEIVHAGNDEDIKLVKNIANECSAGTKGETDECNIGNKYVDCYIEKLFN
>BtOBP7
MKGYLAILVHVLSANILARASNLIQLTKMLNIDIKDIRQCLHQSNLTDTDLIKLDVIFQAKNITRENLEDVMLDSGCFIICVLDKAHMVEDKN
IRFEYLLETAERNNFPIATNQTLNECCKKAQEQDDICKSGFVFATCSLRQSGL
>BtOBP8
MKTVAALFCVLYFVCCGVTANEMSDTCLKQMGLTIADLPSLAHDNSPEVARKRGCLEACVMQKMGLMNGNVINTQKIDELLDNILPDGE
NKEIVRNNVHQCAKDAANDDQCLVAQNFAQCGLEHLKLTARQLLSTIA
>BtOBP9
MKTAVALFCVLYFVCCGVTASEMSETCLKQMGLSVFDLGNLIRDNTPEGIRTRGCFEGCLMQKIGLMDGNVINLRKIDEQIDRAYPDSEKK
DIIRDTVHQCAYSAANDDQCVVAQNFGRCGLDQLRFAELAHRFT
>BtOBP10
MKILAIVFAFCFVGALALSDHKKAQLARFRTSCAIETSINLQLIEDVKRQTIPMNDGRLGCFTYCILRKMGIVEENGNLWIKNNVAKHQLIM
WGSPWHTANKLVKECQNINGANECKKASNLVGCFMKNKSSYKRLLHITKLLSSNE
>BtOBP11
MKILAIVFAFCFVGTLAFSNIVKAKVERFRASCITETSIDPNLIQGAKKGIISKADERLGCYTYCMLRKKGIVEENGHLWVNIAKRQLITYGA
DALMADEMVNKCKNVAGGNECKQASNLIQCFLEIRSSYIF
>BtOBP12
MRSPAIIFAFCLVGALALTEDQKAKLEEYRTACTTESGVDPQVVENAKKGNVAQDDEKLACFSFCMLRKIGIMDEDGDIKEDVAKEKMVA
GGSPADKVDNVVSNCKHITGPNKCKKAGNLMKCFLENKSFNVLESN
>BtOBP13
MLRNCHFFLVLSTIIILLHFSKADIRKDCRRESKVSWAALRRMKAGDLEQEDQNLKCYLKCFMMRHGILDKNAEVDVQRALRHLPRSMQD
SSKKLFNKCKSVQSDDPCDKAYKMIKCYVEYHPEILQSVPFL
>BtOBP14
MQIMNYTVLLSLLIVCWIRLPVVRCGTRPSFVSDEMIATAASVVNACQTQTGVATVDIEAVRNGQWPETRQLKCYMYCLWEQFGLVDDK
RELSLNGMLTFFQRIPAYRAEVQKAISECKGIGKYLAKGDNCEYAYRFNKCYAESSPRTYYLF

>BtOBP15
MRAAAVWMSLICLLTLRFVFVHGGTISSDPDDFRRMTSVHRKKCIVESKTTLEAIEATEYGEFPDDANLKCYFKCVLEKFNMMDKNGKIRY
NILKQVIPNVYKEIGHEMIDSCKDVDAEDKCEKTFMFMKCMYNVNPIAFIAP
>BtOBP16
MKNSAFLASFILYWGLFSQNVIVLHAERPPFPPEIIHDCMDRENITTKELMILDSLPIETIKLLGSNREDFRNIGCFLACAFQQHGDMLGYTMN
LQTMMGRMHGMHHGHMDANPSFANVLQTCSDSVGGTDDECEAALTFHVCLMEAFHNRS

***B. terrestris* and *A. mellifera* Odorant Binding Protein (OBP) phylogenetic tree**

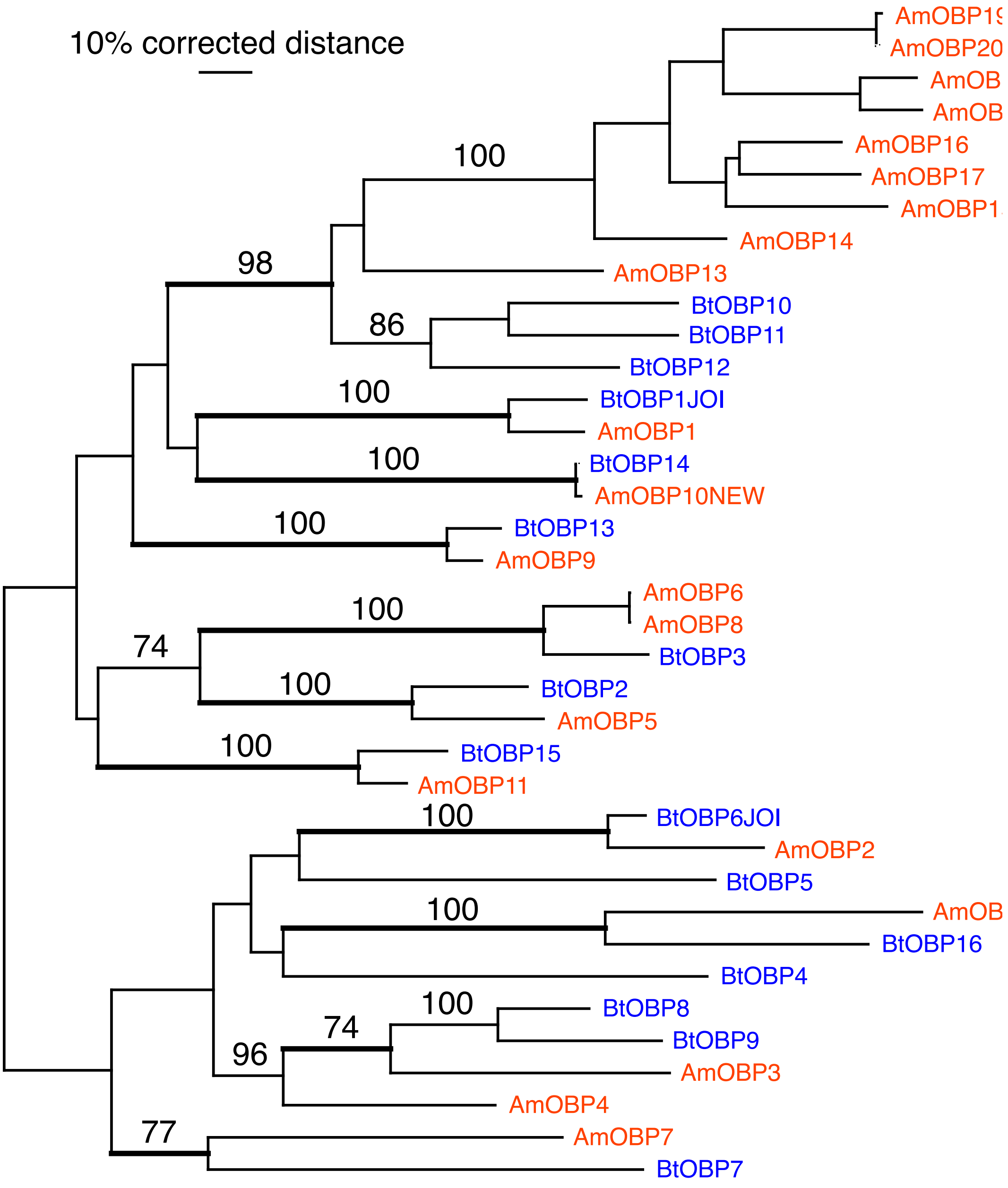

Phylogenetic relationships of the *A. mellifera* and *B. terrestris* odorant binding prot[illegible] corrected distance tree rooted at the midpoint. Percent bootstrap support from 10,000 ite[illegible] uncorrected distance analysis are shown above branches. *A. mellifera* proteins are in oran[illegible]

Hypothetical proteins identified within *B. terrestris* queen hemolymph. Functionally annotated *B. terrestris* hemolymph dataset was parsed for the presence of proteins annotated as hypothetical. Identified proteins were searched against the NCBI nr database using Blast2Go to identify level of conservation across phylogenies. For each protein, assigned BterHAP term, NCBI RefSeq protein_ID, functional description, sequence length (amino acids), presence of functional domains, predicted signal peptide domain and previously identified homologue within the hemolymph of the honey bee, *A. mellifera* (Chan et al., 2006), are shown.

BterHAP	Protein_ID	Functional description	Sequence length (aa)	Functional domain analysis	Predicted signal peptide domain	*Apis* hemolymph homologue
			***Bombus*-specific**			
**BterHAP6**	NA (au6.g3851.t1)	NA	102	Transmembrane domain (TMHMM)	1-23	-
**BterHAP4**	XP_003396531.1	PREDICTED: hypothetical protein LOC100650927	188	-	1-16	
			**Apidae**			
**BterHAP13**<br><br>**BterHAP14**	XP_003400053.1<br><br>XP_003402621.1 (au6.g8405.t1)	PREDICTED: hypothetical protein LOC100647918	93	Transmembrane domain (TMHMM)	1-21	
			**Apoidea**			
**BterHAP7**	XP_003397710.1	PREDICTED: hypothetical protein LOC100646348	796	-	1-21	
			**Hymenoptera**			
**BterHAP9**	XP_003398056.1	PREDICTED: hypothetical protein LOC100649338	676	-	1-19	XP_397488.1
**BterHAP2**	XP_003394564.1	PREDICTED: hypothetical protein LOC100647892	476	-	1-20	
**BterHAP13**	XP_003401022.1	PREDICTED: hypothetical protein LOC100646603	166	Transmembrane domain (TMHMM)	1-27	
**BterHAP5**	XP_003396646.1	PREDICTED:	100	Transmembrane domain	1-25	

		hypothetical protein LOC100644966		(TMHMM)		
**BterHAP1**	XP_003393509.1	PREDICTED: hypothetical protein LOC100644671	281	**-**	**-**	XP_001121746.1
**BterHAP3**	XP_003395760.1	PREDICTED: hypothetical protein LOC100644638	81	-	1-19	
**BterHAP11**	XP_003399422.1 (au6.g5771.t1)	PREDICTED: hypothetical protein LOC100648951	1111	**-**	1-15	
			**Hexapoda**			
**BterHAP8**	XP_003398320.1	PREDICTED: hypothetical protein LOC100652008	177	Prokaryotic lipoprotein (PS51257) Transmembrane domain (TMHMM)	1-20	
**BterHAP10**	XP_003398591.1	PREDICTED: hypothetical protein LOC100644182	153	Transmembrane domain (TMHMM)	1-23	
**BterHAPA1**	XP_003393257.1	PREDICTED: hypothetical protein LOC100652150 isoform 2	318	Fn-I-like domain (SSF57603)	1-27	XP_001120895.2
**BterHAPA2**	XP_003393266.1	PREDICTED: hypothetical protein LOC100643149	327	Fn-I-like domain (SSF57603)	1-42	XP_001120895.2
**BterHAPA3**	XP_003393186.1	PREDICTED: hypothetical protein LOC100643108	316	Transmembrane domain (TMHMM)	1-28	XP_001120895.2

Overview of the genes coding for RNAi core machinery proteins in various insect species.

Gene family	*D. melanogaster*	*T. castaneum*	*B. mori*	*A. mellifera*	*B. terrestris*	*B. impatiens*
**RNAse III**	dicer-1<br>dicer-2<br>drosha	dicer-1<br>dicer-2<br>drosha	dicer-1<br>dicer-2<br>drosha	dicer-1 dicer-2 drosha	dicer-1 dicer-2 drosha	dicer-1<br>dicer-2 drosha
**Argonaute**	argonaute-1<br>argonaute-2<br>argonaute-3<br>aubergine<br>piwi	argonaute-1<br>argonaute-2<br>argonaute-3<br>aubergine<br>piwi	argonaute-1<br>argonaute-2<br>argonaute-3<br>aubergine	argonaute-1<br>argonaute-2<br>argonaute-3<br>aubergine	argonaute-1<br>argonaute-2<br>argonaute-3<br>aubergine	argonaute-1<br>argonaute-2<br>argonaute-3<br>aubergine
**Genes coding dsRNA binding proteins**	pasha<br>R2D2<br>loquacious	pasha<br>R2D2<br>loquacious	pasha<br>R2D2<br>loquacious	pasha<br>R2D2<br>loquacious	pasha<br>R2D2<br>loquacious	pasha<br>R2D2<br>loquacious

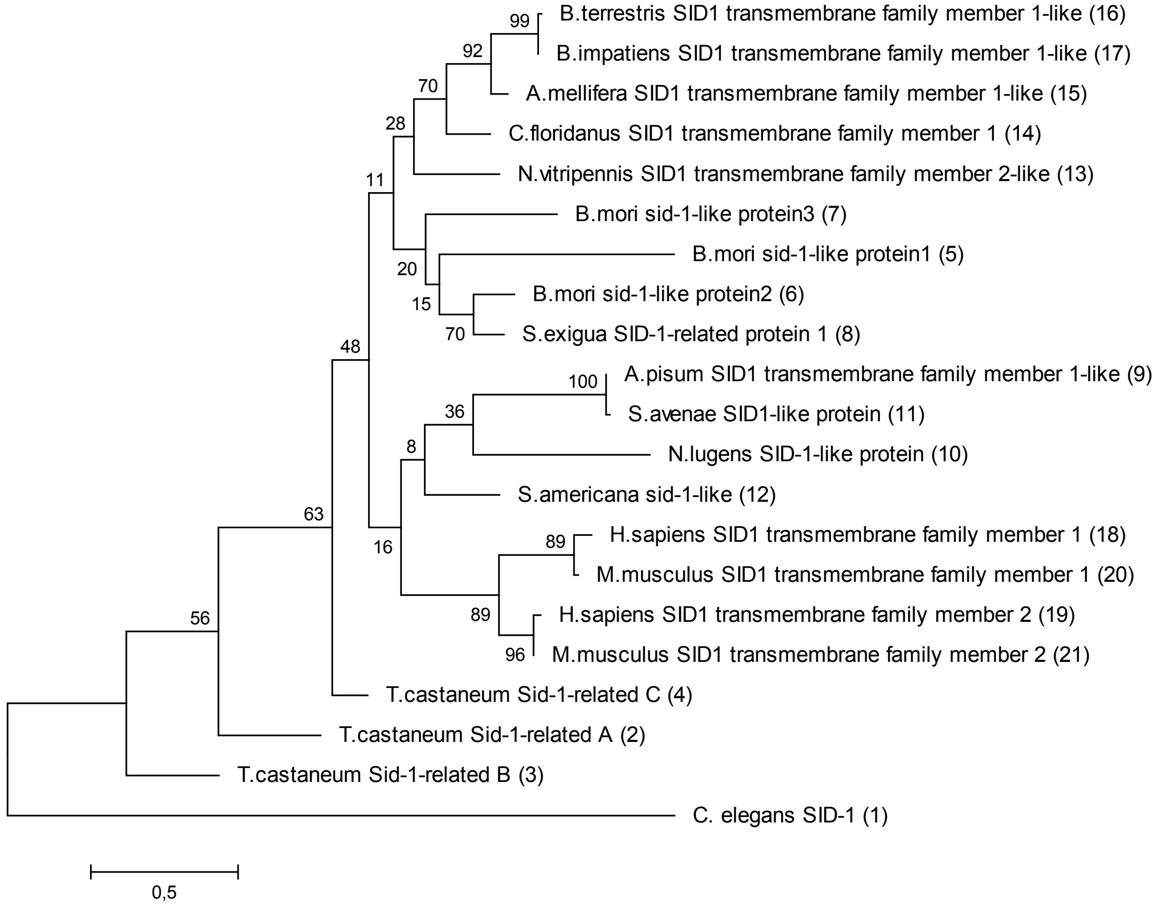

Phylogenetic tree for 21 SID proteins sequences using a Maximum Likelihood method. The numerical values are the bootstrap values after 250 replications. The scale bar represents the number of nucleotide substitutions per site. Accession numbers: (1) NP_504372.2, (2) ABU63672, (3) ABU63673.1, (4) ABU63674.1, (5) BAF95805.1, (7) BAF95806.1, (8) ACM47363, (9) XP_001951907, (10) ADI88514, (11) ABP98805, (13) XP_001605484, (14) EFN66381, (15) XP_395167.4, (16) annotated XP_003399893, (17) annotated sequence that matches XP_003493581, (18) NP_060169, (19) NP_001035545, (20) NP_001152891, (21) NP_758461.

DNA methylation

The *B. terrestris* and *B. impatiens* genomes contain DNA methylation genes. Both the *B. terrestris* and *B. impatiens* genomes contain genes needed for the placement and removal of DNA methylation. This table includes the annotated gene names for the corresponding enzymes needed for de novo DNA methylation (DNMT3), the maintenance of DNA methylation during cell replication (DNMT1), targeting of DNA methylation (PIWI), and DNA de-methylation (TET).

	DNA methyltransferase		tRNA methyltransferase	DNA methylation targeting	DNA de-methylation
	DNMT1	DNMT3	DNMT2	PIWI	TET
B. terrestris	bter_dnmt1a, bter_dnmt2a	bter_dnmt3	bter_dnmt2	bter_aubergine	bter_tet1, bter_tet2
B. impatiens	bimp_dnmt1a, bimp_dnmt2a	bimp_dnmt3	bimp_dnmt2	bimp_ago3	bimp_tet1, bimp_tet2

Methylated genes in *B. terrestris* and *B. impatiens* have the same distinct functional enrichment. Similar to *A. mellifera*, the most significant gene ontology terms (top 5 shown) for methylated bumblebee genes were related to metabolism and ubiquitous housekeeping functions.

GO biological process term	Accession	Fold enrichment (*B. terrestris*)	P-value (*B. terrestris*)	Fold enrichment (*B. impatiens*)	P-value (*B. impatiens*)
Cellular process	GO:0009987	1.29	7.79E-91	1.30	6.12E-105
Cellular metabolic process	GO:0044237	1.50	1.34E-85	1.52	3.60E-100
Primary metabolic process	GO:0044238	1.32	1.89E-43	1.34	1.85E-53
Metabolic process	GO:0008152	1.25	2.09E-38	1.27	1.10E-47
Macromolecule metabolic process	GO:0043170	1.33	6.75E-36	1.36	3.90E-45

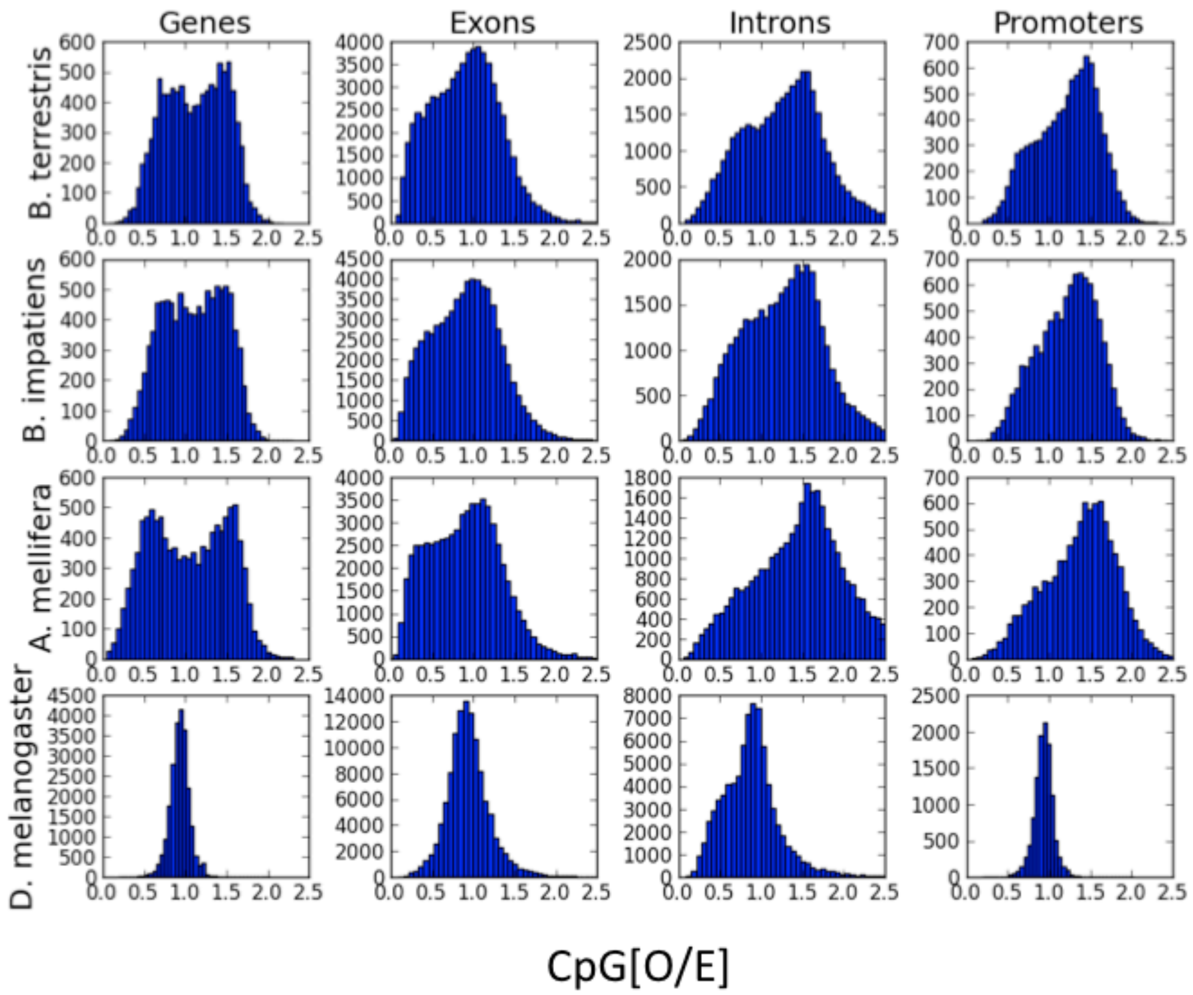

DNA methylation is used on a genome-wide scale in *B. terrestris* and *B. impatiens*. DNA methylation in the germline results in CpG depletion through nucleotide deamination. The CpG[O/E] value (=#CpGs observed/#CpGs expected) was used to predict the presence of DNA methylation in a genomic region. The CpG[O/E] distributions are shown for genes, exons, introns, and promoters. The similarity of the CpG[O/E] distributions between the bumblebee and *A. mellifera* genomes indicates that bumblebee DNA methylation is likely targeted to approximately half of all genes and is primarily confined to exons. The lack of bimodality in the promoter regions indicates that the bumblebee genomes lack the promoter DNA methylation that is functional in mammals. The CpG[O/E] distributions for the *D. melanogaster* genome, which contains no measurable DNA methylation, is shown for comparison.

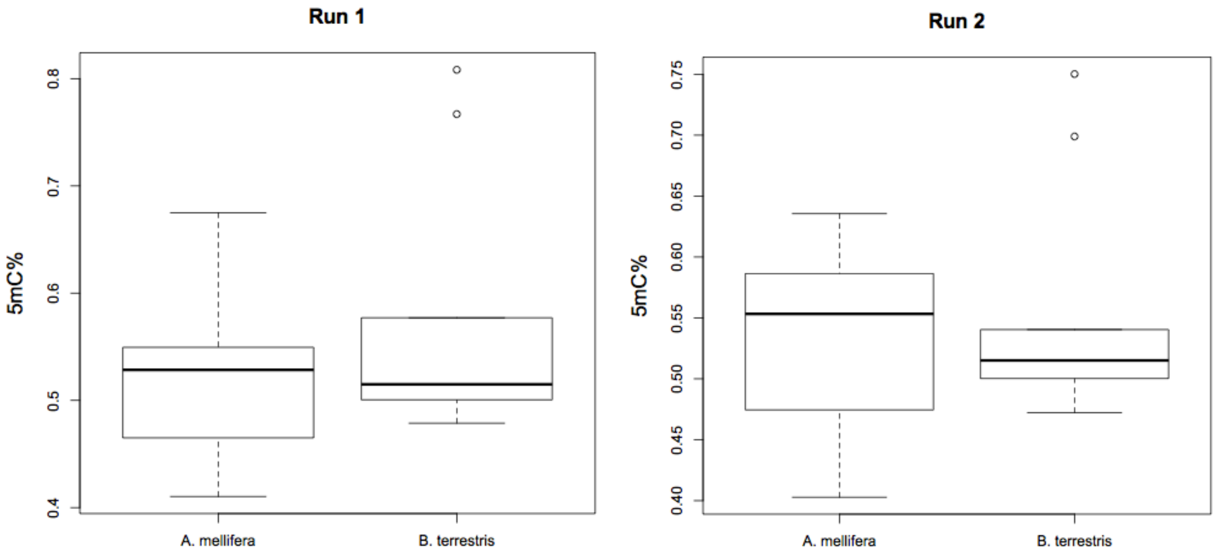

Genomic DNA methylation levels are approximately the same in bumblebees and honeybees. Global DNA methylation patterns in *B. terrestris* and *B. impatiens* using the MethylFlash DNA quantification kit (Fluorometric) from Epigentek. Thoraxes of newly emerged *B. terrestris* and *B. impatiens* workers and collected similar *A. mellifera* samples as controls were used. The two ELISA runs from the MethylFlash kit show that the global 5mc% levels (=% of all cytosines that are methylated) in *B. terrestris* (Run 1 avg. = .573%, Run 2 avg. = .555%, N=9) are similar to *A. mellifera* (Run 1 avg. = .523%, Run 2 avg. = .530%, N=9). Similar results were found for *B. impatiens*.

B. impatiens SNPs

Table of samples used in SNP identification

B. impatiens RADseq sample information. Samples from Cameron et al. (2011).

BAM/fastq File Prefix	State	County	Long.	Lat.	No. Sequence Reads	SRA Acc. No. for BAM alignments
UAlabama_JLozier_Bombus_AL09.0090_sequence_1	Alabama	Bibb	33.05	-87.01	2,570,235	SRR1702551
UAlabama_JLozier_Bombus_AR08.0468_sequence_1	Arkansas	Washing-ton	35.82	-94.16	5,114,961	SRR1710771
UAlabama_JLozier_Bombus_CT09.0749_sequence_1	Connecticut	Hartford	41.77	-72.90	1,738,534	SRR1710772
UAlabama_JLozier_Bombus_IA09.0278_sequence_1	Iowa	Crawford	41.99	-95.39	3,997,417	SRR1710773
UAlabama_JLozier_Bombus_IL09.0331_sequence_1	Illinois	Ogle	41.98	-89.36	4,102,329	SRR1710774
UAlabama_JLozier_Bombus_IL09.1316_sequence_1	Illinois	Peoria	40.83	-89.80	7,417,713	SRR1710775
UAlabama_JLozier_Bombus_IN08.0789_sequence_1	Indiana	Montgome-ry	39.96	-87.07	2,998,470	SRR1713809
UAlabama_JLozier_Bombus_IN09.0200_sequence_1	Indiana	Ripley	39.07	-85.44	6,634,261	SRR1713946
UAlabama_JLozier_Bombus_KS09.0737_sequence_1	Kansas	Osage	38.64	-95.60	3,311,551	SRR1713811
UAlabama_JLozier_Bombus_KY09.0177_sequence_1	Kentucky	McCracken	37.03	-88.76	3,079,121	SRR1713813
UAlabama_JLozier_Bombus_KY09.0915_sequence_1	Kentucky	Franklin	38.16	-84.94	3,237,837	SRR1713814
UAlabama_JLozier_Bombus_MN08.0990_sequence_1	Minnesota	Winona	43.98	-91.43	6,125,072	SRR1713815
UAlabama_JLozier_Bombus_MO09.0593_sequence_1	Missouri	Franklin	38.48	-90.82	16,591,141	SRR1713816
UAlabama_JLozier_Bombus_NC09.0842_sequence_1	North Carolina	Stokes	36.47	-80.39	1,584,066	SRR1713865
UAlabama_JLozier_Bombus_NY09.0009_sequence_1	New York	Seneca	42.68	-76.85	4,305,873	SRR1713866
UAlabama_JLozier_Bombus_OH09.0001_sequence_1	Ohio	Belmont	40.00	-81.14	4,725,157	SRR1713879
UAlabama_JLozier_Bombus_PA09.0299_sequence_1	Pennsylvania	Cameron	41.40	-78.03	3,798,097	SRR1713880
UAlabama_JLozier_Bombus_SC09.0723_sequence_1	South Carolina	Kershaw	34.16	-80.57	1,334,241	SRR1713881
UAlabama_JLozier_Bombus_TN09.0034_sequence_1	Tennessee	Cocke	35.92	-82.98	5,086,922	SRR1713882
UAlabama_JLozier_Bombus_VA09.0646_sequence_1	Virginia	Appomatt-ox	37.26	-78.68	1,133,079	SRR1713883
UAlabama_JLozier_Bombus_VT09.0166_sequence_1	Vermont	Windsor	43.41	-72.71	3,618,134	SRR1713884
UAlabama_JLozier_Bombus_WI08.0504_sequence_1	Wisconsin	Dane	43.04	-89.43	3,168,180	SRR1713885

RAD sequence data in the form of BAM alignments to AEQM02.fasta have been uploaded to Genbank Sequence Read Archive under Project Accession No. SRP051027, and SNP data in vcf format is available from the DRYAD digital repository: http://dx.doi.org/10.5061/dryad.52hj2

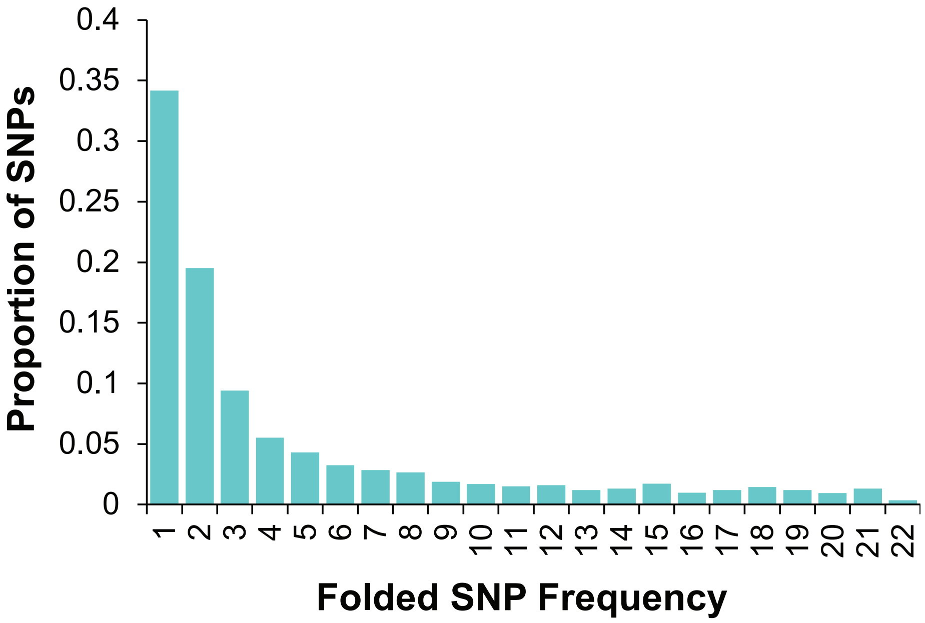

Likelihood-based site frequency spectrum estimate for *B. impatiens* RAD tags. For the 1.113 x 10^6 sites examined θ_{pi} = 0.0014.

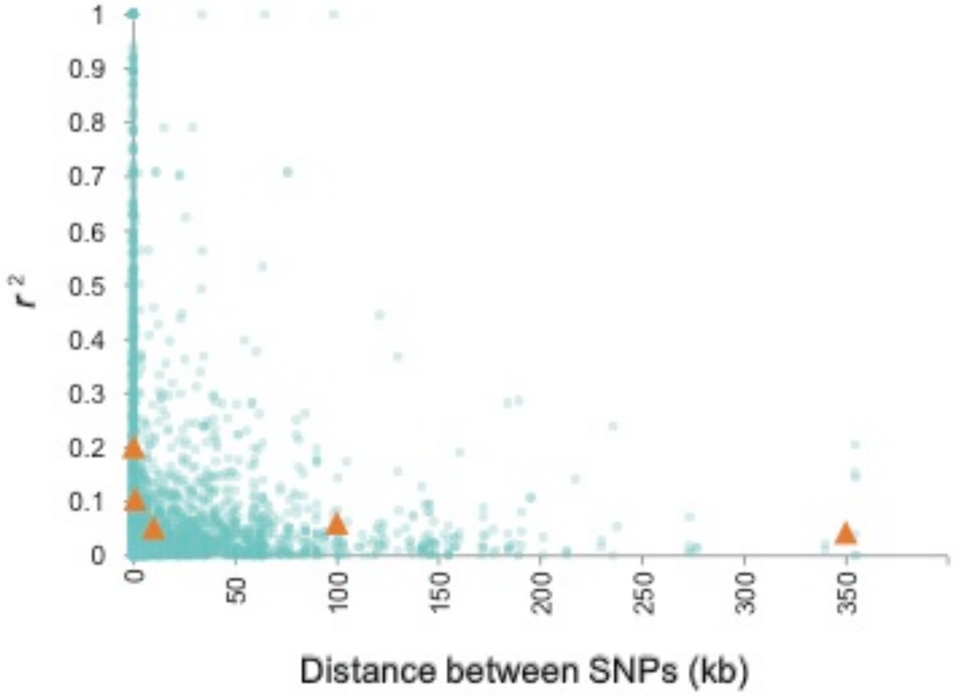

Linkage disequilibrium decays rapidly with intra-scaffold distance among RAD-tag SNPs (minor allele frequency >0.05) in *B. impatiens*. Average r^2 for logarithmic distance bins shown as triangles.
